# Oxidative rearrangement of alkynes to chiral α-arylalkanoic esters

**DOI:** 10.1039/d5sc07882b

**Published:** 2025-11-19

**Authors:** Rawiyah Alkahtani, Johannes Westphäling, Aleksandra Gorecka, Rasool Babaahmadi, Hanaa Gieman, Maylis Finance, Jaime Lorente-Martinez, Dhananjay Bhattacherjee, Rebecca L. Melen, Mu-Hyun Baik, Thomas Wirth

**Affiliations:** a School of Chemistry, Cardiff University Park Place, Main Building, Cymru/Wales Cardiff CF10 3AT UK wirth@cf.ac.uk; b Chemistry Department, College of Science, Princess Nourah bint Abdulrahman University 11671 Riyadh Saudi Arabia; c Department of Chemistry, Korea Advanced Institute of Science and Technology (KAIST) Daejeon 34141 Republic of Korea mbaik2805@kaist.ac.kr; d Center for Catalytic Hydrocarbon Functionalizations, Institute for Basic Science (IBS) Daejeon 34141 Republic of Korea; e School of Chemistry, Cardiff Catalysis Institute, Cardiff University, Translational Research Hub Maindy Road, Cymru/Wales Cardiff CF24 4HQ UK melenr@cardiff.ac.uk

## Abstract

Chiral α-arylalkanoic esters, valued as anti-inflammatory agents, are synthesised through an enantioselective oxidative rearrangement of alkynes under green, metal-free conditions. This study achieves this transformation using chiral iodine(iii) reagents with *para*-toluenesulfonic acid and various alcohols, producing the esters in up to 91% yield and 99% enantiomeric excess. The scope of the reaction particularly includes electron-rich non-terminal arylalkynes. Density functional theory calculations give insight into the origin of enantioselectivities of this process.

## Abstract 2

A green, metal-free method achieves enantioselective oxidative rearrangement of alkynes to chiral α-arylalkanoic esters, giving up to 91% yield and 99% ee. Chiral iodine(iii) reagents convert electron-rich arylalkynes, with DFT explaining the high selectivity.

## Graphical abstract

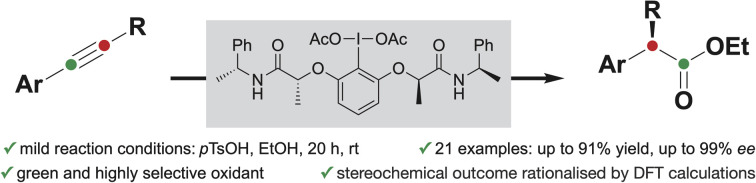

## Introduction

The facile generation of cationic species as reactive intermediates in organic synthesis is an area in which hypervalent iodine reagents have proven exceptionally useful. These intermediates can react directly with nucleophiles^1–6^ or undergo rearrangements such as aryl migration,^7–9^ ring contractions^10–12^ or ring expansions.^13,14^ Alkenes have long served as precursors in such transformations, as their reaction with iodine(iii) reagents readily affords cationic intermediates; ketones have also been employed in related rearrangements.^15,16^ Alkynes are likewise known to react with hypervalent iodine compounds to generate alkynyl- or alkenyl-substituted iodine(iii) species.^17,18^ While these reagents have been primarily used in alkynylations or alkenylations, we report here a distinct approach: the activation of alkynes with iodine(iii) reagents to synthesise α-arylalkanoic esters through a rearrangement pathway.

**Fig. 1:**
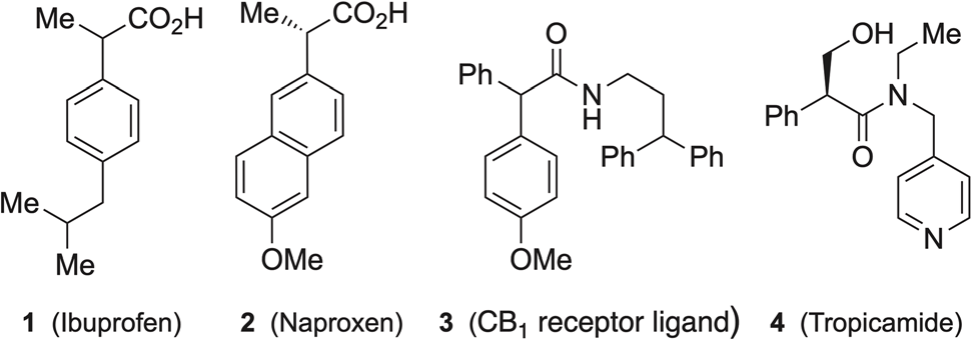
Drug molecules with α-arylalkanoic acid motif.

α-Arylalkanoic esters bearing an α-stereogenic centre as precursors of α-arylalkanoic acids are both synthetically challenging and of considerable importance as intermediates and functional motifs in medicinal, pharmaceutical, agrochemical, and natural products chemistry. Representative examples are shown in Fig. 1. Ibuprofen 1 and naproxen 2, members of the profen family, are widely used non-steroidal anti-inflammatory drugs.^19–21^ The α-arylalkanoic acid motif also occurs in the cannabinoid CB_1_ receptor ligand 3 ^22^ and the mydriatic drug tropicamide 4.^23^

Numerous synthetic strategies for α-arylalkanoic acids and esters have been reported, including Cu-catalysed carboxylation of arylalkenes with CO_2_ in the presence of chiral ligands,^19^ Pd-catalysed enantioselective Markovnikov hydroxycarbonylation of vinyl arenes with CO and water,^24^ Au-catalysed arylation of diazoesters,^25^ Tl-catalysed oxidation and 1,2-migration of aryl ketones in alcohol,^26^ Ti-catalysed hydromethylation of α-alkene esters,^27^ and Ir- and Ru-catalysed α-methylation of aryl esters with methanol as the methylating agent.^28,29^ Many of these methods suffer from unfavourable reaction conditions, including the need for moisture and air-free environments, the high cost and toxicity of metals, and the risk of heavy-metal contamination particularly in drug products. Consequently, there has been growing interest in metal-free approaches that operate under milder and more sustainable conditions.

**Fig. 2:**
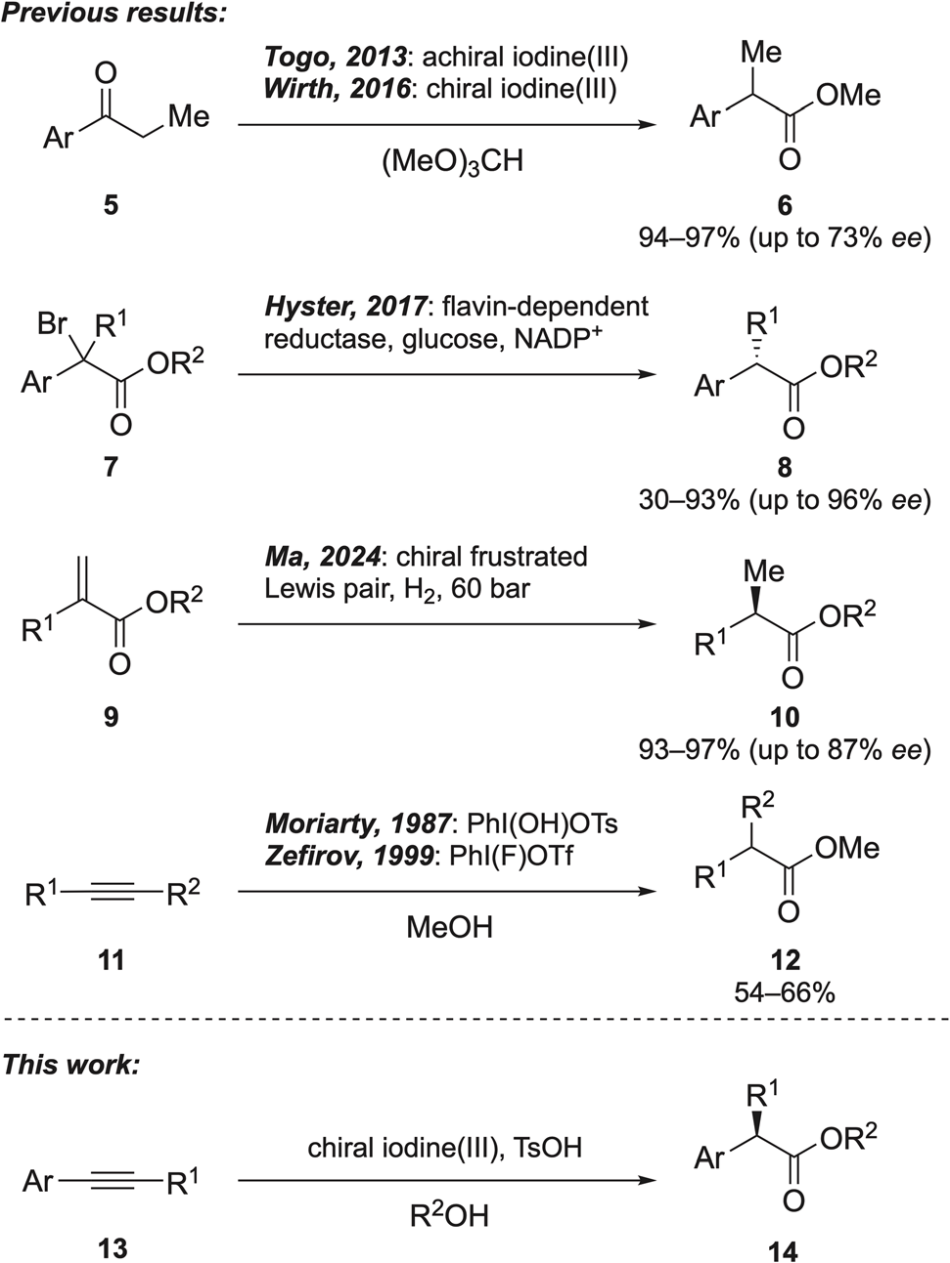
Metal-free strategies for the synthesis of α-arylalkanoic esters.

Togo and coworkers developed an oxidative 1,2-aryl migration of aryl ketones 5 to produce α-arylalkanoic esters 6 using hypervalent iodine reagents,^15^ and our group subsequently extended this approach to enantioselective synthesis using chiral iodine(iii) reagents, affording products 6 with up to 73% *ee* (Fig. 2a).^15^ Hyster and coworkers reported a new methodology for preparing chiral α-arylalkanoic esters 8 through debromination of racemic α-bromoesters 7 using flavoenzymes and glucose in the presence of NADP^+^ (Fig. 2b).^30^ More recently, Ma and coworkers developed a metal-free hydrogenation of α,β-unsaturated esters 9 to synthesise chiral α-arylalkanoic esters 10 using chiral frustrated Lewis pairs under a hydrogen atmosphere (Fig. 2c).^31^ To date, only two protocols for the synthesis of α-arylalkanoic esters from alkynes with iodine(iii) reagents have been reported. In 1987, Moriarty and coworkers used Koser's reagent [PhI(OH)OTs] for the oxidative rearrangement of alkynes 11 to esters 12 in methanol at reflux for extensive times (Fig. 2d).^32^ In 1999, Zefirov *et al.* used a different hypervalent iodine reagent [PhI(F)OTf] for the same reaction.^33^ Given these reported procedures, there is a clear need for a clean and environmentally benign process to produce α-arylalkanoic esters and hypervalent iodine chemistry presents a promising solution. In this work, we establish highly selective, metal-free conditions for the oxidative rearrangement of electron-rich alkynes 13, enabling the efficient synthesis of both chiral and racemic α-arylalkanoic esters 14 by using simply prepared, recyclable hypervalent iodine reagents.

## Results and discussion

For our initial investigation, 1-methoxy-4-(prop-1-yn-1-yl)benzene 15 was selected as a model substrate owing to its electron-rich aryl group. [Bis(trifluoroacetoxy)iodo]benzene was used as a strong oxidant for the reaction; however, only minimal decomposition of the starting material 15 was observed after 20 h at room temperature (Table 1, entry 1).

**Table 1:** Optimisation of the oxidative rearrangement of alkyne 15

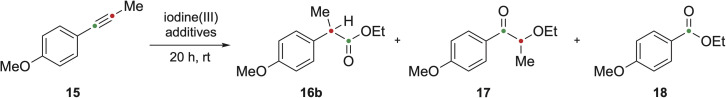							
Entry	Iodine(iii) reagent	Additives	Solvent	Conversion^a^ [%]	16b yield^b^ [%]	17 yield^b^ [%]	18 yield^b^ [%]
1	3 eq. PhI(OCOCF_3_)_2_^c^	—	EtOH	10	0	0	0
2	3 eq. PhI(OH)O*p*Ts	—	HFIP/EtOH (8 eq.)	40	Trace	0	15
3	3 eq. PhI(OH)O*p*Ts	3 eq. *p*TsOH·H_2_O	HFIP/EtOH (8 eq.)	68	13	0	16
4	3 eq. PhI(OAc)_2_	3 eq. *p*TsOH·H_2_O	HFIP/EtOH (8 eq.)	72	15	0	Trace
5	3 eq. PhI(OCOCF_3_)_2_	3 eq. *p*TsOH anh.	HFIP/EtOH (8 eq.)	75	25	0	0
6	3 eq. PhI(OCOCF_3_)_2_	3 eq. *p*TsOH anh.	HFIP/EtOH (30 eq.)	80	37	0	0
7^d^	3 eq. PhI(OCOCF_3_)_2_	3 eq. *p*TsOH anh.	EtOH	100	20	53 (47)^e^	0
8	3 eq. PhI(OCOCF_3_)_2_	3 eq. *p*TsOH anh., 3 eq. BF_3_·OEt	EtOH	91	64 (61)^e^	0	0
9	1.5 eq. PhI(OAc)_2_	1.5 eq. *p*TsOH anh.	EtOH	100	(90)^e^	0	0

a Based on re-isolated 15.

b ^1^H NMR yield using 1,3,5-trimethoxybenzene as internal standard.

c Similar results were obtained with PhI(OH)OTs and PhI(OAc)_2_ (see SI, Table S2).

d Reaction time: 40 h.

e Amount in brackets: isolated yield.

1,1,1,3,3,3-Hexafluoro-2-propanol (HFIP) is known as a highly polar, low-nucleophilicity solvent capable of stabilising charged intermediates in solution. It has been successfully employed to promote challenging transformations, including alkyne cleavage and oxidative rearrangements of alkenes.^34–36^

When a mixture of HFIP and 8 equivalents of ethanol was used in combination with Koser's reagent, only trace amounts of the rearranged product 16b were obtained, accompanied by the formation of ester 18 (Table 1, entry 2). As an oxidative cleavage of triple bonds with iodine(iii) reagents has already been reported,^37^ the generation of the product 18 was not unexpected.

To further improve the conditions for promoting the formation of the desired product, 3 equivalents of hydrated *para*-toluenesulfonic acid were added to the reaction mixture, yielding 13% of product 16b (Table 1, entry 3). When ethanol was used as a solvent with anhydrous *para*-toluenesulfonic acid, the conversion reached 100%, however, with the formation of 17 as the main product (Table 1, entry 7). The synthesis of 1,2-diketones and α-hydroxyketones from alkynes with iodine(iii) reagents has been reported,^38,39^ which explains its presence in the mixture. Application of boron trifluoride etherate had a distinct effect in generating compound 16b (Table 1, entry 8), nevertheless after further optimisation (see supporting information), the use of 1.5 equivalents of (diacetoxyiodo)benzene and anhydrous *para*-toluenesulfonic acid were found to provide 16b in yields up to 90% (Table 1, entry 9). Further optimisation details are in the SI (Tables S1–S4).

With these optimised conditions for the oxidative rearrangement, a series of chiral hypervalent iodine reagents, illustrated in Table 2, were synthesised and screened to access chiral α-arylalkanoic esters. The enantioselective oxidative rearrangement of alkynes was achieved under similar reaction conditions, simply by replacing (diacetoxyiodo)benzene with chiral iodine(iii) reagents 19 (Table 2).

**Table 2:** Screening of chiral hypervalent iodine reagents for the enantioselective synthesis of 16b

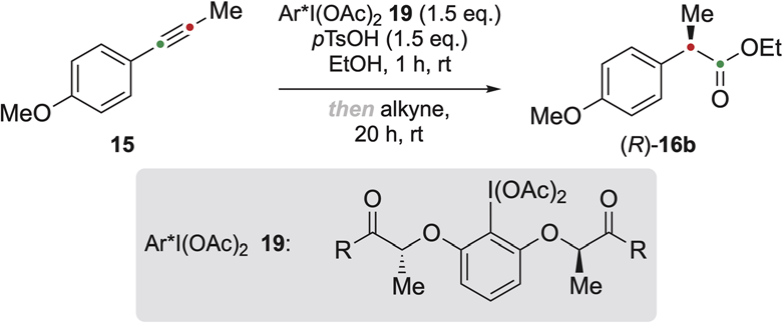				
Entry	Iodine(iii) reagent	R	(*R*)-16b yield^a^ [%]	(*R*)-16b*ee*^b^ [%]
1	19a	OMe	90	30
2	19b	OEt	89	30
3	19c	O*i*Pr	88	32
4	19d	*O*-*l*-menthyl	85	21
5	19e	NH*p*Ts	82	40
6	19f	NHC_6_F_5_	81	8
7	19g	NH[3,5-(CF_3_)_2_–C_6_H_3_]	88	0
8	19h	NH[2,6-(*i*Pr)_2_-C_6_H_3_]	82	76
9	19i	NH[(*R*)-CH(Me)Ph]	80	79
10	19j	NH[(*S*)–CH(Me)Ph]	83	85

a Isolated yields.

b Determined by HPLC.

**Fig. 3:**
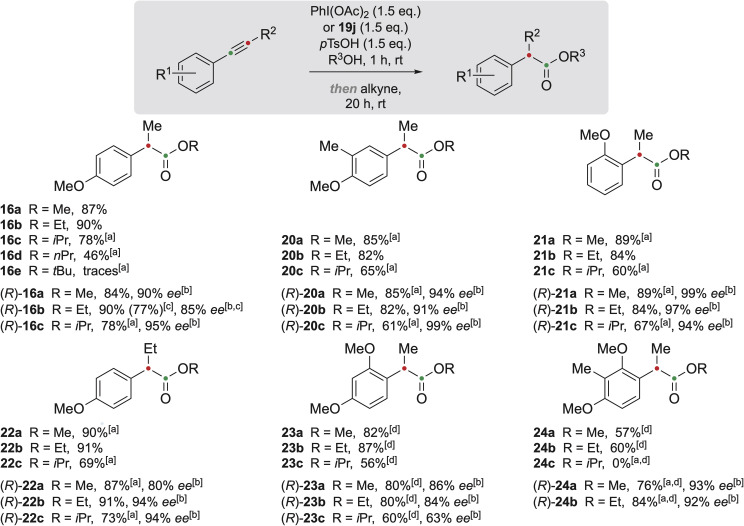
Scope of the alkyne rearrangement. ^*a*^ BF_3_·OEt_2_ (1.5 eq.) was added after the addition of the alkyne. ^*b*^ Determined by HPLC. ^*c*^ Scale-up: 19j (10.26 mmol, 7.23 g), anh. *p*TsOH (10.26 mmol, 1.77 g) in EtOH (20 mL) for 1 h, then 15 (6.84 mmol, 1.00 g) at 25 °C, 20 h. ^*d*^ The reaction was carried out at 45 °C.

Chiral hypervalent iodine reagents with alkyl lactates such as methyl (19a), ethyl (19b), and isopropyl (19c) produced (*R*)-16b in low enantioselectivities (∼30% *ee*) (Table 2, entries 1–3). Replacing alkyl esters with sterically more demanding chiral alkyl esters, such as *l*-menthyl (19d), decreased the enantiomeric excess to 21%. Iodine reagents with amide moieties were investigated because of the potential interactions between the NH hydrogen atom and the oxygen of the acetate ligands on iodine, which could enhance the chiral environment around the iodine centre, leading to higher enantiomeric excess of the desired product.^40^ Indeed, presence of the tosyl amide moiety (19e) increased the enantiomeric excess to 40% (Table 2, entry 5), and arylamides with electron-withdrawing groups such as 19f and 19g furnished (*R*)-16b in almost racemic form (Table 2, entries 6 and 7). In contrast, amides bearing aryl or alkyl substituents (19h–19j), increased the enantiomeric excess of (*R*)-16b significantly to 76%, 79% and 85%, respectively (Table 2, entries 8–10). These results clearly indicate that amide substituents play a significant role in the observed enantioselectivities of this transformation. Reagent 19j showed the best results, forming (*R*)-16b in 83% yield and with 85% *ee* (Table 2, entry 10), and thus was selected and applied to the stereoselective synthesis of the products shown in Fig. 3.

To investigate the applicability of this reaction in the formation of esters of different alcohols, methanol (16a), ethanol (16b), *n*-propanol and *iso*-propanol (16c, 16d) were used as solvents. While the yields for the methyl and ethyl esters were rather satisfying, 87% and 90% respectively, the yields for the *n*-propyl and *iso*-propyl esters were noticeably lower, 78% and 46% respectively, even after application of boron trifluoride etherate. The addition of this Lewis acid was proven to be beneficial for the formation of methyl and *iso*-propyl esters 20, 21, 22 and 24 (see Fig. 3 and SI for details).

Neither the introduction of a methyl group in the *meta*-position relative to the alkyne nor the relocation of the methoxy substituent from *para* to *ortho* significantly influenced the reaction efficiency; in both cases, products 20 and 21 were obtained in excellent yields of up to 90%. 2,4-Dimethoxy-substituted alkynes subjected to the reaction conditions at 25 °C remained unreactive and could be recovered without any noticeable decomposition, which necessitated a temperature increase to 45 °C. The temperature increase led to the formation of products 23 and 24 in 56–87% yield except for the *iso*-propyl ester 24, which was not obtained. An exchange of the methoxy-substituent in 15 to a dimethylamino moiety did not lead to a successful reaction and only 4-dimethylamino benzoic esters were formed. Several other alkynes (see SI, page S19) were also unreactive under the reaction conditions.

The yields of the chiral α-arylalkanoic esters were similar to those of the racemic ones. Alkynes bearing electron-donating groups were successfully oxidised, producing the rearranged products in high enantiomeric excesses of 80–99% (see Fig. 3, compounds (*R*)-16, 20–24), except for product (*R*)-23c which was obtained in a lower enantiomeric excess of 63%. The reaction conditions seem to be very selective to non-terminal alkynes bearing electron-donating substituents in *ortho*- or *para*-positions. When arylalkynes with electron-withdrawing groups were subjected to the reaction conditions, they were completely recovered.

With a wide scope of esters produced, further investigation was conducted to study the applicability of this method for performing this oxidative rearrangement on a larger scale. The reaction was scaled up using alkyne 15 on a gram scale. The reaction with 19j performed smoothly, delivering the desired chiral product (*R*)-16b in 77% yield (1.09 g) with identical enantioselectivity as on the small scale (85% *ee*) (Fig. 3, compound (*R*)-16b, values in brackets).

We also demonstrated that the reduced chiral iodoarene can be easily recovered in almost quantitative yield and re-oxidised to the hypervalent iodine reagent 19j for reuse. The recycled reagent showed identical results (on a small-scale reaction) without any loss of activity and selectivity, forming product (*R*)-16b in 90% yield and with 85% *ee*.

The development of a process using only catalytic amounts of an iodine(i) reagent and a stoichiometric oxidant was unsuccessful, as some oxidants did not provide sufficient reactivity (Oxone®, sodium perborate) while others directly reacted with the alkyne (Selectfluor®, *m*CPBA).

**Fig. 4:**
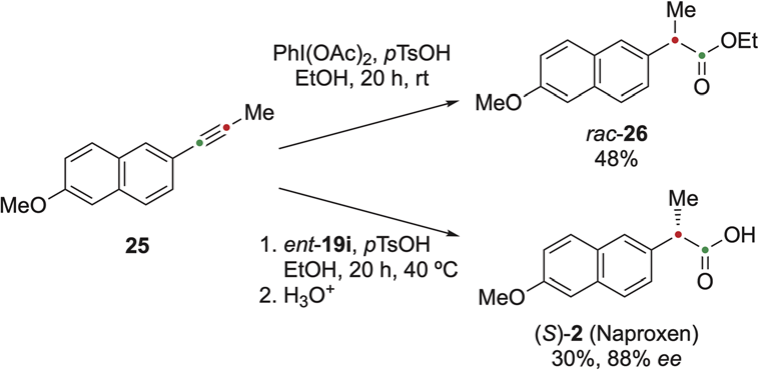
Stereoselective synthesis of Naproxen (*S*)-2. Compound *ent*-19i [(2,6-bis(((*S*)-1-oxo-1-(((*S*)-1-phenylethyl)amino)propan-2-yl)oxy)phenyl)-l^3^-iodanediyl diacetate] is the enantiomer of 19i.

In addition, the methodology was used to prepare naproxen 2 (Fig. 4). Naproxen 2 is a well-known anti-inflammatory drug, but can also be applied as a chiral discriminating reagent in NMR spectroscopy.^41^ The alkyne 25 was synthesised using the Corey–Fuchs methodology from the corresponding aldehyde and converted with (diacetoxyiodo)benzene in ethanol to naproxen ethyl ester *rac*-26 in 48% yield. The use of *ent*-19i provided (*S*)-26 in 88% *ee*, which was hydrolysed to the biologically active compound (*S*)-2 in 30% overall yield.

**Fig. 5:**
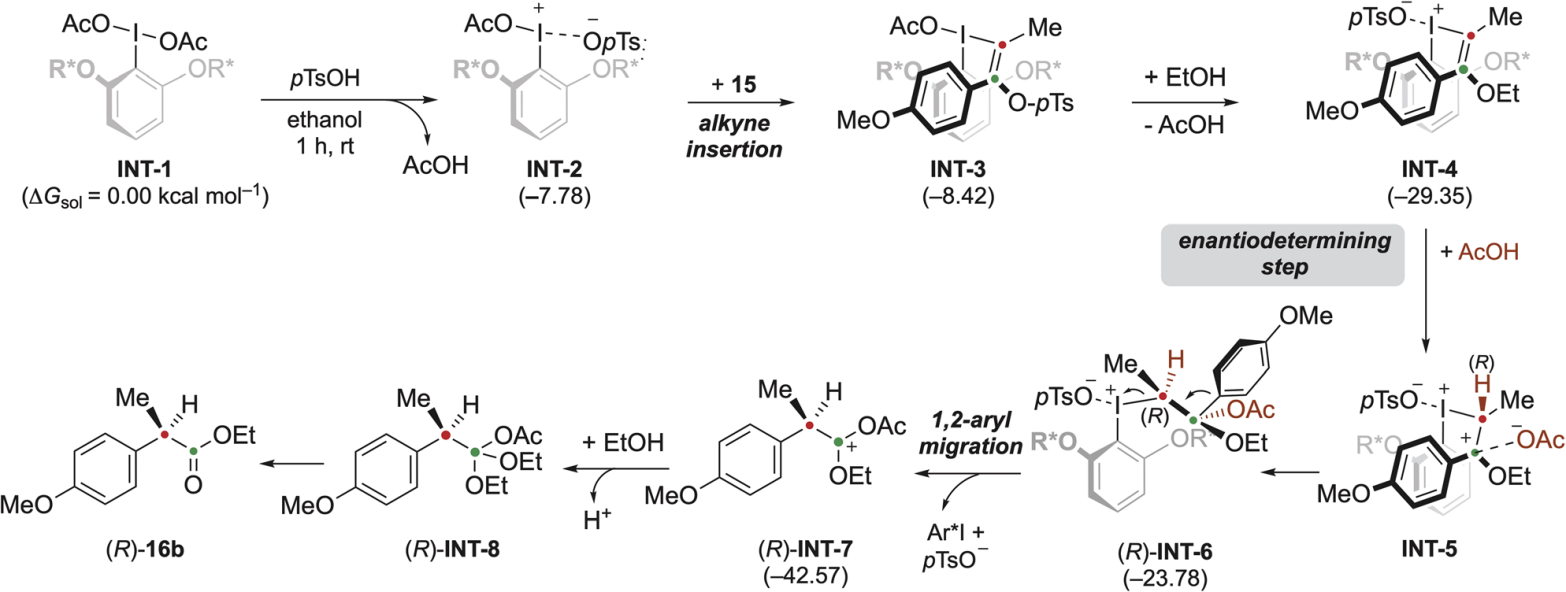
Proposed reaction mechanism. Gibbs free energies in solution are given (in kcal mol^−1^) in parentheses underneath the structure numbers.

To gain deeper insight into the origin of enantioselectivity, density functional theory (DFT) calculations were performed at the B3LYP-D4/def2-TZVP//B3LYP-D4/def2-SVP/CPCM(ethanol) level of theory. The oxidative rearrangement of substrate 15 to (*R*)-16b was chosen as a model substrate for mechanistic investigation. In agreement with literature precedence,^32,33^ the proposed mechanism is outlined in Fig. 5.

The reaction sequence begins with *para*-toluene sulfonic acid (*p*TsOH) reacting with the chiral (diacetoxyiodo)arene (INT-1), releasing acetic acid through ligand exchange and generating the active iodine(iii) species INT-2. Insertion of alkyne 15 into the I–OTs bond affords INT-3. Subsequent displacement of the tosylate by ethanol and further ligand exchange at iodine(iii) produces the more stable vinyl iodonium intermediate INT-4.

The enantiodetermining step is proposed to be the protonation of INT-4 at the α-carbon by the previously released acetic acid, thereby establishing the stereogenic centre and forming the carbocationic intermediate INT-5. Nucleophilic attack by acetate at the β-carbon atom of INT-5 then produces the unstable alkyl iodonium species INT-6. This intermediate undergoes rapid 1,2-aryl migration through an S_N_2 displacement of the iodoarene and elimination of the tosylate, providing the principal driving force for the transformation. The resulting species INT-7 reacts with ethanol to yield *ortho*ester INT-8, which hydrolyses under the reaction conditions to give (*R*)-16b.

This reaction sequence highlights the electronic demand of this reaction: resonance stabilisation by the methoxy group in INT-4 lowers the activation barrier for enantioselective protonation in electron-rich arylalkynes. Consequently, analogous substrates with –CF_3_ substituents face prohibitively high barriers to protonation by acetic acid, in agreement with experimental results (see SI, Fig. S4).

**Fig. 6:**
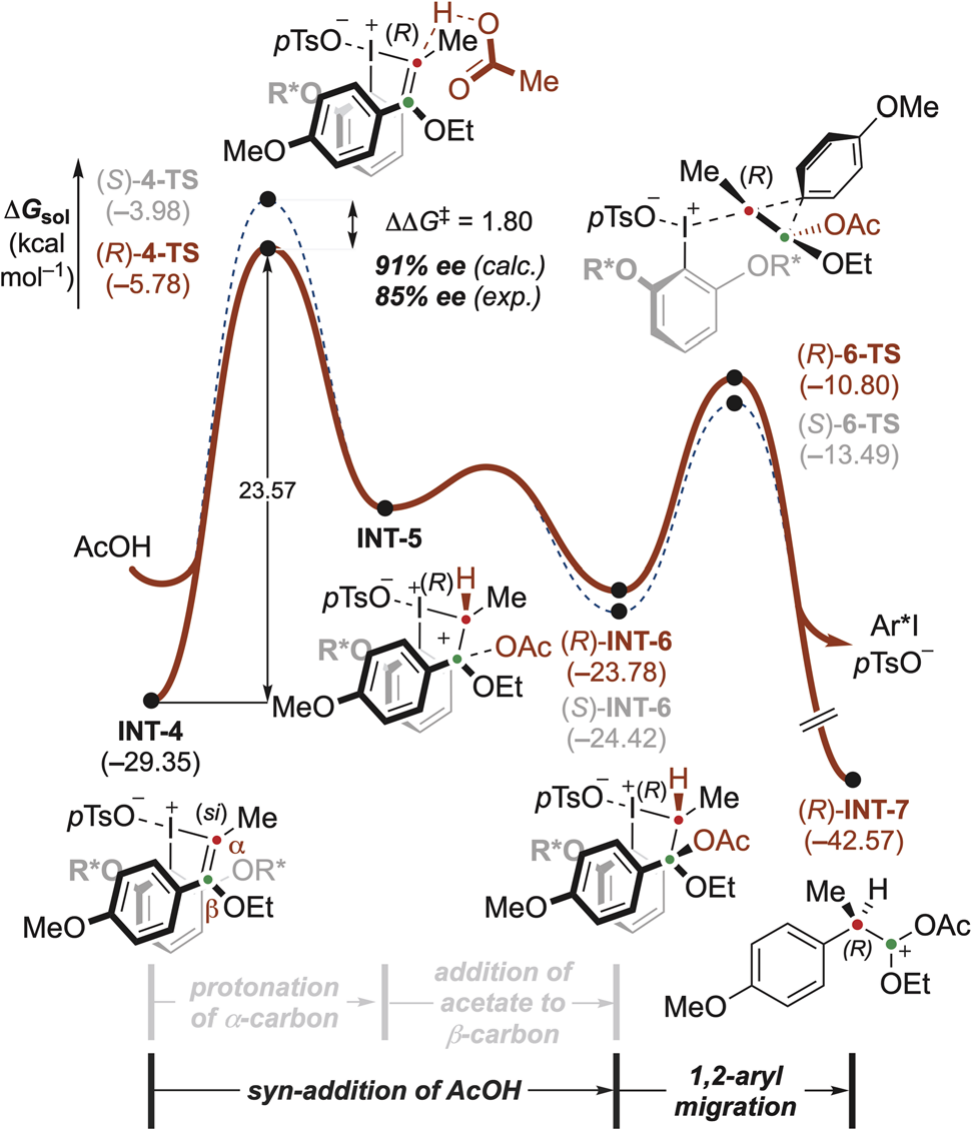
DFT calculated energy profiles for competing protonation of INT-4 by acetic acid.

To rationalise the origin of enantioselectivity, we investigated the enantiodetermining protonation step in detail and the subsequent 1,2-aryl migration. The computed energy profiles for the competing pathways leading to the (*R*)- and (*S*)-enantiomers are presented in Fig. 6. The stepwise addition of acetic acid to INT-4 constitutes the enantiodetermining step of the reaction. Conformational analysis revealed that the ground-state conformer of INT-4, in which the *si*-face is accessible for protonation is favoured by 1.1 kcal mol^−1^ over the conformer exposing the re-side (see SI, Fig. S1 and S2). Intramolecular hydrogen-bonding interactions rigidify the chiral backbone of INT-4. Exposure of the re-side necessitates a rearrangement of the chiral backbone while keeping hydrogen-bonding interactions intact, resulting in an overall unfavoured geometry. This conformational bias is reflected in the computed barrier heights for protonation: the transition state leading to the (*R*)-enantiomer, (*R*)-4-TS, is 1.8 kcal mol^−1^ more favourable than (*S*)-4-TS. The corresponding difference in barriers predicts an *ee* of 91%, in excellent agreement with the experimentally observed value of 85% (see Fig. 6). The subsequent 1,2-aryl migration and elimination of iodine(iii) fragment occur rapidly and irreversibly, confirming that protonation of INT-4 is the enantiodetermining event in this transformation.

## Conclusions

This work highlights a significant breakthrough in the enantioselective oxidative rearrangement of alkynes, enabling a highly efficient synthesis of chiral α-arylalkanoic esters under straightforward, green, and metal-free conditions. The process employs iodine(iii) reagents and *para*-toluenesulfonic acid, affording high yields across a broad range of alcohols within 20 hours. The method exhibits excellent selectivity for electron-rich, non-terminal alkynes. Novel sterically hindered alkynes were also synthesised, expanding the scope of accessible α-arylalkanoic esters. Screening of chiral C_2_-hypervalent iodine reagents identified an amide side chain as being most efficient, achieving up to 91% yield and up to 99% enantiomeric excess. The reaction was successfully scaled for practical applications. The synthesis of the drug naproxen was demonstrated alongside the recyclability of the chiral iodoarene adding to its environmental appeal.

## Experimental

### General procedure for the oxidative rearrangement of alkynes

In a 10 mL dried finger vial or round bottom flask and under nitrogen atmosphere, (diacetoxyiodo)arene (1.5 mmol, 1.5 eq.) and anhydrous *p*TsOH (1.5 mmol, 1.5 eq.) were dissolved in 1 mL of alcohol and stirred for one hour at room temperature followed by the addition of alkyne substrate (1 mmol, 1 eq.). The reaction mixture was stirred at room temperature for 20 h. The solvent was removed under reduced pressure. The residue was dissolved again with ethyl acetate and washed with sat. aq. NaHCO_3_ (10 mL) solution and sat. aq. Na_2_S_2_O_3_ solution (10 mL) and extracted with ethyl acetate (3 × 20 mL). The combined organic layers were dried over MgSO_4_ (15 g), filtered, and concentrated under reduced pressure. The crude product mixture was purified by flash chromatography on silica gel (petroleum ether:ethyl acetate: 97 : 3).

## Author contributions

T. W. conceptualised the work. R. A. developed the methodology. R. A., H. G., M. F., J. L. M, D. and D. B. performed all the experiments and analysed the data. J. W., A. G., R. B. and M.-H. B. performed and analysed the calculations. R. A., J. W., M.-H. B., R. L. M. and T. W. prepared the manuscript.

## Conflicts of interest

There are no conflicts to declare.

## Supplementary Material

SC-OLF-D5SC07882B-s001

## Data Availability

Supplementary information (SI): experimental details, characterisation data, additional discussions, DFT optimised geometries, energies and frequencies. See DOI: https://doi.org/10.1039/d5sc07882b.
