# Supplementary material for: Oxidative rearrangement of alkynes to chiral α-arylalkanoic esters
Source: Chem Sci. 2025 Nov 19;17(2):1318–24. doi: 10.1039/d5sc07882b (PMC12648175; doi:10.1039/d5sc07882b)
Supplement: SC-017-D5SC07882B-s001 [file SC-017-D5SC07882B-s001.pdf]

Supplementary Information (SI) for Chemical Science.
This journal is © The Royal Society of Chemistry 2025

Supporting Information

Oxidative Rearrangement of Alkynes to chiral α-Arylalkanoic Esters

Table of contents:

General information S2
General procedures S2
Synthesis of C_2–symmetric chiral hypervalent iodine reagents S5
Synthesis of alkynes S15
Reaction Optimisation Data S22
Reaction products S25
Computational Details S41
Energies of Computed Structures S46
Frequencies of Optimised Structures S47
Cartesian Coordinates of Optimised Structures S58
NMR spectroscopic data S106
References S164

General information

Reagents were purchased from Apollo Scientific, Acros Organics, Alfa Aesar, Fisher Scientific, FluoroChem, Merck, Sigma Aldrich and TCI were used as received without purification.

Dry solvents (diethyl ether, tetrahydrofuran, toluene, acetonitrile) were collected from a solvent purification system (M BRAUN, MB SPS-800) under nitrogen atmosphere. Dry dichloromethane was freshly distilled from calcium hydride under nitrogen atmosphere.

Thin layer chromatography (TLC) was performed to monitor the reactions using precoated aluminium sheets of Merck silica gel 60 F254 (0.20 m), and detection of compounds was performed under UV light (254 nm) or dipping into a solution of KMnO4 (1.5 g in 200 mL H_2O, 5 g $NaHCO_3$).

Flash column chromatography was performed using Merck silica gel 60 (40-63 μm) to purify products applying air pressure of about 0.2 bar or on a Biotage Isolera Four using Biotage cartridges SNAP Ultra 10 g, SNAP Ultra 25 g, and SNAP Ultra 50 g. The eluting solvents used for the purification are indicated in the text.

NMR spectroscopy: ^{1}H NMR spectra were obtained in $CDCl_3$ at 300 MHz, 400 MHz or 500 MHz. Chemical shifts are reported in ppm and referenced to the $CDCl_3$ singlet at 7.26 ppm. ^{13}C NMR spectra (all proton decoupled {^{1}H}) were obtained in $CDCl_3$ at 75 MHz, 100 MHz or 126 MHz and referenced to the centre of the $CDCl_3$ triplet at 77.16 ppm. The abbreviations s, d, t, q, quint, sext, sept, dd, ddd, dt and m stand for the resonance multiplicities singlet, doublet, triplet, quartet, quintet, septet, doublet of doublets, doublet of doublet of doublets, doublet of triplets and multiplet, respectively.

High-performance liquid chromatography (HPLC) analysis was conducted using Shimadzu Prominence HPLC System equipped with LC-10 AD coupled diode array-detector SPD-MA-10A-VP and chiral column. The solvents used were HPLC grade of *n*-hexane and 2-propanol. The chiral columns that used for the separation of enantiomers were YMC Chiral Amylose C S-5μm (0.46 cm Ø x 25 cm), Daicel Chiralcel ® OD-H (0.46 cm Ø x 25 cm), and CHIRALPAK ® IC (0.46 cm Ø x 25 cm).

Data are reported as follows: column type, eluent, wavelength, flow rate, retention time (t_R).

High resolution mass spectra (HRMS) were obtained from Cardiff University on a Waters GC-TOF spectrometer. Ions were generated using electron ionisation (EI), chemical ionisation (CI) atmospheric pressure chemical ionisation (APCI), or atmospheric-solid-analysis-probe (ASAP) techniques. All signals are reported with a mass-to-charge (m/z) ratio unit. Software: MassLynx Mass Spectrometry Software (Waters).

Note: Hypervalent iodine reagents could not be detected as the ligands fragmented before detection.

General procedures

All Reactions involving air and moisture sensitive reagents were carried out in dried glassware under a dry nitrogen atmosphere using Schlenk technique or using a balloon. All reactions were stirred using magnetic stirring. If needed, heating was performed using a hotplate controlled by a temperature probe. Lower temperatures reactions were achieved by using ice/water bath (0 °C), dry ice/acetone bath (–78 °C) or using a chiller (0 to –20 °C). Büchi rotavapors were used for solvent evaporations, and a high vacuum apparatus was used to further dry the products.

General procedure (GP1) for the Mitsunobu reaction:

In a dry round bottom flask, the protected 2-iodobenzene-1,3-diol (1 eq.), lactate derivative (2.5 eq.) and PPh_3 (2.5 eq.) were dissolved in dry tetrahydrofuran (THF) under nitrogen. The reaction mixture was cooled to 0 °C and DIAD (2.5 eq.) was added dropwise. After 1 h at 0 °C, the mixture was warmed up to room temperature for 16 h. The solvent was removed under reduced pressure and Et_2O was added. Triphenylphosphine oxide, which precipitated, was removed by filtration and the filtrate was concentrated under vacuum. The crude mixture was purified by flash column chromatography with (petroleum ether/ethyl acetate: 8:2).[1]

General Procedure (GP2) for the synthesis of lactamides:

Method A: In a dry round bottom flask, (2*R*,2'*R*)-2,2'-((2-iodo-1,3-phenylene)bis(oxy))dipropionic acid (1 eq.) was suspended in dry dichloromethane under nitrogen atmosphere. After the addition of a catalytic amount of DMF and oxalyl chloride (3.5 eq.) at 0 °C, the reaction mixture was warmed up to room temperature, stirred for 3 h. Then, the reaction mixture was concentrated under reduced pressure. Afterwards, the crude product was redissolved again in dry dichloromethane under nitrogen atmosphere and the solution was cooled to 0 °C. Subsequently, the desired primary amine (4 eq.) and dry pyridine (4 eq.) were added, the reaction solution was warmed to room temperature and stirred for 16 h. The reaction was then quenched with 3 M aqueous HCl (50 mL) and the resulting mixture extracted with dichloromethane (3 × 30 mL). The combined organic layers were dried over anhydrous $MgSO_4$, concentrated under vacuum. The crude mixture was purified by flash column chromatography (ethyl acetate:petroleum ether 30:70).

Method B: In dry round bottom flask, (2*R*,2'*R*)-2,2'-((2-iodo-1,3-phenylene)bis(oxy))dipropionic acid (1 eq.) was suspended in dry dichloromethane under nitrogen atmosphere. After the addition of a catalytic amount of DMF and oxalyl chloride (3.5 eq.) at 0 °C, the reaction mixture was warmed up to room temperature, stirred for 3 h. Then, the reaction mixture was concentrated under reduced pressure. Afterwards, the crude product was redissolved again in dry dichloromethane under nitrogen atmosphere and the solution was cooled to 0 °C. Subsequently, the desired primary amine (4 eq.) and triethylamine (4 eq.) were added, the reaction solution was warmed to room temperature and stirred for 16 h. The reaction was then quenched with 3 M aqueous HCl (50 mL) and the resulting mixture extracted with dichloromethane (3 × 30 mL). The combined organic layers were dried over anhydrous $MgSO_4$ and concentrated under vacuum. The crude mixture was purified by flash column chromatography (ethyl acetate:petroleum ether 50:50).

General Procedure (GP3) for the oxidation of iodoarenes to iodoarene diacetates:

The iodoarene (1 eq.) was dissolved in CH_3CN /glacial acetic acid (3/1) under nitrogen atmosphere. Selectfluor® (10 eq.) was added one portion and the resulting suspension was allowed to stir at room temperature for 7 h. After completion of the reaction, the solvents were removed under vacuum and the product was dissolved in $CHCl_3/H_2O$ (1/1). The organic phase was separated, and the aqueous phase was extracted with CH_2Cl_2 (3 × 30 mL). The combined organic layers were dried over anhydrous $MgSO_4$ and concentrated under a vacuum. The residue was washed with Et_2O/petroleum ether (3:1) to afford the pure product.[2]

General procedure (GP4) for synthesis of alkyne substrates:

In three-neck round bottom flask, derivative of ethynylbenzene (1 eq.) was deprotonated in THF by addition of *n*-BuLi solution (2.5 M, 1.5 eq.) slowly at –78 ºC under nitrogen atmosphere. After the slow addition, the mixture was allowed to warm up to ambient temperature and stirred for 30 minutes. Then, alkyl iodide (3 eq.) was added to the reaction mixture at –78 ºC. The reaction was stirred overnight 16 h at ambient temperature. The reaction was quenched with saturated NH_4Cl solution (100 mL), extracted with ethyl acetate (3 × 100 mL) and the combined organic dried over anhydrous $MgSO_4$, filtered and concentrated under reduced

pressure. The crude mixture was purified by flash column chromatography with (petroleum ether:ethyl acetate 99:1) to afford the pure product.[3]

General procedure (GP5) for oxidative rearrangement of alkynes:

Method A: In a 10 mL dried finger vial or round bottom flask and under nitrogen atmosphere, (diacetoxyiodo)arene (1.5 mmol, 1.5 eq.) and anhydrous *p*TsOH (1.5 mmol, 1.5 eq.) were dissolved in 1 mL of alcohol and stirred for one hour at room temperature followed by the addition of alkyne substrate (1 mmol, 1 eq.). The reaction mixture was stirred at room temperature for 20 h. The solvent was removed under reduced pressure. The residue was dissolved again with ethyl acetate and washed with sat. aq. $NaHCO_3$ (10 mL) solution and sat. aq. $Na_2S_2O_3$ solution (10 mL) and extracted with ethyl acetate (3 × 20 mL). The combined organic layers were dried over $MgSO_4$ (15 g), filtered, and concentrated under reduced pressure. The crude product mixture was purified by flash chromatography on silica gel (petroleum ether:ethyl acetate: 97:3).

Method B: In a 10 mL dried finger vial or round bottom flask and under nitrogen atmosphere, (diacetoxyiodo)arene (1.5 mmol, 1.5 eq.) and anhydrous *p*TsOH (1.5 mmol, 1.5 eq.) were dissolved in 1 mL of alcohol and stirred for one hour at room temperature followed by the addition of alkyne substrate (1 mmol, 1 eq.) and boron trifluoride diethyl etherate $BF_3 \cdot Et_2O$ (1.5 mmol, 1.5 eq.) at 0 ºC. The reaction mixture was stirred at room temperature for 20 h. Then, the solvent was removed under reduced pressure. The residue was dissolved again with ethyl acetate and washed with sat. aq. $NaHCO_3$ (10 mL) solution and sat. aq. $Na_2S_2O_3$ solution (10 mL) and extracted with ethyl acetate (3 × 20 mL). The combined organic layers were dried over $MgSO_4$ (15 g), filtered, and concentrated under reduced pressure. The crude product mixture was purified by flash chromatography on silica gel (petroleum ether::ethyl acetate: 97:3).

Method C: in 10 mL dried finger vial or round bottom flask and under nitrogen atmosphere, (diacetoxyiodo)arene (1.5 mmol, 1.5 eq.) and anhydrous *p*TsOH (1.5 mmol, 1.5 eq.) were dissolved in 1 mL of alcohol and stirred for one hour at room temperature followed by the addition of alkyne substrate (1 mmol, 1 eq.). The reaction mixture was stirred at 40 ºC for 20 h. Then, the solvent was removed under reduced pressure. The residue was dissolved again with ethyl acetate and washed with sat. aq. $NaHCO_3$ (10 mL) solution and sat. aq. $Na_2S_2O_3$ solution (10 mL) and extracted with ethyl acetate (3 × 20 mL). The combined organic layers were dried over $MgSO_4$ (15 g), filtered, and concentrated under reduced pressure. The crude product mixture was purified by flash chromatography on silica gel (petroleum ether:ethyl acetate: 97:3).

The finger vial used in above reactions were dried in the oven at 150 ºC before use.

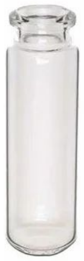

Finger vial, diameter 20 mm, height: 50 mm.

Synthesis of C_2–symmetric chiral hypervalent iodine reagents

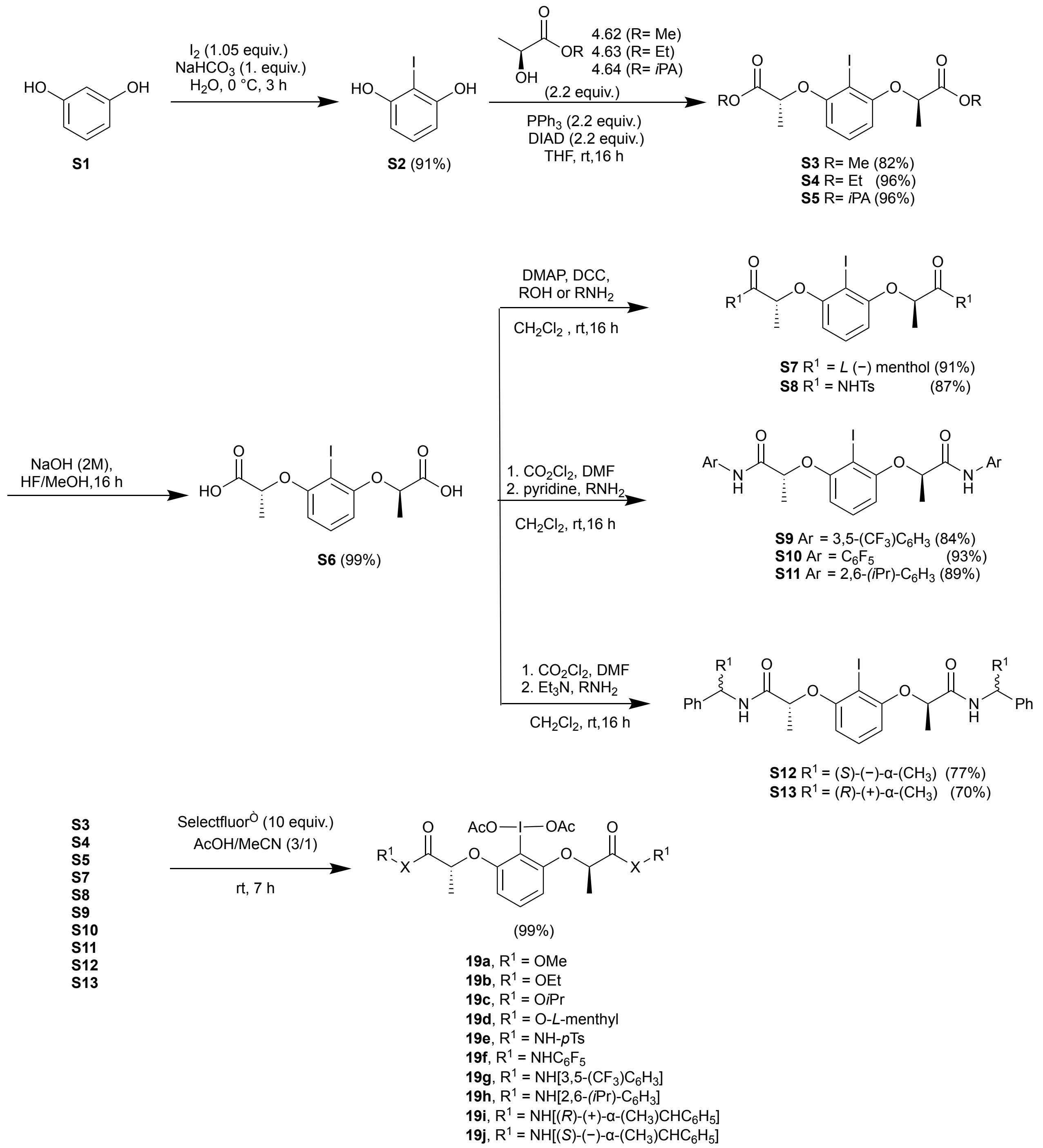

Figure S1: Synthesis of C_2-symmetric chiral hypervalent iodine reagents **19**.

2-Iodobenzene-1,3-diol (S2)

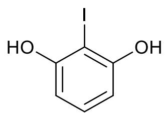

Following a reported procedure,[1] resorcinol (10.0 g, 90.8 mmol) was dissolved in 100 mL H_2O. The reaction mixture was cooling to 0 °C, iodine (24.2 g, 95.36 mmol, 1.05 eq.) was added followed by slow addition of $NaHCO_3$ (8.39 g, 99.9 mmol, 1.1 eq.). The resulting reaction mixture was stirred for 1 hour at 0 °C and then allowed to warm to room temperature over a period of 2-3 hour. The reaction was then quenched by addition of saturated aqueous $Na_2S_2O_3$ solution. After that the reaction was extracted with ethyl acetate, the combined organic phases were washed with brine, dried over anhydrous $MgSO_4$ and concentrated under reduced pressure. The crude product was purified by column chromatography with (petroleum ether/ethyl acetate: 1:9) followed by recrystallization from $CHCl_3$ afforded the title product as colourless solid (19 g, 90% yield).

^{1}H NMR (300 MHz, $CDCl_3$) δ = 7.12 (t, *J* = 8.1 Hz, 1H), 6.56 (d, *J* = 8.1 Hz, 2H), 5.26 (s, 2H) ppm.

^{13}C NMR (75 MHz, $CDCl_3$) δ = 155.8, 130.5, 107.4, 77.7 ppm.

Data agree with the literature.[1]

Dimethyl 2,2'-((2-iodo-1,3-phenylene)bis(oxy))(2*R*,2'*R*)-dipropionate (S3)

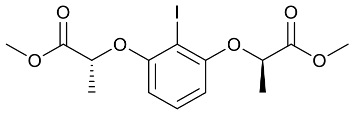

Following GP1: 2-Iodoresorcinol (5.0 g, 21.18 mmol, 1 eq.), PPh_3 (13.89 g, 52.96 mmol, 2.5 eq.), (*S*)-methyl lactate (5.50 g, 5.06 mL, 52.96 mmol, 2.5 eq.), and DIAD (10.70 g, 10.40 mL, 52.96 mmol, 2.5 eq.) in THF (100 mL). The product was collected as colourless solid (7.0 g, 17.15 mmol, 82%).

^{1}H NMR (500 MHz, $CDCl_3$) δ = 7.12 (t, *J* = 8.2 Hz, 1H), 6.34 (d, *J* = 8.2 Hz, 2H), 4.74 (q, *J* = 6.9 Hz, 2H), 3.72 (s, 6H), 1.68 (d, *J* = 6.9 Hz, 6H) ppm.

^{13}C NMR (126 MHz, $CDCl_3$) δ = 172.3, 158.4, 129.7, 107.0, 80.8, 74.3, 52.5, 18.7 ppm.

Data agree with the literature.[1]

Diethyl 2,2'-((2-iodo-1,3-phenylene)bis(oxy))(2R,2'R)-dipropionate (S4)

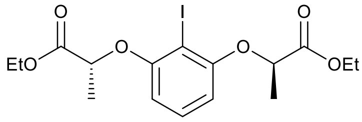

Following GP1: 2-Iodoresorcinol (2.76 g, 11.69 mmol, 1 eq.), PPh_3 (7.67 g, 29.23 mmol, 2.5 eq.), (*S*)-ethyl lactate (3.45 g, 3.31 mL, 29.23 mmol, 2.5 eq.), and DIAD (5.9 g, 5.76 mL, 29.23 mmol, 2.5 eq.) in THF (50 mL). The product was collected as colourless solid (3.9 g, 96%).

^{1}H NMR (500 MHz, $CDCl_3$) δ= 7.12 (td, *J* = 8.2, 1.4 Hz, 1H), 6.37 (dd, *J* = 8.3, 1.4 Hz, 2H), 4.74 (qd, *J* = 6.8, 1.1 Hz, 2H), 4.27 – 4.09 (m, 4H), 1.69 (dd, *J* = 6.8, 1.7 Hz, 6H), 1.24 (td, *J* = 7.1, 1.6 Hz, 6H) ppm.

^{13}C NMR (126 MHz, $CDCl_3$) δ= 171.8, 158.4, 129.6, 107.0, 80.8, 74.4, 61.4, 18.7, 14.2 ppm.

Data agree with the literature.[4]

Diisopropyl 2,2'-((2-iodo-1,3-phenylene)bis(oxy))(2*R*,2'*R*)-dipropionate (S5)

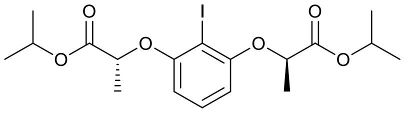

Following GP1: 2-Iodoresorcinol (0.5 g, 2.11 mmol, 1 eq.), PPh_3 (1.39 g, 5.30 mmol, 2.5 eq.), (*S*)-isopropyl lactate (0.70 g, 5.30 mmol, 2.5 eq.), and DIAD (1.07 g, 1.04 mL, 5.30 mmol, 2.5 eq.) in THF (30 mL). The title product was collected as colourless viscous liquid (2.3 g, 96%).

^{1}H NMR (500 MHz, $CDCl_3$) δ= 7.11 (t, *J* = 8.5 Hz, 1H), 6.36 (d, *J* = 8.0 Hz, 2H), 5.05 (hept, *J* = 6.0 Hz, 2H), 4.71 (q, *J* = 7.0 Hz, 2H), 1.68 (d, *J* = 6.5 Hz, 6H), 1.25 (d, *J* = 6.0 Hz, 6H), 1.16 (d, *J* = 6.0 Hz, 6H) ppm.

^{13}C NMR (126 MHz, $CDCl_3$) δ= 171.3, 158.5, 129.5, 107.1, 80.9, 74.5, 69.0, 21.8, 21.7, 18.6 ppm.

Data agree with the literature.[5]

(2*R*,2'*R*)-2,2'-((2-Iodo-1,3-phenylene)bis(oxy))dipropionic acid (S6)

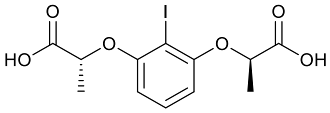

Following a reported procedure,[6] dimethyl 2,2'-((2-iodo-1,3-phenylene)bis(oxy)) (2*R*,2'*R*)-dipropionate (5 g, 12.25 mmol, 1 eq.) was dissolved in THF (30 mL) and MeOH (30 mL) under nitrogen atmosphere. After cooling the solution to 0 °C, a 2 M aqueous solution of NaOH (30 mL) was added slowly and the resulting solution was stirred at room temperature for 18 h. The reaction mixture was then acidified with 3 M aq. HCl at 0 °C and extracted with EtOAc (3 × 20 mL). The combined organic layers were washed with brine (50 mL), dried over anhydrous $MgSO_4$ and concentrated under vacuum to afford the pure product without further purification. The title product was formed as colourless solid (4.5 g, 12.24 mmol, 99 %).

^{1}H NMR (500 MHz, DMSO) δ = 13.05 (sbr, 2H), 7.21 (t, *J* = 8.3 Hz, 1H), 6.43 (d, *J* = 8.4 Hz, 2H), 4.85 (q, *J* = 6.8 Hz, 2H), 1.55 (d, *J* = 6.8 Hz, 6H) ppm.

^{13}C NMR (126 MHz, DMSO) δ = 172.6, 157.7, 129.6, 105.9, 79.5, 72.8, 18.3 ppm.

Data agree with the literature.[5]

Bis((1*S*,2*R*,5*S*)-2-isopropyl-5-methylcyclohexyl) 2,2'-((2-iodo-1,3-phenylene) bis(oxy)) (2*R*,2'*R*) dipropionate (S7)

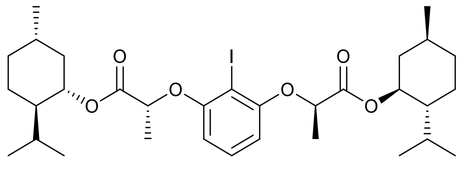

In a dry flask under nitrogen atmosphere, (2*R*,2'*R*)-2,2'-((2-iodo-1,3-phenylene)bis(oxy))dipropionic acid (2.0 g, 5.27 mmol, 1 eq.), *L*-(−)-menthol (2.06 g, 13.18 mmol, 2.5 eq.), and *N,N*-dimethyl-4-aminopyridine (DMAP) (0.45 g, 3.69 mmol, 0.7 eq.) were dissolved in 40 mL dichloromethane. Then, *N,N'*-dicyclohexylcarbodiimide (DCC) (2.72 g, 13.18 mmol, 2.5 eq.) was dissolved in 10 mL dichloromethane and added dropwise to the reaction mixture at 0 °C. The reaction was stirred at room temperature for 18 h. The reaction mixture was quenched by addition of 3M HCl aq. (50 mL) and extracted with dichloromethane (3 × 50 mL). The organic layers were collected and dried with $MgSO_4$. The crude mixture was purified by column chromatography on silica gel with (petroleum ether:ethyl acetate 8:2) to give the title product as a colourless viscous (3.1 g, 91% yield).

^{1}H NMR (300 MHz, $CDCl_3$) δ 7.11 (t, *J* = 8.3 Hz, 1H), 6.35 (d, *J* = 8.3 Hz, 2H), 4.75 (q, *J* = 6.7 Hz, 2H), 4.67 – 4.56 (m, 2H), 1.99 (d, *J* = 11.5 Hz, 2H), 1.72 – 1.29 (m, 16H), 1.06 – 0.50 (m, 24H) ppm.

^{13}C NMR (75 MHz, $CDCl_3$) δ 171.6, 158.4, 129.5, 106.3, 80.2, 77.3, 75.5, 74.4, 46.8, 40.6, 34.2, 31.4, 25.6, 23.0, 22.1, 20.9, 18.7, 15.7 ppm.

Data agree with the literature.[7]

(2*R*,2'*R*)-2,2'-((2-Iodo-1,3-phenylene)bis(oxy))bis(*N*-tosylpropanamide) (S8)

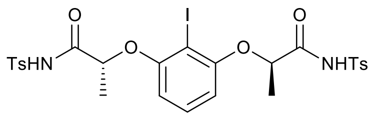

In 50 mL dry dichloromethane, (2*R*,2'*R*)-2,2'-((2-iodo-1,3-phenylene)bis(oxy))dipropionic acid (0.92 g, 2.4233 mmol, 1 eq.), *p*-toluenesulfonamine (0.91 g, 5.33 mmol, 2.2 eq.), DMAP (1.18 g, 9,69 mmol, 4 eq.), and DCC (2 g, 9.69 mmol, 4 eq.) was stirred at room temperature for 18 h. The reaction mixture was quenched by addition of 3M HCl aq. and extracted with dichloromethane (3 × 30 mL). Then, organic layers were collected and dried over $MgSO_4$. The crude mixture was purified by column chromatography on silica gel with (petroleum ether:ethyl acetate 8:2) to give the title product as a yellow viscous liquid (1.4 g, 87% yield).

^{1}H NMR (500 MHz, $CDCl_3$) δ= 9.30 (s, 2H), 7.97 (d, *J* = 8.0 Hz, 4H), 7.35 (d, *J* = 8.0 Hz, 4H), 7.19 (t, *J* = 8.3 Hz, 1H), 6.41 (d, *J* = 8.4 Hz, 2H), 4.70 (q, *J* = 6.8 Hz, 2H), 2.45 (s, 6H), 1.59 (d, *J* = 6.8 Hz, 6H) ppm.

^{13}C NMR (126 MHz, $CDCl_3$) δ= 168.8, 156.6, 145.5, 135.3, 130.8, 129.8, 128.6, 108.0, 81.4, 76.4, 21.8, 17.8 ppm.

Data agree with the literature.[7]

(2*R*,2'*R*)-2,2'-((2-Iodo-1,3-phenylene)bis(oxy))bis(*N*-(3,5-bis(trifluoromethyl)phenyl)propanamide) (S9)

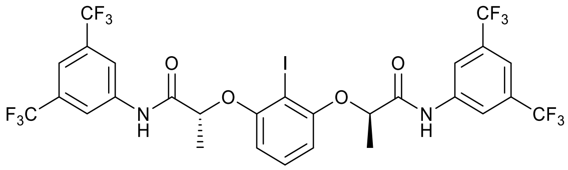

GP2, Method A: (2*R*,2'*R*)-2,2'-((2-iodo-1,3-phenylene)bis(oxy))dipropionic acid (4 g, 10.55 mmol, 1 eq.), DMF (1 mL), oxalyl chloride (4.7 g, 3.13 mL, 36.92 mmol, 3.5 eq.), 3,5-bis(trifluoromethyl)aniline (9.67 g, 6.56 mL, 42.20 mmol, 4.0 eq.), and dry pyridine (3.33 g, 3.4 mL, 42.20 mmol, 4.0 eq.) in dichloromethane

(50 mL). The reaction mixture was purified by column chromatography on silica gel with (petroleum ether:ethyl acetate 8:2) to give the title product as a colourless solid (7.1 g, 84% yield).

^{1}H NMR (300 MHz, $CDCl_3$) δ 9.03 (s, 2H), 8.16 (s, 4H), 7.67 (s, 2H), 7.38 (t, J = 8.3 Hz, 1H), 6.64 (d, J = 8.4 Hz, 2H), 4.99 (q, J = 6.7 Hz, 2H), 1.77 (d, J = 6.7 Hz, 6H) ppm.

^{13}C NMR (75 MHz, $CDCl_3$) δ 169.5, 156.7, 138.7, 132.5 (q, $J_{C\text{–}F}$ = 33 Hz), 131.2, 124.9 (q, $J_{C\text{–}F}$ = 271 Hz), 119.7 (d, $J_{C\text{–}F}$ = 3.6 Hz), 118.3 – 118.0 (m), 107.8, 81.1, 76.3, 18.3 ppm.

Data agree with the literature.[8]

(2*R*,2'*R*)-2,2'-((2-Iodo-1,3-phenylene)bis(oxy))bis(*N*-(perfluorophenyl)propanamide) (S10)

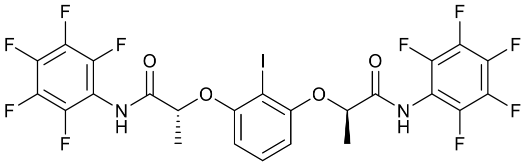

GP2, Method A: (2*R*,2'*R*)-2,2'-((2-iodo-1,3-phenylene)bis(oxy))dipropionic acid (4 g, 10.55 mmol, 1 eq.), DMF (1 mL), oxalyl chloride (4.7 g, 3.13 mL, 36.92 mmol, 3.5 eq.), 2,3,4,5,6-pentafluoroaniline (7.7 g, 4.5 mL, 42.20 mmol, 4.0 eq.), and dry pyridine (3.33 g, 3.4 mL, 42.20 mmol, 4.0 eq.) in dichloromethane (50 mL). The reaction mixture was purified by column chromatography on silica gel with (petroleum ether:ethyl acetate 7:3) to give the title product as a colourless solid (7.0 g, 93% yield).

^{1}H NMR (300 MHz, $CDCl_3$) δ 8.31 (s, 2H), 7.37 (t, J = 8.3 Hz, 1H), 6.63 (d, J = 8.4 Hz, 2H), 5.01 (q, J = 6.7 Hz, 2H), 1.77 (d, J = 6.7 Hz, 6H) ppm.

^{13}C NMR (75 MHz, $CDCl_3$) δ 169.7, 156.8, 144.5 –141.3 (m), 139.9 –138.8 (m), 138.7 –136.3 (m), 131.0, 111.2 (dt, J = 3.5, 15 Hz), 107.8, 81.0, 76.3, 18.4 ppm.

Data agree with the literature.[9]

(2*R*,2'*R*)-2,2'-((2-Iodo-1,3-phenylene)bis(oxy))bis(*N*-(2,6-diisopropylphenyl)propanamide) (S11)

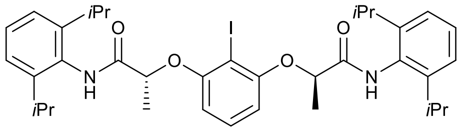

GP2, Method A: (2*R*,2'*R*)-2,2'-((2-iodo-1,3-phenylene)bis(oxy))dipropionic acid (2.4 g, 6.33 mmol, 1 eq.), DMF two drops, oxalyl chloride (2.81 g, 1.87 mL, 22.15 mmol, 3.5 eq.), 2,6-diisopropylaniline (4.49 g, 4.78 mL, 25.32 mmol, 4.0 eq.), and dry pyridine (2.0 g, 2.05 mL, 25.32 mmol, 4.0 eq.) in dichloromethane (30 mL). The reaction mixture was purified by column chromatography on silica gel with (petroleum ether:ethyl acetate 1:1) to give the title product as a colourless solid (3.3 g, 89% yield).

^{1}H NMR (500 MHz, $CDCl_3$) δ= 8.04 (s, 2H), 7.42 (t, J = 8.3 Hz, 1H), 7.35 (t, J = 7.7 Hz, 2H), 7.22 (d, J = 7.8 Hz, 4H), 6.75 (d, J = 8.4 Hz, 2H), 5.11 (q, J = 6.7 Hz, 2H), 3.03 (br, 4H), 1.84 (d, J = 6.7 Hz, 6H), 1.30 – 1.12 (m, 24H) ppm.

^{13}C NMR (126 MHz, $CDCl_3$) δ 170.5, 157.0, 146.2, 130.6, 130.0, 128.6, 123.5, 107.1, 80.5, 76.0, 28.7, 23.6, 18.6 ppm.

Data agree with the literature.[2]

(2*R*,2'*R*)-2,2'-((2-Iodo-1,3-phenylene)bis(oxy))bis(*N*-((*R*)-1-phenylethyl)propanamide) (S12):

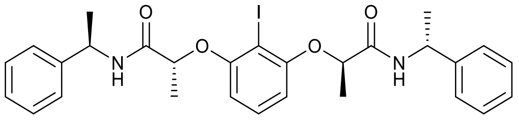

GP2, Method B: (2*R*,2'*R*)-2,2'-((2-iodo-1,3-phenylene)bis(oxy))dipropionic acid (3 g, 7.91 mmol, 1 eq.), DMF two drops, oxalyl chloride (3.50 g, 2.2 mL, 27.69 mmol, 3.5 eq.), (*R*)-(+)-*α*-methylbenzylamine (3.8 g, 4.0 mL, 31.65 mmol, 4.0 eq.), and triethylamine (3.20 g, 4.4 mL, 31.65 mmol, 4.0 eq.) in dichloromethane (35 mL). The reaction mixture was purified by column chromatography on silica gel with (petroleum ether:ethyl acetate 1:1) to give the title product as a colourless solid (3.2 g, 70% yield).

^{1}H NMR (300 MHz, $CDCl_3$) δ 7.36 – 7.21 (m, 13H), 6.48 (d, *J* = 8.4 Hz, 2H), 5.25 – 5.11 (m, 2H), 4.81 (q, *J* = 6.7 Hz, 2H), 1.70 (d, *J* = 6.7 Hz, 6H), 1.61 (d, *J* = 7.0 Hz, 6H) ppm.

^{13}C NMR (75 MHz, $CDCl_3$) δ = 170.2, 157.0, 143.0, 130.6, 128.7, 127.5, 126.2, 107.0, 80.6, 76.0, 48.8, 22.4, 18.4 ppm.

Data agree with the literature.[10]

(2*S*,2'*S*)-2,2'-((2-Iodo-1,3-phenylene)bis(oxy))bis(*N*-((*S*)-1-phenylethyl)propanamide) (*ent*-S12)

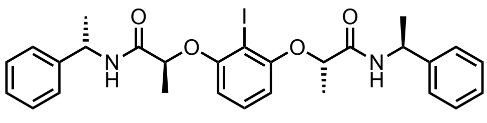

GP2, Method B: (2*R*,2'*R*)-2,2'-((2-iodo-1,3-phenylene)bis(oxy))dipropionic acid (2 g, 5.27 mmol, 1 eq.), DMF two drops, oxalyl chloride (2.30 g, 18.46 mmol, 3.5 eq.), (*S*)-(−)-*α*-methylbenzylamine (2.5 g, 2.7 mL, 21.1 mmol, 4.0 eq.), and triethylamine (2.14 g, 2.94 mL, 21.1 mmol, 4.0 eq.) in dichloromethane (30 mL). The reaction mixture was purified by column chromatography on silica gel (petroleum ether:ethyl acetate 1:1) to give the title product as a colourless solid (2.3 g, 77% yield).

$[\alpha]_D^{20}$ = +81.1 (c = 0.44, $CHCl_3$).

(2*R*,2'*R*)-2,2'-((2-Iodo-1,3-phenylene)bis(oxy))bis(*N*-((*S*)-1-phenylethyl)propanamide) (S13)

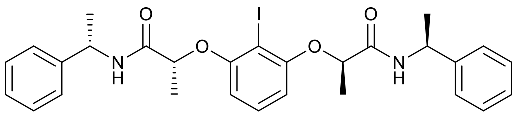

GP2, Method B: (2*R*,2'*R*)-2,2'-((2-iodo-1,3-phenylene)bis(oxy))dipropionic acid (2 g, 5.27 mmol, 1 eq.), DMF two drops, oxalyl chloride (2.30 g, 18.46 mmol, 3.5 eq.), (*S*)-(−)-*α*-methylbenzylamine (2.5 g, 2.7 mL, 21.1 mmol, 4.0 eq.), and triethylamine (2.14 g, 2.94 mL, 21.1 mmol, 4.0 eq.) in dichloromethane (30 mL). The reaction mixture was purified by column chromatography on silica gel (petroleum ether:ethyl acetate 1:1) to give the title product as a colourless solid (2.3 g, 77% yield).

^{1}H NMR (300 MHz, $CDCl_3$) δ 7.39 – 7.22 (m, 13H), 6.54 (d, *J* = 8.3 Hz, 2H), 5.23 – 5.12 (m, 2H), 4.83 (q, *J* = 6.7 Hz, 2H), 1.62 (d, *J* = 6.7 Hz, 6H), 1.49 (d, *J* = 6.9 Hz, 6H) ppm.

^{13}C NMR (75 MHz, $CDCl_3$) δ 170.1, 157.1, 143.1, 130.7, 128.8, 127.5, 126.1, 107.0, 80.7, 76.1, 48.7, 22.4, 18.3 ppm.

Data agree with the literature.[10]

Dimethyl 2,2'-((2-(diacetoxy-λ^3-iodaneyl)-1,3-phenylene)bis(oxy))(2*R*,2'*R*)-dipropionate (19a):

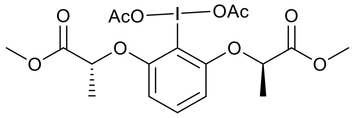

GP3: Dimethyl 2,2'-((2-iodo-1,3-phenylene)bis(oxy))(2*R*,2'*R*)-dipropionate (0.5 g, 1.22 mmol, 1 eq.), Selectfluor® (2.2 g, 6.12 mmol, 5.0 eq.), AcOH (3 mL), acetonitrile (9 mL). The title product was obtained in (0.63 g, 99%).

$[\alpha]_D^{20}$ = –75.0 (c = 0.98, $CHCl_3$).

^1H NMR (300 MHz, $CDCl_3$) δ 7.40 (t, *J* = 8.4 Hz, 1H), 6.58 (d, *J* = 8.4 Hz, 2H), 4.87 (q, *J* = 6.8 Hz, 2H), 3.76 (s, 6H), 1.98 (s, 6H), 1.68 (d, *J* = 6.8 Hz, 6H) ppm.

^{13}C NMR (75 MHz, $CDCl_3$) δ 177.0, 171.4, 156.8, 135.3, 106.9, 106.3, 74.6, 52.6, 20.5, 18.5 ppm.

Data agree with the literature.[5]

Diethyl 2,2'-((2-(diacetoxy-λ^3-iodanyl)-1,3-phenylene)bis(oxy))(2R,2'R)-dipropionate (19b):

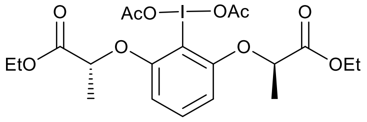

GP3: Diethyl 2,2'-((2-iodo-1,3-phenylene)bis(oxy))(2*R*,2'*R*)-dipropionate (0.5 g, 1.15 mmol, 1 eq.), Selectfluor® (4 g, 11.5 mmol, 10 eq.), AcOH (2 mL), acetonitrile (6 mL). The title product was obtained in (0.63 g, 99%).

$[\alpha]_D^{20}$ = –41.6 (c = 1.25, $CHCl_3$).

^1H NMR (500 MHz, $CDCl_3$) δ 7.38 (t, *J* = 8.4 Hz, 1H), 6.57 (d, *J* = 8.4 Hz, 2H), 4.84 (q, *J* = 6.8 Hz, 2H), 4.25 – 4.18 (m, 4H), 1.97 (s, 6H), 1.67 (d, *J* = 6.8 Hz, 6H), 1.24 (t, *J* = 7.1 Hz, 6 H) ppm.

^{13}C NMR (126 MHz, $CDCl_3$) δ 177.1, 171.0, 156.8, 135.2, 106.8, 106.2, 74.6, 61.7, 20.5, 18.4, 14.1 ppm.

Data agree with the literature.[11]

Diisopropyl 2,2'-((2-(diacetoxy-λ^3-iodanyl)-1,3-phenylene)bis(oxy))(2R,2'R)-dipropionate (19c):

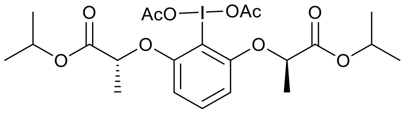

GP3: Diisopropyl 2,2'-((2-iodo-1,3-phenylene)bis(oxy))(2R,2'R)-dipropionate (0.5 g, 1.08 mmol, 1 eq.), Selectfluor® (4 g, 10.8 mmol, 10 eq.), AcOH (2 mL), acetonitrile (6 mL). The title product was obtained as colourless viscous oil (0.62 g, 99%).

$[\alpha]_D^{20} = -370$ (c = 0.40, $CHCl_3$).

^{1}H NMR (500 MHz, $CDCl_3$) δ = 7.36 (t, *J* = 8.4 Hz, 1H), 6.52 (d, *J* = 8.4 Hz, 2H), 5.13 – 4.95 (m, 2H), 4.79 (q, *J* = 6.8 Hz, 2H), 1.95 (s, 6H), 1.63 (d, *J* = 6.8 Hz, 6H), 1.23 (d, *J* = 6.3 Hz, 6H), 1.14 (d, *J* = 6.3 Hz, 6H) ppm.

^{13}C NMR (126 MHz, $CDCl_3$) δ = 177.2, 170.4, 156.7, 135.0, 105.9, 77.1, 74.4, 69.3, 21.7, 21.5, 20.5, 18.2 ppm.

IR (neat): *v* = 2983, 2360, 1732, 1463, 1276, 1095, 667, 553 cm^{-1}.

Bis((1*S*,2*R*,5*S*)-2-isopropyl-5-methylcyclohexyl) 2,2'-((2-(diacetoxy-λ^3-iodanyl)-1,3-phenylene) bis(oxy))(2*R*,2'*R*)- dipropionate (19d):

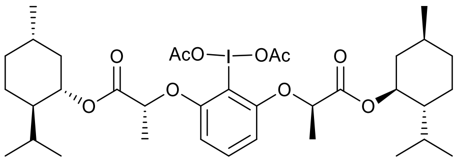

GP3: Dimethyl 2,2'-((2-(diacetoxy-l3-iodaneyl)-1,3-phenylene)bis(oxy)) (2*R*,2'*R*)-dipropionate (0.93 g, 1.42 mmol, 1 eq.), and Selectfluor® (5 g, 14.2 mmol, 10 eq.), MeCN/AcOH (3/1, 24 mL). The title product was formed as colourless viscous liquid (1.1 g, 99%).

$[\alpha]_D^{20} = -101.5$ (c = 1.3, $CHCl_3$).

^{1}H NMR (300 MHz, $CDCl_3$) δ 7.36 (t, *J* = 8.4 Hz, 1H), 6.52 (d, *J* = 8.4 Hz, 2H), 4.80 (q, *J* = 6.7 Hz, 2H), 4.67 (td, *J* = 10.8, 4.3 Hz, 2H), 2.03 (d, *J* = 23.8 Hz, 2H), 1.96 (s, 6H), 1.70 – 1.30 (m br, 16 H), 1.07 – 0.82 (m, 12H), 0.71 (d, *J* = 6.8 Hz, 6H), 0.59 (d, *J* = 6.8 Hz, 6H) ppmn.

^{13}C NMR (75 MHz, $CDCl_3$) δ= 177.1, 170.8, 156.9, 135.0, 106.1, 105.7, 75.8, 74.6, 46.6, 40.6, 34.2, 31.4, 25.6, 22.9, 22.0, 20.8, 20.6, 18.4, 15.8 ppm.

IR (neat): *v* = 2953, 2927, 1728, 1589, 1463, 1132, 1097, 705, 665 cm^{-1}.

(2,6-Bis(((*R*)-1-((4-methylphenyl)sulfonamido)-1-oxopropan-2-yl)oxy)phenyl)-λ^3-iodanediyl diacetate (19e):

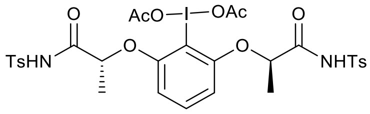

GP3: (2*R*,2'*R*)-2,2'-((2-Iodo-1,3-phenylene)bis(oxy))bis(*N*-tosylpropanamide) (1 g, 1.45 mmol, 1 eq.), and Selectfluor® (5.16 g, 14.5 mmol, 10 eq.) in MeCN/ AcOH (3/1, 24 mL). The title product was formed as yellow viscous oil (1.1 g, 99%).

$[\alpha]_D^{20} = -266.6$ (c = 0.24, $CHCl_3$).

^{1}H NMR (400 MHz, $CDCl_3$) δ= 9.72 (s, 2H), 7.66 (d, *J* = 8.2 Hz, 4H), 7.22 (d, *J* = 8.2 Hz, 4H), 7.04 (t, *J* = 8.4 Hz, 1H), 6.37 (d, *J* = 8.5 Hz, 2H), 4.83 (q, *J* = 6.7 Hz, 2H), 2.41 (s, 6H), 2.18 (s, 6H), 1.66 (d, *J* = 6.7 Hz, 6H) ppm.

^{13}C NMR (101 MHz, $CDCl_3$) δ= 177.8, 169.4, 155.3, 145.2, 136.4, 135.1, 129.5, 128.4, 105.7, 77.3, 75.8, 21.7, 20.4, 18.0 ppm.

IR (neat): v = 3369, 2943, 1718, 1593, 1, 1251, 1159, 1082, 842, 661 cm^{-1}.

(2,6-Bis(((*R*)-1-oxo-1-((perfluorophenyl)amino)propan-2-yl)oxy)phenyl)-λ^3-iodanediyl diacetate (19f):

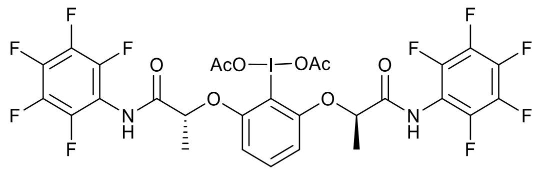

GP3: (2*R*,2'*R*)-2,2'-((2-Iodo-1,3- phenylene) bis(oxy))bis (*N*-(perfluorophenyl) propanamide) (0.5 g, 0.704 mmol, 1 eq.), Selectfluor® (2.5 g, 7.04 mmol, 10 eq.) in MeCN/ AcOH (3/1, 20 mL). After completion of the reaction, the reaction mixture was concentrated under a vacuum, and the resulting crude product was diluted with CH_2Cl_2 (25 mL) and H_2O (25 mL). The organic phase was separated, and the aqueous phase was extracted with CH_2Cl_2 (2 × 30 mL), and the combined organic layers were dried over anhydrous $MgSO_4$ and concentrated under a vacuum. The title product was formed as yellow solid (0.51 g, 89% yield).

m.p. 89−90 ºC.

^1H NMR (400 MHz, $CDCl_3$) δ 8.87 (s, 2H), 7.57 (t, J = 8.4 Hz, 1H), 6.79 (d, J = 8.4 Hz, 2H), 5.12 (q, J = 6.7 Hz, 2H), 1.85 (d, J = 6.7 Hz, 6H), 1.83 (s, 6H) ppm.

^{13}C NMR (126 MHz, $CDCl_3$) δ 176.3, 170.2, 155.2, 144.6 – 144.2 (m), 142.6 – 142.2 (m), 139.1 – 138.5 (m), 136.7, 111.1 (dt, $J_{C\text{-}F}$ = 3.5, 11.6 Hz), 106.2, 77.3, 76.0, 20.1, 18.8 ppm.

Data agree with the literature.[9]

(2,6-Bis(((*R*)-1-((3,5-bis(trifluoromethyl)phenyl)amino)-1-oxopropan-2-yl)oxy)phenyl)-λ^3-iodanediyl diacetate (19g):

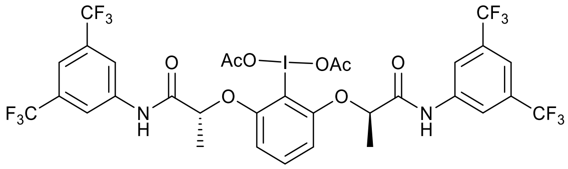

GP3: (2*R*,2'*R*)-2,2'-((2-Iodo-1,3-phenylene)bis(oxy))bis (*N*-(3,5-bis(trifluoromethyl) phenyl)propanamide) (1.0 g, 1.25 mmol, 1 eq.) and Selectfluor® (5.07 g, 12.5 mmol, 10 eq.) in MeCN/ AcOH (3/1, 24 mL). The title product was formed as yellow viscous liquid (1.08 g, 95%).

$[\alpha]_D^{20}$ = −251.5 (c = 0.66, $CHCl_3$).

^1H NMR (500 MHz, $CDCl_3$) δ 9.82 (s, 2H), 8.40 (s, 4H), 7.59 (s, 2H), 7.49 (t, J = 8.4 Hz, 1H), 6.69 (d, J = 8.5 Hz, 2H), 5.01 (q, J = 6.7 Hz, 2H), 2.03 (s, 6H), 1.84 (d, J = 6.7 Hz, 6H) ppm.

^{13}C NMR (126 MHz, $CDCl_3$) δ 176.1, 170.1, 155.0, 139.6, 137.0, 132.1 (q, $J_{C\text{-}F}$ = 33.4 Hz), 123.1 (q, $J_{C\text{-}F}$ = 273.3 Hz), 120.3 (d, $J_{C\text{-}F}$ = 3.1 Hz), 117.8 − 117.7 (m), 111.5, 105.5, 76.3, 20.2, 18.7 ppm.

IR (neat): v = 3307, 1691, 1641, 1544, 1379, 1274 1124, 887, 669 cm^{-1}.

(2,6-Bis(((*R*)-1-((2,6-diisopropylphenyl)amino)-1-oxopropan-2-yl)oxy)phenyl)-λ³-iodanediyl diacetate (19h):

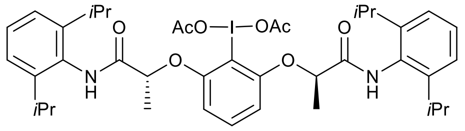

GP3: (2*R*,2'*R*)-2,2'-((2-Iodo-1,3-phenylene)bis(oxy))bis (*N*-(2,6-diisopropylphenyl) propanamide) (1.0 g, 1.431 mmol, 1 eq.), Selectfluor® (5.07 g, 14.31 mmol, 10 eq.), MeCN/ AcOH (3/1, 24 mL). The title product was formed as a colourless solid (1.1 g, 94%).

$[\alpha]_D^{20}$ = –37.5 (c = 0.4, $CHCl_3$).

^{1}H NMR (300 MHz, $CDCl_3$) δ= 8.24 (s, 2H), 7.66 (t, *J* = 8.4 Hz, 1H), 7.26 (t, *J* = 7.7 Hz, 2H), 7.10 (br, 4H), 6.98 (d, *J* = 8.5 Hz, 2H), 5.16 (q, *J* = 6.7 Hz, 2H), 3.01 (br, 2H), 2.19 (br, 2H), 1.89 (d, *J* = 6.7 Hz, 6H), 1.42 (s, 6H), 1.14 (br, 12H), 0.96 (br, 6H), 0.70 (b, 6H) ppm.

^{13}C NMR (75 MHz, $CDCl_3$) δ= 176.8, 170.6, 156.2, 146.2, 136.4, 129.8, 128.9, 123.6, 106.2, 105.2, 76.5, 28.6, 23.8, 23.4, 19.7, 19.4 ppm.

Data agree with the literature.[2]

(2,6-Bis(((*R*)-1-oxo-1-(((*R*)-1-phenylethyl)amino)propan-2-yl)oxy)phenyl)-λ³-iodanediyl diacetate (19i):

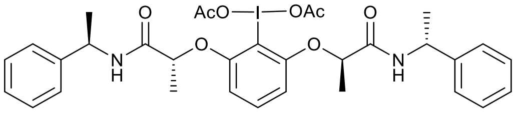

GP3: (2*R*,2'*R*)-2,2'-((2-Iodo-1,3- phenylene) bis(oxy)) bis(*N*-((*R*)-1-phenylethyl) propanamide) **S12** (1 g, 1.70 mmol, 1 eq.), Selectfluor® (6 g, 17.0 mmol, 10 eq.), MeCN/ AcOH (3/1, 24 mL). The title product was formed as yellow viscous liquid (1.1 g, 99%).

$[\alpha]_D^{20}$ = −58.0 (c = 0.16, $CHCl_3$).

^{1}H NMR (400 MHz, $CDCl_3$) δ 7.51 (t, *J* = 8.4 Hz, 1H), 7.38 – 7.27 (m, 8H), 7.24 (s, 1H), 7.17 (d, *J* = 8.5 Hz, 2H), 6.73 (d, *J* = 8.5 Hz, 2H), 5.20 – 5.12 (m, 2H), 4.92 (q, *J* = 6.7 Hz, 2H), 1.68 (d, *J* = 6.7 Hz, 6H), 1.64 (s, 6H), 1.28 (d, *J* = 7.0 Hz, 6H) ppm.

^{13}C NMR (126 MHz, $CDCl_3$) δ 177.0, 176.7, 170.4, 155.7, 142.7, 136.0, 128.8, 127.5, 126.2, 106.3, 76.4, 48.1, 21.2, 20.0, 18.8 ppm.

IR (neat): v = 2980, 2905, 1700, 1687, 1374, 1187, 1132, 753, 698 cm^{-1}.

(2,6-Bis(((*S*)-1-oxo-1-(((*S*)-1-phenylethyl)amino)propan-2-yl)oxy)phenyl)-λ³-iodanediyl diacetate (*ent*-19i):

GP3: (2*S*,2'*S*)-2,2'-((2-Iodo-1,3-phenylene)bis(oxy))bis(*N*-((*S*)-1-phenylethyl)propanamide) *ent*-**S12** (3.71 g, 6.33 mmol), Selectfluor® (22.4 g, 63.3 mmol, 10 eq.), MeCN/ AcOH (3/1, 100 mL). The title product was formed as yellow viscous liquid (4.01 g, 5.70 mmol, 90%).

$[\alpha]_D^{20}$ = +41.0 (c = 0.536, $CHCl_3$).

(2,6-Bis(((*R*)-1-oxo-1-(((*S*)-1-phenylethyl)amino)propan-2-yl)oxy)phenyl)-λ^3-iodanediyl diacetate (19j):

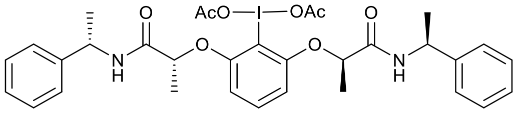

GP3: (2*R*,2'*R*)-2,2'-((2-Iodo-1,3- phenylene)bis(oxy))bis(*N*-((*S*)-1-phenylethyl)propanamide) (1 g, 1.70 mmol, 1 eq.), Selectfluor® (6 g, 17.0 mmol, 10 eq.), MeCN/ AcOH (3/1, 24 mL). The title product was formed as yellow viscous liquid (1.1 g, 99%).

$[\alpha]_D^{20} = -100.0$ (c = 0.20, $CHCl_3$).

^{1}H NMR (400 MHz, $CDCl_3$) δ= 7.51 (t, *J* = 8.4 Hz, 1H), 7.38 – 7.27 (m, 9H), 7.23 (d, *J* = 5.3 Hz, 1H), 7.17 (d, *J* = 8.4 Hz, 2H), 6.73 (d, *J* = 8.5 Hz, 2H), 5.20 – 5.11 (m, 2H), 4.92 (q, *J* = 6.7 Hz, 2H), 1.68 (d, *J* = 6.7 Hz, 6H), 1.64 (s, 6H), 1.28 (d, *J* = 7.0 Hz, 6H) ppm.

^{13}C NMR (126 MHz, $CDCl_3$) δ= 176.7, 176.4, 170.3, 155.7, 142.7, 136.0, 128.8, 127.5, 126.1, 106.3, 76.4, 48.1, 21.2, 20.0, 18.8 ppm.

IR (neat): v = 3001, 2900, 1718, 1687, 1374, 1187, 1132, 753, 698 cm^{-1}.

Synthesis of alkynes

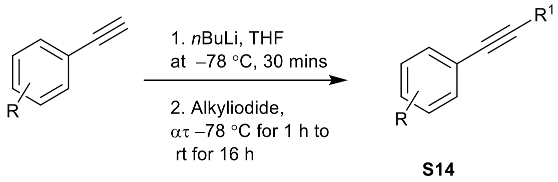

1-Methoxy-4-(prop-1-yn-1-yl)benzene (15):

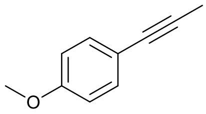

GP4: 1-Ethynyl-4-methoxybenzene (3 g, 22.70 mmol, 1 eq.), *n*-BuLi solution (13.5 mL, 2.5 M in hexane, 34.05 mmol, 1.5 eq.), MeI (10.5 g, 4.60 mL, 68.1 mmol. 3 eq.) in 100 mL THF. The crude product was purified to afford the title product as colourless oil (2.3 g, 70 % yield).

^{1}H NMR (300 MHz, $CDCl_3$) δ 7.33 (d, *J* = 8.9 Hz, 2H), 6.81 (d, *J* = 8.9 Hz, 2H), 3.79 (s, 3H), 2.03 (s, 3H) ppm.

^{13}C NMR (75 MHz, $CDCl_3$) δ= 159.1, 132.9, 116.3, 113.9, 84.2, 79.5, 55.3, 4.4 ppm.

Data agree with the literature.[3]

1-Methoxy-2-methyl-4-(prop-1-yn-1-yl)benzene (S14a):

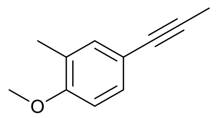

GP4: 1-Ethynyl-4-methoxybenzene (3 g, 22.70 mmol, 1 eq.), *n*-BuLi solution (13.5 mL, 2.5 M, 34.05 mmol, 1.5 eq.), MeI (10.5 g, 4.60 mL, 68.1 mmol. 3 eq.) in 100 mL THF. The crude product was purified to afford the title product as yellow oil (0.85 g, 25% yield).

^{1}H NMR (300 MHz, $CDCl_3$) δ = 7.24 – 7.16 (m, 2H), 6.72 (d, J = 8.3 Hz, 2H), 3.82 (s, 3H), 2.17 (s, 3H), 2.03 (s, 3H) ppm.

^{13}C NMR (75 MHz, $CDCl_3$) δ = 157.4, 133.8, 130.3, 126.7, 115.7, 109.8, 83.8, 79.7, 55.4, 16.1, 4.4 ppm.

IR (neat): v= 2914, 2358, 2341, 1498, 1223, 1132,1033, 810, 650 cm^{-1}.

HRMS (EC-EI): m/z calcd for $C_{11}H_{12}O$: 160.0882 $[M]^+$; found 160.0880.

1-Methoxy-2-(prop-1-yn-1-yl)benzene (S14b):

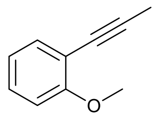

GP4: 1-Ethynyl-3-methoxybenzene (1 g, 1 mL, 7.57 mmol, 1 eq.), *n*-BuLi solution (4.5 mL, 2.5 M, 11.35 mmol, 1.5 eq.), MeI (3.22 g, 1.40 mL, 22.70 mmol, 3.0 eq.) in 100 mL THF. The crude product was purified to afford the title product as colourless oil (0.88 g, 80% yield).

^{1}H NMR (300 MHz, $CDCl_3$) δ 7.38 (dd, J = 7.5, 1.7 Hz, 1H), 7.28 – 7.21 (m, 1H), 6.92 – 6.82 (m, 2H), 3.88 (s, 3H), 2.12 (s, 3H) ppm.

^{13}C NMR (75 MHz, $CDCl_3$) δ 159.8, 133.7, 129.0, 120.5, 113.1, 110.5, 90.1, 75.9, 55.8, 4.9 ppm.

Data agree with the literature.[12]

1-(But-1-yn-1-yl)-4-methoxybenzene (S14c):

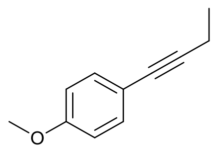

In a dry 50 mL round bottom flask under nitrogen atmosphere, a THF (20 mL) solution of 1-ethynyl-4-methoxybenzene (0.50 g, 3.8 mmol, 1 eq.) was added at −40 °C. Then, *n*-butyllithium (2.5 M, 2.2 mL, 5.49 mmol, 1.5 eq.) was added dropwise at −40 °C. After stirring at this temperature for 30 min, HMPA (0.17 mL, 1.1 mmol, 0.3 eq.) was added, and the reaction allowed to warm to room temperature over the course of 1 h. Subsequently, iodoethane (1.7 g, 0.88 mL, 10.98 mmol, 3 eq.) was added to the solution and the mixture was heated at reflux temperature for 24 h. The reaction was then quenched with a saturated aqueous NH_4Cl solution. The aqueous solution was extracted with ethyl acetate (3 × 10 mL), and the organic phase dried over $MgSO_4$, and evaporated to dryness. The residues were purified through a silica gel column to afford title product as pure colourless solid 95% yield (0.58 g).[13]

^{1}H NMR (400 MHz, $CDCl_3$) δ 7.33 (d, J = 8.8 Hz, 2H), 6.81 (d, J = 8.7 Hz, 2H), 3.80 (s, 3H), 2.40 (q, J = 7.5 Hz, 2H), 1.23 (t, J = 7.5 Hz, 3H) ppm.

^{13}C NMR (101 MHz, $CDCl_3$) δ 159.1, 132.9, 116.2, 113.9, 90.1, 79.6, 55.3, 14.1, 13.2 ppm.

Data agree with the literature.[14]

Synthesis of 2,4-dimethoxy-1-(prop-1-yn-1-yl)benzene:

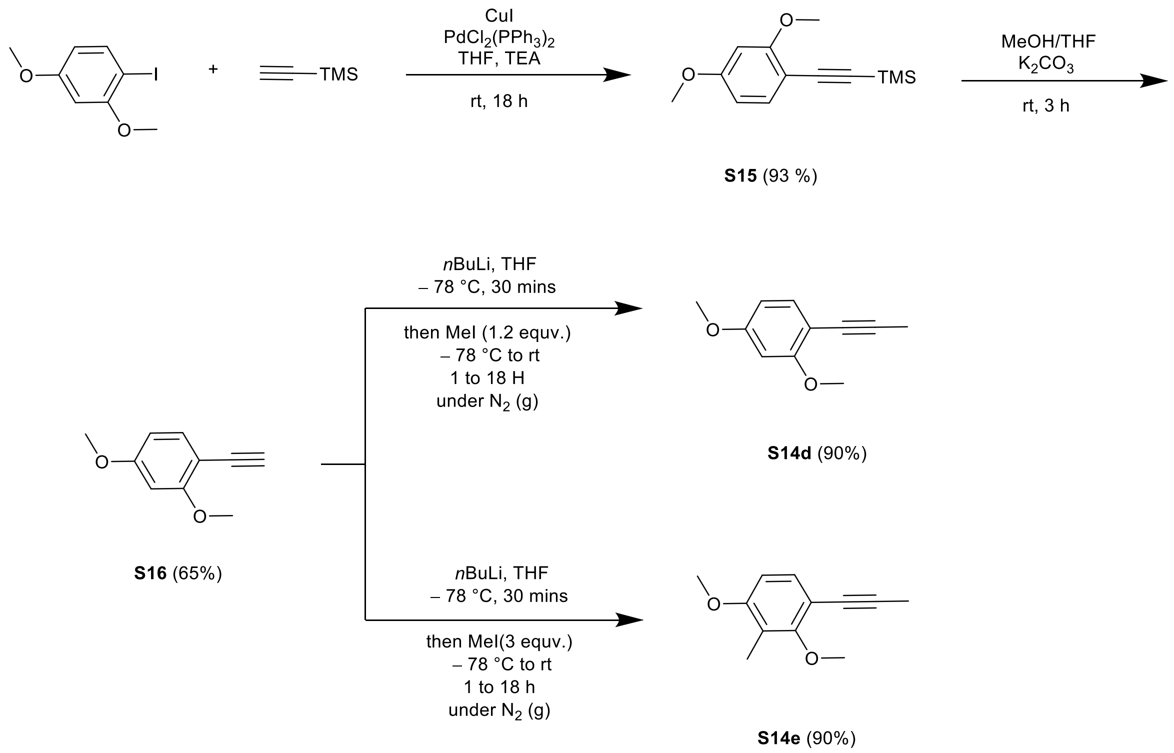

Figure S2. Synthesis of internal alkynes of **S14d** and **S14e**.

(2,4-Dimethoxyphenyl)ethynyl)trimethylsilane (S15)

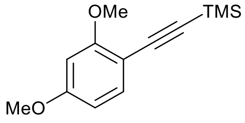

Following the reported procedure,[15] to a solution of 1-iodo-2,4-dimethoxybenzene (2 g, 7.58 mmol, 1 eq.), and Et_3N in THF (40 mL) (stirring and deoxygenation for 10 min under N_2 gas). Then, $PdCl_2(PPh_3)_2$ (0.11 g, 0.15 mmol, 2 mol %), and CuI (0.03 g, 0.15 mmol, 2 mol %) were added in one portion and stirred for 5 min under nitrogen atmosphere. Then, trimethylsilyl acetylene (0.90 g, 1.3 mL, 9.1 mmol, 1.2 eq.) in TEA (5 mL) was added dropwise over 5 min. The reaction flask was flushed with N_2 and the mixture was stirred at room temperature for 18 h. The resulting solution was concentrated under vacuum to yield the crude product which was purified by flash chromatography (petroleum ether:ethyl acetate 95:5) afforded the title product in 93% yield (1.65 g) as a brown solid.

^{1}H NMR (400 MHz, $CDCl_3$) δ = 7.36 (d, J = 8.2 Hz, 1H), 6.44 – 6.38 (m, 2H), 3.85 (s, 3H), 3.81 (s, 3H), 0.25 (s, 9H) ppm.

^{13}C NMR (101 MHz, $CDCl_3$) δ = 161.7, 161.4, 135.1, 105.0, 104.8, 101.5, 98.4, 96.8, 55.9, 55.5, 0.3 ppm.

Data agree with the literature.[15]

1-Ethynyl-2,4-dimethoxybenzene (S16)

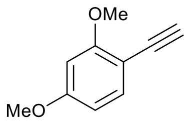

Following the reported procedure,[15,16] in a round bottom flask equipped with a stir bar ((2,4 dimethoxyphenyl)ethynyl)trimethylsilane (1 g, mmol, 1.0 eq.) and potassium carbonate (0.282 g, 2.04 mmol, 2 eq.) were dissolved in methanol and tetrahydrofuran (1:1, 50 mL). The reaction was stirred for 3 hours at room temperature and then concentrated under reduced pressure. The crude product was purified by column chromatography (petroleum ether: ethyl acetate/ 9.5:0.5), the desired product was obtained as a colourless oil (0.45 g, 65 % yield).

^{1}H NMR (400 MHz, $CDCl_3$) δ = 7.38 (d, *J* = 8.9 Hz, 1H), 6.45 (dd, *J* = 7.3, 2.1 Hz, 2H), 3.88 (s, 3H), 3.82 (s, 3H), 3.23 (s, 1H) ppm.

^{13}C NMR (101 MHz, $CDCl_3$) δ = 161.9, 161.6, 135.1, 104.9, 103.8, 98.5, 80.3, 79.7, 55.9, 55.6 ppm.

Data agree with the literature.[15]

2,4-Dimethoxy-1-(prop-1-yn-1-yl)benzene (S14d):

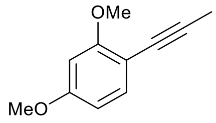

GP4: 1-Ethynyl-2,4-dimethoxybenzene (1.3 g, 8.02 mmol, 1 eq.), *n*-BuLi solution (4.8 mL, 2.5 M, 12.03 mmol, 1.5 eq.), MeI (1.25 g, 0.55 mL, 8.82 mmol. 1.1 eq.) in 30 mL THF. The crude product was purified to afford the title product as colourless oil (1.3 g, 92% yield).

^{1}H NMR (500 MHz, $CDCl_3$) δ 7.22 (d, *J* = 8.8 Hz, 1H), 6.35 (s, 2H), 3.78 (s, 3H), 3.72 (s, 3H), 2.02 (s, 3H) ppm.

^{13}C NMR (126 MHz, $CDCl_3$) δ 161.0, 160.5, 134.2, 105.6, 104.7, 98.4, 88.2, 75.6, 55.8, 55.4, 4.8 ppm.

Data agree with the literature.[17]

1,3-Dimethoxy-2-methyl-4-(prop-1-yn-1-yl)benzene (S14e):

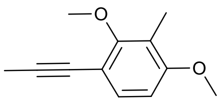

GP4: (1.3 g, 8.02 mmol, 1 eq.), *n*-BuLi solution (4.8 mL, 2.5 M, 34.05 mmol, 1.5 eq.), MeI (3.41 g, 1.5 mL, 68.1 mmol. 3 eq.) in 30 mL THF. The crude product was purified to afford the title product as colourless oil (1.53 g, 90% yield).

^{1}H NMR (400 MHz, $CDCl_3$) δ = 7.19 (d, *J* = 8.5 Hz, 1H), 6.55 (d, *J* = 8.5 Hz, 1H), 3.86 (s, 3H), 3.81 (s, 3H), 2.12 (s, 3H), 2.08 (s, 3H) ppm.

^{13}C NMR (101 MHz, $CDCl_3$) δ = 160.0, 158.4, 130.9, 119.9, 110.1, 105.9, 88.0, 76.1, 60.6, 55.7, 8.9, 4.7 ppm.

IR (neat): v = 2935, 1595, 1485, 1271, 1100, 800, 696 cm^{-1}.

HRMS (ESP-TOF): m/z calcd for $C_{12}H_{15}O_2+H^+$: 191.1072 $[M+H]^+$; found 191.1073.

2-Methoxy-6-(prop-1-yn-1-yl)naphthalene (25):

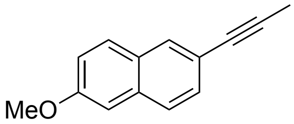

In variation of a literature procedure,[18] 2-ethynyl-6-methoxynaphthalene (2 g, 10.975 mmol, 1 eq.) was deprotonated in dry THF (20 mL) by addition of *n*BuLi solution (2.5 M in hexane, 7 mL, 17.56 mmol, 1.6 eq., diluted with dry THF (5 mL) and precooled to –78 ºC), slowly at –78 ºC under nitrogen atmosphere. 10 min after the addition, the mixture was allowed to warm up to room temperature and stirred for 30 min. Methyl iodide (1 mL, 16.46 mmol, 1.5 eq., diluted with THF (5 mL) and precooled to –78 ºC) was added to the reaction mixture at –78 ºC. The reaction was stirred for 16 h at room temperature. The reaction was quenched with aq. Sat. NH_4Cl solution (200 mL), extracted with ethyl acetate (3 × 150 mL) and the combined organic phases dried over anhydrous $MgSO_4$, filtered and concentrated under reduced pressure. The crude mixture was purified by flash column chromatography on silica gel with petroleum ether to afford **7a** in 72% yield (1.438 g, 7.9 mmol) as colourless solid powder.

^1H NMR (500 MHz, $CDCl_3$) δ = 7.75 (s, 1H), 7.58 (dd, J = 13.1, 8.7 Hz, 2H), 7.34 (dd, J = 8.4, 1.7 Hz, 1H), 7.06 (d, J = 8.9 Hz, 1H), 7.02 (d, J = 2.5 Hz, 1H), 3.85 (s, 3H), 2.02 (s, 3H) ppm.

^{13}C NMR (101 MHz, $CDCl_3$) δ = 158.0, 133.7, 130.8, 129.2, 129.1, 128.5, 126.6, 119.2, 119.0, 105.7, 85.3, 80.1, 55.3, 4.4 ppm.

Data agree with the literature.[18]

The following arylalkynes **S26** did not react under the established reaction conditions and were recovered after without decomposition. **S26a** is commercially available.

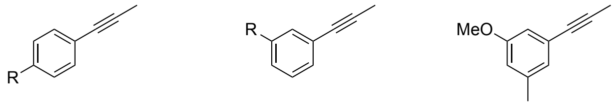

S26a R = H
S26b R = Me
S26c R = F
S26d R = CF_3
S26e R = NMe_2

S26f R = Cl
S26g R = OMe

S26h

1-Methyl-4-(prop-1-yn-1-yl)benzene (S26b)

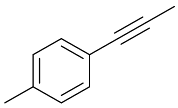

Following GP7, 1-ethynyl-4-methylbenzene (0.92 g, 1.0 mL, 7.88 mmol, 1 equiv.), *n*-BuLi solution (4.73 mL, 2.5 M, 11.83 mmol, 1.5 equiv.), MeI (3.36 g, 1.50 mL, 17.61 mmol, 3.0 equiv.) in 100 mL THF. The crude product was purified to afford the title product as yellow oil (0.95 g, 92% yield).

^{1}H NMR (400 MHz, $CDCl_3$) δ = 7.29 (d, J = 8.0 Hz, 2H), 7.09 (d, J = 7.9 Hz, 2H), 2.33 (s, 3H), 2.04 (s, 3H) ppm.

^{13}C{^{1}H} NMR (101 MHz, $CDCl_3$) δ = 137.6, 131.4, 129.1, 121.0, 85.0, 79.8, 21.5, 4.4 ppm.

Data are in agreement with the literature.[19]

1-Fluoro-4-(prop-1-yn-1-yl)benzene (S26c)

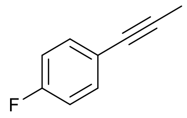

Following GP7, 1-ethynyl-4-fluorobenzene (0.91 g, 7.57 mmol, 1 equiv.), *n*-BuLi solution (4.5 mL, 2.5 M, 11.35 mmol, 1.5 equiv.), MeI (3.22 g, 1.40 mL, 22.70 mmol, 3.0 equiv.) in 50 mL THF. The crude product was purified to afford the title product as colorless oil (0.85 g, 85% yield).

^{1}H NMR (400 MHz, $CDCl_3$) δ = 7.35 (dd, J = 8.4, 5.6 Hz, 2H), 6.96 (t, J = 8.7 Hz, 2H), 2.03 (s, 3H) ppm.

^{13}C{^{1}H} NMR (101 MHz, $CDCl_3$) δ = 162.8 (d, $J_{C\text{-}F}$ = 249.5 Hz), 133.2 (d, $J_{C\text{-}F}$ = 8.0 Hz), 120.1 (d, $J_{C\text{-}F}$ = 3.3 Hz), 115.4 (d, $J_{C\text{-}F}$ = 22.2 Hz), 85.3, 78.6, 4.0 ppm.

Data are in agreement with the literature.[20]

1-(Prop-1-yn-1-yl)-4-(trifluoromethyl)benzene (S26d)

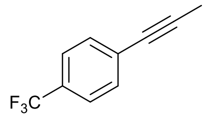

Following GP7, 1-ethynyl-4-(trifluoromethyl)benzene (1 g, 0.82 mL, 5.87 mmol, 1 equiv.), *n*-BuLi solution (5.16 mL, 2.5 M, 8.81 mmol, 1.5 equiv.), MeI (3 g, 1.20 mL, 17.61 mmol, 3.0 equiv.) in 100 mL THF. The crude product was purified to afford the title product as yellow oil (900 mg, 83% yield).

^{1}H NMR (500 MHz, $CDCl_3$) δ = 7.53 (d, J = 8.2 Hz, 2H), 7.48 (d, J = 8.2 Hz, 2H), 2.07 (s, 3H) ppm.

^{13}C{^{1}H} NMR (126 MHz, $CDCl_3$) δ = 134.8, 131.8, 129.4 (q, J = 32.6 Hz), 128.1 (q, J = 99.5 Hz), 125.2 (q, J = 3.8 Hz), 88.8, 78.8, 4.5 ppm.

Data are in agreement with the literature.[21]

***N,N*-Dimethyl-4-(prop-1-yn-1-yl)aniline (S26e)**

Following GP7, 4-ethynyl-*N,N*-dimethylaniline (0.5 g, 3.44 mmol, 1 equiv.), *n*-BuLi solution (2.0 mL, 2.5 M, 5.17 mmol, 1.5 equiv.), MeI (1.47 g, 0.65 mL, 10.33 mmol, 3.0 equiv.) in 20 mL THF. The crude product was purified to afford the title product as brown solid (0.509 g, 93% yield).

^{1}H NMR (500 MHz, $CDCl_3$) δ = 7.28 (d, J =8.9 Hz, 2H), 6.62 (d, J = 8.9 Hz, 2H), 2.95 (s, 6H), 2.04 (s, 3H) ppm.

^{13}C{^{1}H} NMR (126 MHz, $CDCl_3$) δ = 149.8, 132.5, 112.1, 111.3, 83.1, 80.3, 40.4, 4.5 ppm.

Data are in agreement with the literature.[22]

1-Chloro-3-(prop-1-yn-1-yl)benzene (S26f)

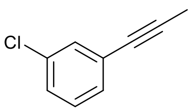

Following GP7, 1-ethynyl-3-(chloromethyl)benzene (1 g, 7.32 mmol, 1 equiv.), *n*-BuLi solution (4.4 mL, 2.5 M, 11.00 mmol, 1.5 equiv.), MeI (3.11 g, 1.37 mL, 22.0 mmol, 3.0 equiv.) in 50 mL THF. The crude product was purified to afford the title product as brown oil (1.0 g, 90% yield).

^{1}H NMR (400 MHz, $CDCl_3$) δ = 7.37 (t, *J* = 1.8 Hz, 1H), 7.30 – 7.18(m, 3H), 2.06 (s, 3H).

^{13}C{^{1}H} NMR (101 MHz, CDCl3) δ = 134.2, 131.6, 129.8, 129.4, 128.1, 125.9, 87.4, 78.6, 4.5 ppm.

Data are in agreement with the literature.[23]

1-Methoxy-3-(prop-1-yn-1-yl)benzene (S26g)

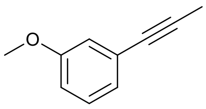

Following GP7, 1-ethynyl-3-methoxybenzene (1 g, 1 mL, 7.57 mmol, 1 equiv.), *n*-BuLi solution (4.5 mL, 2.5 M, 11.35 mmol, 1.5 equiv.), MeI (3.22 g, 1.40 mL, 22.70 mmol, 3.0 equiv.) in 50 mL THF. The crude product was purified to afford the title product as colorless oil (0.88 g, 80 % yield).

^{1}H NMR (300 MHz, $CDCl_3$) δ = 7.19 (t, *J* = 7.8 Hz, 1H), 6.99 (dt, *J* = 7.6, 1.1 Hz, 1H), 6.94 – 6.91 (m, 1H), 6.86 – 6.80 (m, 1H), 3.79 (s, 3H), 2.05 (s, 3H) ppm.

^{13}C{^{1}H} NMR (75 MHz, $CDCl_3$) δ = 159.3, 129.3, 125.1, 124.1, 116.4, 114.2, 85.8, 79.7, 55.3, 4.4 ppm.

Data are in agreement with the literature.[24]

1-Methoxy-3-methyl-5-(prop-1-yn-1-yl)benzene (S26h)

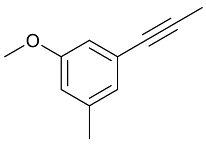

Following GP7, 1-ethynyl-3-methoxybenzene (1 g, 1 mL, 7.57 mmol, 1 equiv.), *n*-BuLi solution (4.5 mL, 2.5 M, 11.35 mmol, 1.5 equiv.), MeI (3.22 g, 1.40 mL, 22.70 mmol, 3.0 equiv.) in 50 mL THF. The crude product was purified to afford the title product as colorless oil (0.15 g, 14% yield).

^{1}H NMR (300 MHz, $CDCl_3$) δ = 7.03 (d, *J* = 7.6 Hz, 1H), 6.91 (dd, *J* = 7.6, 1.2 Hz, 1H), 6.85 (s, 1H), 3.81 (s, 3H), 2.20 (s, 3H), 2.05 (s, 3H) ppm.

^{13}C{^{1}H} NMR (75 MHz, $CDCl_3$) δ = 157.4, 130.5, 126.8, 123.7, 122.2, 112.9, 84.8, 80.0, 55.3, 16.3, 4.4 ppm.

IR (neat): *v* = 2910, 2308, 2341, 1500, 1233, 1132,1023, 780, 663 cm^{-1}.

HRMS (EC-EI) m/z: $[M]^+$ calcd for $C_{11}H_{12}O$ 160.0872; found 160.0870.

Reaction Optimisation Data

Table S1: Control experiments.[a]

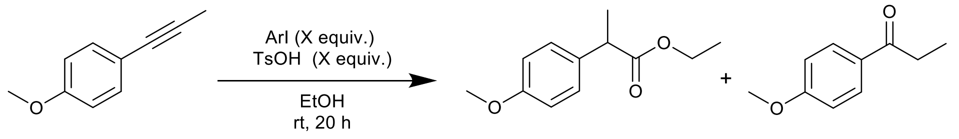

15 16b S17

	ArI	Additive	Solvent	Yield [%][b]		
				**15**	**16b**	**S17**
1	PhI (1 equiv.)	-	EtOH	99	0	0
2	-	*p*TsOH (1 equiv.)	EtOH	33	0	66

[a] General method: **15** (0.3 mmol), PhI, *p*TsOH in EtOH (1 mL) at 25 ºC, 20 h.
[b] ^{1}H NMR yield determined using 1,3,5-trimethoxybenzene as an internal standard.

Table S2. Preliminary optimisation of the reaction conditions for the oxidative rearrangement of alkyne **15**.[a]

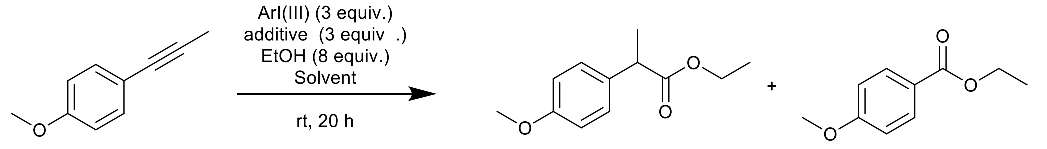

15 **16b** **18**

Entry	ArI(III)	Additives	Solvent	Yield [%][b]		
				**15**	**16b**	**18**
1	PhI(OH)(OTs)	-	EtOH	80	0	0
2	$PhI(OAc)_2$	-	EtOH	90	0	0
3	$PhI(OCOCF_3)_2$	-	EtOH	90	0	0
4	PhI(OH)(OTs)	-	HFIP	60	trace	15
5	$PhI(OAc)_2$	-	HFIP	70	trace	trace
6	$PhI(OCOCF_3)_2$	-	HFIP	70	trace	trace
7	PhI(OH)(O*p*Ts)	*p*TsOH•H_2O	HFIP	32	13	16
8	$PhI(OAc)_2$	*p*TsOH•H_2O	HFIP	28	15	trace
9	$PhI(OCOCF_3)_2$	*p*TsOH•H_2O	HFIP	28	20	trace
10	$PhI(OCOCF_3)_2$	*p*TsOH anhydrous	HFIP	25	25	0
11[c]	$PhI(OCOCF_3)_2$	*p*TsOH•H_2O	HFIP, EtOH	25	25	0
12[d]	$PhI(OCOCF_3)_2$	*p*TsOH anhydrous	HFIP, dry EtOH	25	25	0
13[d]	1. $PhI(OCOCF_3)_2$, *p*TsOH, HFIP, for 1 h<br>2. **15**, EtOH			25	25	0

[a] All reactions were carried out with **15** (0.5 mmol), ArI(III) (1.5 mmol), *p*TsOH•H_2O or *p*TsOH (1.5 mmol), EtOH (4 mmol) in solvent (1 mL), at 25 ºC, 20 h.
[b] Isolated yields.
[c] under air.
[d] under N_2 atmosphere.

Table S3: Solvent optimisation for oxidative rearrangement of alkyne **15**.[a]

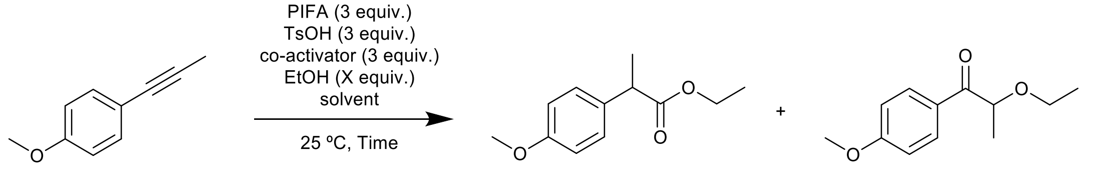

15 **16b** **17**

Entry	Solvent	EtOH [equiv.]	Time [h]	Co-activator	Yield [%][b]		
					**15**	**16b**	**17**
1	HFIP	8	20	-	20	25	0
2		20	20	-	20	38	0
3		30	20	-	20	37	0
4	TFE	30	20	-	20	37	0
5	$CHCl_3$	30	20	-	8	21	9
6	CH_2Cl_2	30	20	-	10	9	0
7	acetone	30	20	-	0	0	0
8	DMSO	30	20	-	0	0	0
9	DMF	30	20	-	6	4	0
10	toluene	30	20	-	13	37	0
11	THF	30	20	-	3	37	0
12	MeCN	30	20	-	2	34	0
13	Et_2O	30	20	-	2	44	0
14	EtOH	30	20		14	41	4
15	EtOH	-	40	-	0	20	53 (47)[c]
16	EtOH	-	20	BF_3•OEt_2 (3 equiv)	9	64 (61)[c]	0

[a] General method: PIFA (0.9 mmol) and *p*TsOH (0.9 mmol) in solvent (1 mL) for 1 h. Then, **15** (0.3 mmol), EtOH (X mmol), and BF_3•OEt_2 (0.9 mmol) at 25 °C.
[b] ^{1}H NMR yield determined using 1,3,5-trimethoxybenzene as an internal standard.
[c] Isolated yields.

Table S4: Hypervalent iodine reagent and additive optimisation for oxidative rearrangement of alkyne **15**.[a]

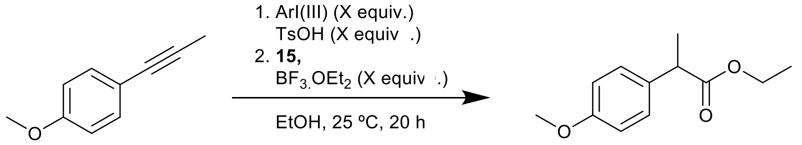

15 **16b**

Entry	ArI(III)	TsOH [equiv.]	BF_3•OEt_2 [equiv.]	Yield [%][b]
1	$PhI(OCOCF_3)_2$	3	3	61
2	$PhI(OAc)_2$	3	3	58
3	$PhI(OAc)_2$	1.5	1.5	38
4	$PhI(OAc)_2$	3	-	87
5	$PhI(OAc)_2$	1.5	-	90
6	$PhI(OAc)_2$	1.3	-	78

[a] General method: ArI(III) and *p*TsOH in EtOH (1 mL) for 1 h. Then, **15** (0.3 mmol) and BF_3•OEt_2 (0.9 mmol) at 25 °C, for 20 h.
[b] Isolated yields.

Reaction products

Methyl (*R*)-2-(4-methoxyphenyl)propanoate (16a):

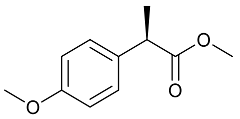

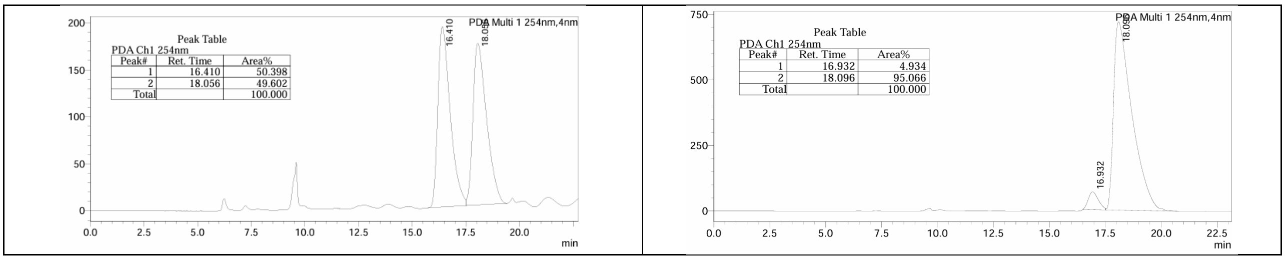

GP5, Method B: (Diacetoxyiodo)benzene (0.48 g, 1.5 mmol, 1.5 eq.) and anhydrous *p*TsOH (0.26 g, 1.5 mmol, 1.5 eq.) were dissolved in 1.5 mL of dry methanol and stirred for one hour at room temperature followed by the addition of 1-methoxy-4-(prop-1-yn-1-yl)benzene (0.14 g, 1 mmol, 1 eq.) and BF_3•Et_2O (0.21 g, 0.16 mL, 1.5 mmol, 1.5 eq.). The racemic product was formed as a colourless oil (169 mg, 87% yield).

GP5, Method B: (Diacetoxyiodo)arene **19j** (1.06 g, 1.5 mmol, 1.5 eq.) and anhydrous *p*TsOH (0.26 g, 1.5 mmol, 1.5 eq.) were dissolved in 2 mL of dry methanol and stirred for one hour at room temperature followed

by the addition of 1-methoxy-4-(prop-1-yn-1-yl)benzene (0.14 g, 1 mmol, 1 eq.) and $BF_3 \bullet Et_2O$ (0.21 g, 0.16 mL, 1.5 mmol, 1.5 eq.). The chiral product was formed as a colourless oil (163 mg, 84% yield, 90% *ee*).

The title product determined as (*R*)-isomer according to reported literature.[25]

$[\alpha]_D^{20} = -40$ (c = 0.6, $CHCl_3$).

HPLC data: Diacel CHIRALCEL® OD-H 5 μm, 25 cm (*n*-hexane/*i*PrOH = 99.6/0.4, flow rate = 0.5 mL/min, 254 nm). Minor isomer t_R = 16.90 min, major isomer t_R = 18.09 min.

^{1}H NMR (400 MHz, $CDCl_3$) δ = 7.22 (d, *J* = 8.7 Hz, 2H), 6.86 (d, *J* = 8.8 Hz, 2H), 3.79 (s, 3H), 3.69 (q, *J* = 7.2 Hz, 1H), 3.65 (s, 3H), 1.48 (d, *J* = 7.2 Hz, 3H) ppm.

^{13}C NMR (101 MHz, $CDCl_3$) δ = 175.4, 158.8, 132.8, 128.6, 114.1, 55.4, 52.1, 44.7, 18.7 ppm.

HRMS (EC-CI): m/z calcd for $C_{11}H_{14}O_3$: 194.0937 $[M]^+$; found 194.0938.

Data agree with the literature.[25]

Ethyl (*R*)-2-(4-methoxyphenyl)propanoate (16b):

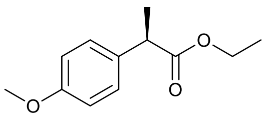

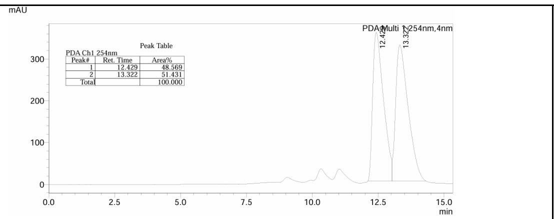

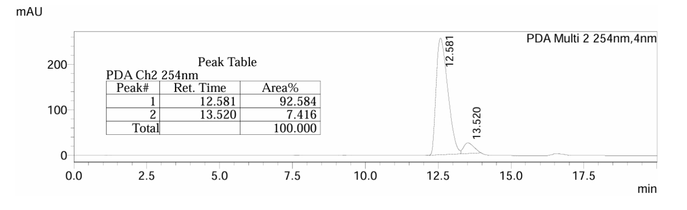

GP5, Method A: (Diacetoxyiodo)benzene (0.48 g, 1.5 mmol, 1.5 eq.) and anhydrous *p*TsOH (0.26 g, 1.5 mmol, 1.5 eq.) were dissolved in dry ethanol (1.5 mL) and stirred for one hour at room temperature, followed by the addition of 1-methoxy-4-(prop-1-yn-1-yl)benzene (0.14 g, 1 mmol, 1 eq.). The racemic product was formed as a colourless oil (187 mg, 90% yield).

GP5, Method A: (Diacetoxyiodo)arene **19j** (1.06 g, 1.5 mmol, 1.5 eq.) and anhydrous *p*TsOH (0.26 g, 1.5 mmol, 1.5 eq.) were dissolved in dry ethanol (2 mL) and stirred for one hour at room temperature, followed by the addition of 1-methoxy-4-(prop-1-yn-1-yl)benzene (0.14 g, 1 mmol, 1 eq.). The chiral product was formed as a colourless oil (187 mg, 90% yield, 85% *ee*).

Large scale: GP5, Method A: (Diacetoxyiodo)arene **19j** (7.23 g, 10.26 mmol, 1.5 eq.) and anhydrous *p*TsOH (1.77 g, 10.26 mmol, 1.5 eq.) were dissolved in dry ethanol (20 mL) and stirred for one hour at room temperature, followed by the addition of 1-methoxy-4-(prop-1-yn-1-yl)benzene (1.0 g, 6.84 mmol, 1 eq.). The chiral product was formed as a colourless oil (1.09 g, 77% yield, 85% *ee*). 90 mg (0.616 mmol) of starting material **15** were recovered after the reaction.

Recycled iodoarene: Following GP3, the recovered iodoarene **S13** was re-oxidized to **19i**: **S13** (1 g, 1.70 mmol, 1 eq.), Selectfluor® (6 g, 17.0 mmol, 10 eq.), MeCN/AcOH (3/1, 24 mL). The title product was formed as yellow viscous liquid (1.1 g, 99%). Following GP5, method A, **19j** (1.06 g, 1.5 mmol, 1.5 eq.) and anhydrous *p*TsOH (0.26 g, 1.5 mmol, 1.5 eq.) were dissolved in dry ethanol (2 mL) and stirred for one hour

at room temperature, followed by the addition of 1-methoxy-4-(prop-1-yn-1-yl)benzene (0.14 g, 1 mmol, 1 eq.). Product (*R*)-**16b** was formed as a colourless oil (187 mg, 90% yield, 85% *ee*).

$[\alpha]_D^{20} = -70$ (c = 0.2, $CHCl_3$).

HPLC data: Diacel CHIRALCEL® OD-H 5 μm, 25 cm (*n*-hexane/*i*PrOH = 99/1, flow rate = 0.5 mL/min, 254 nm). Major isomer t_R = 12.58 min, minor isomer t_R = 13.52 min.

^{1}H NMR (500 MHz, $CDCl_3$) δ = 7.23 (d, *J* = 8.7 Hz, 2H), 6.86 (d, *J* = 8.8 Hz, 2H), 4.17 – 4.05 (m, 2H), 3.79 (s, 3H), 3.65 (q, *J* = 7.2 Hz, 1H), 1.47 (d, *J* = 7.2 Hz, 3H), 1.20 (t, *J* = 7.1 Hz, 3H) ppm.

^{13}C NMR (126 MHz, $CDCl_3$) δ = 174.9, 158.7, 132.9, 128.6, 114.0, 60.8, 55.3, 44.8, 18.8, 14.2 ppm.

HRMS (EC-CI): m/z calcd for $C_{12}H_{16}O_3$: 208.1094 $[M]^+$; found 208.1091.

Data agree with the literature.[26]

Isopropyl (*R*)-2-(4-methoxyphenyl)propanoate (16c):

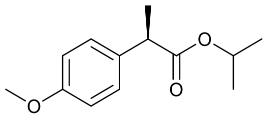

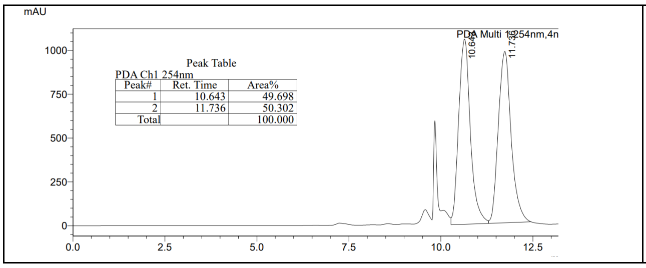

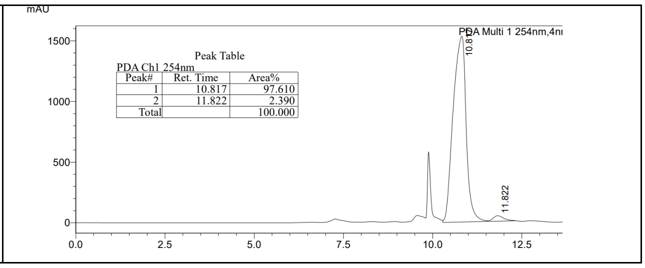

GP5, Method B: (Diacetoxyiodo)benzene (0.48 g, 1.5 mmol, 1.5 eq.) and anhydrous *p*TsOH (0.26 g, 1.5 mmol, 1.5 eq.) were dissolved in 1.5 mL of dry isopropanol and stirred for one hour at room temperature followed by the addition of 1-methoxy-4-(prop-1-yn-1-yl)benzene (0.14 g, 1 mmol, 1 eq.) and BF_3•Et_2O (0.21 g, 0.16 mL, 1.5 mmol, 1.5 eq.). The racemic product was formed as a colourless oil (173 mg, 78% yield).

GP5, Method B: (Diacetoxyiodo)arene **19j** (1.06 g, 1.5 mmol, 1.5 eq.) and anhydrous *p*TsOH (0.26 g, 1.5 mmol, 1.5 eq.) were dissolved in 2 mL of dry isopropanol and stirred for one hour at room temperature followed by the addition of 1-methoxy-4-(prop-1-yn-1-yl)benzene (0.14 g, 1 mmol, 1 eq.) and BF_3•Et_2O (0.21 g, 0.16 mL, 1.5 mmol, 1.5 eq.). The chiral product was formed as a colourless oil (173 mg, 78% yield, 95% *ee*).

$[\alpha]_D^{20} = -34$ (c = 1, $CHCl_3$).

HPLC data: Diacel CHIRALCEL® OD-H 5 μm, 25 cm (*n*-hexane/*i*PrOH = 99.6/0.4, flow rate = 0.5 mL/min, 254 nm). Major isomer t_R = 10.81 min, minor isomer t_R = 11.82 min.

^{1}H NMR (500 MHz, $CDCl_3$) δ = 7.22 (d, *J* = 8.8 Hz, 2H), 6.85 (d, *J* = 8.8 Hz, 2H), 4.98 (hept, *J* = 6.3 Hz, 1H), 3.79 (s, 3H), 3.62 (q, *J* = 7.2 Hz, 1H), 1.45 (d, *J* = 7.2 Hz, 3H), 1.21 (d, *J* = 6.3 Hz, 3H), 1.13 (d, *J* = 6.2 Hz, 3H) ppm.

^{13}C NMR (126 MHz, $CDCl_3$) δ = 174.4, 158.6, 133.0, 128.5, 114.0, 67.9, 55.3, 44.9, 21.8, 21.6, 18.7 ppm.

HRMS (EC-CI): m/z calcd for $C_{13}H_{18}O_3$: 222.1250 $[M]^+$; found 222.1251.

Data agree with the literature.[27]

Propyl-2-(4-methoxyphenyl)propanoate (16d):

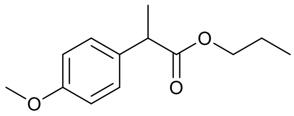

GP5, Method B: (Diacetoxyiodo)benzene (0.48 g, 1.5 mmol, 1.5 eq.) and anhydrous *p*TsOH (0.26 g, 1.5 mmol, 1.5 eq.) were dissolved in 1.5 mL of dry *n*-propanol and stirred for one hour at room temperature followed by the addition of 1-methoxy-4-(prop-1-yn-1-yl)benzene (0.14 g, 1 mmol, 1 eq.) and BF_3•Et_2O (0.21 g, 0.16 mL, 1.5 mmol, 1.5 eq.). The racemic product was formed as a colourless oil (142 mg, 64% yield).

^{1}H NMR (300 MHz, $CDCl_3$) δ = 7.23 (d, *J* = 8.8 Hz, 2H), 6.85 (d, *J* = 8.8 Hz, 2H), 4.01 (t, *J* = 6.7 Hz, 2H), 3.79 (s, 3H), 3.67 (q, *J* = 7.2 Hz, 1H), 1.66 – 1.53 (m, 2H), 1.47 (d, *J* = 7.2 Hz, 3H), 0.86 (t, *J* = 7.4 Hz, 3H) ppm.

^{13}C NMR (75 MHz, $CDCl_3$) δ = 175.0, 158.7, 132.9, 128.6, 114.0, 66.3, 55.3, 44.8, 22.0, 18.7, 10.4 ppm.

IR (neat): *v* = 2554, 2341, 1722, 1509, 1199, 652 cm^{-1}.

Methyl (*R*)-2-(4-methoxy-3-methylphenyl)propanoate (20a):

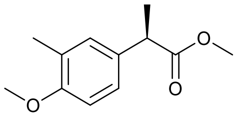

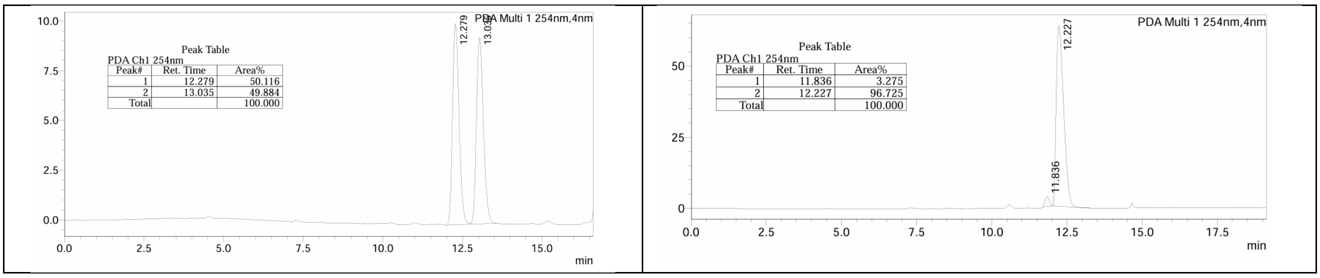

GP5, Method B: (Diacetoxyiodo)benzene (0.48 g, 1.5 mmol, 1.5 eq.) and anhydrous *p*TsOH (0.26 g, 1.5 mmol, 1.5 eq.) were dissolved in 1.5 mL of dry methanol and stirred for one hour at room temperature followed by the addition of 1-methoxy-2-methyl-4-(prop-1-yn-1-yl)benzene (0.16 g, 1 mmol, 1 eq.) and BF_3•Et_2O (0.21 g, 0.16 mL, 1.5 mmol, 1.5 eq.). The racemic product was formed as a colourless oil (177 mg, 85% yield).

GP5, Method B: (Diacetoxyiodo)arene **19j** (1.06 g, 1.5 mmol, 1.5 eq.) and anhydrous *p*TsOH (0.26 g, 1.5 mmol, 1.5 eq.) were dissolved in 2 mL of dry methanol and stirred for one hour at room temperature followed by the addition of 1-methoxy-2-methyl-4-(prop-1-yn-1-yl)benzene (0.16 g, 1 mmol, 1 eq.) and BF_3•Et_2O (0.21 g, 0.16 mL, 1.5 mmol, 1.5 eq.). The chiral product was formed as a colourless oil (177 mg, 85% yield, 94% *ee*).

$[\alpha]_D^{20}$ = −60 (c = 0.4, $CHCl_3$).

HPLC data: YMC chiral amylose-C S-5 μm, (n-hexane/iPrOH = 99.8/0.2, flow rate = 0.7 mL/min, 254 nm). Minor isomer: t_R = 11.83 min, major isomer: t_R = 12.22 min.

^{1}H NMR (300 MHz, $CDCl_3$) δ = 7.13 – 7.04 (m and br, 2H), 6.77 (d, J = 8.0 Hz, 1H), 3.81 (s, 3H), 3.66 (s, 3H), 3.65 (q, J = 7.2 Hz, 1H), 2.21 (s, 3H), 1.47 (d, J = 7.2 Hz, 3H) ppm.

^{13}C NMR (75 MHz, $CDCl_3$) δ = 175.5, 156.9, 132.3, 129.8, 126.9, 125.7, 110.0, 55.4, 52.1, 44.6, 18.8, 16.4 ppm.

IR (neat): ν = 2344, 2341, 1734, 1506, 1247, 669 cm^{-1}.

HRMS (EC-EI): m/z calcd for $C_{12}H_{16}O_3$: 208.1094 [M]$^+$; found 208.1091.

Ethyl (*R*)-2-(4-methoxy-3-methylphenyl)propanoate (20b):

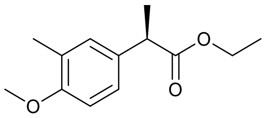

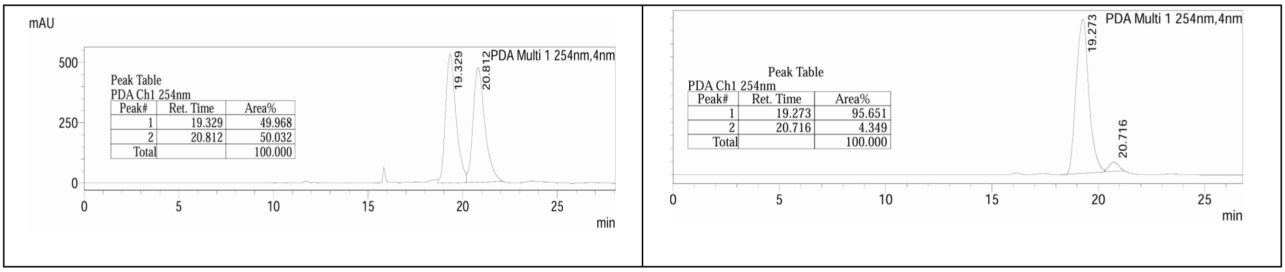

GP5, Method A: (Diacetoxyiodo)benzene (0.48 g, 1.5 mmol, 1.5 eq.) and anhydrous pTsOH (0.26 g, 1.5 mmol, 1.5 eq.) were dissolved in 1.5 mL of dry ethanol and stirred for one hour at room temperature followed by the addition of 1-methoxy-2-methyl-4-(prop-1-yn-1-yl)benzene (0.16 g, 1 mmol, 1 eq.). The racemic product was formed as a colourless oil (182 mg, 82% yield).

GP5, Method A: (Diacetoxyiodo)arene **19j** (1.06 g, 1.5 mmol, 1.5 eq.) and anhydrous pTsOH (0.26 g, 1.5 mmol, 1.5 eq.) were dissolved in 2 mL of dry ethanol and stirred for one hour at room temperature followed by the addition of 1-methoxy-2-methyl-4-(prop-1-yn-1-yl)benzene (0.16 g, 1 mmol, 1 eq.). The chiral product was formed as a colourless oil (182 mg, 82% yield, 91% *ee*).

$[\alpha]_D^{20}$ = −42.11 (c = 0.76, $CHCl_3$).

HPLC data: Diacel CHIRALCEL® OD-H 5 μm, 25 cm (n-hexane/iPrOH = 99.7/0.3, flow rate = 0.3 mL/min, 254 nm). Major isomer t_R = 19.27 min, minor isomer t_R = 20.71 min.

^{1}H NMR (500 MHz, $CDCl_3$) δ = 7.11 – 7.06 (m br, 2H), 6.77 (d, J = 8.2 Hz, 1H), 4.18 – 4.05 (m, 2H), 3.81 (s, 3H), 3.62 (q, J = 7.2 Hz, 1H), 2.21 (s, 3H), 1.46 (d, J = 7.2 Hz, 3H), 1.21 (t, J = 7.1 Hz, 3H) ppm.

^{13}C NMR (126 MHz, $CDCl_3$) δ = 175.0, 156.9, 132.5, 129.8, 126.8, 125.7, 110.0, 60.7, 55.4, 44.8, 18.8, 16.4, 14.2 ppm.

IR (neat): ν = 3327, 2360, 2341, 1504, 1247, 1176, 885 cm^{-1}.

HRMS (EC-EI): m/z calcd for $C_{13}H_{18}O_3$: 222.1250 [M]$^+$; found 222.1248.

Isopropyl (*R*)-2-(4-methoxy-3-methylphenyl)propanoate (20c):

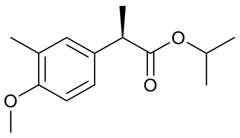

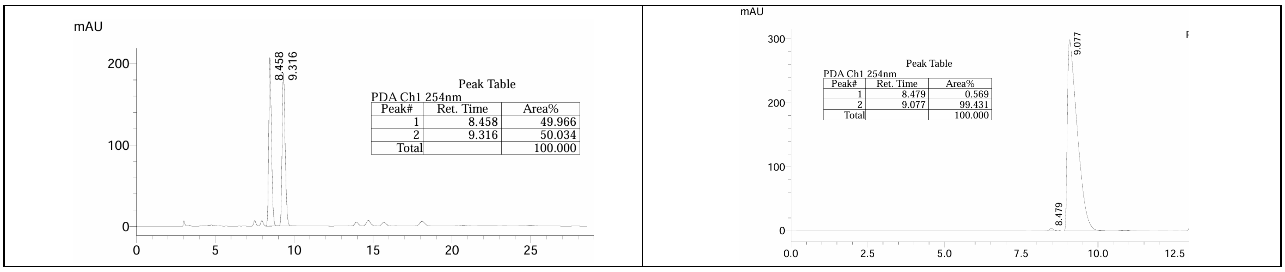

GP5, Method B: (Diacetoxyiodo)benzene (0.48 g, 1.5 mmol, 1.5 eq.) and anhydrous *p*TsOH (0.26 g, 1.5 mmol, 1.5 eq.) were dissolved in 1.5 mL of dry isopropanol and stirred for one hour at room temperature followed by the addition of 1-methoxy-2-methyl-4-(prop-1-yn-1-yl)benzene (0.16 g, 1 mmol, 1 eq.) and BF_3•Et_2O (0.21 g, 0.16 mL, 1.5 mmol, 1.5 eq.). The racemic product was formed as a colourless oil (154 mg, 65% yield).

GP5, Method B: (Diacetoxyiodo)arene **19j** (1.06 g, 1.5 mmol, 1.5 eq.) and anhydrous *p*TsOH (0.26 g, 1.5 mmol, 1.5 eq.) were dissolved in 2 mL of dry isopropanol and stirred for one hour at room temperature followed by the addition of 1-methoxy-2-methyl-4-(prop-1-yn-1-yl)benzene (0.16 g, 1 mmol, 1 eq.) and BF_3•Et_2O (0.21 g, 0.16 mL, 1.5 mmol, 1.5 eq.). The chiral product was formed as a colourless oil (144 mg, 61% yield, 99% *ee*).

$[\alpha]_D^{20} = -26.67$ (c = 0.3, $CHCl_3$).

HPLC data: YMC chiral amylose-C S-5 μm, (*n*-hexane/*i*PrOH = 99.8/0.2, flow rate = 0.7 mL/min, 254 nm). Minor isomer: t_R = 8.47 min, major isomer: t_R = 9.07 min.

^{1}H NMR (300 MHz, $CDCl_3$) δ = 7.12 – 7.05 (m, 2H), 6.76 (d, *J* = 8.6 Hz, 1H), 4.98 (hept, *J* = 6.3 Hz, 1H), 3.81 (s, 3H), 3.58 (q, *J* = 7.2 Hz, 1H), 2.20 (s, 3H), 1.45 (d, *J* = 7.2 Hz, 3H), 1.22 (d, *J* = 6.3 Hz, 3H), 1.14 (d, *J* = 6.2 Hz, 3H) ppm.

^{13}C NMR (101 MHz, $CDCl_3$) δ = 174.6, 156.8, 132.6, 129.8, 126.7, 125.6, 109.9, 67.8, 55.4, 44.9, 21.8, 21.7, 18.8, 16.4 ppm.

IR (neat): ν = 3360, 2358, 2341, 2000, 1726, 1506, 1247, 929 cm^{-1}.

HRMS (ESP-TOF): *m/z* calcd for $C_{14}H_{20}O_3+Na^+$: 259.1310 $[M+Na]^+$; found 259.1308.

Methyl (*R*)-2-(2-methoxyphenyl)propanoate (21a):

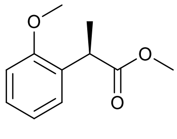

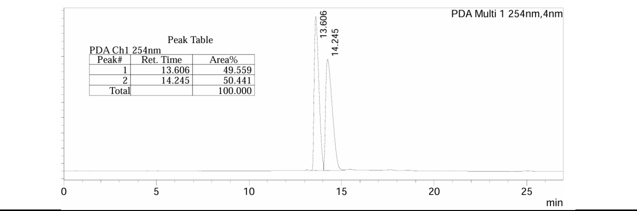

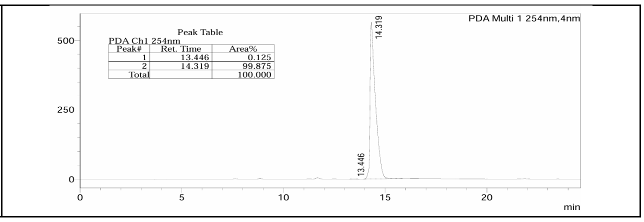

GP5, Method B: (Diacetoxyiodo)benzene (0.48 g, 1.5 mmol, 1.5 eq.) and anhydrous *p*TsOH (0.26 g, 1.5 mmol, 1.5 eq.) were dissolved in 1.5 mL of dry methanol and stirred for one hour at room temperature followed by the addition of 1-methoxy-2-(prop-1-yn-1-yl)benzene (0.14 g, 1 mmol, 1 eq.) and BF_3•Et_2O (0.21 g, 0.16 mL, 1.5 mmol, 1.5 eq.). The racemic product was formed as a colourless oil (173 mg, 89% yield).

GP5, Method B: (Diacetoxyiodo)arene **19j** (1.06 g, 1.5 mmol, 1.5 eq.) and anhydrous *p*TsOH (0.26 g, 1.5 mmol, 1.5 eq.) were dissolved in 2 mL of dry methanol and stirred for one hour at room temperature followed by the addition of 1-methoxy-2-(prop-1-yn-1-yl)benzene (0.16 g, 1 mmol, 1 eq.) and BF_3•Et_2O (0.21 g, 0.16 mL, 1.5 mmol, 1.5 eq.). The chiral product was formed as a colourless oil (173 mg, 89% yield, 99% *ee*).

$[\alpha]_D^{20} = -90$ (c = 0.4, $CHCl_3$).

HPLC data: YMC chiral amylose-C S-5 μm, (*n*-hexane/*i*PrOH = 99.8/0.2, flow rate = 0.7 mL/min, 254 nm). Minor isomer: t_R = 13.44 min, major isomer: t_R = 14.31 min.

^{1}H NMR (500 MHz, $CDCl_3$) δ = 7.22 (t, *J* = 8.0 Hz, 2H), 6.94 (t, *J* = 7.1 Hz, 1H), 6.88 (d, *J* = 8.0 Hz, 1H), 4.06 (q, *J* = 6.4 Hz, 1H), 3.83 (s, 3H), 3.66 (s, 3H), 1.46 (d, *J* = 6.7 Hz, 3H) ppm.

^{13}C NMR (126 MHz, $CDCl_3$) δ = 175.6, 156.7, 129.6, 128.2, 128.0, 120.8, 110.8, 55.6, 52.0, 39.2, 17.5 ppm.

Data agree with the literature.[25]

Ethyl (*R*)-2-(2-methoxyphenyl)propanoate (21b):

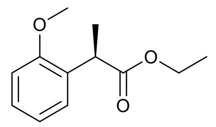

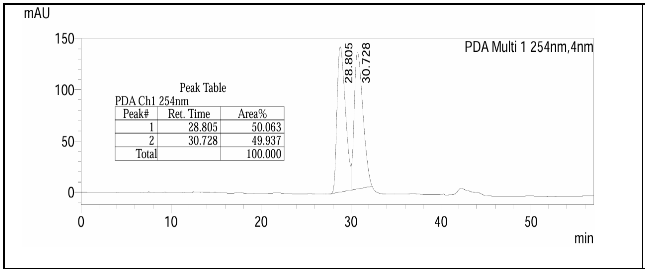

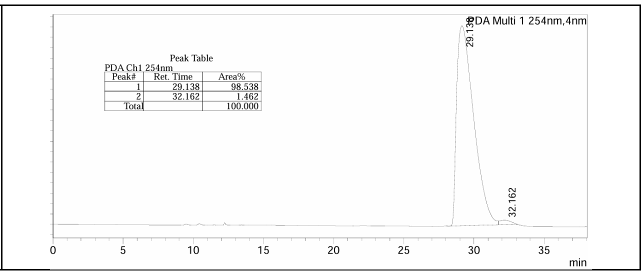

GP5, Method B: (Diacetoxyiodo)benzene (0.48 g, 1.5 mmol, 1.5 eq.) and anhydrous *p*TsOH (0.26 g, 1.5 mmol, 1.5 eq.) were dissolved in 1.5 mL of dry ethanol and stirred for one hour at room temperature followed by the addition of 1-methoxy-2-(prop-1-yn-1-yl)benzene (0.14 g, 1 mmol, 1 eq.) and BF_3•Et_2O (0.21 g, 0.16 mL, 1.5 mmol, 1.5 eq.). The racemic product was formed as a colourless oil (176 mg, 84% yield).

GP5, Method B: (Diacetoxyiodo)arene **19j** (1.06 g, 1.5 mmol, 1.5 eq.) and anhydrous *p*TsOH (0.26 g, 1.5 mmol, 1.5 eq.) were dissolved in 2 mL of dry ethanol and stirred for one hour at room temperature followed by the addition of 1-methoxy-2-(prop-1-yn-1-yl)benzene (0.16 g, 1 mmol, 1 eq.) and BF_3•Et_2O (0.21 g, 0.16 mL, 1.5 mmol, 1.5 eq.). The chiral product was formed as a colourless oil (175 mg, 84% yield, 97% *ee*).

$[\alpha]_D^{20} = -56.67$ (c = 1.2, $CHCl_3$).

HPLC data: CHIRALPAK® IC, 5 μm, 25 cm (*n*-hexane/*i*PrOH = 99.8/0.2, flow rate = 0.7 mL/min, 254 nm). Major isomer: t_R = 29.13 min, minor isomer: t_R = 32.16 min.

^{1}H NMR (500 MHz, $CDCl_3$) δ = 7.25 – 7.20 (m, 2H), 6.94 (td, *J* = 7.5, 1.1 Hz, 1H), 6.87 (d, *J* = 8.1 Hz, 1H), 4.14 (q, *J* = 7.1 Hz, 2H), 4.03 (q, *J* = 7.2 Hz, 1H), 3.82 (s, 3H), 1.45 (d, *J* = 7.2 Hz, 3H), 1.20 (t, *J* = 7.1 Hz, 3H) ppm.

^{13}C NMR (126 MHz, $CDCl_3$) δ = 175.2, 156.8, 129.8, 128.1, 128.0, 120.8, 110.7, 60.5, 55.5, 39.4, 17.4, 14.3 ppm.

Data agree with the literature.[28]

Isopropyl (*R*)-2-(2-methoxyphenyl)propanoate (21c):

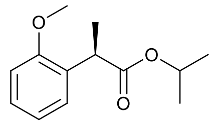

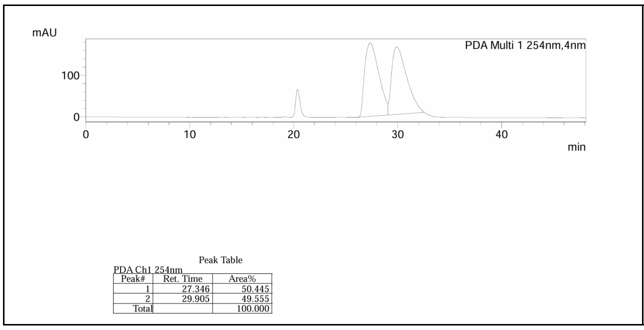

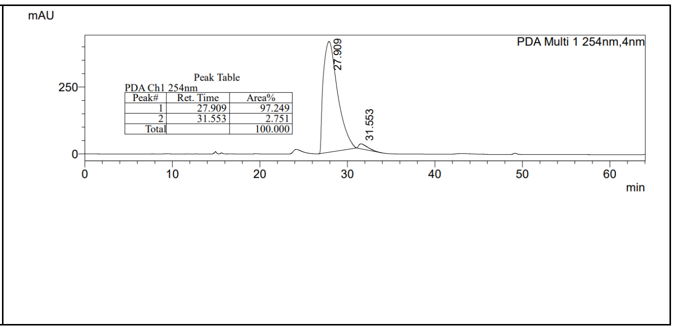

GP5, Method B: (Diacetoxyiodo)benzene (0.48 g, 1.5 mmol, 1.5 eq.) and anhydrous *p*TsOH (0.26 g, 1.5 mmol, 1.5 eq.) were dissolved in 1.5 mL of isopropanol and stirred for one hour at room temperature followed by the addition of 1-methoxy-2-(prop-1-yn-1-yl)benzene (0.14 g, 1 mmol, 1 eq.) and BF_3•Et_2O (0.21 g, 0.16 mL, 1.5 mmol, 1.5 eq.). The racemic product was formed as a colourless oil (142 mg, 64% yield).

GP5, Method B: (Diacetoxyiodo)arene **19j** (1.06 g, 1.5 mmol, 1.5 eq.) and anhydrous *p*TsOH (0.26 g, 1.5 mmol, 1.5 eq.) were dissolved in 2 mL of isopropanol and stirred for one hour at room temperature followed by the addition of 1-methoxy-2-(prop-1-yn-1-yl)benzene (0.16 g, 1 mmol, 1 eq.) and BF_3•Et_2O (0.21 g, 0.16 mL, 1.5 mmol, 1.5 eq.). The chiral product was formed as a colourless oil (150 mg, 67% yield, 94% *ee*).

$[\alpha]_D^{20} = -33.33$ (c = 0.6, $CHCl_3$).

HPLC data: CHIRALPAK® IC, 5 μm, 25 cm (*n*-hexane/*i*PrOH = 99.8/0.2, flow rate = 0.5 mL/min, 254 nm). Major isomer: t_R = 27.90 min, minor isomer: t_R = 31.55 min.

^{1}H NMR (500 MHz, $CDCl_3$) δ = 7.21 (d, *J* = 8.0 Hz, 2H), 6.93 (t, *J* = 7.2 Hz, 1H), 6.86 (d, *J* = 8.0 Hz, 1H), 5.08 – 4.98 (m, 1H), 3.98 (q, *J* = 7.0 Hz, 1H), 3.81 (s, 3H), 1.44 (d, *J* = 7.1 Hz, 3H), 1.21 (d, *J* = 6 Hz, 3H), 1.16 (d, *J* = 5.7 Hz, 3H) ppm.

^{13}C NMR (126 MHz, $CDCl_3$) δ = 174.7, 156.8, 129.9, 128.0, 120.7, 110.6, 67.6, 55.4, 39.7, 21.8, 21.7, 17.3 ppm.

IR (neat): ν = 3381, 2970, 1726, 1494, 1244, 1107, 950, 750 cm^{-1}.

HRMS (EC-CI): *m*/*z* calcd for $C_{13}H_{18}O_3$: 222.1251 $[M]^+$; found 222.1250.

Methyl (*R*)-2-(4-methoxyphenyl)butanoate (22a):

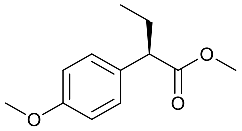

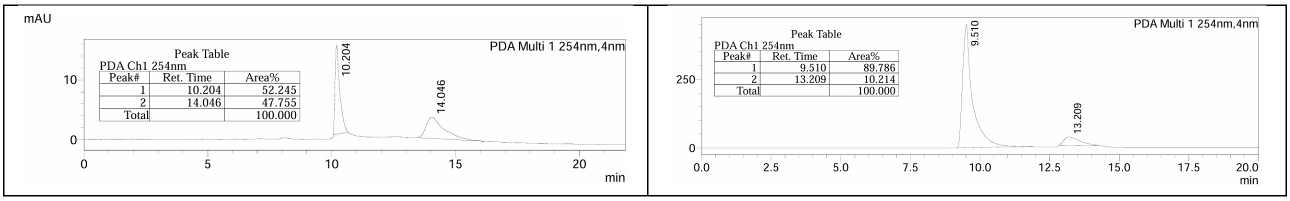

GP5, Method B: (Diacetoxyiodo)benzene (0.48 g, 1.5 mmol, 1.5 eq.) and anhydrous *p*TsOH (0.26 g, 1.5 mmol, 1.5 eq.) were dissolved in 1.5 mL of dry methanol and stirred for one hour at room temperature followed by the addition of 1-(but-1-yn-1-yl)-4-methoxybenzene (0.16 g, 1 mmol, 1 eq.) and BF_3•Et_2O (0.21 g, 0.16 mL, 1.5 mmol, 1.5 eq.). The racemic product was formed as a colourless oil (187 mg, 90% yield).

GP5, Method B: (Diacetoxyiodo)arene **19j** (1.06 g, 1.5 mmol, 1.5 eq.) and anhydrous *p*TsOH (0.26 g, 1.5 mmol, 1.5 eq.) were dissolved in 2 mL of dry methanol and stirred for one hour at room temperature followed by the addition of 1-(but-1-yn-1-yl)-4-methoxybenzene (0.16 g, 1 mmol, 1 eq.) and BF_3•Et_2O (0.21 g, 0.16 mL, 1.5 mmol, 1.5 eq.). The chiral product was formed as a colourless oil (181 mg, 87% yield, 80% *ee*).

$[\alpha]_D^{20} = -53.33$ (c = 0.3, $CHCl_3$).

HPLC data: Diacel CHIRALCEL® OD-H 5 μm, 25 cm (*n*-hexane/*i*PrOH = 99.8/0.2, flow rate = 0.7 mL/min, 254 nm). Major isomer t_R = 9.51 min, minor isomer t_R = 13.20 min.

^{1}H NMR (400 MHz, $CDCl_3$) δ = 7.22 (d, *J* = 8.6 Hz, 2H), 6.85 (d, *J* = 8.8 Hz, 2H), 3.79 (s, 3H), 3.65 (s, 3H), 3.40 (t, *J* = 7.7 Hz, 1H), 2.13 – 2.00 (m, 1H), 1.83 – 1.70 (m, 1H), 0.88 (t, *J* = 7.4 Hz, 3H) ppm.

^{13}C NMR (101 MHz, $CDCl_3$) δ = 174.9, 158.8, 131.3, 129.0, 114.0, 55.3, 52.6, 52.0, 26.9, 12.2 ppm.

Data agree with the literature.[29]

Ethyl (*R*)-2-(4-methoxyphenyl)butanoate (22b):

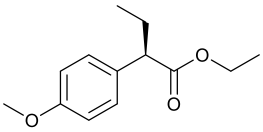

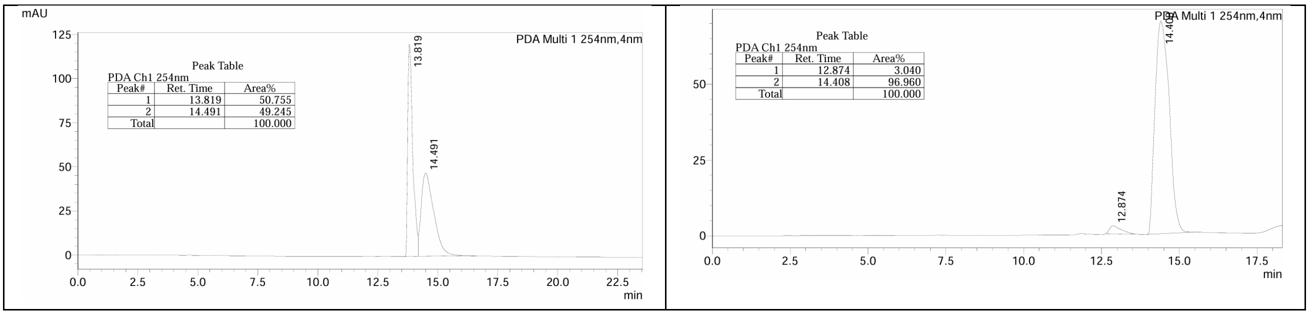

GP5, Method A: (Diacetoxyiodo)benzene (0.48 g, 1.5 mmol, 1.5 eq.) and anhydrous *p*TsOH (0.26 g, 1.5 mmol, 1.5 eq.) were dissolved in 1.5 mL of dry ethanol and stirred for one hour at room temperature followed by the addition of 1-(but-1-yn-1-yl)-4-methoxybenzene (0.16 g, 1 mmol, 1 eq.). The racemic product was formed as a colourless oil (202 mg, 91% yield).

GP5, Method A: (Diacetoxyiodo)arene **19j** (1.06 g, 1.5 mmol, 1.5 eq.) and anhydrous *p*TsOH (0.26 g, 1.5 mmol, 1.5 eq.) were dissolved in 2 mL of dry ethanol and stirred for one hour at room temperature followed by the addition of 1-(but-1-yn-1-yl)-4-methoxybenzene (0.16 g, 1 mmol, 1 eq.). The chiral product was formed as a colourless oil (202 mg, 91% yield, 94% *ee*).

$[\alpha]_D^{20} = -38.50$ (c = 0.26, $CHCl_3$).

HPLC data: Diacel CHIRALCEL® OD-H 5 μm, 25 cm (*n*-hexane/*i*PrOH = 99.8/0.2, flow rate = 0.7 mL/min, 254 nm). Minor isomer t_R = 12.87 min, major isomer t_R = 14.40 min.

^{1}H NMR (400 MHz, $CDCl_3$) δ = 7.23 (d, *J* = 8.7 Hz, 2H), 6.85 (d, *J* = 8.8 Hz, 2H), 4.19 – 4.03 (m, 2H), 3.79 (s, 3H), 3.38 (t, *J* = 7.7 Hz, 1H), 2.12 – 2.00 (m, 1H), 1.81 – 1.70 (m, 1H), 1.21 (t, *J* = 7.1 Hz, 3H), 0.88 (t, *J* = 7.4 Hz, 3H) ppm.

^{13}C NMR (101 MHz, $CDCl_3$) δ = 174.4, 158.7, 131.4, 129.0, 113.9, 60.6, 55.3, 52.7, 26.9, 14.3, 12.2 ppm.

Data agree with the literature.[30]

Isopropyl (*R*)-2-(4-methoxyphenyl)butanoate (22c):

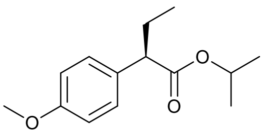

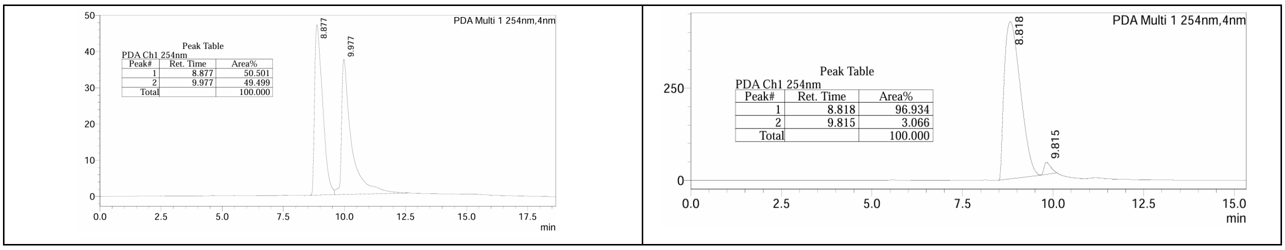

GP5, Method B: (Diacetoxyiodo)benzene (0.48 g, 1.5 mmol, 1.5 eq.) and anhydrous *p*TsOH (0.26 g, 1.5 mmol, 1.5 eq.) were dissolved in 1.5 mL of dry isopropanol and stirred for one hour at room temperature followed by the addition of 1-(but-1-yn-1-yl)-4-methoxybenzene (0.16 g, 1 mmol, 1 eq.) and BF_3•Et_2O (0.21 g, 0.16 mL, 1.5 mmol, 1.5 eq.). The racemic product was formed as a colourless oil (136 mg, 69% yield).

GP5, Method B: (Diacetoxyiodo)arene **19j** (1.06 g, 1.5 mmol, 1.5 eq.) and anhydrous *p*TsOH (0.26 g, 1.5 mmol, 1.5 eq.) were dissolved in 2 mL of dry isopropanol and stirred for one hour at room temperature followed by the addition of 1-(but-1-yn-1-yl)-4-methoxybenzene (0.16 g, 1 mmol, 1 eq.) and BF_3•Et_2O (0.21 g, 0.16 mL, 1.5 mmol, 1.5 eq.). The chiral product was formed as a colourless oil (173 mg, 73% yield, 94% *ee*).

$[\alpha]_D^{20} = -90$ (c = 0.2, $CHCl_3$).

HPLC data: Diacel CHIRALCEL® OD-H 5 μm, 25 cm (*n*-hexane/*i*PrOH = 99.8/0.2, flow rate = 0.7 mL/min, 254 nm). Major isomer t_R 8.81 min, minor isomer t_R 9.81 min.

^{1}H NMR (300 MHz, $CDCl_3$) δ = 7.23 (d, J = 8.6 Hz, 2H), 6.84 (d, J = 8.8 Hz, 2H), 4.99 (hept, J = 6.3 Hz, 1H), 3.78 (s, 3H), 3.35 (t, J = 7.7 Hz, 1H), 2.13 – 1.98 (m, 1H), 1.82 – 1.66 (m, 1H), 1.18 (dd, J = 24.4, 6.3 Hz, 6H), 0.88 (t, J = 7.4 Hz, 3H) ppm.

^{13}C NMR (101 MHz, $CDCl_3$) δ = 173.9, 158.7, 131.6, 129.0, 113.9, 67.8, 55.3, 53.0, 26.9, 21.9, 21.7, 12.2 ppm.

Data agree with the literature.[31]

Methyl (*R*)-2-(2,4-dimethoxyphenyl)propanoate (23a):

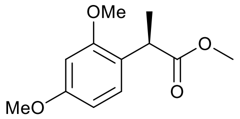

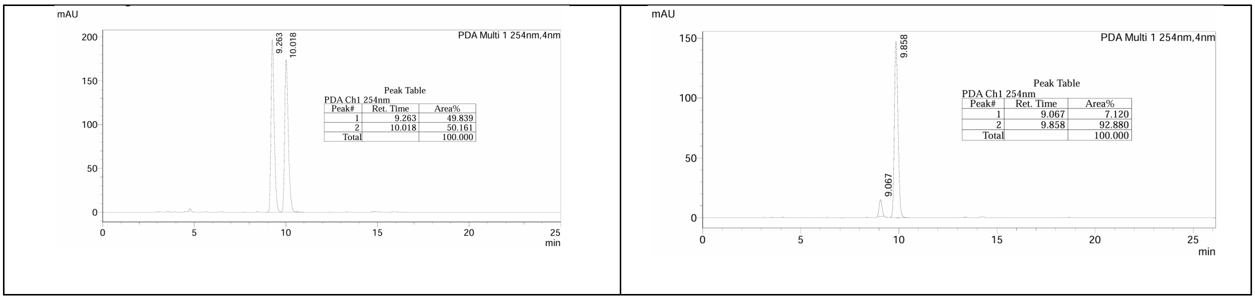

GP5, Method C: (Diacetoxyiodo)benzene (0.48 g, 1.5 mmol, 1.5 eq.) and anhydrous *p*TsOH (0.26 g, 1.5 mmol, 1.5 eq.) were dissolved in 1.5 mL of dry methanol and stirred for one hour at room temperature followed by the addition of 2,4-dimethoxy-1-(prop-1-yn-1-yl)benzene (0.17 g, 1 mmol, 1 eq.). The racemic product was formed as a colourless oil (184 mg, 82% yield).

GP5, Method C: (Diacetoxyiodo)arene **19j** (1.06 g, 1.5 mmol, 1.5 eq.) and anhydrous *p*TsOH (0.26 g, 1.5 mmol, 1.5 eq.) were dissolved in 2 mL of dry methanol and stirred for one hour at room temperature followed by the addition of 2,4-dimethoxy-1-(prop-1-yn-1-yl)benzene (0.17 g, 1 mmol, 1 eq.). The chiral product was formed as a colourless oil (180 mg, 80% yield, 86% *ee*).

$[\alpha]_D^{20}$ = −53.85 (c = 0.26, $CHCl_3$).

HPLC data: YMC chiral amylose-C S-5 μm, (*n*-hexane/*i*PrOH = 98/2, flow rate = 1.0 mL/min, 254 nm). Minor isomer t_R 9.06 min, major isomer t_R 9.85 min.

^{1}H NMR (300 MHz, $CDCl_3$) δ = 7.11 (d, J = 8.8 Hz, 1H), 6.50 – 6.43 (m, 2H), 3.97 (q, J = 7.2 Hz, 1H), 3.80 (s-overlapped, 6H), 3.65 (s, 3H), 1.42 (d, J = 7.2 Hz, 3H) ppm.

^{13}C NMR (126 MHz, $CDCl_3$) δ = 175.9, 159.9, 157.7, 128.4, 122.1, 104.4, 98.8, 55.6, 55.4, 52.0, 38.6, 17.6 ppm.

IR (neat): v = 2981, 2949, 2358, 1750, 1610, 1506, 1205, 1033, 833 cm^{-1}.

HRMS (EC-EI): m/z calcd for $C_{12}H_{16}O_4$: 224.1043 $[M]^+$; found 224.1036.

Ethyl (*R*)-2-(2,4-dimethoxyphenyl)propanoate (23b):

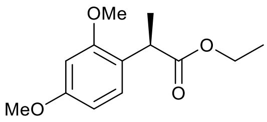

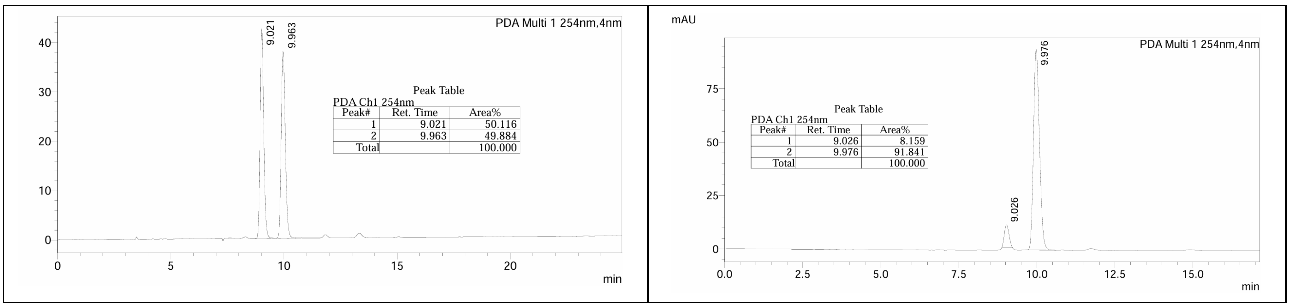

GP5, Method C: (Diacetoxyiodo)benzene (0.48 g, 1.5 mmol, 1.5 eq.) and anhydrous *p*TsOH (0.26 g, 1.5 mmol, 1.5 eq.) were dissolved in 1.5 mL of dry ethanol and stirred for one hour at room temperature followed by the addition of 2,4-dimethoxy-1-(prop-1-yn-1-yl)benzene (0.17 g, 1 mmol, 1 eq.). The racemic product was formed as a colourless oil (208 mg, 87% yield).

GP5, Method C: (Diacetoxyiodo)arene **19j** (1.06 g, 1.5 mmol, 1.5 eq.) and anhydrous *p*TsOH (0.26 g, 1.5 mmol, 1.5 eq.) were dissolved in 2 mL of dry ethanol and stirred for one hour at room temperature followed by the addition of 2,4-dimethoxy-1-(prop-1-yn-1-yl)benzene (0.17 g, 1 mmol, 1 eq.). The chiral product was formed as a colourless oil (190 mg, 80% yield, 84% *ee*).

$[\alpha]_D^{20} = -28.0$ (c = 1.0, $CHCl_3$).

HPLC data: YMC chiral amylose-C S-5 μm, (*n*-hexane/*i*PrOH = 98/2, flow rate = 1.0 mL/min, 254 nm). Minor isomer t_R 9.02 min, major isomer t_R 9.97 min.

^{1}H NMR (400 MHz, $CDCl_3$) δ = 7.12 (d, *J* = 8.0 Hz, 1H), 6.49 – 6.41 (m, 2H), 4.13 (q, *J* = 7.1 Hz, 2H), 3.94 (q, *J* = 7.2 Hz, 1H), 3.79 (s-overlapped, 6 H), 1.42 (d, *J* = 7.2 Hz, 3H), 1.20 (t, *J* = 7.1 Hz, 3H) ppm.

^{13}C NMR (101 MHz, $CDCl_3$) δ = 175.4, 159.8, 157.7, 128.3, 122.3, 104.2, 98.7, 60.5, 55.5, 55.4, 38.8, 17.6, 14.3 ppm.

Data agree with the literature.[27]

Isopropyl (*R*)-2-(2,4-dimethoxyphenyl)propanoate (23c):

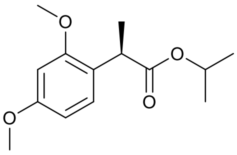

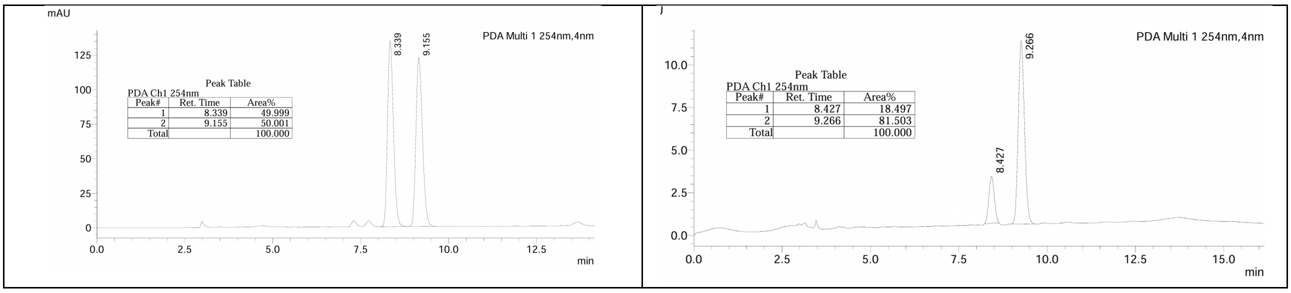

GP5, Method C: (Diacetoxyiodo)benzene (0.48 g, 1.5 mmol, 1.5 eq.) and anhydrous *p*TsOH (0.26 g, 1.5 mmol, 1.5 eq.) were dissolved in 1.5 mL of dry isopropanol and stirred for one hour at room temperature followed by the addition of 2,4-dimethoxy-1-(prop-1-yn-1-yl)benzene (0.17 g, 1 mmol, 1 eq.). The racemic product was formed as a colourless oil (141 mg, 56% yield).

GP5, Method C: (Diacetoxyiodo)arene **19j** (1.06 g, 1.5 mmol, 1.5 eq.) and anhydrous *p*TsOH (0.26 g, 1.5 mmol, 1.5 eq.) were dissolved in 2 mL of dry isopropanol and stirred for one hour at room temperature followed by the addition of 2,4-dimethoxy-1-(prop-1-yn-1-yl)benzene (0.17 g, 1 mmol, 1 eq.). The chiral product was formed as a colourless oil (152 mg, 60% yield, 63% *ee*).

$[\alpha]_D^{20} = -40.0$ (c = 0.4, $CHCl_3$).

HPLC data: YMC chiral amylose-C S-5 μm, (*n*-hexane/*i*PrOH = 98/2, flow rate = 1.0 mL/min, 254 nm). Minor isomer t_R 8.42 min, major isomer t_R 9.26 min.

^{1}H NMR (400 MHz, $CDCl_3$) δ = 7.11 (d, *J* = 7.9 Hz, 1H), 6.48 – 6.41 (m, 2H), 5.01 (hept, *J* = 6.3 Hz, 1H), 3.90 (q, *J* = 7.2 Hz, 1H), 3.79 (s, 3H), 3.78 (s, 3H), 1.41 (d, *J* = 7.2 Hz, 3H), 1.20 (d, *J* = 6.3 Hz, 3H), 1.16 (d, *J* = 6.2 Hz, 3H) ppm.

^{13}C NMR (101 MHz, $CDCl_3$) δ = 175.0, 159.7, 157.7, 128.2, 122.4, 104.1, 98.6, 67.5, 55.4, 39.0, 21.8, 21.7, 17.4 ppm.

IR (neat): *v* = 2978, 2935, 1724, 1612, 1587, 1506, 1200, 1100, 1035 cm^{-1}.

HRMS (EC-CI): *m*/*z* calcd for $C_{14}H_{20}O_4$: 252.1356 $[M]^+$; found 252.1358.

Methyl (*R*)-2-(2,4-dimethoxy-3-methylphenyl)propanoate (24a):

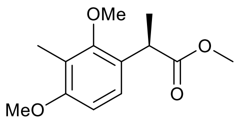

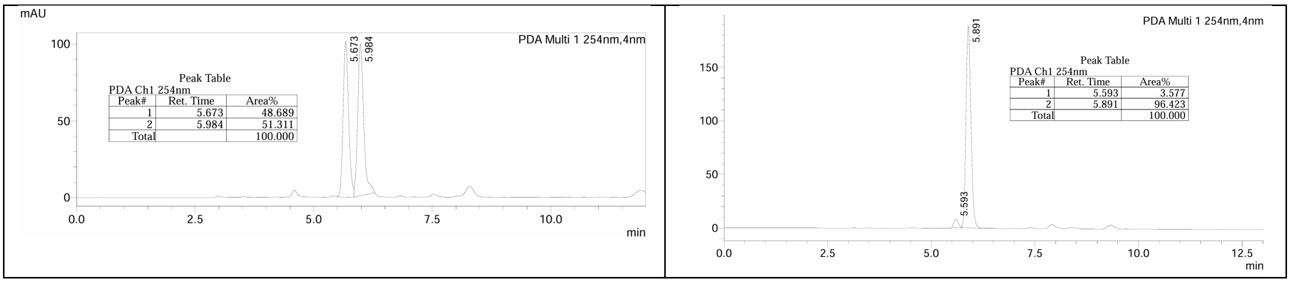

GP5, Method C: (Diacetoxyiodo)benzene (0.48 g, 1.5 mmol, 1.5 eq.) and anhydrous *p*TsOH (0.26 g, 1.5 mmol, 1.5 eq.) were dissolved in 1.5 mL of dry methanol and stirred for one hour at room temperature followed by the addition of 1,3-dimethoxy-2-methyl-4-(prop-1-yn-1-yl)benzene (0.17 g, 1 mmol, 1 eq.). The racemic product was formed as a colourless oil (136 mg, 57% yield).

GP5, Method C: (Diacetoxyiodo)arene **19j** (1.06 g, 1.5 mmol, 1.5 eq.) and anhydrous *p*TsOH (0.26 g, 1.5 mmol, 1.5 eq.) were dissolved in 2 mL of dry methaonl and stirred for one hour at room temperature followed by the addition of 1,3-dimethoxy-2-methyl-4-(prop-1-yn-1-yl)benzene (0.17 g, 1 mmol, 1 eq.). The chiral product was formed as a colourless oil (180 mg, 76% yield, 93% *ee*).

$[\alpha]_D^{20} = -71.42$ (c = 0.28, $CHCl_3$).

HPLC data: YMC chiral amylose-C S-5 μm, (*n*-hexane/*i*PrOH = 98/2, flow rate = 1.0 mL/min, 254 nm). Minor isomer t_R 5.59 min, major isomer t_R 5.89 min.

^{1}H NMR (300 MHz, $CDCl_3$) δ = 7.09 (d, *J* = 8.6 Hz, 1H), 6.64 (d, *J* = 8.6 Hz, 1H), 4.07 (q, *J* = 7.2 Hz, 1H), 3.81 (s, 3H), 3.75 (s, 3H), 3.66 (s, 3H), 2.17 (s, 3H), 1.44 (d, *J* = 7.2 Hz, 3H) ppm.

^{13}C NMR (126 MHz, $CDCl_3$) δ = 175.8, 157.9, 156.7, 126.3, 125.1, 119.7, 106.4, 61.1, 55.7, 52.0, 38.1, 18.7, 9.5 ppm.

IR (neat): *ν* = 2939, 2360, 2341, 1734, 1487, 1107 cm^{-1}.

HRMS (ESP-TOF): *m*/*z* calcd for $C_{13}H_{18}O_4$+Na^+: 261.1103 [M+Na]$^+$; found 261.1108.

Ethyl (*R*)-2-(2,4-dimethoxy-3-methylphenyl)propanoate (24b):

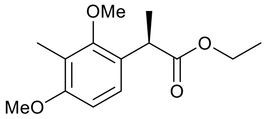

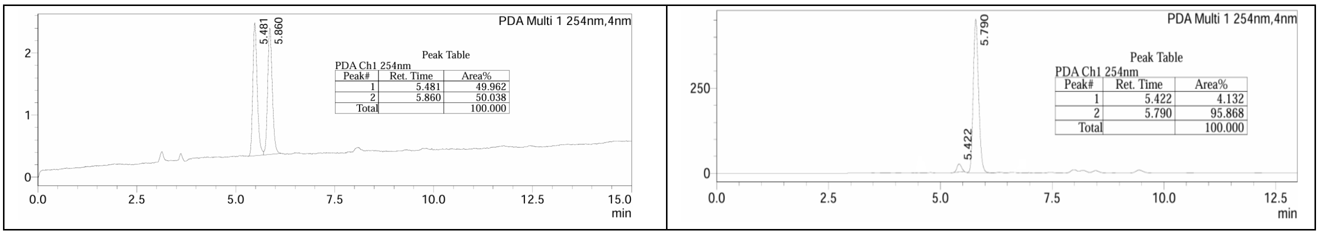

GP5, Method C: (Diacetoxyiodo)benzene (0.48 g, 1.5 mmol, 1.5 eq.) and anhydrous *p*TsOH (0.26 g, 1.5 mmol, 1.5 eq.) were dissolved in 1.5 mL of dry ethanol and stirred for one hour at room temperature followed by the addition of 1,3-dimethoxy-2-methyl-4-(prop-1-yn-1-yl)benzene (0.17 g, 1 mmol, 1 eq.). The racemic product was formed as a colourless oil (151 mg, 60% yield).

GP5, Method C: (Diacetoxyiodo)arene **19j** (1.06 g, 1.5 mmol, 1.5 eq.) and anhydrous *p*TsOH (0.26 g, 1.5 mmol, 1.5 eq.) were dissolved in 2 mL of dry ethanol and stirred for one hour at room temperature followed by the addition of 1,3-dimethoxy-2-methyl-4-(prop-1-yn-1-yl)benzene (0.17 g, 1 mmol, 1 eq.). The chiral product was formed as a colourless oil (212 mg, 84% yield, 92% *ee*). HPLC data: YMC chiral amylose-C S-5 μm, (*n*-hexane/*i*PrOH = 98/2, flow rate = 1.0 mL/min, 254 nm). Minor isomer t_R 5.42 min, major isomer t_R 5.79 min.

$[\alpha]_D^{20}$ = −25.0 (c = 0.8, $CHCl_3$).

^{1}H NMR (500 MHz, $CDCl_3$) δ = 7.11 (d, *J* = 8.6 Hz, 1H), 6.64 (d, *J* = 8.6 Hz, 1H), 4.19 – 4.03 (m, 3H), 3.81 (s, 3H), 3.75 (s, 3H), 2.17 (s, 3H), 1.44 (d, *J* = 7.2 Hz, 3H), 1.20 (t, *J* = 7.1 Hz, 3H) ppm.

^{13}C NMR (126 MHz, $CDCl_3$) δ = 175.3, 157.8, 156.7, 126.4, 125.0, 119.6, 106.4, 61.14, 60.6, 55.6, 38.1, 18.8, 14.2, 9.4 ppm.

IR (neat): *ν* = 2980, 2937, 1728, 1600, 1487, 1105, 796 cm^{-1}.

HRMS (ESP-TOF): *m*/*z* calcd for $C_{14}H_{20}O_4$+Na^+: 275.1259 [M+Na]$^+$; found 275.1264.

Ethyl 2-(6-methoxynaphthalen-2-yl)propanoate (26)

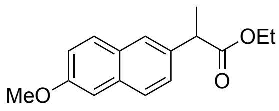

In 10 mL round bottom flask and under nitrogen atmosphere, (diacetoxyiodo)benzene (0.24 g, 0.75 mmol, 1.5 equiv.) and anhyd. *p*TsOH (0.13 g, 0.75 mmol, 1.5 equiv.) were dissolved in 1 mL of dry ethanol and stirred for 1 h at room temperature followed by the addition of compound **25** (100 mg, 0.5 mmol, 1 equiv.). The reaction mixture was stirred at room temperature for 20 h. Then, the solvent was removed under reduced pressure. The residue was dissolved again with ethyl acetate and washed with sat. aq. $NaHCO_3$ (10 mL) solution and sat. aq. $Na_2S_2O_3$ solution (10 mL) and extracted with ethyl acetate (3 × 20 mL). The combined organic layers were dried over $MgSO_4$, filtered, and concentrated under reduced pressure. The crude product was purified by flash chromatography on silica gel (cyclohexane/ethyl acetate = 95:5). A colourless solid was obtained (48 mg, yield: 48%).

^{1}H NMR (300 MHz, $CDCl_3$) δ = 7.68 (dd, *J* = 10.8, 4.9 Hz, 3H), 7.41 (dd, *J* = 8.5, 1.9 Hz, 1H), 7.16 – 7.10 (m, 2H), 4.19 – 4.05 (m, 2H), 3.91 (s, 3H), 3.84 (q, *J* = 7.1 Hz, 1H), 1.57 (d, *J* = 7.2 Hz, 3H), 1.20 (t, *J* = 7.1 Hz, 3H) ppm.

^{13}C NMR (126 MHz, $CDCl_3$) δ = 174.9, 157.8, 136.0, 133.8, 129.4, 129.1, 127.2, 126.4, 126.1, 119.1, 105.7, 60.9, 55.5, 45.6, 18.8, 14.3 ppm.

GC-MS (m/z): 258.1256 [M].

(*S*)-Ethyl 2-(6-methoxynaphthalen-2-yl)propanoate [(*S*)-26]

(Diacetoxyiodo)arene *ent*-**19i** (3.90 g, 5.54 mmol) and anhyd. *p*TsOH (0.95 g, 5.54 mmol, 1.5 eq.) were dissolved in 28 mL of dry ethanol and stirred for 1 h at room temperature followed by the addition of of compound **25** (0.72 g, 3.69 mmol, 1 eq.). The reaction mixture was stirred at 40 ºC for 20 h. The crude product was purified by flash chromatography on silica gel (cyclohexane/ethyl acetate = 95:5). A colourless solid was obtained (399 mg, 1.55 mmol, yield: 42%). 303 mg of compound **25** (40%, 1.55 mmol) and iodine(I) compound *ent*-**S12** (2.7 g, 4.59 mmol) were recovered.

$[\alpha]_D^{20}$ = +39.5 (c = 0.12, $CHCl_3$).

HPLC data: Chiralcel OD-H (250 mm × 4.6 mm, 5 μm), *n*-hexane/*i*PrOH = 99.2:0.8, flow rate = 0.7 mL/min, room temperature, detection at 254 nm. 88% *ee.*

Minor isomer (*R*)-**26** t_R = 11.1 min, major isomer (*S*)-**26** t_R = 12.9 min

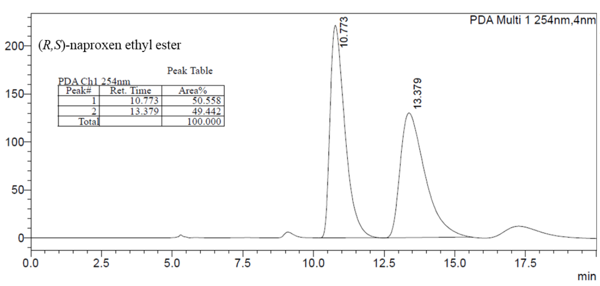

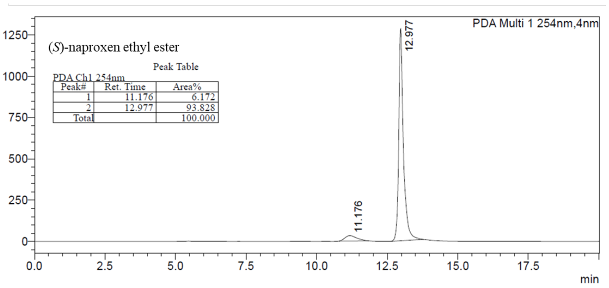

(*S*)-2-(6-Methoxynaphthalen-2-yl)propanoic acid, naproxen, (*S*)-2

(*S*)-Ethyl 2-(6-methoxynaphthalen-2-yl)propanoate (*S*)-**26** (50 mg, 0.19 mmol, 1 eq.) was dissolved in THF (2 mL) and H_2O (2 mL) under nitrogen atmosphere. The solution was further charged with aq. HCl solution (2 M, 0.2 mL) , and the resulting solution was stirred at 50 ºC for 24 h. The reaction mixture was then diluted with 2 mL water and extracted with EtOAc (3 × 2 mL). The combined organic layers were dried over

anhydrous $MgSO_4$ and concentrated under reduced pressure. The crude product was purified by flash chromatography on silica gel (n-hexane/ethyl acetate = 3:1) to afford (*S*)-**2** as a colourless solid (32 mg, 0.14 mmol, 72%).

^{1}H NMR (400 MHz, $CDCl_3$) δ = 7.76 – 7.69 (m, 3H), 7.44 (dd, *J* = 8.5, 1.7 Hz, 1H), 7.19 – 7.10 (m, 2H), 3.93 (s, 3H), 3.93 – 3.86 (m, 1H), 1.62 (t, *J* = 5.7 Hz, 3H) ppm.

^{13}C NMR (75 MHz, $CDCl_3$) δ = 180.5, 157.7, 134.8, 133.8, 129.3, 128.8, 127.2, 126.2, 126.1, 119.0, 105.5, 55.3, 45.2, 18.1 ppm.

$[\alpha]_D^{20}$ = +56.0 (c = 0.12, $CHCl_3$). Literature reference: $[\alpha]_D^{20}$ = +65.0 (c = 1.00, $CHCl_3$).[32]

As we could not reliably measure the *ee* of naproxen by HPLC, 20 mg of (*S*)-**2** was esterified again (EtOH, H^+) showing an almost identical *ee* of 87.6%.

HPLC of re-esterified (EtOH) naproxen (*S*)-**2**:

Minor isomer (*R*)-**26** t_R = 11.8 min, major isomer (*S*)-**26** t_R = 14.2 min

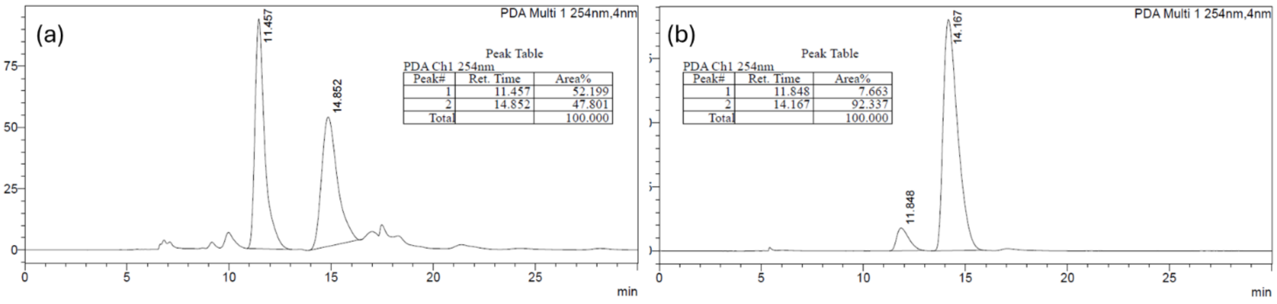

Computational Details

Density functional theory (DFT) calculations were carried out with the ORCA quantum chemistry program, version 6.1.0.[33,34,35,36] The Becke, 3-parameter, Lee-Young-Parr (B3LYP) functional was used to approximate exchange and correlation energy contributions to the total energy,[37,38,39,40] alongside Grimme's D4 dispersion correction (B3LYP-D4).[41,42,43] Geometry optimization and frequency calculations were performed with the def2-SVP basis set for all atoms. The electronic energy was refined with a def2-TZVP basis set on an otherwise identical level of theory. The Stuttgart-Dresden effective core potential as implemented in def2-ECP, was used for iodine in all calculations.[44,45,46,47,48]

Geometry optimizations were performed in gas phase, the solvation energy was approximated by a single point calculation on the same level of theory, alongside the conductor-like polarizable continuum model (C-PCM), using the dielectric constant of ethanol ($\varepsilon = 24.5$).[49,50,51] To account for the conversion of concentrations in gas and solution phase, 1.89 kcal mol^{-1} were added to each species.[52]

For geometry optimizations, tight optimization criteria were used. For single point calculations, solvation single point calculations, frequency calculations and geometry optimizations, tight self consistent field convergence criteria were chosen.[53,54] Further, in all cases, the RIJCOSX approximation was used,[55,56,57,58,59,60] employing Weigend's universal coulomb fitting basis (def2/J) as an auxiliary basis.[61] Thermodynamic corrections (zero point energy, enthalpy and entropy contributions) to the electronic energies were obtained at 298.15 K. The Gibbs free energy was computed according to the equations shown below:

$$\Delta G_{\text{solv}} = E_{\text{SCF, solv}} - E_{\text{SCF, gas}}$$

$$H_{\text{gas}} = E_{\text{SCF, gas}} + ZPE + \text{k}_{\text{b}}T$$

$$G_{\text{sol}} = H_{\text{gas}} - TS_{\text{gas}} + \Delta G_{\text{solv}} + 1.89 \text{ kcal mol}^{-1}$$

$$\Delta G_{\text{sol}} = \sum G_{\text{sol, products}} - \sum G_{\text{sol, reactants}}$$

Lastly, to ensure that the most stable conformer of intermediates was located, the global geometry optimization and ensemble generator algorithm as implemented in ORCA 6.1.0 was used where appropriate.[62] Given the significant computational cost, these conformer searches were performed using the semiempirical GFN2-xTB method as developed by the Grimme group.[63] To ensure that conformational energies were evaluated correctly, the 10 lowest lying conformers found by the GOAT algorithm were re-evaluated using the methods described above.

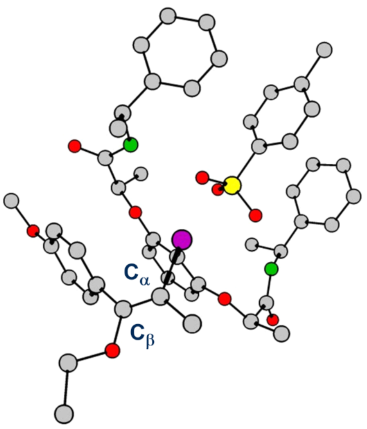

INT-4A
ΔG_{sol} = 0.00 kcal mol^{-1}

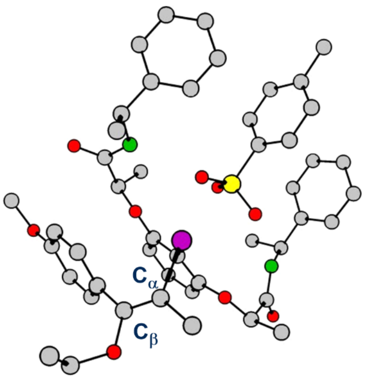

INT-4B
ΔG_{sol} = +0.13 kcal mol^{-1}

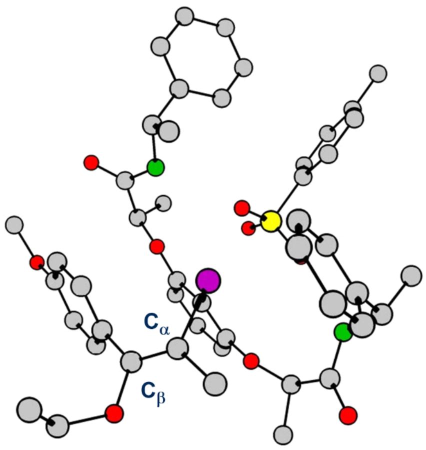

INT-4C
ΔG_{sol} = +0.63 kcal mol^{-1}

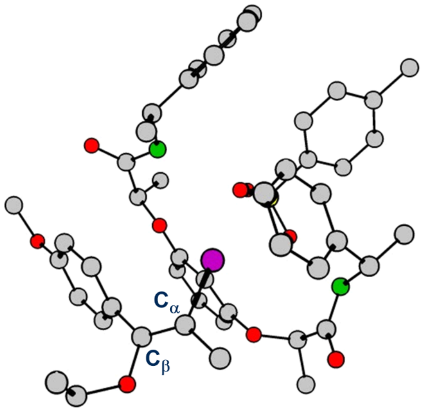

INT-4D
ΔG_{sol} = +0.89 kcal mol^{-1}

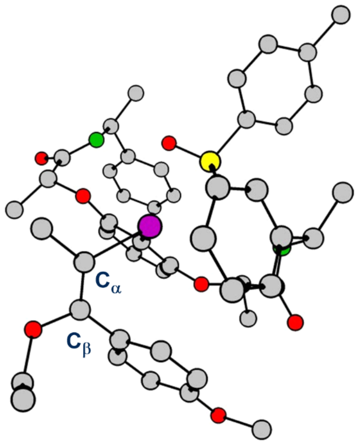

INT-4E
ΔG_{sol} = +1.11 kcal mol^{-1}

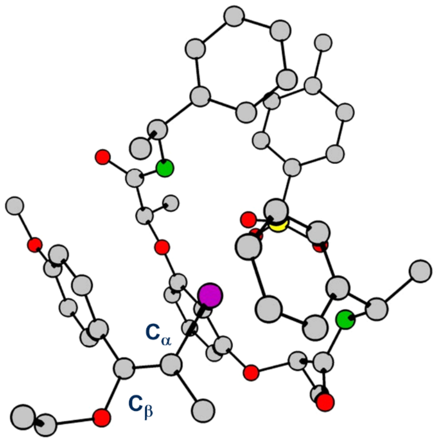

INT-4F
ΔG_{sol} = +1.14 kcal mol^{-1}

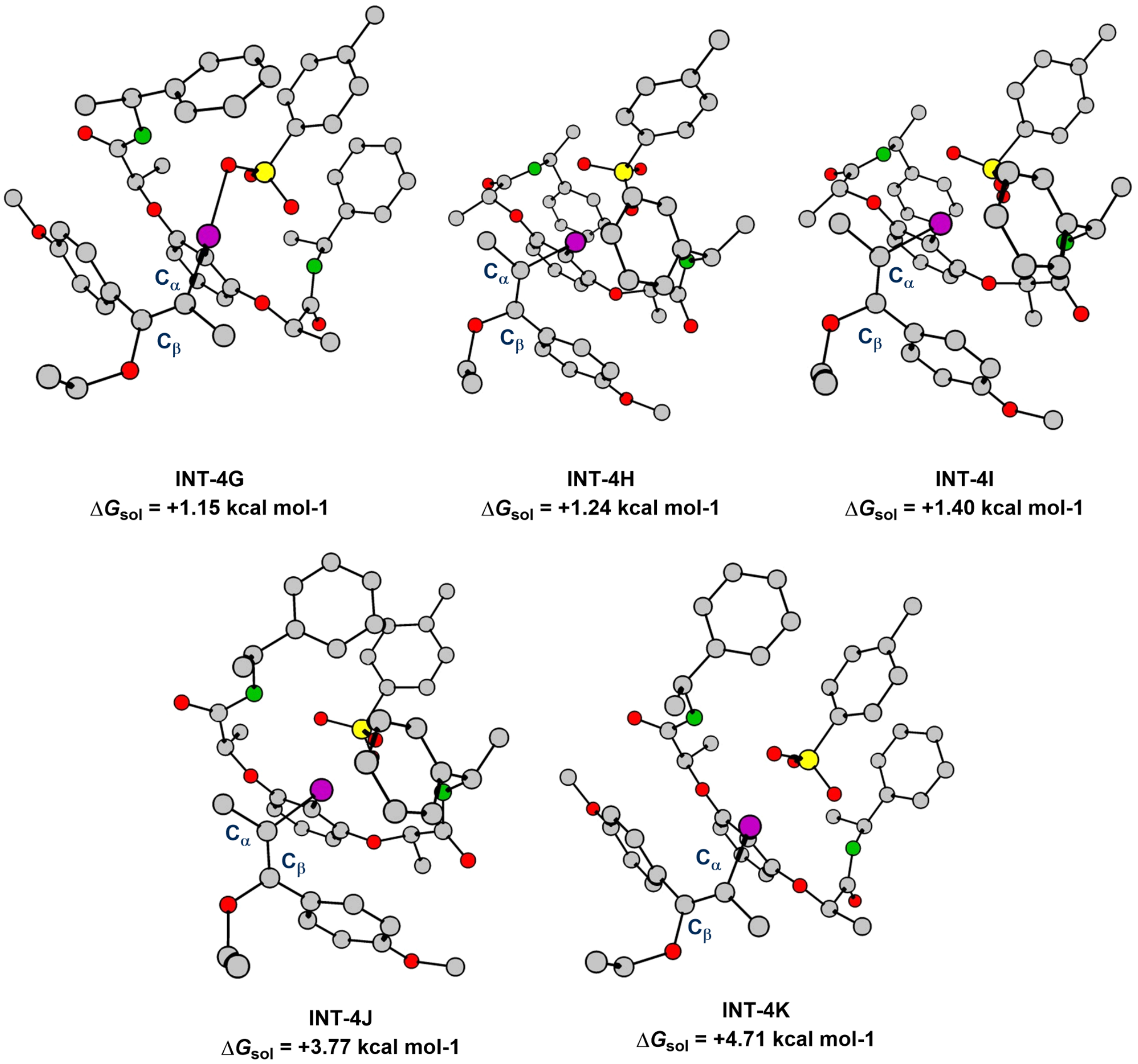

Figure S1. Conformers of intermediate **INT-4** ranked according to their difference in Gibbs free energy in solution, referenced to the most stable conformer found, **INT-4A**. Conformers have either open (*si*)-side, or open (*re*)-side, with respect to the ɑ–Carbon atom. Addition of AcOH to the (*si*)-side would lead to the major enantiomer, addition of AcOH to the (*re*)-side would lead to the minor enantiomer under the conditions described in the main text. Hydrogen atoms are omitted for clarity.

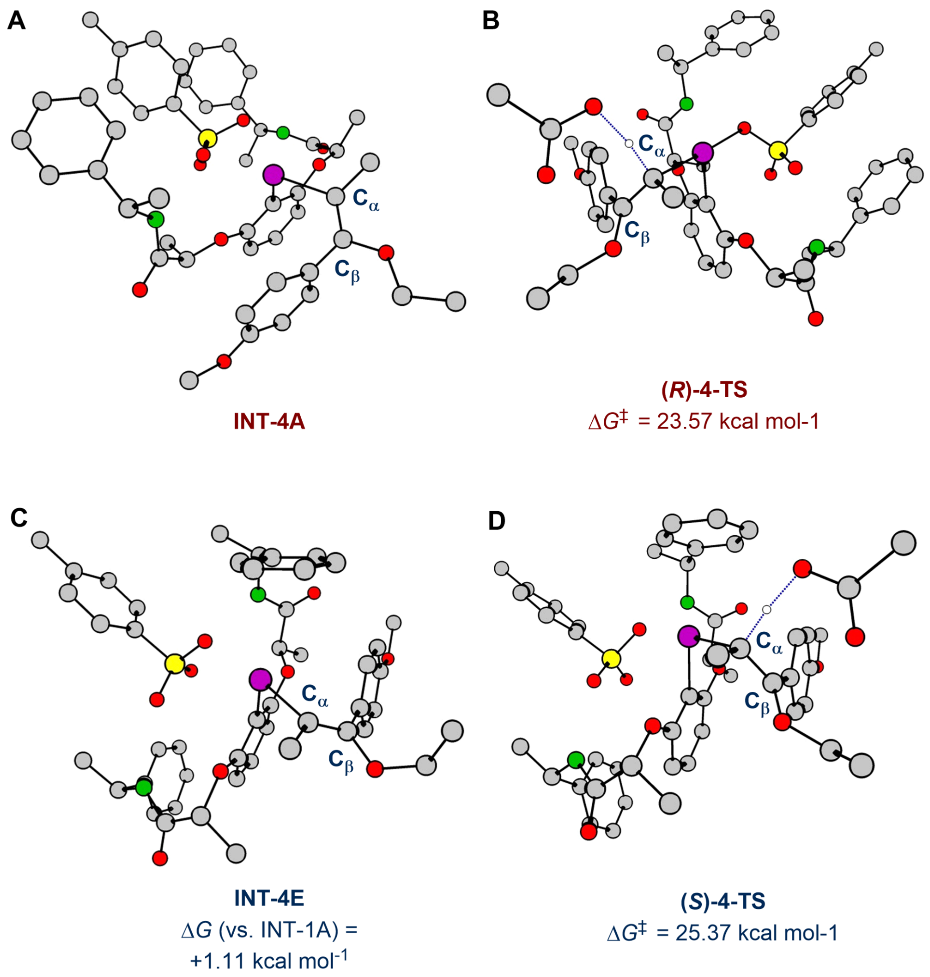

Figure S2. (A) Most stable conformers of **INT-4** with open *si*-side. **(B)** Transition state of the *syn*-addition of acetic acid to the C–C double bond (*R*)-**4-TS**, leading to the formation of an (*R*)-configured α-carbon atom, as observed in the major enantiomer under the investigated reaction conditions. **(C)** Most stable conformers of **INT-4** with open *re*-side. **(D)** Transition state of the *syn*-addition of acetic acid to the C–C double bond (*S*)-**4-TS**, leading to the formation of an (*S*)-configured α-carbon atom, as observed in the minor enantiomer under the investigated reaction conditions.

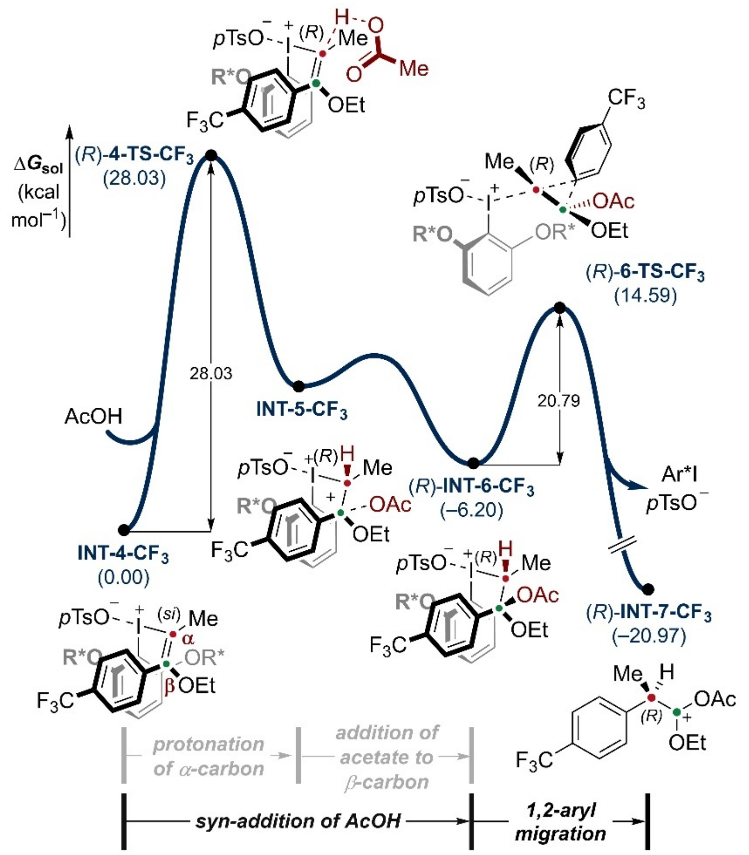

Figure S3. Calculated energy profiles (DFT) for competing protonation of **INT-4-CF₃** by acetic acid.

Energies of Computed Structures

Name	E(SCF-DZ, gas) (eV)	E(SCF- DZ, solv) (eV)	E(SCF-TZ, gas) (eV)	ZPE (kcal/mol)	E(TC) (kcal/mol)	H (eV)	S (eu)	G(gas) (eV)	G(solv) (kcal/mol)
**INT-1**	-61180.309	-61181.131	-61239.673	404.23	28.02	-61220.903	244.61	72.93	-61224.066
**INT-2**	-79297.487	-79298.445	-79370.286	455.58	31.79	-79349.126	269.33	80.30	-79352.608
**INT-3**	-91861.359	-91862.342	-91947.490	565.02	38.51	-91921.293	311.29	92.81	-91925.317
**INT-4A**	-89849.341	-89850.300	-89933.128	577.06	38.29	-89906.418	311.52	92.88	-89910.446
**INT-4A-CF3**	-95897.909	-95898.945	-95989.145	559.55	39.03	-95963.162	316.52	94.37	-95967.255
**INT-4B**	-89849.363	-89850.324	-89933.143	577.25	38.14	-89906.431	309.91	92.40	-89910.438
**INT-4C**	-89849.283	-89850.224	-89933.104	576.9	38.5	-89906.392	312.83	93.27	-89910.437
**INT-4D**	-89849.379	-89850.306	-89933.175	577.50	38.07	-89906.456	308.13	91.87	-89910.439
**INT-4E**	-89849.425	-89850.285	-89933.183	577.38	38.23	-89906.462	312.06	93.04	-89910.497
**INT-4F**	-89849.402	-89850.310	-89933.193	577.50	38.02	-89906.476	307.19	91.59	-89910.447
**INT-4G**	-89849.324	-89850.290	-89933.118	577.14	38.03	-89906.416	307.29	91.62	-89910.389
**INT-4H**	-89849.452	-89850.337	-89933.223	577.73	37.98	-89906.498	306.96	91.52	-89910.466
**INT-4I**	-89849.425	-89850.285	-89933.186	577.40	38.21	-89906.465	310.88	92.69	-89910.484
**INT-4J**	-89849.281	-89850.192	-89933.059	577.37	38.07	-89906.345	308.23	91.90	-89910.330
**INT-4K**	-89849.363	-89849.977	-89933.291	577.25	38.15	-89906.579	309.98	92.42	-89910.587
**(R)-4-TS**	-96075.177	-96076.298	-96166.149	614.50	41.36	-96137.683	332.22	99.05	-96141.978
**(R)-4-TS-CF3**	-102123.626	-102124.796	-102222.016	596.95	42.02	-102194.282	335.64	100.07	-102198.621
**(S)-4-TS**	-96075.396	-96076.349	-96166.275	614.62	41.32	-96137.805	329.7	98.30	-96142.068
**(R)-INT-6**	-96076.215	-96077.338	-96167.110	618.16	41.14	-96138.494	329.63	98.28	-96142.756
**(R)-INT-6-CF3**	-102124.899	-102126.009	-102223.213	600.86	41.74	-102195.322	333.09	99.31	-102199.628
**(S)-INT-6**	-96076.254	-96077.346	-96167.167	617.98	41.10	-96138.561	329.03	98.10	-96142.815
**(R)-6-TS**	-96074.830	-96076.604	-96165.786	616.36	41.35	-96137.239	332.82	99.23	-96141.542
**(R)-6-TS-CF3**	-102123.184	-102124.872	-102221.590	598.74	42.05	-102193.777	338.12	100.81	-102198.149
**(S)-6-TS**	-96075.220	-96076.636	-96166.237	615.90	41.69	-96137.695	334.26	99.66	-96142.017
**(R)-INT-7**	-22980.790	-22982.759	-23006.492	193.35	12.32	-22997.548	140.16	41.79	-22999.360
**(R)-INT-7-CF3**	-29029.266	-29031.428	-29062.424	176.04	12.98	-29054.202	146.24	43.60	-29056.092
**TsOH**	-24342.693	-24343.126	-24363.580	89.04	6.78	-24359.399	101.09	30.14	-24360.706
**TsO-**	-24328.181	-24330.771	-24349.533	81.58	6.38	-24345.693	99.38	29.63	-24346.978
**AcOH**	-6226.099	-6226.348	-6233.497	38.69	2.85	-6231.670	67.99	20.27	-6232.549
**Alkyne (15)**	-12562.332	-12562.556	-12576.200	106.71	6.83	-12571.251	101.59	30.29	-12572.564
**EtOH**	-4213.249	-4213.416	-4218.302	49.91	2.68	-4215.996	64.36	19.19	-4216.828
**Ph*I**	-48761.534	-48762.179	-48806.392	339.40	21.50	-48790.716	203.25	60.60	-48793.344

Frequencies of Optimised Structures

================================
INT-1
================================

13.33 20.37 22.15 28.88 32.52 36.01 40.91 45.35
49.57 52.88 58.63 64.32 65.40 68.82 69.80 76.28 78.34
80.17 82.25 86.60 90.38 111.86 121.30 129.97 137.83
140.66 147.40 153.46 166.16 186.01 191.73 198.74
206.01 213.25 215.91 219.78 227.21 235.23 247.79
252.87 256.64 275.69 284.76 286.56 297.57 305.65
311.48 318.08 320.66 333.39 352.51 371.12 403.88
415.22 418.25 441.58 456.16 457.60 497.16 506.90
508.81 514.04 517.93 523.30 555.76 560.37 580.30
598.70 607.58 608.72 611.23 621.40 631.35 632.61
635.42 649.06 655.69 673.65 687.43 717.86 721.94
723.12 738.57 753.43 767.99 771.37 778.50 780.23
790.59 797.06 805.39 821.20 863.71 863.90 865.24
869.77 907.80 919.29 920.99 936.63 943.83 950.23
951.66 956.99 966.71 996.13 1001.01 1007.53 1009.26
1009.79 1016.25 1016.99 1020.39 1020.57 1022.33 1039.29
1042.13 1042.87 1050.12 1050.42 1057.61 1058.74 1065.60
1085.71 1088.74 1090.01 1109.90 1118.27 1126.22 1127.34
1130.49 1138.08 1147.45 1163.71 1166.60 1169.89 1170.04
1195.04 1195.87 1196.82 1223.82 1228.84 1278.48 1281.70
1300.66 1314.31 1317.69 1319.91 1326.80 1330.76 1331.19
1345.30 1349.33 1358.13 1363.79 1365.19 1367.71 1375.64
1375.94 1377.03 1378.45 1379.85 1383.06 1392.58 1397.46
1400.80 1401.11 1435.92 1437.00 1448.54 1449.07 1450.80
1452.74 1460.54 1463.11 1466.21 1467.43 1475.53 1480.02
1482.70 1485.44 1489.98 1509.67 1527.76 1530.15 1579.59
1585.37 1623.91 1639.90 1643.62 1657.70 1662.80 1665.68
1742.45 1761.19 1792.59 1793.21 3032.14 3032.45 3033.51
3036.07 3045.49 3047.47 3049.11 3052.29 3066.41 3088.56
3121.22 3124.87 3125.81 3126.80 3128.52 3132.31 3134.08
3141.30 3152.28 3157.46 3167.01 3168.26 3168.83 3173.89
3176.82 3180.90 3183.34 3187.28 3192.86 3194.29 3205.33
3207.93 3213.12 3220.89 3223.79 3474.68 3491.96

================================
INT-2
================================

12.43 17.56 21.14 23.50 27.15 30.93 33.11 34.88
37.69 45.27 48.93 54.10 58.39 59.51 61.47 65.73 74.83
75.89 78.99 84.20 85.04 87.10 99.16 104.19 111.41
121.47 127.27 138.17 142.33 153.15 158.81 160.85
169.21 184.43 192.26 197.16 199.22 206.81 214.22
226.49 234.82 238.35 249.29 255.10 260.30 265.83
274.37 279.69 282.70 289.01 292.43 312.69 317.94
323.90 325.79 332.36 352.74 360.41 365.50 387.81
408.99 411.70 415.60 420.12 426.03 455.45 472.14
482.02 488.69 511.47 514.01 516.76 539.90 554.14
556.18 561.26 568.89 574.36 599.97 609.38 611.25
614.57 628.05 631.71 632.38 647.19 652.21 653.97
660.04 666.10 682.79 712.51 720.40 723.85 729.63
732.33 748.58 764.36 773.57 777.27 787.99 791.43
794.71 796.76 814.89 818.34 836.88 863.11 863.68
866.15 872.14 873.45 874.77 907.15 918.63 922.91
940.25 950.70 952.26 955.17 968.57 990.29 997.97
1000.16 1006.51 1008.38 1009.46 1012.43 1014.94 1015.59
1022.09 1023.51 1025.50 1030.40 1035.99 1038.95 1044.30
1051.09 1053.05 1054.01 1057.19 1062.84 1082.94 1088.02
1094.87 1096.74 1113.70 1118.42 1123.96 1127.14 1129.53
1135.40 1139.92 1147.60 1153.95 1156.66 1162.82 1169.07
1171.45 1193.47 1197.24 1197.83 1203.17 1227.86 1234.45
1237.86 1274.65 1292.46 1301.67 1306.13 1309.98 1315.66
1319.10 1319.47 1320.23 1328.00 1342.09 1349.11 1356.15
1364.24 1365.18 1366.25 1366.67 1373.54 1375.81 1379.64
1380.72 1389.54 1392.74 1394.35 1395.66 1401.20 1404.49
1430.95 1435.46 1447.21 1449.75 1455.98 1457.47 1460.50
1462.08 1463.86 1468.61 1469.77 1473.12 1479.25 1484.50
1487.50 1489.20 1511.07 1526.96 1529.35 1530.80 1576.03
1583.28 1623.33 1634.85 1636.98 1643.56 1656.42 1660.51
1662.18 1665.48 1761.64 1788.43 1794.77 3026.23 3026.63
3037.04 3037.21 3044.34 3048.73 3061.53 3066.38 3089.62
3094.82 3098.75 3118.90 3125.77 3126.18 3126.42 3131.03
3134.24 3141.37 3151.13 3153.19 3159.30 3164.53 3171.26
3172.68 3173.83 3176.08 3180.82 3181.65 3185.32 3187.98
3189.58 3191.64 3201.39 3204.23 3207.09 3211.34 3225.23
3227.73 3230.41 3497.31 3513.49

================================
INT-3
================================

13.66 15.87 18.67 20.88 23.98 25.15 30.50 35.41
42.01 42.90 47.80 49.55 53.32 56.20 58.98 63.21 68.61
70.88 73.31 80.14 83.79 86.57 88.15 94.43 97.12 110.81
111.70 113.49 118.35 122.06 128.74 131.58 136.95
142.91 147.68 147.97 155.46 160.07 167.27 169.79
174.49 176.71 178.41 184.70 193.04 195.54 207.83
215.07 217.88 227.12 233.76 239.92 244.26 250.77
255.76 262.24 265.38 272.45 273.92 279.06 284.24
286.38 302.72 303.85 308.91 322.51 327.04 334.79
342.52 344.14 352.90 359.18 378.62 389.27 398.78
400.84 418.79 421.73 424.81 426.93 444.68 457.91
463.65 475.60 479.95 490.65 494.36 512.33 515.58
524.16 533.07 539.15 555.41 560.71 567.73 568.84
583.63 605.16 610.01 611.16 611.89 615.65 622.59
632.80 634.07 638.69 643.86 645.34 662.75 667.54
680.48 697.24 720.08 725.01 726.58 728.65 728.92
742.96 760.78 765.31 778.71 786.37 789.12 791.19
797.54 802.83 814.27 822.02 823.87 829.30 831.92
837.39 844.73 854.49 858.92 863.33 876.18 879.16
880.16 902.66 916.54 922.17 939.30 942.94 950.66
954.64 962.85 971.42 983.35 994.98 995.51 1001.46
1002.81 1005.35 1011.13 1013.22 1014.11 1015.94 1017.51
1024.57 1026.10 1026.87 1028.57 1029.58 1033.79 1036.44
1037.26 1039.28 1045.01 1053.56 1055.50 1057.08 1059.13
1065.64 1070.24 1074.95 1085.17 1095.27 1097.46 1099.19
1107.62 1116.19 1119.28 1124.38 1125.18 1129.74 1133.90
1139.71 1144.03 1161.60 1163.49 1169.77 1170.04 1170.79
1182.87 1184.60 1191.38 1193.64 1195.87 1202.31 1210.76
1233.39 1236.99 1238.91 1279.88 1286.40 1293.61 1310.02
1314.18 1317.58 1317.72 1318.42 1323.25 1329.62 1331.23
1339.36 1349.35 1356.67 1359.82 1364.06 1365.38 1365.92
1369.06 1370.40 1378.46 1380.95 1381.74 1387.13 1389.58
1389.86 1392.51 1393.96 1395.89 1400.07 1407.70 1408.61
1434.53 1443.16 1444.54 1448.65 1456.14 1456.21 1457.52
1458.81 1460.70 1461.44 1461.94 1462.93 1466.72 1468.24
1472.61 1475.48 1480.40 1482.47 1485.67 1486.78 1491.86
1502.02 1510.13 1527.18 1527.57 1534.43 1554.35 1595.84
1611.33 1624.32 1625.57 1637.07 1638.66 1644.83 1657.58
1659.69 1661.42 1667.05 1676.76 1694.17 1714.29 1774.66
1775.92 3000.86 3025.90 3031.50 3035.95 3037.53 3044.85
3047.94 3055.96 3059.85 3070.81 3088.45 3096.45 3096.75
3102.05 3116.10 3121.39 3122.16 3123.49 3124.62 3128.50

3128.82 3131.67 3140.42 3147.22 3148.44 3152.97 3158.23
3162.31 3164.62 3170.57 3172.00 3179.68 3180.83 3180.85
3184.19 3186.55 3188.75 3193.22 3194.28 3194.77 3196.44
3197.60 3208.93 3209.17 3211.11 3215.50 3221.83 3230.01
3230.36 3322.84 3369.01

==============================

INT-4A

==============================

8.46 16.08 16.97 17.51 19.51 20.33 25.46 30.37 34.69
39.61 40.09 47.89 51.48 53.48 56.01 59.68 65.86 70.89
75.66 79.34 84.44 86.10 87.07 88.40 90.44 93.48 101.11
104.53 107.35 110.98 114.40 126.91 129.38 140.06
142.70 145.51 157.32 161.64 169.57 173.02 182.02
184.84 190.46 194.06 195.08 212.99 224.23 230.78
233.60 236.65 243.57 245.75 251.20 255.84 260.78
263.90 265.77 272.19 275.70 282.45 294.80 301.56
309.48 319.11 324.27 325.80 329.87 340.76 347.32
355.44 357.58 365.58 373.02 384.67 394.82 394.91
402.11 410.21 420.25 422.95 427.79 464.50 478.73
489.57 491.92 495.78 510.03 515.43 523.05 538.58
551.70 553.16 564.62 565.67 575.27 576.49 596.43
607.45 610.46 620.31 629.33 634.12 634.90 640.82
643.92 647.60 669.04 671.94 693.28 701.04 718.20
723.40 725.12 727.10 746.59 749.19 753.30 760.83
769.20 784.35 792.30 797.12 799.28 802.68 816.83
819.69 829.40 829.78 833.02 835.39 841.96 856.54
861.06 863.79 866.85 872.80 879.32 909.93 917.84
923.82 925.15 935.40 943.00 949.43 967.35 969.15
974.30 981.76 993.72 997.42 998.98 999.64 1000.79
1006.64 1010.73 1014.56 1016.44 1016.94 1020.65 1028.44
1031.00 1033.52 1034.19 1038.31 1041.34 1047.57 1051.80
1052.17 1055.65 1062.05 1064.48 1066.58 1074.96 1090.81
1092.35 1095.60 1105.26 1109.04 1118.91 1122.31 1124.94
1125.48 1126.38 1126.74 1134.22 1138.25 1141.08 1155.11
1157.26 1160.11 1168.46 1169.29 1170.63 1180.96 1184.49
1185.76 1191.35 1196.52 1201.85 1205.47 1210.92 1225.05
1228.87 1235.46 1237.62 1291.24 1295.30 1299.03 1306.17
1313.13 1314.95 1317.64 1319.43 1320.72 1324.35 1330.66
1333.55 1340.18 1354.83 1356.12 1363.27 1363.40 1364.65
1366.69 1366.92 1371.47 1377.63 1381.80 1385.08 1387.29
1387.60 1389.15 1395.99 1396.20 1400.74 1406.99 1421.78
1430.16 1447.01 1454.35 1456.22 1457.89 1458.57 1459.62
1459.80 1460.85 1464.31 1466.48 1467.83 1469.14 1471.29
1474.36 1475.74 1480.12 1480.58 1485.26 1486.94 1492.45
1502.68 1503.72 1514.11 1531.35 1532.42 1533.75 1555.09
1580.43 1605.13 1621.73 1624.24 1636.48 1644.12 1645.04
1662.02 1664.42 1667.29 1668.62 1675.28 1690.39 1769.39
1777.15 3005.29 3020.59 3021.79 3027.25 3027.48 3037.35
3038.33 3041.02 3050.33 3054.59 3061.56 3080.53 3080.79
3090.79 3096.32 3098.08 3105.25 3115.79 3119.99 3121.37
3123.70 3126.92 3128.01 3129.87 3140.86 3141.51 3141.72
3143.66 3147.40 3163.76 3164.63 3167.94 3173.16 3173.47
3174.20 3178.69 3183.40 3185.43 3185.49 3187.40 3190.15
3196.85 3198.60 3207.22 3210.09 3213.87 3216.04 3222.50
3226.70 3229.23 3230.23 3362.73 3381.77

==============================

INT-4A-CF3

==============================

10.37 12.17 16.85 19.24 21.06 25.00 25.67 30.07
34.49 39.80 41.30 45.29 46.36 50.93 51.89 55.62 59.56
63.54 68.48 74.42 76.75 81.64 83.54 85.87 87.65 88.54
92.62 95.93 103.15 107.90 109.61 111.95 123.26 129.35
132.16 138.78 144.95 149.35 158.76 164.61 171.35
179.77 183.50 187.87 192.88 205.34 211.79 222.09
224.96 232.12 237.11 243.01 245.24 246.98 254.49
261.43 264.13 267.45 274.57 280.79 292.07 293.74
302.45 309.26 323.56 325.52 327.92 338.93 342.79
353.20 357.07 368.40 373.89 384.23 394.36 394.49
401.18 410.29 410.44 416.88 420.29 422.54 458.67
466.58 480.67 491.34 496.06 503.24 510.06 515.48
537.98 552.75 554.08 564.60 565.13 568.72 571.86
583.03 594.15 605.43 610.37 623.30 630.73 634.24
635.48 640.32 643.63 647.48 668.24 671.92 681.28
691.58 699.22 718.17 724.82 725.70 727.20 744.77
752.17 755.78 769.50 774.34 779.34 791.72 797.45
798.36 800.35 809.37 813.76 818.45 820.00 833.08
840.80 856.57 857.78 861.09 865.14 866.45 873.01
882.46 909.89 916.86 923.09 924.92 935.58 942.43
948.96 965.08 966.61 981.95 986.71 996.77 998.87
1001.14 1001.61 1002.23 1006.97 1014.20 1015.61 1016.48
1017.14 1020.85 1029.18 1033.57 1034.29 1036.69 1038.44
1041.68 1047.50 1051.97 1052.25 1055.41 1060.89 1064.23
1072.79 1090.01 1091.56 1093.08 1095.00 1104.57 1111.22
1117.67 1121.06 1125.35 1125.80 1126.68 1126.91 1133.69
1135.41 1139.86 1154.75 1156.93 1159.49 1167.03 1168.58
1169.38 1170.41 1182.49 1191.60 1195.79 1196.52 1202.11
1204.99 1225.49 1226.78 1230.84 1233.66 1237.58 1291.42
1297.10 1300.07 1307.77 1313.30 1313.96 1319.58 1320.80
1325.92 1330.92 1332.39 1341.39 1349.61 1355.10 1358.96
1363.43 1364.28 1366.03 1367.34 1368.23 1371.26 1377.42
1382.70 1383.98 1385.82 1386.50 1390.17 1395.99 1396.58
1399.98 1409.01 1422.67 1430.94 1440.32 1446.35 1454.40
1456.43 1457.25 1459.13 1459.81 1461.04 1462.93 1468.63
1469.01 1469.59 1474.50 1475.86 1480.09 1485.29 1486.30
1491.19 1501.76 1513.57 1531.26 1532.46 1534.00 1549.85
1582.55 1598.93 1622.86 1633.17 1636.57 1643.88 1644.97
1660.99 1664.40 1667.37 1668.54 1678.32 1696.64 1777.23
1783.39 3019.96 3023.64 3028.09 3030.34 3038.09 3039.47
3041.05 3048.89 3056.13 3062.18 3078.97 3091.12 3093.23
3102.27 3117.25 3118.34 3120.17 3121.70 3123.76 3128.50
3129.48 3129.59 3139.33 3142.11 3143.17 3145.26 3164.22
3164.78 3173.49 3173.72 3173.90 3174.96 3178.59 3185.30
3187.68 3190.52 3190.66 3194.32 3196.89 3197.17 3204.94
3209.19 3213.10 3216.89 3223.31 3224.79 3227.45 3229.82
3359.25 3412.63

==============================

INT-4B

==============================

8.19 16.81 17.50 19.29 20.77 24.56 28.83 30.68 35.23
40.09 43.85 49.15 51.60 53.72 57.73 62.96 70.42 74.18
75.20 78.99 84.15 85.43 88.15 90.25 93.78 97.09 101.77
106.06 109.31 111.12 126.95 129.22 130.09 140.37
143.38 148.86 159.05 163.92 166.51 173.60 181.50
187.04 191.82 194.31 196.06 212.91 224.24 231.07
234.97 239.20 244.38 247.89 250.51 255.03 259.76
265.20 268.09 273.82 277.64 290.88 294.73 301.83
309.75 323.80 325.35 330.08 340.53 348.59 355.67
358.85 366.30 371.21 374.13 384.83 394.59 394.68
410.33 420.45 422.76 426.33 443.35 464.64 478.66
490.75 491.95 496.20 502.22 510.22 515.67 538.39
551.91 553.12 564.65 565.41 569.22 575.23 596.25
607.39 610.41 617.65 629.34 634.08 634.94 640.30
643.80 647.55 669.02 671.76 693.30 703.33 718.24
723.36 725.09 727.33 744.15 746.99 753.28 758.25
769.35 784.39 792.29 797.06 799.21 802.65 805.72
819.75 829.32 829.77 833.20 835.73 842.03 856.60

861.06 864.05 866.96 872.81 879.33 907.68 910.01
917.87 925.24 935.42 943.00 949.41 967.37 969.16
974.88 981.76 994.46 996.72 998.74 999.65 1000.99
1006.11 1006.67 1014.52 1016.47 1016.90 1020.61 1028.42
1029.55 1033.63 1034.37 1038.37 1039.06 1043.18 1047.51
1052.16 1055.65 1062.04 1064.32 1066.61 1074.92 1090.84
1092.41 1095.10 1098.46 1105.35 1118.88 1119.96 1123.22
1124.96 1125.47 1126.33 1127.48 1138.25 1141.16 1155.13
1157.38 1160.06 1163.98 1168.50 1169.29 1182.49 1185.27
1191.35 1192.21 1196.58 1201.91 1205.47 1210.98 1225.00
1228.88 1235.48 1237.49 1291.34 1295.58 1299.14 1313.15
1314.98 1317.10 1317.74 1320.47 1324.27 1324.91 1330.86
1333.63 1340.25 1354.92 1356.24 1363.38 1363.53 1364.48
1366.80 1367.09 1371.52 1377.71 1381.55 1384.94 1387.24
1387.42 1388.58 1396.00 1396.16 1400.67 1407.08 1415.50
1430.71 1446.43 1455.82 1456.53 1457.89 1458.74 1459.59
1459.74 1460.87 1464.24 1466.53 1468.63 1469.15 1469.44
1471.24 1475.65 1480.16 1480.56 1485.34 1486.94 1487.33
1492.66 1503.73 1514.02 1531.32 1532.45 1533.79 1554.86
1580.68 1605.11 1621.38 1624.23 1636.51 1644.12 1645.10
1662.09 1664.48 1667.32 1668.63 1675.14 1689.61 1769.22
1776.94 3005.33 3022.98 3027.05 3027.19 3034.88 3037.18
3040.99 3050.18 3054.73 3056.71 3061.51 3079.82 3090.40
3096.39 3097.98 3105.37 3109.01 3115.62 3120.14 3120.57
3121.00 3123.40 3129.90 3130.38 3141.20 3141.66 3142.31
3143.59 3147.59 3163.74 3164.65 3168.39 3172.92 3173.34
3174.62 3178.60 3182.55 3185.41 3187.08 3187.40 3190.13
3196.76 3198.57 3207.54 3210.07 3213.65 3216.20 3222.57
3226.60 3229.31 3229.99 3361.04 3381.39
==============================
INT-4C
==============================
9.19 13.96 16.37 18.96 20.84 25.60 26.22 31.81 34.84
36.20 39.59 43.69 44.88 48.11 50.34 55.09 55.80 58.83
62.23 65.07 73.28 78.17 84.05 86.00 94.63 97.69 103.47
104.84 108.28 115.13 119.26 125.65 130.55 137.03
138.98 144.61 157.18 159.05 172.00 172.50 176.73
180.11 188.84 192.44 195.85 200.62 211.87 223.00
229.24 234.23 241.30 244.09 248.88 254.75 255.24
260.53 269.49 272.01 274.65 280.98 290.62 293.06
299.06 307.63 320.41 327.08 328.97 338.73 350.55
357.06 361.88 366.37 373.22 390.27 394.62 400.76
416.91 419.84 420.64 426.99 444.07 448.88 471.64
482.90 491.20 493.79 500.77 510.35 526.75 528.94
555.02 556.25 563.74 568.03 569.92 571.13 594.29
606.34 614.97 616.71 619.44 634.00 634.51 636.74
640.29 648.87 667.01 677.83 691.93 702.25 718.66
722.86 725.79 730.68 742.07 743.90 755.98 759.00
768.55 782.94 788.95 791.35 794.86 801.54 805.95
819.45 820.03 829.10 830.33 833.54 837.31 857.22
860.18 864.62 868.45 872.70 873.88 902.80 907.10
919.44 923.93 939.71 947.42 950.85 962.07 971.67
976.15 984.74 994.62 995.23 997.91 1005.47 1005.81
1007.02 1010.16 1016.51 1018.06 1018.94 1020.86 1027.63
1029.23 1031.41 1033.41 1034.76 1035.66 1038.51 1045.06
1052.95 1053.23 1053.36 1060.18 1066.26 1076.39 1086.81
1095.35 1096.60 1100.06 1107.37 1109.37 1118.68 1120.26
1122.94 1126.75 1127.43 1128.08 1135.97 1141.06 1148.05
1164.49 1165.64 1166.81 1167.44 1168.98 1182.49 1185.72
1191.00 1192.27 1193.91 1195.09 1197.37 1210.41 1235.53
1236.06 1237.28 1240.65 1276.32 1295.17 1299.47 1312.41
1312.76 1314.12 1314.46 1316.37 1318.16 1319.54 1324.07
1330.67 1341.58 1350.67 1358.62 1361.81 1362.32 1364.05
1366.05 1370.21 1380.05 1383.48 1386.69 1389.29 1393.19
1393.41 1396.19 1396.63 1398.72 1405.56 1406.95 1415.21
1429.36 1447.42 1451.00 1455.78 1456.71 1456.89 1457.83
1459.61 1460.87 1461.82 1463.83 1466.36 1467.50 1469.10
1471.05 1472.75 1479.60 1483.43 1486.78 1486.91 1488.36
1490.27 1502.52 1516.14 1527.17 1529.62 1531.42 1554.50
1589.30 1599.87 1622.34 1625.79 1633.10 1641.55 1645.41
1659.91 1661.99 1663.10 1666.20 1675.28 1694.62 1773.25
1780.87 3003.63 3024.13 3027.08 3034.49 3038.66 3040.36
3049.84 3051.50 3052.73 3056.17 3089.69 3089.72 3090.77
3092.06 3100.72 3107.03 3113.73 3116.00 3120.08 3122.52
3125.03 3125.37 3129.56 3136.20 3136.43 3141.46 3142.71
3145.67 3152.33 3163.39 3167.65 3172.80 3173.08 3173.57
3175.19 3177.29 3182.26 3187.11 3187.20 3187.39 3187.51
3194.92 3199.49 3206.63 3211.36 3215.46 3217.76 3221.14
3229.24 3229.41 3237.58 3407.20 3418.29
==============================
INT-4D
==============================
12.28 14.58 17.56 21.00 23.44 27.61 28.45 35.43
39.17 45.54 48.07 49.18 51.49 55.10 58.16 58.72 63.34
66.41 68.14 73.04 76.52 79.94 83.35 91.34 98.15 102.76
109.34 110.94 113.11 118.97 125.29 127.52 137.93
144.02 150.81 158.66 162.00 167.67 176.36 178.60
186.24 191.93 194.33 198.37 206.43 221.90 222.63
229.24 233.57 235.93 239.13 247.19 249.00 249.65
261.80 263.04 271.73 274.82 277.90 292.36 295.86
302.36 306.84 311.42 330.05 330.65 340.96 344.80
355.36 363.39 365.39 370.08 376.80 387.94 392.21
407.14 417.25 420.45 421.89 426.61 444.33 452.23
480.37 485.12 491.01 491.86 501.29 508.73 511.64
527.27 537.52 554.55 557.48 564.25 569.83 571.23
590.68 607.07 613.14 615.72 617.83 633.23 633.80
639.50 641.21 648.55 668.18 685.90 701.67 706.20
721.57 724.41 727.34 731.85 741.93 745.61 759.20
764.15 767.70 781.76 791.33 802.28 804.40 806.16
807.94 819.50 822.82 829.45 835.12 837.71 846.47
856.07 862.91 864.70 867.66 877.11 879.34 904.71
906.67 920.37 923.90 939.71 943.30 952.26 962.86
974.28 977.28 986.14 995.28 996.84 997.15 1003.74
1005.47 1007.53 1009.79 1015.15 1018.07 1021.30 1025.72
1027.10 1029.21 1032.32 1034.67 1035.96 1040.27 1042.00
1043.63 1052.92 1053.83 1056.93 1062.91 1068.01 1071.96
1086.84 1094.59 1096.18 1096.76 1105.95 1107.77 1117.41
1119.48 1123.02 1125.52 1126.44 1129.44 1136.20 1141.55
1144.41 1162.21 1162.71 1169.37 1170.11 1172.17 1181.95
1185.09 1190.61 1191.15 1191.74 1196.28 1199.11 1210.91
1232.05 1234.69 1236.22 1237.56 1277.75 1292.30 1293.72
1311.35 1313.15 1313.93 1316.68 1319.30 1320.10 1323.58
1327.28 1338.65 1340.35 1354.41 1359.62 1361.57 1362.63
1364.30 1365.86 1367.15 1378.00 1382.52 1384.62 1387.67
1393.11 1395.32 1395.76 1396.92 1401.22 1406.96 1412.74
1415.14 1428.99 1448.53 1449.76 1455.99 1455.99 1457.50
1462.75 1464.74 1466.03 1466.93 1467.28 1467.53 1468.68
1469.18 1473.83 1478.99 1480.88 1480.90 1481.24 1487.22
1487.70 1488.07 1502.90 1514.20 1528.51 1529.06 1529.90
1554.41 1596.67 1610.51 1622.68 1625.74 1632.22 1640.58
1641.24 1657.38 1662.43 1662.70 1665.24 1675.69 1694.20
1768.41 1769.57 3004.61 3023.42 3034.01 3038.74 3039.01
3043.91 3044.55 3046.93 3049.73 3056.56 3084.86 3089.36
3089.78 3090.85 3103.91 3105.91 3119.51 3122.27 3122.89
3123.25 3124.49 3126.27 3128.64 3129.97 3132.23 3133.76
3146.47 3149.22 3150.80 3161.38 3168.79 3168.86 3170.99

3181.33 3182.68 3183.40 3187.83 3188.21 3191.33 3194.04
3196.51 3199.66 3200.63 3206.25 3206.28 3206.54 3218.65
3228.66 3229.52 3230.39 3238.78 3325.80 3369.63
===============================
INT-4E
===============================
5.71 11.02 12.89 16.67 18.11 20.07 24.16 26.99 28.83
37.50 42.74 43.57 49.54 54.88 58.11 59.70 61.69 66.14
70.10 75.98 77.35 81.45 86.72 90.56 94.47 103.55
105.10 109.30 112.51 113.94 122.98 130.62 139.68
144.24 147.22 150.61 156.99 160.69 169.89 174.13
178.19 184.58 192.44 200.37 203.00 211.63 223.55
231.34 234.93 239.19 241.40 245.31 252.55 254.77
263.70 265.58 275.33 276.84 277.80 289.06 295.25
297.96 310.46 315.21 324.78 330.67 339.72 345.36
350.67 357.25 366.49 369.42 377.58 391.61 397.44
417.64 418.43 420.17 428.28 442.03 445.82 452.11
475.22 479.14 494.42 497.01 501.86 513.97 523.48
526.88 538.26 555.05 558.01 564.03 566.26 568.07
574.39 597.70 611.46 616.53 617.35 632.25 633.67
635.11 641.62 647.89 651.99 669.24 690.06 703.04
719.29 722.12 723.96 730.86 737.41 743.89 760.08
765.05 776.30 779.38 796.28 798.40 800.51 806.45
809.92 815.42 819.42 830.04 831.45 836.07 840.17
857.65 860.94 863.09 867.98 869.05 888.82 895.69
906.73 924.32 928.17 937.86 944.07 947.44 960.54
978.80 981.46 985.32 994.92 998.26 1003.22 1003.31
1004.60 1005.10 1005.84 1015.81 1017.16 1018.73 1024.57
1025.06 1030.27 1031.31 1033.63 1036.67 1037.86 1039.80
1041.73 1051.12 1052.05 1055.61 1061.94 1069.51 1074.26
1085.23 1091.03 1095.84 1099.44 1106.02 1118.97 1120.37
1123.86 1124.66 1127.38 1128.49 1137.38 1139.45 1143.81
1149.86 1162.38 1163.17 1163.75 1165.39 1168.57 1182.92
1186.04 1189.22 1189.78 1192.45 1196.11 1206.69 1209.88
1231.66 1233.64 1234.85 1236.68 1270.48 1283.01 1293.07
1299.07 1311.39 1313.01 1314.61 1317.70 1318.76 1322.13
1325.89 1328.70 1333.45 1357.93 1360.02 1361.92 1363.39
1366.93 1368.31 1377.69 1379.88 1384.17 1386.87 1392.02
1393.14 1395.96 1397.55 1398.34 1399.68 1400.98 1415.17
1420.04 1428.65 1445.52 1455.66 1457.28 1458.53 1459.03
1459.70 1459.93 1461.15 1462.40 1466.35 1466.65 1469.52
1469.80 1470.61 1477.82 1478.29 1481.16 1481.36 1485.58
1486.88 1487.18 1500.67 1512.06 1526.33 1527.51 1528.98
1554.83 1567.06 1607.78 1624.24 1626.14 1633.66 1639.64
1643.06 1658.43 1661.07 1662.31 1663.43 1675.67 1689.00
1768.19 1772.07 3004.43 3023.76 3025.25 3033.22 3036.72
3038.84 3055.16 3055.25 3058.34 3063.52 3069.71 3089.58
3090.64 3093.23 3096.35 3101.37 3107.69 3118.86 3119.25
3124.32 3124.53 3125.77 3128.71 3139.64 3140.99 3142.14
3147.33 3147.44 3159.87 3163.05 3167.54 3170.41 3173.81
3176.05 3177.36 3185.91 3187.09 3187.63 3189.46 3193.88
3194.40 3205.73 3207.17 3208.60 3209.58 3211.32 3214.86
3237.56 3240.88 3251.32 3251.46 3340.53 3459.17
===============================
INT-4F
===============================
9.12 17.63 23.28 25.85 28.11 32.18 35.29 39.79 41.67
45.16 50.34 52.16 55.15 56.65 60.84 63.43 65.89 72.22
77.96 80.17 80.81 84.62 89.13 97.06 101.27 102.77
104.88 111.19 114.47 120.43 127.02 134.16 136.25
141.58 146.41 150.65 157.52 164.46 167.43 171.64
175.54 185.84 191.23 195.25 198.53 216.91 229.92
231.69 235.37 238.32 241.31 244.91 250.03 254.24
258.43 262.71 268.77 270.41 274.67 280.47 293.39
302.78 305.23 309.94 319.64 328.52 336.60 350.07
352.74 361.02 366.16 370.41 376.83 384.33 389.02
394.58 416.81 418.86 426.06 427.30 445.25 449.43
482.65 490.76 491.85 495.64 501.58 503.69 521.36
526.64 545.41 553.71 554.67 566.10 570.01 574.63
590.97 607.32 614.63 615.80 616.98 633.08 634.17
640.01 645.13 648.49 669.31 690.06 701.59 717.05
719.36 724.91 727.46 736.74 742.75 749.96 758.72
760.18 781.44 784.60 792.62 801.99 805.34 806.56
819.33 823.33 827.66 829.75 834.67 836.40 838.79
857.64 860.28 865.92 867.99 870.53 894.89 904.32
905.82 918.96 926.17 939.12 946.05 952.57 963.16
970.11 975.06 983.63 996.55 998.01 1001.31 1001.41
1001.79 1005.74 1006.71 1016.76 1017.93 1018.56 1024.15
1029.09 1029.30 1030.40 1033.12 1034.55 1036.69 1040.69
1045.79 1049.69 1052.11 1056.97 1058.06 1069.06 1075.86
1085.16 1090.18 1093.69 1098.99 1100.64 1102.40 1108.23
1118.07 1119.04 1124.20 1126.39 1130.49 1136.96 1140.12
1143.81 1153.73 1161.08 1162.02 1169.55 1170.80 1181.72
1182.96 1186.61 1191.09 1195.14 1195.89 1203.87 1209.90
1231.26 1235.18 1238.94 1239.33 1279.22 1284.48 1291.80
1310.34 1314.02 1315.52 1316.23 1316.59 1321.98 1323.58
1330.06 1335.37 1339.82 1355.95 1364.15 1364.63 1365.54
1367.05 1369.48 1372.15 1378.61 1383.11 1388.59 1389.43
1390.64 1393.61 1394.40 1396.20 1399.27 1404.99 1414.85
1419.68 1431.95 1448.54 1455.22 1455.46 1455.71 1457.56
1460.14 1462.35 1464.19 1465.50 1466.54 1468.27 1468.83
1469.04 1469.25 1475.10 1477.16 1477.80 1486.15 1486.63
1487.28 1487.42 1499.92 1512.02 1528.68 1533.10 1534.97
1554.19 1589.61 1608.72 1622.30 1625.87 1634.64 1641.52
1645.07 1652.02 1661.14 1665.21 1667.74 1675.92 1692.35
1767.69 1772.81 3006.06 3025.24 3032.87 3034.32 3037.67
3038.25 3046.06 3048.21 3052.90 3069.88 3080.68 3086.60
3093.07 3100.44 3106.32 3108.35 3119.38 3120.21 3122.53
3123.07 3124.37 3125.30 3127.03 3128.86 3129.06 3136.36
3142.69 3148.46 3149.60 3163.99 3164.27 3171.37 3171.92
3174.94 3180.66 3183.62 3185.39 3186.19 3187.74 3188.94
3195.54 3198.32 3201.43 3205.91 3210.39 3214.17 3215.65
3217.64 3226.15 3230.97 3234.29 3309.84 3396.81
===============================
INT-4G
===============================
14.47 17.52 20.78 21.63 27.83 30.26 32.49 38.87
39.87 43.54 45.21 50.30 51.89 57.72 63.32 64.66 71.29
74.94 76.93 79.48 84.44 85.36 87.90 89.09 92.13 97.07
101.89 104.74 112.02 115.69 127.12 129.80 131.47
141.57 142.06 152.23 159.99 166.02 168.44 176.85
184.64 190.38 194.77 198.86 213.61 222.67 227.50
234.51 237.28 239.64 245.61 247.54 250.57 255.89
259.28 265.88 267.96 269.61 276.98 288.13 290.28
295.80 302.82 320.89 326.65 327.76 343.93 349.58
356.46 361.80 366.94 369.79 373.33 378.72 391.26
394.06 412.25 418.84 419.76 427.00 443.43 462.28
480.25 491.63 499.08 499.51 502.15 510.11 515.81
535.01 541.46 554.39 563.58 564.24 570.81 574.61
595.70 604.54 610.51 617.44 630.55 634.15 635.16
640.04 644.03 647.73 668.58 671.13 693.36 704.34
717.19 719.08 724.54 726.45 744.55 747.33 751.79
758.26 769.91 780.87 786.59 790.54 795.54 797.42
804.36 805.77 819.00 829.34 834.35 836.73 847.38
856.87 860.30 862.12 863.90 867.72 880.94 907.96
911.41 922.27 924.89 934.05 936.67 949.61 968.38

974.50 979.52 982.66 994.35 996.68 998.05 998.36
999.85 1001.86 1006.26 1015.40 1016.98 1017.10 1019.95
1027.00 1029.32 1034.09 1034.46 1039.04 1040.78 1043.58
1046.98 1050.47 1056.50 1061.25 1063.64 1068.21 1074.56
1090.08 1092.43 1096.93 1099.74 1105.29 1120.35 1122.89
1123.48 1124.01 1125.63 1126.24 1127.00 1139.29 1141.53
1156.04 1156.76 1160.66 1163.89 1168.08 1169.99 1181.58
1184.57 1190.44 1192.07 1192.21 1196.85 1205.29 1209.82
1220.66 1223.65 1227.52 1237.24 1290.57 1295.63 1300.57
1303.96 1312.95 1313.72 1316.41 1319.76 1322.96 1324.90
1331.09 1333.40 1339.07 1353.97 1355.97 1356.82 1363.01
1364.00 1364.86 1366.79 1368.73 1377.10 1378.69 1381.56
1384.01 1386.96 1388.59 1395.84 1396.04 1401.24 1406.03
1415.39 1429.65 1446.40 1453.73 1455.86 1457.77 1458.67
1459.64 1460.04 1464.58 1466.80 1467.18 1469.12 1469.45
1472.56 1474.68 1480.13 1480.55 1485.11 1485.52 1487.25
1488.91 1491.54 1500.10 1512.77 1531.81 1532.35 1532.61
1554.53 1584.14 1595.67 1621.89 1624.32 1636.85 1644.73
1646.73 1660.63 1664.43 1667.96 1669.15 1675.54 1690.06
1770.63 1778.03 3004.99 3016.49 3021.57 3026.90 3034.85
3036.40 3041.01 3041.13 3052.45 3052.66 3055.61 3061.19
3072.93 3091.82 3097.25 3104.01 3108.44 3120.44 3120.57
3121.46 3123.31 3130.15 3130.34 3139.12 3139.92 3141.30
3141.92 3146.26 3151.63 3162.38 3163.41 3165.76 3170.63
3171.20 3172.60 3176.47 3181.92 3183.91 3186.17 3186.90
3190.08 3192.17 3194.97 3206.83 3207.48 3207.68 3222.85
3225.79 3226.18 3228.13 3231.30 3354.16 3397.38
==============================
INT-4H
==============================
12.52 16.71 22.56 23.18 25.39 29.10 34.88 38.63
43.31 44.67 47.42 52.79 55.20 57.11 64.22 65.84 67.28
70.63 76.44 78.64 81.54 83.82 85.21 92.79 99.08 101.14
104.74 110.45 112.86 115.92 122.73 132.33 140.26
143.11 144.80 152.08 158.24 161.05 171.93 177.83
185.94 188.52 194.25 198.52 199.79 211.45 221.79
225.55 234.83 239.30 245.22 249.93 253.11 259.88
261.29 267.56 270.43 275.80 281.64 288.00 295.05
297.55 310.63 315.93 325.46 328.70 343.03 346.41
350.38 356.41 367.10 368.53 377.04 385.23 396.40
419.29 419.48 420.73 428.17 437.32 444.27 452.28
478.01 483.54 494.40 500.13 502.13 516.03 519.97
531.78 542.93 554.15 557.98 563.99 565.35 566.91
570.94 603.24 608.76 617.00 618.64 632.40 632.84
640.01 641.09 647.04 647.96 667.98 702.43 703.61
722.85 727.24 729.86 734.68 737.14 743.54 757.62
772.21 778.96 786.77 795.53 801.71 805.56 806.32
816.98 819.39 822.46 827.06 830.69 839.19 846.40
859.35 861.27 865.49 873.85 876.80 894.67 900.50
907.66 925.45 925.54 938.21 947.81 956.80 961.37
973.06 980.37 986.58 991.67 998.25 1004.28 1006.45
1007.01 1009.36 1012.84 1015.18 1016.45 1022.17 1025.91
1028.02 1031.38 1034.08 1037.01 1037.56 1039.78 1042.56
1042.67 1052.13 1055.14 1055.45 1060.71 1071.15 1073.24
1086.82 1093.60 1095.35 1096.64 1106.91 1119.59 1121.15
1123.19 1125.16 1127.56 1128.11 1136.62 1141.03 1145.15
1148.05 1160.86 1163.11 1164.18 1167.55 1167.69 1182.68
1185.76 1189.45 1192.77 1194.98 1202.44 1206.39 1210.42
1231.44 1233.27 1236.58 1243.54 1269.28 1278.98 1289.03
1299.09 1311.90 1313.87 1317.42 1318.92 1319.71 1321.03
1326.26 1334.33 1340.11 1359.35 1361.56 1362.13 1363.26
1365.69 1367.90 1371.18 1380.00 1384.43 1386.71 1393.70
1393.81 1396.47 1396.52 1400.32 1400.68 1405.85 1412.83
1415.04 1428.18 1445.00 1455.20 1457.31 1457.68 1459.03
1460.28 1462.23 1463.91 1465.14 1467.04 1467.22 1467.70
1469.15 1469.68 1475.84 1476.84 1480.10 1484.42 1485.33
1486.69 1488.33 1502.02 1510.12 1526.43 1529.31 1532.12
1555.30 1584.57 1608.25 1622.88 1626.02 1632.88 1639.07
1643.26 1656.72 1660.56 1661.73 1663.55 1675.93 1688.87
1769.06 1769.88 3004.77 3023.95 3025.46 3034.57 3037.93
3040.06 3053.61 3054.83 3055.97 3061.71 3066.22 3085.70
3093.86 3094.44 3095.53 3102.91 3108.81 3119.67 3120.06
3124.86 3124.87 3128.55 3130.67 3133.33 3138.70 3140.84
3147.31 3149.95 3156.37 3161.16 3169.80 3170.15 3170.94
3172.26 3173.38 3182.26 3183.77 3185.45 3191.04 3193.03
3198.49 3202.66 3205.08 3205.57 3210.13 3210.75 3211.02
3225.15 3239.63 3239.90 3244.57 3346.94 3391.75
==============================
INT-4I
==============================
11.50 12.30 15.02 17.14 18.56 20.70 25.92 26.69
29.33 38.25 42.31 43.66 50.09 54.83 58.42 59.86 62.34
66.26 70.00 75.89 77.48 81.30 86.54 90.10 94.46 103.50
105.19 109.32 113.14 114.28 122.82 130.49 139.91
144.11 147.25 150.68 156.90 160.73 169.83 174.24
178.07 184.74 192.31 200.34 202.67 211.67 223.98
231.53 235.11 239.32 242.74 245.13 252.66 255.04
263.71 265.47 275.55 276.87 277.69 289.01 295.14
298.06 310.51 315.34 324.53 330.34 340.32 345.41
350.50 357.22 366.55 369.39 377.76 391.44 397.20
417.63 418.42 419.85 428.35 442.02 445.93 452.20
475.37 479.59 494.31 497.10 501.95 513.95 523.25
527.22 538.08 555.18 558.02 563.89 566.20 567.99
574.35 597.68 611.14 616.42 617.01 632.30 633.45
635.00 641.75 647.81 651.75 669.15 690.10 703.05
719.24 721.94 723.96 731.02 737.32 743.95 760.10
764.12 776.33 779.06 796.16 798.17 800.29 806.28
809.67 815.45 819.49 829.80 831.28 835.99 840.16
857.58 860.89 863.20 867.71 868.95 888.72 895.70
906.65 924.11 928.09 937.78 943.89 947.32 960.59
978.39 981.53 985.41 994.98 997.48 1002.95 1003.28
1004.34 1004.82 1005.84 1015.97 1016.85 1018.88 1024.36
1024.81 1030.33 1031.29 1033.90 1037.00 1037.87 1039.78
1041.58 1051.13 1052.76 1055.66 1061.84 1069.40 1074.20
1085.17 1090.76 1095.90 1099.46 1105.99 1119.10 1120.39
1123.67 1124.70 1127.41 1128.66 1137.39 1139.46 1143.68
1149.67 1162.31 1163.14 1163.76 1165.37 1168.54 1182.97
1185.97 1189.14 1189.76 1192.43 1196.03 1206.68 1209.85
1231.57 1233.49 1235.03 1236.56 1270.40 1282.81 1292.94
1299.00 1311.41 1313.18 1314.61 1317.73 1318.75 1322.16
1325.79 1328.69 1333.69 1358.01 1360.19 1362.15 1363.39
1366.95 1368.17 1377.65 1379.87 1384.22 1386.83 1392.06
1392.93 1396.27 1397.56 1398.32 1399.76 1401.01 1415.06
1420.28 1429.15 1445.53 1455.68 1457.39 1457.61 1458.53
1459.72 1459.97 1461.36 1462.68 1466.63 1467.57 1469.38
1469.60 1470.57 1477.72 1478.46 1481.32 1481.80 1485.56
1486.81 1487.29 1500.66 1512.08 1526.24 1527.49 1528.91
1554.88 1566.97 1608.14 1624.36 1626.08 1633.68 1639.56
1643.00 1658.65 1661.11 1662.40 1663.49 1675.69 1689.15
1768.38 1772.08 3004.36 3024.73 3025.07 3033.37 3036.49
3038.81 3054.95 3055.40 3058.43 3063.49 3069.77 3090.16
3091.16 3092.62 3095.99 3101.07 3107.72 3118.99 3119.34
3124.45 3124.55 3125.53 3128.89 3139.80 3140.94 3142.70
3146.75 3147.38 3159.87 3163.09 3167.75 3169.68 3174.37
3176.39 3177.48 3185.98 3187.05 3187.57 3189.88 3194.13
3194.41 3205.74 3207.17 3208.93 3209.52 3211.27 3215.48

3236.96 3241.38 3251.84 3253.77 3339.58 3460.20
===============================
INT-4J
===============================
8.78 14.86 19.95 21.34 25.05 28.49 31.58 34.74 35.99
40.68 45.74 48.72 51.91 56.62 60.50 61.79 65.03 67.46
74.65 76.73 82.01 88.94 90.25 93.12 94.92 97.19 110.31
112.11 116.72 119.42 125.48 132.93 134.12 141.98
143.71 152.18 157.04 169.14 171.68 176.28 180.91
185.48 198.93 201.56 212.34 225.07 227.84 230.67
235.67 237.85 242.47 245.66 248.52 253.29 262.39
264.25 277.42 280.67 283.53 288.82 296.58 299.09
305.11 312.09 326.94 329.76 333.16 336.21 345.39
352.19 364.96 367.33 372.01 377.42 393.92 399.84
415.65 420.40 424.80 426.86 444.82 455.21 487.07
492.97 494.21 496.27 501.02 511.41 530.87 535.36
553.03 554.58 557.47 562.58 565.43 570.34 579.35
600.37 606.21 614.80 617.32 632.33 633.80 641.30
643.79 648.08 666.41 670.50 702.43 703.30 720.35
723.22 725.74 726.50 727.49 741.81 744.94 758.23
775.40 784.97 787.58 797.28 800.22 803.44 806.41
819.00 825.84 827.60 830.15 830.68 846.43 858.38
861.57 866.59 867.92 872.07 885.04 900.18 906.99
920.15 927.53 938.50 941.66 950.05 959.63 974.02
975.07 979.20 992.80 999.46 1000.40 1000.89 1002.88
1006.00 1007.16 1016.78 1017.44 1019.67 1023.48 1025.78
1030.60 1031.31 1033.68 1035.62 1038.06 1040.38 1042.33
1051.25 1054.97 1055.67 1061.12 1069.99 1073.30 1084.96
1089.16 1091.29 1094.10 1100.73 1109.42 1117.12 1118.35
1119.43 1123.06 1127.44 1129.96 1137.41 1140.68 1141.76
1158.75 1161.66 1164.26 1168.47 1169.30 1182.86 1186.12
1189.94 1191.52 1192.67 1192.88 1197.39 1210.40 1227.26
1231.28 1234.94 1237.77 1282.70 1292.25 1297.79 1305.04
1314.16 1314.51 1317.19 1318.42 1319.41 1325.05 1329.67
1334.19 1339.46 1353.66 1357.83 1362.51 1363.44 1367.86
1370.04 1377.09 1377.70 1382.49 1383.77 1387.31 1388.72
1394.62 1395.43 1397.42 1398.74 1408.25 1415.17 1423.28
1429.69 1442.27 1456.98 1459.00 1459.27 1460.16 1461.58
1461.89 1463.70 1466.15 1467.03 1469.17 1469.43 1469.89
1474.84 1477.40 1478.11 1480.21 1483.78 1485.25 1486.84
1487.16 1501.12 1512.85 1527.32 1531.54 1531.80 1555.01
1587.34 1608.67 1625.24 1626.40 1634.75 1639.47 1644.40
1652.98 1660.97 1664.13 1667.02 1676.09 1688.64 1767.08
1779.47 3004.66 3022.52 3034.15 3035.16 3035.19 3037.29
3049.08 3050.26 3053.99 3059.63 3075.37 3077.96 3092.10
3094.16 3102.45 3108.04 3111.55 3120.10 3120.27 3122.71
3123.01 3124.05 3128.79 3129.66 3135.52 3142.14 3143.35
3147.20 3165.08 3165.73 3169.37 3169.99 3177.82 3178.04
3178.14 3182.08 3185.37 3187.04 3187.64 3189.21 3197.62
3198.74 3199.98 3204.93 3214.49 3222.25 3224.16 3226.64
3236.32 3237.89 3245.96 3313.11 3392.90
===============================
INT-4K
===============================
8.49 16.64 17.58 19.78 20.85 24.11 28.83 30.26 34.22
39.71 43.80 48.40 51.58 53.74 57.47 62.94 70.43 74.00
75.03 78.80 84.08 85.21 87.84 90.22 93.70 97.05 101.84
106.13 109.33 111.18 126.91 129.24 129.95 140.20
143.33 148.78 159.00 163.90 166.43 173.64 181.56
186.95 191.74 194.33 196.09 212.88 224.16 231.07
234.92 239.18 243.81 247.82 250.49 254.98 259.76
265.19 268.11 273.88 277.64 290.92 294.74 301.86
309.76 324.23 325.34 330.04 340.57 348.64 355.67
358.81 366.33 371.17 374.03 384.95 394.55 395.20
410.26 420.50 422.74 426.35 443.37 464.64 478.76
490.78 491.93 496.21 502.18 510.20 515.64 538.50
551.54 553.15 564.64 565.63 569.19 575.25 596.30
607.39 610.40 617.62 629.32 634.12 634.94 640.30
643.79 647.59 669.00 671.86 693.29 703.31 718.28
723.33 725.06 727.10 744.13 746.95 753.27 758.27
769.38 784.32 792.26 797.05 799.22 802.67 805.71
819.55 829.33 829.93 832.98 835.73 841.44 856.46
861.04 864.05 866.95 872.81 879.31 907.66 909.99
917.86 925.22 935.43 943.04 949.46 967.33 969.09
974.91 981.77 994.47 997.81 999.47 999.64 1000.66
1006.12 1006.71 1014.47 1016.43 1016.94 1020.68 1028.37
1029.55 1033.31 1033.99 1038.36 1039.05 1043.19 1047.53
1051.64 1055.65 1062.03 1064.32 1066.60 1074.90 1090.83
1092.43 1095.10 1098.46 1105.32 1118.84 1119.95 1123.21
1125.04 1125.54 1126.50 1127.43 1138.24 1141.13 1155.13
1157.29 1160.09 1163.97 1168.47 1169.32 1182.45 1185.27
1191.36 1192.23 1196.58 1201.85 1205.47 1210.99 1224.99
1228.89 1235.56 1237.70 1291.30 1295.54 1298.96 1313.12
1314.95 1317.08 1317.81 1320.40 1324.17 1324.87 1330.80
1333.56 1340.22 1354.85 1356.26 1363.31 1363.45 1364.52
1366.64 1366.89 1371.48 1377.66 1381.56 1384.92 1387.29
1387.42 1388.59 1396.04 1396.20 1400.77 1407.12 1415.46
1429.88 1446.43 1455.81 1457.87 1458.72 1459.18 1459.60
1459.76 1460.85 1464.29 1466.52 1467.19 1469.13 1469.42
1471.29 1475.72 1480.20 1480.65 1485.37 1486.98 1487.33
1492.59 1503.76 1514.05 1531.32 1532.47 1533.76 1554.86
1580.39 1605.19 1621.41 1624.25 1636.41 1644.12 1645.08
1662.08 1664.34 1667.31 1668.62 1675.16 1689.46 1769.29
1777.06 3005.30 3021.24 3027.07 3027.19 3034.86 3037.17
3040.97 3050.31 3054.70 3056.74 3061.54 3079.83 3091.35
3096.53 3097.97 3105.31 3108.97 3115.65 3120.17 3120.55
3121.04 3124.23 3129.87 3130.32 3141.11 3141.56 3142.31
3143.63 3147.60 3163.72 3164.59 3168.37 3173.21 3173.66
3174.16 3178.58 3182.61 3185.42 3187.09 3187.38 3190.19
3196.81 3198.48 3207.52 3209.88 3213.68 3216.11 3222.59
3226.67 3229.22 3230.08 3362.18 3381.56
===============================
(R)-4-TS
===============================
-400.54 9.10 13.85 14.15 15.29 17.68 18.82 20.15
25.32 29.89 33.23 38.87 41.96 46.67 51.55 52.60 57.48
61.28 66.95 72.87 75.91 78.17 80.32 84.00 84.87 87.51
88.26 90.36 94.28 98.51 101.00 109.62 110.45 111.73
116.83 126.82 128.84 131.04 142.54 146.14 154.35
157.93 160.44 163.13 171.71 178.80 183.77 185.54
193.56 195.57 205.94 213.18 223.31 228.73 232.54
234.43 238.95 242.80 245.83 248.85 251.54 254.28
265.45 271.43 278.12 279.19 291.56 295.33 303.34
310.18 324.14 326.37 330.84 332.10 339.84 354.89
356.99 359.88 372.00 383.15 387.51 392.87 394.24
409.86 412.56 420.40 422.75 433.20 456.01 465.58
478.63 492.33 498.16 499.59 509.72 514.79 529.07
537.68 543.89 551.01 552.58 564.78 565.07 573.78
587.59 595.69 606.86 610.53 619.45 621.64 631.12
633.97 635.67 644.22 647.35 648.12 668.76 669.68
671.73 692.19 712.69 718.40 724.32 725.26 726.53
747.11 754.33 754.81 770.36 783.87 792.40 797.43
798.58 803.01 813.92 819.37 827.01 830.12 832.61
839.68 841.40 856.17 860.99 864.73 866.22 871.39
874.47 882.67 909.13 909.99 917.09 924.82 935.42
942.32 946.31 948.07 963.46 967.43 981.93 992.43

998.05 999.89 1000.28 1001.22 1003.32 1006.05 1008.08
1016.19 1016.55 1016.99 1018.43 1019.91 1021.11 1027.54
1032.27 1032.89 1035.46 1037.24 1044.25 1047.44 1050.48
1051.28 1055.40 1060.43 1061.34 1064.10 1073.86 1090.20
1091.72 1094.09 1104.29 1108.53 1119.20 1121.91 1123.06
1124.75 1125.92 1126.98 1137.47 1140.29 1143.13 1154.10
1157.05 1158.53 1161.70 1168.80 1169.39 1178.74 1191.46
1191.63 1196.87 1201.46 1205.47 1206.30 1210.19 1225.02
1230.02 1234.66 1237.80 1291.59 1300.57 1307.46 1313.17
1315.91 1317.08 1322.32 1325.79 1331.63 1333.41 1335.45
1340.74 1346.35 1355.65 1356.43 1359.61 1363.05 1363.73
1365.70 1366.19 1366.72 1370.80 1377.61 1382.33 1385.22
1386.74 1388.87 1393.34 1396.12 1397.21 1400.13 1400.32
1408.99 1429.45 1429.51 1436.77 1445.04 1449.58 1451.56
1456.94 1459.82 1460.57 1460.68 1462.70 1463.87 1464.50
1464.82 1465.73 1467.16 1469.69 1471.55 1475.82 1476.45
1480.24 1481.68 1485.30 1485.59 1486.90 1490.68 1498.91
1501.14 1513.33 1526.44 1531.16 1532.57 1533.68 1571.17
1583.71 1604.91 1610.94 1624.94 1636.33 1644.19 1645.23
1658.54 1663.95 1667.29 1668.41 1668.67 1747.91 1772.44
1776.78 3017.84 3020.82 3031.41 3037.17 3037.40 3039.46
3043.37 3045.82 3049.31 3055.25 3060.62 3069.02 3075.20
3092.14 3097.20 3117.25 3119.19 3120.92 3120.95 3123.30
3123.44 3124.98 3128.25 3133.94 3140.76 3142.66 3144.04
3148.95 3152.04 3152.80 3159.86 3164.04 3164.63 3165.05
3169.24 3173.58 3173.67 3175.81 3178.48 3185.74 3187.60
3189.60 3197.15 3198.75 3200.69 3202.45 3210.37 3213.09
3215.85 3216.95 3223.30 3226.01 3228.67 3231.36 3356.40
3391.13

==============================

(R)-4-TS-CF3

==============================

-513.29 10.46 15.07 16.26 18.44 20.99 24.21 25.76 28.93
30.87 34.00
39.98 41.74 45.38 48.21 51.45 53.05 57.85 61.16 65.14
72.49 75.55 77.21 79.90 80.66 85.44 85.84 87.04 89.84
91.70 95.83 102.31 110.14 111.46 112.32 121.61 126.08
128.48 131.75 134.05 144.75 148.23 150.01 161.11
162.97 170.26 179.06 182.60 188.20 189.41 193.32
203.33 212.62 222.15 226.01 232.94 237.26 239.73
245.52 245.93 247.72 251.70 263.55 269.25 274.54
275.34 289.73 293.98 301.35 304.78 309.79 322.84
326.05 328.78 339.24 352.59 357.00 372.86 377.53
384.42 385.26 393.09 393.22 405.34 410.23 410.92
419.87 420.37 423.05 438.17 467.35 478.13 480.55
491.13 497.66 504.17 509.40 514.20 536.30 552.50
553.71 562.44 564.14 564.92 570.81 571.45 587.27
593.90 604.51 604.68 610.40 625.36 631.57 634.00
636.15 642.01 643.77 647.15 667.10 669.07 671.56
672.71 690.22 718.49 725.06 726.42 726.95 734.79
743.35 752.92 765.27 770.28 775.94 791.46 796.36
797.37 797.76 800.41 813.20 819.87 822.83 832.99
840.40 847.71 856.23 861.23 864.26 864.97 872.89
884.11 885.07 909.21 909.99 916.18 924.60 935.75
942.00 944.39 947.33 957.64 966.03 982.12 991.39
996.54 999.17 1001.28 1002.60 1006.40 1007.37 1009.45
1015.75 1016.43 1017.00 1017.08 1021.28 1027.84 1029.81
1033.06 1033.62 1035.64 1038.12 1047.32 1049.32 1050.98
1052.48 1054.98 1059.65 1063.94 1072.07 1088.54 1090.65
1093.03 1096.86 1104.15 1108.57 1117.83 1120.13 1123.63
1125.60 1126.06 1127.38 1134.94 1139.84 1141.36 1154.61
1156.81 1158.59 1160.76 1169.01 1169.38 1169.49 1191.83
1193.18 1196.86 1201.55 1204.91 1208.22 1225.21 1231.88
1232.38 1233.71 1237.72 1292.28 1301.48 1306.51 1312.25
1313.42 1318.45 1324.95 1330.28 1330.99 1331.82 1340.04
1347.16 1348.53 1355.69 1358.97 1359.74 1363.75 1363.92
1365.79 1367.24 1367.53 1370.46 1377.46 1378.35 1383.50
1387.07 1387.36 1394.39 1396.11 1397.35 1399.26 1401.69
1409.43 1428.06 1431.16 1435.59 1443.63 1447.87 1448.95
1451.97 1456.20 1456.76 1458.14 1460.40 1463.26 1464.38
1468.15 1469.00 1469.64 1469.67 1475.70 1480.08 1481.43
1484.79 1485.35 1486.34 1489.85 1492.51 1513.44 1527.41
1531.01 1532.59 1533.85 1555.71 1585.27 1597.60 1623.88
1631.90 1636.53 1643.91 1645.17 1658.27 1664.05 1667.33
1668.57 1679.33 1737.31 1777.10 1785.64 3024.75 3032.00
3038.21 3038.59 3041.16 3043.28 3045.66 3048.04 3056.96
3060.58 3073.50 3091.74 3094.78 3109.70 3120.01 3120.91
3121.46 3122.60 3123.35 3124.44 3128.10 3133.70 3139.38
3143.07 3145.57 3149.86 3154.61 3155.24 3164.45 3165.38
3168.21 3173.78 3174.32 3175.05 3176.15 3178.57 3185.63
3187.87 3190.24 3196.79 3197.07 3198.87 3209.07 3209.69
3212.62 3215.44 3217.73 3223.64 3223.88 3226.49 3231.00
3356.06 3427.41

==============================

(S)-4-TS

==============================

-765.11 13.97 14.22 19.01 21.08 24.05 25.02
27.47 29.69 30.35 34.48 45.28 47.04 49.99 53.13 57.27
60.30 61.51 63.94 70.54 75.58 77.15 79.80 84.90 87.07
89.45 94.53 96.61 99.15 100.31 106.07 109.57 112.18
115.72 120.43 124.63 127.72 136.24 145.81 147.58
153.41 158.26 160.71 171.31 173.23 177.01 179.11
179.53 191.79 202.17 205.60 214.74 220.60 224.99
234.08 236.35 239.16 242.26 243.41 250.36 257.18
265.17 266.84 275.78 277.10 279.51 289.32 293.61
296.51 311.50 313.51 323.08 325.05 329.27 343.18
344.07 347.83 356.61 363.40 368.34 390.24 393.28
397.33 418.02 418.73 419.71 432.33 442.79 444.33
452.93 476.19 479.89 495.47 496.62 513.01 522.74
524.28 526.87 536.80 542.40 555.55 558.22 563.58
567.01 570.60 573.13 597.62 610.38 611.42 617.35
620.56 633.19 634.31 638.01 647.81 648.35 652.59
669.19 674.94 696.50 713.54 722.37 722.84 726.28
731.36 737.30 739.44 764.29 776.34 779.03 779.65
797.40 799.68 803.34 809.77 812.33 819.69 821.31
828.36 831.86 833.22 837.34 854.47 860.68 865.77
868.40 872.33 890.30 895.87 910.54 923.93 927.62
934.75 940.03 945.98 949.82 960.30 977.64 986.02
991.19 997.70 1001.84 1003.60 1005.02 1005.13 1005.39
1007.99 1014.03 1016.83 1017.34 1019.43 1020.69 1023.86
1024.78 1030.63 1032.05 1033.60 1037.98 1038.28 1043.92
1049.67 1051.52 1053.02 1057.35 1060.07 1062.01 1074.49
1083.85 1090.75 1096.71 1101.51 1103.34 1120.00 1123.45
1124.08 1124.23 1128.88 1137.00 1140.07 1142.29 1142.79
1149.26 1160.62 1161.07 1162.33 1165.56 1168.42 1181.14
1189.70 1190.14 1191.10 1196.99 1206.90 1209.78 1209.83
1230.27 1233.39 1234.73 1236.62 1270.69 1283.18 1291.80
1307.73 1311.70 1313.23 1318.12 1321.24 1326.17 1329.16
1333.70 1336.88 1345.99 1357.62 1358.57 1360.44 1362.46
1363.10 1366.84 1367.21 1378.94 1382.38 1383.98 1391.55
1392.39 1393.33 1396.35 1397.64 1398.76 1400.16 1400.84
1402.51 1421.17 1429.38 1431.28 1438.62 1443.05 1450.17
1451.74 1455.75 1457.04 1457.49 1459.85 1462.29 1462.81
1464.12 1465.90 1467.72 1469.17 1471.22 1474.23 1475.91
1477.49 1479.73 1481.46 1481.89 1483.38 1484.04 1487.25
1499.18 1500.84 1511.12 1522.41 1525.58 1526.38 1528.90

1567.36 1572.33 1607.64 1614.37 1625.44 1633.83 1637.81
1642.92 1656.07 1661.18 1662.19 1663.27 1669.56 1729.30
1768.34 1771.56 3014.46 3025.18 3035.96 3037.34 3039.59
3039.63 3048.41 3059.28 3061.99 3062.05 3066.53 3076.70
3089.78 3090.79 3092.96 3113.17 3116.83 3119.87 3120.48
3122.13 3125.28 3125.95 3126.04 3137.37 3145.55 3146.75
3150.57 3154.14 3155.90 3160.68 3162.16 3162.91 3163.42
3170.61 3173.35 3174.94 3177.33 3184.03 3184.19 3189.68
3194.02 3202.45 3204.86 3207.46 3208.88 3210.34 3211.12
3215.71 3216.58 3228.77 3240.94 3241.64 3251.16 3252.39
3357.72 3460.80

==============================

(R)-INT-6

==============================

8.52 11.55 17.35 20.22 20.87 22.91 24.14 27.33 30.70
35.80 37.93 40.81 43.71 50.61 51.66 55.33 56.48 60.87
62.41 64.17 72.27 75.68 78.82 81.73 82.37 85.87 88.02
91.48 97.44 100.50 108.29 111.43 115.44 126.17 129.98
133.44 137.92 138.60 146.49 152.20 159.88 164.84
172.32 181.53 187.61 193.55 195.80 199.47 206.95
212.19 224.74 229.20 236.62 238.10 241.81 252.78
253.72 262.58 265.22 267.07 270.77 278.40 279.44
280.70 295.12 300.05 302.59 309.64 318.14 322.74
323.25 323.69 336.06 343.98 354.20 360.99 363.66
368.81 379.89 391.41 394.01 401.02 414.13 414.35
419.84 420.68 437.51 460.37 467.76 476.59 479.10
489.01 497.71 510.32 515.37 538.74 540.21 552.55
553.86 559.79 563.63 566.15 580.90 592.77 596.48
597.76 611.99 616.55 630.99 631.79 634.35 639.08
640.99 646.24 647.66 668.35 668.94 671.32 693.45
718.43 725.81 727.70 727.91 748.53 752.66 754.31
767.86 775.76 784.40 791.38 797.50 799.02 802.68
816.78 817.01 819.56 824.38 834.85 841.05 849.86
857.84 861.24 862.75 865.59 874.28 882.87 886.27
908.75 916.55 921.96 925.70 936.33 944.42 951.50
966.54 972.65 975.92 977.97 982.29 986.33 995.04
997.28 1000.42 1002.63 1002.84 1009.60 1014.18 1014.94
1015.23 1016.77 1018.71 1020.62 1021.10 1028.08 1034.19
1035.12 1037.73 1047.76 1049.54 1051.00 1053.23 1060.20
1062.91 1064.75 1067.80 1077.71 1092.69 1094.21 1097.42
1102.68 1104.68 1120.56 1120.82 1122.97 1124.73 1125.57
1128.64 1131.68 1137.23 1138.18 1139.96 1156.02 1157.58
1161.27 1167.81 1168.63 1169.05 1176.22 1183.49 1190.76
1194.72 1195.62 1196.45 1204.75 1206.33 1211.98 1223.33
1226.69 1237.45 1239.09 1257.31 1281.49 1292.75 1294.16
1303.44 1310.33 1312.76 1317.71 1320.82 1322.52 1324.19
1324.78 1329.84 1334.39 1351.09 1353.02 1353.27 1362.33
1363.39 1364.87 1366.56 1367.99 1375.86 1378.14 1381.94
1382.78 1385.62 1390.65 1391.00 1396.01 1396.18 1396.73
1399.76 1407.48 1422.62 1429.70 1436.45 1446.65 1454.13
1458.36 1458.75 1459.02 1460.29 1462.02 1464.03 1466.92
1467.23 1468.25 1473.20 1474.80 1475.65 1476.60 1479.37
1483.98 1485.04 1487.15 1488.29 1489.34 1503.44 1507.64
1514.01 1529.68 1531.63 1532.13 1559.31 1589.15 1608.30
1626.35 1633.13 1636.89 1644.96 1645.25 1656.79 1664.65
1665.64 1669.13 1679.45 1770.03 1778.51 1848.62 3003.47
3020.20 3021.89 3028.06 3037.49 3037.87 3040.57 3043.24
3049.51 3055.37 3058.79 3059.69 3067.46 3085.12 3086.09
3091.74 3094.79 3115.80 3121.27 3123.68 3124.19 3124.66
3128.18 3129.02 3130.50 3139.73 3142.22 3143.70 3144.53
3144.91 3147.70 3164.02 3166.38 3166.41 3167.86 3171.66
3172.42 3174.08 3176.41 3179.80 3187.29 3187.88 3190.55
3192.04 3196.06 3199.65 3204.72 3213.02 3214.72 3217.14
3223.36 3225.90 3229.09 3231.86 3244.62 3341.97 3402.10

==============================

(R)-INT-6-CF3

==============================

9.58 13.75 17.44 18.77 21.95 27.50 29.92 31.79 33.58
34.29 39.13 41.50 43.96 47.49 49.92 53.15 54.28 57.99
61.69 63.89 71.77 74.80 76.18 78.38 84.78 85.87 90.64
91.42 92.46 97.92 99.81 102.99 104.79 109.86 122.12
126.58 133.35 139.43 147.26 151.30 152.89 160.73
167.99 170.88 176.26 180.05 189.52 192.02 197.15
212.42 214.46 223.54 231.72 232.95 237.62 243.74
246.99 251.62 256.15 263.41 266.57 268.03 277.27
283.37 294.07 300.04 307.39 311.46 318.51 323.20
324.68 328.87 339.37 341.87 348.31 352.49 359.99
372.87 381.19 383.66 391.15 392.64 395.21 410.47
415.64 419.74 421.93 428.20 439.39 467.51 480.74
498.98 499.41 500.06 509.81 514.69 530.69 537.80
552.09 552.98 555.90 563.30 565.27 571.00 583.04
585.43 593.21 605.29 607.22 610.77 619.75 632.54
633.98 636.65 644.70 645.55 647.54 654.19 668.67
671.53 692.19 706.24 718.10 722.70 726.67 728.08
748.12 755.47 765.67 770.77 781.75 782.32 792.80
798.12 798.60 800.85 804.22 817.06 818.49 820.74
831.85 844.51 855.76 860.61 864.30 867.06 867.35
883.84 885.30 891.22 911.57 919.72 920.05 925.06
935.76 940.09 948.58 967.49 969.02 981.21 981.83
989.67 996.07 998.22 999.84 1001.56 1003.31 1003.90
1014.55 1016.29 1016.97 1017.18 1020.97 1024.87 1028.63
1030.22 1033.05 1035.05 1039.03 1043.97 1047.17 1049.58
1051.70 1056.24 1059.45 1060.56 1063.80 1072.79 1089.68
1092.05 1093.85 1095.79 1104.20 1107.18 1119.20 1121.74
1124.20 1125.76 1126.32 1126.82 1127.55 1136.38 1139.20
1140.55 1155.17 1156.46 1156.82 1159.75 1163.94 1168.90
1169.63 1179.22 1191.81 1196.62 1200.77 1202.37 1206.57
1207.48 1224.06 1225.04 1228.71 1232.75 1237.56 1256.07
1275.67 1293.50 1302.25 1304.55 1312.25 1313.30 1320.48
1326.38 1328.78 1332.25 1335.40 1342.41 1348.84 1356.94
1359.31 1360.10 1363.21 1364.15 1365.52 1368.04 1373.30
1377.55 1377.61 1382.23 1383.75 1384.66 1386.30 1387.97
1393.37 1395.17 1397.60 1399.38 1410.74 1425.85 1431.21
1437.29 1444.44 1444.89 1454.72 1455.72 1458.26 1458.51
1459.51 1461.16 1466.85 1467.93 1468.79 1473.53 1473.80
1474.27 1480.93 1483.54 1485.70 1486.20 1489.45 1500.58
1513.77 1531.63 1532.46 1533.80 1553.93 1589.37 1598.17
1623.98 1636.95 1641.15 1644.00 1645.15 1656.34 1664.95
1667.70 1668.37 1683.79 1772.97 1777.96 1850.98 3016.81
3023.16 3026.80 3037.85 3038.21 3040.45 3048.88 3054.43
3054.74 3056.69 3058.62 3066.34 3089.83 3089.99 3115.72
3118.12 3120.39 3121.25 3123.40 3126.12 3128.71 3129.72
3134.72 3135.26 3138.68 3143.66 3144.60 3146.05 3164.48
3164.55 3168.22 3172.04 3173.88 3174.01 3174.74 3177.80
3179.43 3184.86 3187.89 3191.50 3196.52 3198.82 3199.23
3209.14 3211.14 3211.39 3218.35 3224.84 3225.39 3225.42
3233.29 3246.36 3346.38 3384.52

==============================

(S)-INT-6

==============================

2.66 15.83 16.64 22.68 24.53 28.13 32.40 36.08 38.40
41.53 45.09 45.98 50.88 52.24 56.75 59.44 62.48 64.09
70.69 71.99 75.92 77.89 81.45 82.76 86.37 93.84 96.79
99.68 103.16 105.00 106.34 111.46 115.33 123.97 127.06
129.81 137.78 140.35 142.97 147.00 149.80 158.35
166.36 171.36 180.66 187.93 195.08 202.45 214.80

224.25 225.41 226.92 230.02 232.33 245.84 247.69
252.09 255.88 260.38 263.08 268.05 270.25 272.93
279.35 283.38 292.45 297.84 298.88 303.60 314.11
315.71 330.21 332.58 340.57 355.09 364.41 370.09
374.21 376.98 388.55 394.54 415.66 417.28 418.43
420.75 423.86 432.39 441.11 455.17 464.57 480.93
496.00 500.25 504.99 522.93 526.21 529.18 535.42
552.61 556.47 562.76 566.15 578.42 590.05 592.71
598.25 612.90 618.82 622.65 626.27 634.24 634.28
647.48 648.15 649.66 671.18 688.52 698.10 720.02
724.00 727.02 728.97 736.67 749.31 751.70 759.48
763.07 769.97 785.54 791.41 793.90 800.69 814.77
816.24 820.36 821.46 825.67 831.62 834.28 836.40
855.83 859.36 862.78 866.97 876.14 894.42 897.59
904.48 908.79 925.24 928.88 940.81 946.03 953.18
960.54 967.17 973.99 975.51 983.33 989.28 996.33
999.07 1003.16 1003.77 1004.21 1009.42 1009.70 1014.88
1015.39 1016.89 1020.39 1024.80 1025.58 1025.97 1027.63
1030.64 1034.04 1036.70 1042.43 1048.71 1049.78 1053.34
1053.84 1055.14 1067.80 1079.88 1088.15 1089.48 1099.64
1101.41 1102.51 1103.33 1110.31 1114.38 1120.13 1124.43
1126.92 1128.48 1132.98 1134.80 1142.06 1143.25 1163.86
1167.87 1168.08 1169.51 1171.00 1179.39 1184.58 1185.90
1187.55 1191.15 1192.54 1195.82 1204.13 1209.70 1228.31
1236.19 1238.31 1240.41 1247.23 1268.51 1279.40 1284.46
1300.77 1310.76 1311.44 1311.60 1313.22 1315.99 1319.69
1327.24 1331.95 1335.12 1336.47 1346.58 1359.13 1364.36
1365.23 1366.01 1368.67 1375.33 1378.98 1380.27 1382.39
1387.64 1390.04 1393.51 1394.91 1396.35 1396.82 1403.56
1407.24 1420.97 1421.62 1432.05 1436.23 1446.10 1453.77
1456.65 1456.85 1457.22 1460.98 1461.30 1462.77 1466.05
1469.07 1469.42 1470.49 1472.08 1473.53 1474.27 1479.00
1480.52 1483.38 1483.88 1485.86 1487.38 1502.41 1506.22
1512.70 1528.53 1528.80 1531.02 1556.51 1598.84 1608.43
1626.77 1634.62 1636.51 1642.90 1644.04 1651.73 1661.98
1664.25 1666.03 1678.82 1777.05 1777.88 1863.53 3004.39
3026.65 3033.62 3035.06 3037.38 3038.39 3043.65 3048.15
3055.36 3055.61 3059.52 3063.49 3083.30 3092.34 3096.79
3097.46 3105.48 3117.31 3122.22 3123.33 3123.44 3124.40
3128.19 3128.75 3132.92 3139.10 3141.13 3141.63 3142.26
3142.47 3149.67 3159.88 3160.63 3163.56 3168.47 3172.43
3172.79 3175.23 3175.62 3179.99 3181.97 3186.01 3188.01
3190.12 3197.51 3198.54 3206.57 3206.85 3209.23 3209.36
3215.61 3224.36 3224.74 3227.90 3237.66 3335.48 3398.76

===============================

(R)-6-TS

===============================

-304.88 4.55 12.46
13.35 15.17 21.85 25.14 27.88 29.51 31.28 33.04 38.25
38.90 39.97 47.19 51.68 54.92 57.51 61.74 63.55 66.82
73.88 79.50 79.54 81.52 84.77 88.14 93.91 94.91 100.79
104.46 108.49 118.82 123.73 125.45 130.00 135.14
136.53 137.82 142.42 147.59 149.25 152.05 169.08
176.00 178.28 180.46 184.26 187.84 195.20 209.98
214.16 220.44 224.26 226.70 231.28 236.22 241.53
250.42 251.06 251.38 261.49 266.45 274.20 274.85
285.16 287.59 290.91 294.69 309.61 319.58 325.13
328.21 328.63 343.21 345.29 355.42 362.22 363.85
371.74 388.74 390.85 397.56 415.48 420.06 420.53
423.30 430.49 463.95 474.19 481.07 483.76 493.70
497.19 511.13 517.09 519.07 533.06 556.18 556.81
563.69 570.06 571.12 580.06 595.99 600.26 613.06
615.30 631.92 632.47 634.18 636.65 640.92 645.67
648.51 655.91 668.35 670.54 682.03 693.91 717.48
727.49 730.20 731.66 732.29 748.42 757.86 767.11
785.53 791.65 795.65 798.92 805.55 819.32 820.56
829.43 836.39 837.29 843.77 848.25 857.03 859.83
861.17 864.43 876.92 877.45 885.40 898.48 909.90
910.03 921.11 926.18 930.31 934.94 944.62 953.63
967.65 982.58 987.93 989.06 996.21 997.78 999.27
1002.34 1003.23 1005.36 1011.22 1014.68 1016.22 1017.51
1019.22 1019.72 1020.01 1029.98 1034.30 1036.77 1039.01
1041.49 1044.50 1048.06 1050.33 1053.37 1053.79 1063.19
1065.16 1066.11 1076.70 1076.90 1093.15 1097.33 1102.36
1120.15 1121.37 1125.57 1126.65 1128.52 1129.31 1131.18
1134.13 1134.62 1151.30 1158.12 1158.63 1159.22 1165.24
1167.39 1167.67 1167.86 1170.18 1187.95 1192.17 1195.80
1197.71 1200.81 1201.25 1202.66 1204.88 1231.20 1234.31
1236.20 1252.23 1283.79 1292.33 1299.95 1303.17 1309.11
1311.00 1311.56 1312.55 1321.06 1327.50 1329.90 1330.84
1346.60 1353.49 1355.18 1355.31 1363.65 1365.17 1366.90
1369.02 1378.39 1379.69 1381.15 1386.39 1390.26 1391.92
1393.36 1394.63 1395.29 1396.03 1404.18 1415.96 1416.89
1429.04 1432.83 1441.05 1445.60 1447.41 1455.40 1457.66
1458.75 1460.35 1463.16 1466.83 1467.39 1468.17 1470.88
1471.60 1472.23 1473.46 1476.54 1478.47 1481.50 1487.36
1489.67 1490.50 1491.76 1497.19 1503.88 1513.56 1529.85
1531.13 1532.04 1536.98 1584.77 1597.32 1619.54 1628.32
1635.84 1644.24 1645.02 1653.58 1661.89 1664.59 1665.14
1668.92 1767.94 1777.09 1866.52 3017.61 3021.46 3024.48
3030.98 3033.52 3038.43 3040.07 3044.14 3047.37 3050.88
3052.08 3053.42 3058.49 3084.17 3086.56 3107.52 3115.28
3116.63 3119.37 3121.62 3123.46 3126.60 3131.26 3134.99
3140.82 3141.58 3142.97 3143.54 3159.90 3161.32 3162.85
3163.87 3164.21 3166.09 3166.87 3169.54 3170.03 3177.55
3181.31 3183.97 3185.18 3185.26 3187.27 3194.88 3195.61
3205.54 3207.16 3213.89 3216.28 3219.13 3220.23 3223.45
3227.84 3231.14 3246.97 3287.62 3316.10

===============================

(R)-6-TS-CF3

===============================

-457.82 5.51 11.90 13.79 14.50 18.81 22.77 25.34
26.44 29.88 30.33 33.11 38.60 39.70 40.72 46.75 50.09
55.25 56.72 61.09 63.13 67.37 74.21 80.29 80.79 84.21
87.15 89.52 93.01 95.05 100.52 103.66 113.13 119.44
120.34 123.85 127.99 131.83 138.11 140.61 143.72
148.45 151.55 152.36 168.22 169.42 176.52 180.11
187.13 190.06 195.26 209.03 213.39 220.09 224.27
229.56 231.75 234.41 235.83 250.70 251.26 260.59
264.60 273.31 275.15 286.05 290.41 291.56 295.74
309.85 320.01 324.86 328.09 328.31 344.17 345.11
355.24 359.96 361.73 371.65 389.04 391.30 398.24
403.17 410.34 415.46 415.74 419.94 420.51 434.77
464.40 473.97 484.02 486.76 491.54 492.91 510.81
516.26 532.93 556.00 556.78 562.53 564.18 571.02
579.45 585.23 586.24 594.87 612.96 615.10 618.72
631.49 632.17 634.18 635.75 640.74 646.58 648.52
659.91 668.64 670.52 686.03 693.63 717.66 727.47
729.84 732.49 740.78 748.62 758.13 767.16 785.78
789.17 791.88 796.34 799.15 806.18 815.52 819.33
836.50 839.20 845.91 857.22 858.53 859.40 861.17
864.82 876.97 877.47 887.15 895.88 905.28 909.52
920.89 926.00 928.57 935.28 944.43 953.64 967.09
982.54 990.82 991.25 996.46 998.30 998.92 1002.62
1005.27 1009.57 1011.51 1014.68 1016.46 1018.76 1019.28
1020.08 1020.19 1028.33 1030.62 1034.15 1036.43 1039.66

1040.88 1048.05 1050.14 1053.28 1053.66 1062.75 1066.01
1074.94 1077.15 1093.07 1096.02 1097.28 1102.11 1119.46
1121.54 1125.10 1126.30 1127.92 1128.95 1131.66 1131.85
1134.15 1148.97 1157.52 1158.26 1160.82 1166.39 1167.76
1168.11 1168.34 1188.18 1191.89 1195.92 1197.58 1199.31
1201.60 1202.90 1207.51 1228.51 1231.05 1234.31 1236.35
1267.34 1283.99 1293.00 1304.50 1309.73 1311.15 1312.65
1314.49 1317.17 1320.48 1327.52 1330.04 1331.02 1342.55
1354.59 1355.32 1355.69 1363.56 1364.92 1366.78 1368.73
1378.82 1380.26 1382.39 1386.78 1390.50 1393.30 1394.08
1394.83 1396.32 1397.05 1402.99 1416.69 1417.47 1428.78
1432.10 1441.00 1441.94 1446.81 1449.28 1455.80 1457.81
1459.95 1460.33 1465.74 1467.27 1468.17 1471.60 1473.43
1476.49 1478.50 1481.40 1487.48 1488.68 1489.14 1495.11
1503.99 1512.84 1529.77 1531.14 1532.18 1545.85 1586.96
1619.79 1622.23 1628.32 1635.85 1644.36 1645.06 1652.40
1659.66 1664.52 1665.14 1669.11 1768.69 1775.51 1878.87
3017.01 3023.87 3030.68 3034.60 3039.37 3041.65 3046.74
3049.75 3051.32 3052.35 3057.84 3066.20 3084.58 3087.54
3114.39 3117.42 3119.96 3126.44 3127.36 3132.09 3133.85
3135.27 3140.56 3144.03 3144.21 3148.99 3160.66 3163.46
3163.69 3163.73 3164.03 3167.56 3170.74 3173.88 3178.09
3182.15 3185.57 3185.84 3186.44 3188.00 3195.64 3195.72
3205.75 3208.20 3209.93 3214.09 3220.66 3222.49 3223.72
3223.97 3226.86 3244.09 3283.56 3310.31

==============================

(S)-6-TS

==============================

-229.12 7.80 12.91 17.28 19.52 24.18 26.64 28.35
30.41 34.03 37.24 39.35 40.57 42.53 43.72 45.76 49.61
56.40 57.60 57.80 60.26 62.02 63.90 64.48 70.87 75.02
84.20 87.35 91.73 93.76 98.07 99.09 101.24 106.11
110.68 115.53 120.90 124.43 134.28 138.56 145.10
149.61 152.11 155.79 159.88 171.16 184.43 185.21
190.82 191.88 198.08 213.13 217.95 220.38 224.13
229.64 233.69 235.96 250.22 252.74 254.97 257.84
259.43 264.97 270.29 278.12 283.15 291.70 302.57
309.68 311.54 317.86 325.49 328.45 329.30 342.36
352.06 354.92 368.18 376.43 381.75 388.86 396.46
419.51 421.01 422.85 427.10 430.34 442.71 471.38
481.33 483.74 498.28 505.66 514.26 522.73 530.24
532.68 550.51 554.79 562.24 566.30 568.86 580.48
587.78 598.78 608.49 618.48 622.01 630.41 633.37
633.69 636.11 648.63 652.12 659.30 668.18 692.08
699.32 723.05 727.02 730.77 732.83 734.10 736.17
741.05 757.54 775.58 786.09 787.55 793.62 795.11
796.94 817.96 818.36 823.47 825.27 827.86 833.76
854.85 859.84 861.78 869.39 874.35 885.18 887.29
891.35 901.51 905.14 918.00 927.68 930.29 939.71
944.92 951.65 957.74 962.31 966.16 982.41 987.35
994.48 995.92 998.88 1002.03 1003.95 1009.64 1013.81
1014.73 1015.18 1015.25 1017.95 1022.26 1026.44 1033.81
1035.58 1037.49 1038.56 1043.73 1049.82 1051.51 1051.75
1052.13 1054.39 1057.24 1061.08 1069.72 1073.27 1091.92
1096.81 1097.49 1101.26 1103.68 1109.62 1116.99 1121.90
1129.47 1131.87 1132.32 1136.09 1139.54 1140.46 1144.48
1158.95 1161.98 1166.76 1167.02 1167.54 1168.06 1169.53
1180.23 1188.57 1189.23 1195.97 1198.21 1201.06 1201.66
1204.12 1234.01 1235.96 1236.67 1257.14 1265.97 1300.86
1304.91 1305.96 1307.38 1310.61 1311.84 1312.60 1316.47
1319.93 1324.30 1330.11 1347.63 1349.37 1360.73 1361.26
1364.84 1365.20 1367.69 1370.31 1375.95 1383.20 1386.89
1386.97 1392.13 1393.72 1394.17 1394.78 1395.30 1396.58
1398.54 1412.52 1428.06 1428.26 1433.85 1435.62 1436.02
1444.20 1456.22 1458.06 1458.72 1460.17 1460.92 1461.92
1464.93 1465.45 1470.20 1470.44 1472.03 1472.40 1474.32
1475.20 1479.43 1483.25 1485.61 1488.11 1490.85 1491.78
1499.57 1504.58 1529.11 1529.43 1530.66 1540.20 1586.53
1600.88 1624.32 1626.77 1634.02 1641.75 1644.28 1645.40
1661.66 1663.37 1664.93 1665.30 1751.33 1793.07 1863.37
3019.64 3020.31 3029.85 3032.92 3033.39 3035.61 3048.82
3051.08 3051.38 3056.06 3061.24 3072.62 3075.45 3076.75
3089.30 3090.27 3105.13 3114.10 3119.16 3122.10 3123.09
3123.46 3129.35 3129.94 3132.36 3132.60 3133.51 3143.34
3152.20 3157.30 3161.33 3163.94 3168.54 3169.20 3170.92
3173.59 3178.42 3180.53 3180.75 3181.92 3182.86 3184.94
3191.92 3193.80 3201.04 3203.04 3203.29 3205.02 3210.25
3213.04 3214.30 3219.24 3225.53 3227.45 3232.50 3236.58
3438.21

==============================

(R)-INT-7

==============================

21.70 29.19 35.14 58.13 67.47 82.56 97.41 109.04
131.89 150.55 160.64 172.66 187.20 217.17 234.85
249.46 266.23 280.74 298.65 347.69 355.68 389.14
426.23 439.55 502.70 527.90 545.99 548.59 576.05
634.05 647.84 682.56 724.55 739.41 800.99 815.10
828.97 832.81 841.94 862.74 922.20 974.67 988.44
994.64 1000.31 1007.86 1020.08 1044.38 1066.92 1074.37
1103.04 1107.90 1124.23 1144.07 1162.88 1166.21 1185.33
1200.70 1201.73 1254.62 1289.10 1321.54 1328.04 1338.28
1366.10 1377.38 1383.81 1398.06 1406.13 1421.96 1428.83
1434.61 1453.52 1462.98 1465.53 1467.60 1470.11 1471.90
1474.76 1476.95 1480.00 1549.74 1587.81 1617.11 1663.75
1960.09 3013.84 3049.82 3052.50 3052.77 3082.12 3096.04
3096.90 3133.19 3138.16 3138.97 3146.35 3153.32 3164.48
3170.20 3178.90 3188.41 3196.50 3212.26 3226.02

==============================

(R)-INT-7-CF3

==============================

17.35 21.39 28.34 35.66 57.48 67.61 83.45 101.21
115.46 136.57 144.20 154.05 169.87 194.34 226.47
243.95 249.54 272.53 291.88 320.22 354.40 391.50
402.75 415.32 429.05 478.79 512.61 544.03 556.96
584.66 603.24 613.77 627.47 645.56 691.38 744.38
760.22 801.12 809.69 822.93 841.03 856.50 871.37
926.75 983.98 995.05 998.61 1009.96 1019.02 1027.42
1043.59 1071.88 1096.35 1102.04 1106.05 1124.11 1141.46
1160.47 1193.25 1200.41 1207.07 1232.65 1260.25 1288.82
1327.06 1332.50 1345.28 1366.24 1378.12 1379.66 1396.59
1405.55 1425.47 1430.28 1432.61 1448.25 1452.63 1464.41
1470.29 1473.78 1476.03 1542.99 1601.22 1637.00 1672.73
1980.33 3050.69 3051.71 3053.36 3081.23 3094.25 3132.66
3139.75 3139.92 3148.16 3156.22 3171.83 3186.16 3187.71
3196.94 3213.71 3216.12

==============================

TsOH

==============================

25.37 39.22 83.97 164.29 188.96 204.37 286.23 326.47
347.89 386.53 420.51 457.91 471.34 538.87 549.89
645.75 655.89 729.46 786.31 827.09 836.69 859.44
989.29 998.58 1008.00 1029.73 1053.64 1101.83 1112.14
1128.19 1171.54 1193.27 1237.29 1313.24 1361.55 1384.59
1396.52 1430.59 1456.82 1468.01 1526.90 1634.48 1659.62
3027.86 3096.75 3128.98 3177.36 3180.43 3211.24 3212.73
3763.88

================================
TsO-
================================
15.33 28.68 85.98 157.39 205.08 268.38 313.82 352.98 384.66 418.63 481.21 530.09 552.36 570.68 650.16 657.93 732.59 811.89 833.65 856.82 978.68 991.02 998.06 1001.21 1033.30 1047.97 1108.52 1113.78 1174.96 1223.50 1226.45 1231.83 1301.51 1358.37 1389.01 1423.19 1457.01 1465.47 1521.77 1627.72 1656.33 3000.39 3065.16 3098.25 3136.38 3137.73 3194.10 3194.75
================================
AcOH
================================
83.15 424.73 544.31 593.76 683.33 874.55 992.36 1054.56 1215.23 1336.89 1407.98 1439.59 1447.93 1869.91 3049.82 3126.80 3179.05 3743.17
================================
Alkyne
================================
10.84 58.79 78.23 106.96 191.12 238.19 253.53 308.18 328.93 352.59 427.49 478.11 490.33 548.21 564.81 642.51 661.68 748.81 822.17 831.01 863.71 977.10 993.13 994.99 1023.76 1033.22 1035.81 1093.01 1121.47 1171.01 1182.54 1203.43 1296.72 1307.85 1318.45 1352.09 1397.61 1444.42 1446.03 1455.04 1465.77 1468.75 1480.98 1553.83 1614.37 1674.27 2342.83 2993.05 3013.86 3064.02 3085.95 3092.57 3138.28 3190.28 3192.19 3205.56 3216.13
================================
EtOH
================================
258.26 295.56 419.17 820.66 912.34 1039.57 1129.94 1177.12 1264.01 1290.77 1384.31 1452.32 1457.05 1476.80 1512.23 2950.31 2978.84 3032.73 3119.86 3120.83 3822.76
================================
Ph*I
================================
11.08 13.51 16.86 24.19 31.55 38.71 43.52 53.63 59.71 60.19 70.44 78.41 84.67 101.27 105.69 120.68 136.39 141.49 145.59 171.85 179.63 202.04 204.35 213.26 228.47 235.64 236.56 252.24 256.84 259.10 263.96 273.19 294.65 307.49 324.76 340.78 341.42 350.52 411.31 416.13 419.26 420.30 449.24 475.63 505.66 514.91 534.81 539.33 562.58 569.67 578.64 583.65 592.01 597.89 607.26 620.13 633.96 635.12 638.68 648.05 678.01 717.96 722.12 726.42 752.73 769.57 777.35 787.27 788.18 790.02 798.95 813.26 858.01 859.84 864.67 870.24 901.94 914.42 921.64 938.36 941.51 950.46 969.32 987.35 997.62 1002.29 1014.89 1015.46 1028.41 1029.16 1031.11 1034.95 1037.77 1042.23 1053.21 1054.57 1061.58 1082.08 1088.86 1090.71 1113.28 1116.35 1121.91 1128.51 1129.52 1137.12 1150.36 1158.71 1166.30 1170.18 1171.52 1191.60 1193.59 1202.48 1222.31 1229.13 1246.32 1262.40 1270.90 1311.21 1316.38 1323.71 1327.77 1344.66 1350.95 1358.90 1361.14 1366.33 1369.69 1373.93 1378.44 1382.36 1385.16 1388.74 1392.27 1394.04 1400.27 1458.55 1460.09 1460.38 1461.53 1462.58 1464.14 1470.60 1477.74 1483.95 1486.73 1488.56 1500.09 1529.92 1530.65 1547.37 1552.09 1625.80 1643.33 1644.39 1654.29 1666.54 1667.34 1784.72 1800.07 3030.69 3038.11 3042.36 3044.91 3048.00 3058.04 3068.71 3094.72 3118.09 3121.04 3126.11 3130.78 3143.14 3143.89 3151.73 3159.84 3168.64 3168.89 3171.16 3174.08 3181.81 3183.20 3188.97 3190.12 3192.74 3197.98 3220.94 3225.71 3248.06 3572.76 3588.22

Cartesian Coordinates of Optimised Structures

80
INT-1
C 2.97585534923675 3.57964399191416 -
2.02694520371103
C 3.19737602788377 2.55188642221055 -
0.93302872605654
C 4.12640031556775 1.40561618821885 -
1.39286347144498
N 3.93598814794217 0.25111954529566 -
0.72409244415148
C 4.65942921289868 -0.97903268921828 -
1.01961945079531
C 5.76793659169067 -1.21748584721754
0.00626010344640
C 3.63344227507714 -2.10125270887194 -
1.13979205706394
C 2.67792452313452 -2.02596034581584 -
2.16573830621423
C 1.66976540201828 -2.98250585563918 -
2.28037832898453
C 1.59550249332377 -4.03608450172050 -
1.36220458386407
C 2.54342349577401 -4.12434447245435 -
0.34184494910153
C 3.55604516848444 -3.16483555185168 -
0.23390038507443
O 4.95930722623996 1.58692497688226 -
2.26922322878914
O 1.96365211314117 1.94282614860688 -
0.54232871737968
C 1.21283328379630 2.37988996820256
0.47712687702422
C 1.40343663511717 3.58127608646526
1.17652297216622
C 0.53359944057688 3.90597011372874
2.21905397528481
C -0.52336331057822 3.07691085027142
2.59761446585627
C -0.72928332915336 1.87521836099296
1.90281817342988
O -1.68644519576543 0.97570934034412
2.16752839674471
C -2.81154575165779 1.24793847508501
3.01311067072976
C -2.52594991316576 0.87546719156319
4.45429743687362
C -3.95448762566828 0.39717467108089
2.41113093460727
N -4.10180837119077 0.56911014342977
1.07913743743358
C -4.89825053199104 -0.33642531461394
0.25355793441481
C -5.81625487069562 0.46008046603325 -
0.67103736040211
C -3.93889448078962 -1.29300984017123 -
0.45036744903855
C -3.58782503514392 -1.15750845552437 -
1.79888475593141
C -2.61853898001610 -1.98720010111959 -
2.37266240627418
C -1.99198669031343 -2.97082260743154 -
1.60327610966072
C -2.33851618622376 -3.11875549062818 -
0.25475501131620
C -3.30293721965860 -2.28636411768426
0.31321517761787
O -4.62565528904120 -0.35193058187264
3.10506431021929
C 0.13965356106373 1.55630606320633
0.84907549595669
I -0.22546726331738 -0.19277404789430 -
0.25341596264181
O 0.28988210777667 -1.40856963684519
1.45712060980645
C 1.51570207240231 -1.34324391806600
1.92300332624305
C 1.76329171816770 -2.33606760832970
3.03898902699581
O 2.38233840465646 -0.58681424402576
1.51050855315827
O -0.71991504108767 1.06923483126669 -
1.95675841623570
C -1.80065386179911 1.80611963522526 -
1.88924415774796
C -2.08558583088319 2.54182643827604 -
3.18361683308912
O -2.53143976794156 1.89733307367954 -
0.91187910608579
H 2.39298671501936 4.43516449272811 -
1.65371301238286
H 3.94821548706568 3.93014416153601 -
2.39842428501470
H 2.43202353320619 3.11900245901088 -
2.86527595358562
H 3.67247940940203 3.01236427115585 -
0.04791576408158
H 3.22029255603357 0.18819170699799
0.00127623808732
H 5.12464886855287 -0.82132167639235 -
2.00599848817925
H 6.48278627180742 -0.38208988327522 -
0.03005585667319
H 6.31685947906393 -2.14942140088495 -
0.20213280079601
H 5.35352545820071 -1.26876658689870
1.02515711896380
H 2.72118534405701 -1.19408778718335 -
2.87414139662732
H 0.93937200252930 -2.90574299098135 -
3.09012201459043
H 0.80904087023170 -4.79047580535006 -
1.44667534022709
H 2.49926216562786 -4.94845964875411
0.37553766879703
H 4.28045405273471 -3.24620396450164
0.57753456452047
H 2.21369516273916 4.25742564168507
0.90608587502712
H 0.68781499137675 4.84268464680987
2.76055178675196

H -1.17253853158314 3.35599141976515
3.42677545707602
H -3.08325284062745 2.31470402999451
2.91669748939061
H -1.71368362977740 1.48973018398002
4.87017644392489
H -2.24308989096727 -0.18499817271068
4.51617635403040
H -3.43296137340524 1.01616034904469
5.05860642704754
H -3.41066509071069 1.12141049682027
0.56692646403927
H -5.50777310283222 -0.91984467940058
0.95922847748943
H -6.52496768710415 1.04942069035785 -
0.07028929922556
H -6.39023112623204 -0.20728871734534 -
1.33259864395509
H -5.23930016649224 1.16345994684250 -
1.29130834148131
H -4.06259719760708 -0.39038161039438 -
2.41218913618532
H -2.35172070818049 -1.86229750593997 -
3.42534394469358
H -1.24117753894174 -3.62342527518967 -
2.05140907377326
H -1.84448746424692 -3.87667628870626
0.35771273173594
H -3.55916280550379 -2.38893810609995
1.37108014103320
H 2.69808107094114 -2.09100235726689
3.55906884301523
H 1.84542506414849 -3.34072938125318
2.59420777765774
H 0.91672734651536 -2.35126063761856
3.74002970528459
H -1.16521743386268 2.99227963283675 -
3.58218687391706
H -2.44458616956702 1.81506974619256 -
3.93071570819789
H -2.85504711150012 3.30721155540567 -
3.02081108623999

91
INT-2
C 1.87594234936951 1.30497435205645 -
4.71220455481808
C 2.29401164376394 1.38919447718433 -
3.25188659721541
C 3.38347302675435 0.34626996959772 -
2.94938810759396
N 2.94356989101811 -0.81797892948155 -
2.42810625953228
C 3.82350474370579 -1.97335257642778 -
2.26159649604006
C 3.72360747298564 -2.53585193437603 -
0.84018724832019
C 3.54029338455728 -3.03002352894603 -
3.32077181408876
C 4.54291936985213 -3.40818308396990 -
4.22227200093020
C 4.30107466763755 -4.38467377697696 -
5.19364918016714
C 3.04635176144091 -4.99252389124004 -
5.27580207443520
C 2.03709552292104 -4.61683827129685 -
4.38272916945034
C 2.27967858364760 -3.64391276361927 -
3.41132229566798
O 4.54346537638336 0.60571225248016 -
3.23792778778521
O 1.10580127755745 1.20413989122897 -
2.47791982195613
C 1.08756267360763 1.49648613737830 -
1.17006525320371
C 2.18554093318130 1.96403613308181 -
0.42903322572867
C 2.01368526856943 2.26320474796871
0.92421115187720
C 0.79634912333012 2.08983273248620
1.58404846891016
C -0.29984992586812 1.59330163758201
0.86485226294077
O -1.50643178330997 1.31433464592140
1.36891453397325
C -1.95001414644807 1.73798213396800
2.66569365459825
C -1.55473005967398 0.75170890326523
3.74594495354578
C -3.48729979969768 1.83748281963598
2.51453310491694
N -3.87586658071262 2.55208097048418
1.43423371142257
C -5.24025984276983 2.50483766123318
0.91012471010028
C -5.74538505574146 3.91606709034298
0.61851111326580
C -5.25046749416641 1.55095508031089 -
0.28304273628225
C -5.11437091540617 0.17384881104380 -
0.03964537456529
C -4.99568577688826 -0.73337898117837 -
1.09296214978267
C -5.01407385850166 -0.27599702022890 -
2.41684988009410
C -5.15927950171734 1.09162273213033 -
2.67154724520588
C -5.27890882008549 1.99686152516496 -
1.61034115601402
O -4.24124215921039 1.29196420734590
3.30550510999440
C -0.13619684962978 1.32881919381073 -
0.50427432275010
I -1.82205155013061 0.72073384642969 -
1.58718054726345
O -1.76710817511059 2.58937003457762 -
2.64188969868277
C -2.11544833008549 3.66970162824465 -
1.97285421171256
C -2.12125981652363 4.90658847121918 -
2.84561376852348
O -2.41265908960608 3.69106343048520 -
0.78940793838403
H 1.11314709000911 2.06678142265651 -
4.93199791502354

H 2.75475265100728 1.46722087285330 -
5.35172273970009
H 1.45769916326520 0.31038809155938 -
4.93003610785976
H 2.72899099768200 2.37896736245462 -
3.03848332930250
H 1.95041393393027 -0.95075783957421 -
2.23581732882694
H 4.83913855376969 -1.58811069452202 -
2.42806231764724
H 3.99178810685319 -1.76561347274685 -
0.09982922497522
H 4.40821257253886 -3.38945415160160 -
0.71997494137363
H 2.70323516080934 -2.88305438313305 -
0.62304742390489
H 5.52393900635094 -2.92776519720133 -
4.16564894556375
H 5.09536752731958 -4.66801872575173 -
5.88964739717119
H 2.85360474354523 -5.75603110833929 -
6.03430494894681
H 1.05128795855821 -5.08623915342380 -
4.44056708970305
H 1.47848112962641 -3.35555647141851 -
2.72741495293415
H 3.16091512607602 2.08899760494641 -
0.89751757777910
H 2.87184255780379 2.63357257590362
1.49067342716747
H 0.70857840753610 2.30423407351011
2.64812370096293
H -1.54645435475502 2.74740594425719
2.86706824333086
H -1.92793180257063 -0.24971111310714
3.49225307855568
H -2.01169847568547 1.05310080454207
4.69875770606964
H -0.46376986545754 0.69974267215573
3.86309842710521
H -3.16876691239711 2.86827265782142
0.76913537691904
H -5.84827181659213 2.05814818372462
1.71052394604194
H -5.78768208765233 4.49497642981558
1.55312379944613
H -6.75361876146921 3.89335787461467
0.17658467363420
H -5.06955738213273 4.44539814812876 -
0.07091146245588
H -5.07531125937688 -0.18156080395552
0.99320910139611
H -4.86360703926819 -1.79683055205857 -
0.88293522479209
H -4.91972158909897 -0.98403868929525 -
3.24428323887830
H -5.17755574195168 1.45800676696332 -
3.70136132100212
H -5.38087851794294 3.06122954565468 -
1.82735108607884
H -1.17347613268699 4.98571399839558 -
3.39813778462192
H -2.92673841585404 4.81613852001449 -
3.59182030407419
H -2.28448162622744 5.79911768799762 -
2.22884490442633
O 0.41152251338032 -1.68309452589924 -
1.43771983528306
S -0.62961217558749 -2.19343035518863 -
0.51934633920865
C 0.00744524771885 -2.09537505289472
1.14253502589110
O -1.82521233551900 -1.16857971578743 -
0.48857172729496
O -1.15154067169710 -3.53884086647115 -
0.73642446742438
C 1.12026847757404 -1.30630033828466
1.42141266941377
C -0.64378458958693 -2.81194663197486
2.15159117202604
C 1.58159503035444 -1.22804259725785
2.73708841621585
H 1.62664095310974 -0.76885164439141
0.62096574188191
C 0.94406396160315 -1.92272588968200
3.77335357249768
H 2.45605606770402 -0.61075010266309
2.95822027260397
C 1.42786855606172 -1.82288992718264
5.19663949435585
C -0.17313095581432 -2.71762907670020
3.45765333503031
H 2.34127688223894 -1.21504324235044
5.27373511656123
H 0.65973189623792 -1.36487452172658
5.84291262309832
H 1.64561935444503 -2.81934824832670
5.61503465904589
H -0.67925010010599 -3.27395648088093
4.25159412328585
H -1.50048062467842 -3.44096270409530
1.90357749536462

112
INT-3
C 3.06985016361934 0.37774457323222
3.14204570650790
C 1.64501764978398 0.46062206941195
2.63750758536498
C 0.85136773141271 -0.83448573501037
2.93309788639027
N -0.48662378554815 -0.68085021502527
2.87811098595066
C -1.41024571610637 -1.80414294028019
3.03966268435286
C -2.60711092454483 -1.35217843117307
3.87563688473943
C -1.77149932799464 -2.38591037479357
1.67597036101428
C -0.87840907845611 -3.27880861272601
1.06150392255813
C -1.12695927783178 -3.76490375801231 -
0.22427177740332
C -2.27328121967684 -3.36239083607782 -
0.92088218193428

C -3.17477823585396 -2.48496156796323 -
0.31157720768976
C -2.92477682948281 -2.00518541086420
0.97772964929093
O 1.42932824882182 -1.89026381240642
3.16494605376279
O 1.61658772973349 0.57726810749912
1.19905597768104
C 1.27989836059905 1.73887103916568
0.60184231921084
C 1.78219670690660 2.99386867982308
0.97461972059027
C 1.42175400544813 4.11835547853944
0.23033597538095
C 0.56695086002128 4.03550078970617 -
0.87072768027033
C 0.02823439991141 2.79206569224336 -
1.23084400425386
O -0.80679086556231 2.56492818944270 -
2.25139504103963
C -1.62172668118207 3.54854818641882 -
2.89105466998455
C -2.48739449341486 4.35659441093703 -
1.92663541731424
C -2.51481444615467 2.73215353857604 -
3.84307403909663
N -3.08031758516716 1.65556916034244 -
3.26215768381239
C -3.90818619438927 0.71189770080696 -
4.00195163429342
C -3.18874267847709 -0.63920887613554 -
4.13655355768248
C -5.28288221399081 0.54916985084923 -
3.37394413197350
C -5.42044096381229 0.26444529879437 -
2.00596064383530
C -6.68578518319238 0.07852923028730 -
1.44512812235455
C -7.83194399033786 0.16863291047998 -
2.24178487072806
C -7.70314794324923 0.45111632358550 -
3.60363226213767
C -6.43568061164946 0.64141750650341 -
4.16303305458781
O -2.69114142078609 3.08244050023297 -
5.00580499217143
C 0.41170152682905 1.66660350662647 -
0.49034484996725
I -0.37335031686459 -0.22674075085209 -
0.97785580366037
O -1.98863671500635 1.30513479349430
1.65971375579296
C -2.75927775734696 1.40924362855912
0.69483166934566
C -4.12094396746551 2.06172562293964
0.91348542188276
O -2.52490845808608 0.97246237459511 -
0.48979943306082
H 3.56385205022590 -0.50823329101053
2.72013921824627
H 3.63917147779733 1.27854194150032
2.86874352641211
H 3.07559505701586 0.27424549258207
4.23773959324371
H 1.10903175867291 1.32418092314002
3.06449517687839
H -0.89900444434447 0.17565155943396
2.47976030621664
H -0.84933081591040 -2.57535211121550
3.58719877058024
H -2.27070121732703 -1.06900566554959
4.88440180121350
H -3.35253108852280 -2.15699338098911
3.96785534413366
H -3.08914619864954 -0.46960164162651
3.42800377747048
H 0.02725355802160 -3.57148624845022
1.59819832461074
H -0.42841163700279 -4.46864220091568 -
0.68480644972280
H -2.46973049981350 -3.74320976752713 -
1.92655203266472
H -4.07585074128525 -2.16870543688492 -
0.84213179051141
H -3.63556254479128 -1.31605938798253
1.43490591385858
H 2.46334100001092 3.07806102648102
1.82171396668654
H 1.83272956943457 5.09213165494138
0.50815839767908
H 0.33020213616663 4.92357955518807 -
1.45580211310627
H -0.99598857913966 4.20411955577271 -
3.51796821594496
H -1.89405650804194 5.00857786587469 -
1.27195528140625
H -3.08666866496259 3.68406864753085 -
1.29846816782878
H -3.17060148820381 4.98855529652603 -
2.51442211928168
H -2.86423952467230 1.45123520863628 -
2.27813744357569
H -4.03193235129175 1.14988736118593 -
5.00238130668928
H -2.99919374733021 -1.08188379250258 -
3.14582215358396
H -2.22232101627310 -0.51089664048526 -
4.64874544152243
H -3.80127239886325 -1.34932480053969 -
4.71338192188479
H -4.53181841399332 0.20519153257933 -
1.37373184534538
H -6.77826297495827 -0.13565501661762 -
0.37669422092446
H -8.82167024855456 0.02268840601713 -
1.80123606920124
H -8.59286204902625 0.52943286812176 -
4.23414975063936
H -6.34086107766558 0.86910613829651 -
5.22881770490032
H -4.56498885817958 2.43279438227990 -
0.02001856542400
H -4.03718972936829 2.86812184695294
1.65561547719253

H -4.80471935610887 1.30083232656240
1.32754185365359
C -0.16037302443543 3.75909051923632 -
6.91926480165340
O 0.84206645192629 3.89693969460501 -
5.91996605619128
H -1.15082278316534 3.58994602634300 -
6.46851343070733
H 0.08434075114907 2.93308221779560 -
7.61177857551738
H -0.16215008020041 4.70361480505544 -
7.47971220888075
C 1.08739957461597 2.86148171821478 -
5.08590559689280
C 0.39772603580125 1.64052715578757 -
5.10478637714683
C 2.12959676866016 3.05493964042974 -
4.16121125550862
C 0.76869108568983 0.62829701220327 -
4.21873777092908
H -0.42673136550133 1.47638816869075 -
5.79672074813049
C 1.81458848949751 0.80566830734018 -
3.30734690307525
H 0.25072720538167 -0.33190273453651 -
4.25651089730418
C 2.28517463301365 -0.28784006383381 -
2.43132860191626
C 2.49180908091563 2.03822449070991 -
3.29461380593297
C 1.60360231134076 -0.96503772444648 -
1.49118845842123
C 2.12296925764186 -2.10612806039286 -
0.68044907709653
H 2.06954306589611 -1.89202339826160
0.39760182032002
H 1.53536137164761 -3.01906811171689 -
0.86523437474387
H 3.16787726967678 -2.30806633513347 -
0.95407193820924
H 3.31451719218883 2.18934070583245 -
2.59345654035334
H 2.65095261170954 4.01371940647195 -
4.16020131933569
O 3.12284102650975 -2.17579825224132 -
4.44729317038051
S 4.19648865174635 -1.31643471271994 -
3.96038445910548
C 4.39055645615663 0.06834747802090 -
5.05150018239788
C 3.46897438476018 0.25055869779178 -
6.08268648579844
C 3.54343139033204 1.40941279628231 -
6.84799049615762
C 4.51606110589637 2.38911084098600 -
6.59185304404323
C 4.50430838184438 3.67842465506434 -
7.36886042224178
C 5.45639405380174 2.15187271770094 -
5.57660252079719
C 5.40371934448566 0.99453351950008 -
4.80115871120054
O 3.64423377816451 -0.57647584205523 -
2.55120457140279
O 5.49741873609329 -1.82571655783836 -
3.56435392473299
H 2.69504003191215 -0.49675360921425 -
6.25495434813302
H 2.81205242554802 1.57334332842199 -
7.64359483368934
H 4.53055403325510 3.49331561470050 -
8.45528580401610
H 3.57593055831081 4.23600732481929 -
7.15673568448458
H 5.35816708613527 4.32123812419797 -
7.10997294597520
H 6.23345422657626 2.89500599880080 -
5.37891945371277
H 6.12379715249992 0.81675094530850 -
4.00034568758624

113
INT-4A
C 2.37562877783718 0.61027774735190 -
2.57849307643708
C 0.95873672611261 0.60593502232307 -
2.02889475174941
C 0.62339160801334 1.94571632246010 -
1.34034620686949
N 1.00518145450668 2.05937779203427 -
0.05593574486287
C 0.61777469333991 3.21889260060345
0.74994567580676
C -0.80091281883489 3.03819899090771
1.29605163940034
C 1.68587938045570 3.48387768039928
1.80345255824286
C 1.39179687180173 3.58894032806127
3.16733907963199
C 2.39890456204312 3.86755525204906
4.09704665220823
C 3.71838281175016 4.03177039027676
3.67916714173530
C 4.02704433299256 3.91455159565265
2.31936056016196
C 3.01934029471654 3.64574134757548
1.39472683376115
O 0.06203992755789 2.81834063437250 -
1.99291867414835
O 0.85820599196147 -0.52330674014421 -
1.15140186893837
C -0.28311820565733 -0.73225167673687 -
0.46565951171271
C -1.49530698615891 -0.06669411487168 -
0.70250451988689
C -2.59541587599282 -0.34279193769528
0.11112964962341
C -2.53267060735031 -1.24733671199348
1.16837572983904
C -1.32878891968186 -1.91749726438914
1.42410033092043
O -1.12514030240016 -2.81001096328828
2.39671411629101
C -1.93958740805326 -2.95053174743723
3.56912841767298

C -2.14564716136012 -1.65199559217533 4.34557624160231
C -1.17841393370422 -3.97381128720291 4.42797448442381
N 0.13316068975234 -3.70377419381368 4.56953397904587
C 1.03431294100885 -4.59686416723955 5.28968797650197
C 1.88587443865910 -5.40769795341666 4.30118559949756
C 1.88542287024217 -3.83775021021344 6.29465781145121
C 2.80060802099991 -2.85932291013420 5.87543554043164
C 3.59196089545723 -2.18329289967504 6.80486619607797
C 3.48166377639149 -2.47370936752636 8.16807206277937
C 2.57164605453480 -3.44344643124473 8.59617720876254
C 1.77992253297124 -4.11958733513191 7.66281253327894
O -1.75951615985420 -4.92310387491267 4.94800316163439
C -0.24577732502511 -1.67903535303175 0.56595173123214
I 1.49643393444155 -2.84040864115739 0.82918470685797
H 2.50978977067166 1.48649930621117 -3.22934925194482
H 3.10211274339962 0.66319065052501 -1.75359202479903
H 2.57046942047642 -0.29828553405477 -3.16864738495891
H 0.23079958698855 0.50287197946509 -2.85021555679005
H 1.42172022515506 1.27188172229599 0.45752731241549
H 0.60787166543236 4.07442695778711 0.05408010490353
H -1.49319525052820 2.89434520578068 0.45401914959677
H -1.12779039246194 3.93159667944014 1.85093389010984
H -0.85392359304918 2.16125105640452 1.95761502763248
H 0.37214724759346 3.43639497344956 3.51963668805892
H 2.14854566455093 3.93975486559804 5.15771935015283
H 4.50527921247675 4.24174038471818 4.40763143489845
H 5.05914922367271 4.03660056032690 1.97886041164826
H 3.26495290144429 3.55162362012659 0.33354226166546
H -1.57306709208365 0.68304587382190 -1.48839468650747
H -3.53436241773650 0.18155948189871 -0.08214980143887
H -3.40799487954538 -1.44300165128111 1.78547279231804
H -2.90012098636817 -3.41385481512610 3.29492437386690
H -1.19463144762198 -1.11947892541190 4.47673359053069
H -2.57111459060357 -1.90278135430088 5.32954094208412
H -2.83816558313750 -0.96721802170948 3.83902031525970
H 0.53875980480432 -2.90256245291915 4.07044135080616
H 0.38187359373798 -5.29095416395019 5.83751062876097
H 2.54684081652609 -6.10655576840163 4.83747709953872
H 2.51824910395304 -4.74332238725770 3.69083097996395
H 1.23628970298280 -5.98547284821079 3.62440891169244
H 2.87689186439048 -2.59946066698565 4.81825011395897
H 4.29106267374970 -1.41988849873068 6.45885874145762
H 4.10239873593625 -1.94296294108852 8.89525100499995
H 2.47579231681906 -3.67588808418846 9.66030360818171
H 1.06700141115234 -4.87699449968129 8.00146466648965
C -4.03012969424284 -6.46758527556607 3.25876155133790
O -4.10167580416664 -5.57616151117427 2.15086685542231
H -3.32052704393490 -6.10201307712445 4.01752148021879
H -3.74528977460141 -7.48201148644256 2.92649011938093
H -5.04265730226977 -6.50306556615510 3.68263201117431
C -3.01624860048444 -5.39972706538863 1.37134748853390
C -1.74397806917648 -5.92832696595740 1.64206612871421
C -3.20664803866776 -4.60945239998493 0.22202404274363
C -0.68562941532879 -5.65352761706718 0.77471210478849
H -1.56738389956514 -6.51820955644866 2.54037839702325
C -0.86516526377393 -4.86512444227591 -0.36789588828098
H 0.30366875386720 -6.06131994883518 0.99598185832054
C 0.23789718054079 -4.57417765615954 -1.31666116012758
C -2.14818802603051 -4.35280685287026 -0.63509326073651
C 1.34872659362049 -3.84765219711846 -1.05118748539811
C 2.44239583465771 -3.53613395651954 -2.02530631121043
H 2.32326890008182 -4.16043478211385 -2.92185271861005

H 2.40729589238383 -2.47680117593769 -
2.32703934493955
H 3.44242777515447 -3.72329754741776 -
1.59916897436970
H -2.30375122935210 -3.72521615725346 -
1.51593200936634
H -4.20129872513335 -4.20118116703486
0.03444504811010
O 0.15779661683886 0.41833322301732
3.27073078238891
S 1.50970851369619 -0.12391289012282
3.02500158888891
C 2.57123690858228 0.47284096673194
4.33050457821178
O 1.54578675636475 -1.64598852334073
3.15391866753112
O 2.15394058878298 0.26434719392333
1.73986024161175
C 2.02001796858295 0.79342551284926
5.57128108869607
C 3.93783784474569 0.60570837834138
4.09614073488653
C 2.85455811527199 1.25060229226661
6.58781213722659
H 0.94399672744996 0.70059389425283
5.72432348362461
C 4.23686101284806 1.39236234167850
6.38377706416618
H 2.42543754649906 1.50403191455600
7.56129172612947
C 5.12929094476605 1.86687972489987
7.50170831253341
C 4.76156854674299 1.06377493392132
5.12513060136608
H 6.12537620388877 2.15372634643750
7.13291805583446
H 4.69382216673927 2.73394175924545
8.02385960122654
H 5.27014884940744 1.07412609281358
8.25753548075663
H 5.83278343772350 1.18434524808550
4.94139108633594
H 4.33822206410645 0.38336313418961
3.10616055633592
O 0.07738543049456 -4.98886244802258 -
2.60368292695719
C -0.54706762749694 -6.25490607207261 -
2.85344620976525
H -1.58881198097202 -6.24148823547238 -
2.49377419653884
H -0.01290792804572 -7.04234133238642 -
2.29175791232484
C -0.48767448415134 -6.50610639132191 -
4.34552791043348
H 0.55437858648775 -6.54020511954151 -
4.69961096514311
H -1.01146150045816 -5.70724859516487 -
4.89333718867995
H -0.96930994572625 -7.46645102210415 -
4.58748449345958

112
INT-4A-CF3

C 2.41544359569829 0.69109729166019 -
2.56667213522707
C 0.99247086378870 0.69955140090235 -
2.03310231098049
C 0.66304938546714 2.04061164859225 -
1.34420817064790
N 1.04179390364918 2.15058849396307 -
0.05899452936899
C 0.66893293810156 3.31738852039755
0.74405317000971
C -0.76115114900533 3.16902851470764
1.26953320933400
C 1.72850724432263 3.55458319064561
1.81260373850159
C 1.42001262121920 3.65266815455413
3.17388205888141
C 2.42088388609248 3.90409746824065
4.11796669807133
C 3.74809579241988 4.04784214070828
3.71723321807887
C 4.07091407373727 3.93733726431165
2.36019180398473
C 3.06955715959368 3.69561317269120
1.42122725954724
O 0.10907640361591 2.91709703986881 -
1.99795399267146
O 0.87134117764412 -0.43283102249561 -
1.16073773115401
C -0.27996150683944 -0.63862019685670 -
0.49232433541086
C -1.48181115466919 0.04545190046303 -
0.73078219469000
C -2.59653562572345 -0.23500059944198
0.06148615102976
C -2.56019675259776 -1.16414234329141
1.09919534751226
C -1.36634833663721 -1.85130789027162
1.35864051413344
O -1.18870614196222 -2.76775540650562
2.31325951629433
C -1.98561478086853 -2.86728696535732
3.50836604641020
C -2.11188708134625 -1.55864424440678
4.28048867103417
C -1.25228718529822 -3.93845979547804
4.33281197400559
N 0.05529355198809 -3.66473134777727
4.53877095135750
C 0.93356141129249 -4.58873360599337
5.24658041932201
C 1.74860056696064 -5.43417552162800
4.25530327665652
C 1.82281848753272 -3.85687813892008
6.23807213866344
C 2.73682863662923 -2.88204888640019
5.80747771790072
C 3.56560280416524 -2.23194303113258
6.72282491450115
C 3.49554713475299 -2.54623592306997
8.08338825692850
C 2.58807395039478 -3.51332594210816
8.52280447550072

C 1.75820721946841 -4.16265700491928 7.60362253861448
O -1.83868922347256 -4.92761364930009 4.75367510841096
C -0.26835786550156 -1.60540474970524 0.52186055117287
I 1.46536980967371 -2.76990058119746 0.82343346594439
H 2.56561308758638 1.56777891469350 -3.21340185543197
H 3.13315000926577 0.73453714261820 -1.73360477941438
H 2.60865266857419 -0.21743987915395 -3.15751233832362
H 0.27195255998459 0.60574622021440 -2.86199131556251
H 1.45591874133242 1.36031084834245 0.45245819776377
H 0.68829041491715 4.17469198968774 0.05040159534195
H -1.44461187864445 3.04472800067702 0.41722613869185
H -1.07414759051734 4.06841244935754 1.82268794433237
H -0.84326327235069 2.29161570527781 1.92717781945815
H 0.39359953327585 3.51606248358882 3.51263356555018
H 2.15903947600065 3.97135780223743 5.17623319779164
H 4.52991666007971 4.23662532913529 4.45685236250305
H 5.10902317839423 4.04381961969136 2.03305488368301
H 3.32630666590942 3.60733578891973 0.36214332898138
H -1.54071455406032 0.81153789519258 -1.50247547985886
H -3.52600825917878 0.30546045400687 -0.13328328999111
H -3.44441851151798 -1.36041088805166 1.70416856944101
H -2.97073965224945 -3.28218673937652 3.25519149881594
H -1.13517459498408 -1.07497108113324 4.40551177804735
H -2.54406689497625 -1.78199012526512 5.26818236590836
H -2.76992739607054 -0.83889513911036 3.77588177393596
H 0.47356617242719 -2.84665610779198 4.08324956100366
H 0.26531762326968 -5.26214577236237 5.80173631801266
H 2.39554226519757 -6.14756741835276 4.78955942662960
H 2.39206170386514 -4.79476243809204 3.62961690431394
H 1.07235599628911 -6.00006415902032 3.59562276929226
H 2.78471322378467 -2.60417679200207 4.75305021270049
H 4.26269470459264 -1.47053091273440 6.36841601435511
H 4.14583193911029 -2.03643276707227 8.79962921675805
H 2.52346535780939 -3.76429930980435 9.58505851858353
H 1.04715097232286 -4.91751902019629 7.95163541067587
C -2.91737355541016 -5.60688075639689 1.48284048655520
C -1.61797804561794 -6.04423360061497 1.74272620848405
C -3.18085884210713 -4.80552015593358 0.36484338842570
C -0.58565119143887 -5.69856282989078 0.86935676697241
H -1.42037533031223 -6.64214115443048 2.63215584419100
C -0.84171129760726 -4.91469325917993 -0.26251999790009
H 0.43008197117877 -6.04932011563012 1.06316408873956
C 0.22014200143754 -4.59106842207240 -1.25544745035406
C -2.15003467866956 -4.46521141509636 -0.50437530186090
C 1.30140179575915 -3.80826583051483 -1.04166337197699
C 2.34122237973636 -3.46747404718545 -2.06339005458285
H 2.21991357192373 -4.11280851028340 -2.94454463584406
H 2.24618419441931 -2.41622552059252 -2.37914005368440
H 3.36240653115701 -3.59896253127470 -1.66832497773265
H -2.35179513638328 -3.84006310603154 -1.37673814981800
H -4.19679132238495 -4.45499710763953 0.17114138333077
O 0.16002075513687 0.53410243497873 3.20710836309397
S 1.49726202101358 -0.05010512186165 2.98487075682747
C 2.55853495834854 0.50754048040933 4.30645140067105
O 1.47899408540500 -1.57449388436539 3.11516577350645
O 2.17325330931616 0.31175003854280 1.70779765605970
C 2.00351373882205 0.80811074437998 5.55109767936238
C 3.92790479809084 0.62523933280201 4.08294517698545
C 2.83825613433654 1.22811946422382 6.58266395268745
H 0.92540897897514 0.72683593060130 5.69643375228490
C 4.22424238394587 1.35407496815864 6.38993304062915
H 2.40731760473981 1.46288854788378 7.56000215251452

C 5.11285929373187 1.79695800221945
7.52375569641199
C 4.75192544224522 1.04744996268132
5.12750066911907
H 6.15219381376547 1.94167176228108
7.19373099976374
H 4.75939255780590 2.74427163780437
7.96327234598743
H 5.11835390662671 1.04953473748692
8.33581838222632
H 5.82620423931149 1.15426216311482
4.95377663785619
H 4.33123341868028 0.41739425818760
3.09109668682478
O 0.02779170832900 -5.04810239959459 -
2.52239259461278
C -0.51316388384291 -6.36312667973338 -
2.71496809205794
H -1.53453629293745 -6.41599131431185 -
2.30339894752108
H 0.10598689909562 -7.09520427078962 -
2.16579188294605
C -0.51041484443806 -6.64522615124136 -
4.20229919190726
H 0.51235020681169 -6.61364982220737 -
4.60867012254682
H -1.11792192282152 -5.89970315614722 -
4.73856996012142
H -0.93248609732383 -7.64320051932114 -
4.39846664171308
C -4.06887098868212 -5.99738160407175
2.37688664228192
F -4.62804295352171 -4.91633074502304
2.96223041413842
F -5.05001305523445 -6.57996523204990
1.65314348122036
F -3.71579486913796 -6.85206923784049
3.33573903938847

113
INT-4B
C 2.29014282798640 0.73023146176090 -
2.61748512401687
C 0.88146275084736 0.72565519845259 -
2.04710578272296
C 0.56745593539611 2.05340532784251 -
1.32620296993107
N 0.97024672391883 2.13825071371174 -
0.04587747340559
C 0.60685279151640 3.28605936189973
0.78747658572587
C -0.80535458354886 3.11016075046364
1.35151983978127
C 1.69313392823648 3.52004699498478
1.82955746532530
C 3.02098096543235 3.68303808133241
1.40331832507838
C 4.04495615291140 3.92402516387001
2.31773536989987
C 3.75846811151213 4.01196605732547
3.68459142329060
C 2.44471195484244 3.84644011406806
4.11966321750625
C 1.42132368789005 3.59529619359300
3.20002419719103
O 0.00308522955372 2.94357906051226 -
1.95192915438275
O 0.78467228711992 -0.42059221568928 -
1.19164339071073
C -0.34795726082280 -0.63493970948826 -
0.49345079556305
C -1.55807380989540 0.04495722968542 -
0.69783488651258
C -2.64813058430251 -0.23993062310388
0.12623577791984
C -2.57678173122090 -1.16708943729371
1.16321764094956
C -1.37467923652530 -1.85190884777469
1.38628855967210
O -1.16333720771363 -2.76617368402207
2.33702315041543
C -1.95999264659991 -2.92306818396820
3.51942832288983
C -2.14045007608183 -1.63878817126085
4.32522768681609
C -1.19488183922201 -3.97123958017708
4.34410503682456
N 0.12154791481296 -3.71701886210453
4.46941666859591
C 1.02501062774520 -4.63381715402353
5.15619512428843
C 1.85010771633940 -5.43425099845404
4.13719068646422
C 1.90165045998399 -3.90387816707377
6.16079585780673
C 1.81847037361974 -4.21325517581966
7.52452026867998
C 2.63335043120495 -3.56378629765646
8.45690699735677
C 3.54431833818363 -2.59342323876287
8.03226807281162
C 3.63253620923542 -2.27566887956723
6.67354274893605
C 2.81820115494295 -2.92520012024383
5.74508412068911
O -1.77653448081692 -4.92589612101854
4.85366833719926
C -0.30299142193264 -1.60393319987464
0.51698622557227
I 1.43204161588405 -2.78701533418387
0.72511329486452
H 2.46883610240229 -0.16758169121209 -
3.22882782118235
H 2.42172252718567 1.61848860757913 -
3.25236240629352
H 3.02906814834981 0.76061900969009 -
1.80256241140723
H 0.14081755362497 0.64504569478485 -
2.85954207454078
H 1.38950781903485 1.33797570334751
0.44534229524461
H 0.59546917876663 4.15471637442556
0.10807692303622
H -1.51162931735704 2.98861818411545
0.51760295093566

H -1.11446919721777 3.99695250182533
1.92689911507656
H -0.85835891963424 2.22223750864025
1.99827705249080
H 3.24887176986706 3.61220313181445
0.33638293241956
H 5.07235993299676 4.04749115910661
1.96381037864133
H 4.55816934320740 4.20025505492429
4.40501866114284
H 2.21161897167515 3.89581394336761
5.18557574681231
H 0.40636746190277 3.44084671715639
3.56481870519779
H -1.64133245074757 0.81191615467418 -
1.46636427561851
H -3.58567524879584 0.29550055586357 -
0.04185738615191
H -3.44440109720420 -1.36916895847583
1.78909863401430
H -2.92955641856453 -3.37123231146748
3.25161746363729
H -2.55509614269880 -1.90485477303477
5.30979894971799
H -2.83172570995905 -0.93603291410400
3.84221697704604
H -1.18161252625500 -1.11987488677715
4.45346115108769
H 0.52716249058732 -2.90925420949821
3.98096804166191
H 0.37442356535403 -5.33153881872891
5.70158016850787
H 2.47974646923842 -4.76511925517350
3.52921961036284
H 1.18191623773369 -5.99072860068978
3.46065823083064
H 2.51166534796056 -6.15145165203298
4.64788460037637
H 1.10478906474793 -4.97114119578317
7.86051424586093
H 2.55476148954314 -3.81748106313402
9.51759269816366
H 4.18311118553721 -2.08350587812994
8.75869927311383
H 4.33222509153178 -1.51133669526388
6.33073687551247
H 2.87776334569671 -2.64415849856062
4.69225800519850
C -4.08070374825296 -6.42413308889734
3.17101328050971
O -4.16772291816616 -5.51099122877301
2.08202444628933
H -3.35835604394399 -6.07483326374758
3.92537364540145
H -3.80343439993030 -7.43239821391508
2.81447829917483
H -5.08650841181953 -6.46598529327273
3.61000873007836
C -3.09481713240813 -5.32181522815950
1.28864003997953
C -1.81980468115512 -5.85915449676176
1.52782064058764
C -3.30133987037766 -4.50837748866910
0.15846707315863
C -0.77562028064021 -5.57098485436667
0.64792462813676
H -1.62995346957793 -6.46668826027701
2.41151070125811
C -0.97119156058758 -4.76061464891011 -
0.47686068778730
H 0.21625029772821 -5.98398124314949
0.84723093444975
C 0.11733782046056 -4.45640966597211 -
1.43858043059610
C -2.25671511302115 -4.23881641541018 -
0.71163243424572
C 1.24131910379747 -3.75030219508753 -
1.17431723745061
C 2.32233130827959 -3.42791762961888 -
2.15891034859482
H 2.30204771420472 -2.35914681539259 -
2.42657622372609
H 3.32636231458483 -3.64732017836548 -
1.75803934896044
H 2.17434221515685 -4.02101353521694 -
3.07210569724341
H -2.42512244950865 -3.59511392736447 -
1.57821930660217
H -4.29752385854116 -4.09327502890914 -
0.00435053478087
O 0.16069877225276 0.43028226712740
3.25212887521998
S 1.50453735193538 -0.11698457996612
2.97551708374939
C 2.58976937626871 0.44555111639195
4.27682791904403
O 1.53067834767709 -1.64156067532028
3.07365090318617
O 2.13248752720971 0.29220035225297
1.68880284931167
C 2.05979514122277 0.73937235851628
5.53374428926359
C 3.95302267887380 0.57640701820636
4.02416495593661
C 2.91255582052383 1.16748916530745
6.54732690724149
H 0.98593112648093 0.64739791594228
5.70183664837081
C 4.29248579628839 1.30729244992820
6.32461206955238
H 2.50054694227018 1.39832033198160
7.53377708304129
C 5.20154677211101 1.75994806163051
7.43828957287534
C 4.79557400598446 1.00544162938533
5.05088635656044
H 4.83951916108287 2.69538715211706
7.89579198394436
H 5.24648839365342 1.00606605067140
8.24331724817171
H 6.22808395889657 1.93052771790225
7.08156360705112
H 5.86467937141367 1.12307864740453
4.85371414976750

H 4.33687110009281 0.37412614075380
3.02340656043870
O -0.07758282146253 -4.82609174925545 -
2.73620723913128
C -0.69688217191880 -6.08842274590978 -
3.01624555683338
H -0.90071345308490 -6.05682012562473 -
4.09674177780017
H -1.66269745984621 -6.16167819650642 -
2.49068951387342
C 0.20405203549153 -7.25933238496565 -
2.66344772353006
H 0.39204654604575 -7.30295259186035 -
1.57928264942631
H 1.17299766114330 -7.18164485766913 -
3.18152091797299
H -0.27306745583200 -8.20686341539411 -
2.96126535939813

113
INT-4C
C -1.06024360122082 1.64816970778858 -
2.70443605216574
C -0.44792193102440 1.56298519892650 -
1.32133809331112
C 1.04211000406484 1.98025444542380 -
1.32278264531225
N 1.56162290028327 2.15821095531752 -
0.09121024333295
C 2.98264984579348 2.43342785116230
0.10682319758806
C 3.16333290787431 3.36132690422732
1.31017611184675
C 3.77926897424068 1.13798951261470
0.21758862979758
C 4.52967481169849 0.67729630475966 -
0.87214141028115
C 5.24081218220294 -0.52475078185387 -
0.79375507410194
C 5.20787487042402 -1.28597556878521
0.37843123179603
C 4.45421162263671 -0.83803765810507
1.46925032327186
C 3.74345619502080 0.36163216178282
1.38480671740453
O 1.66675752710121 2.10329178780462 -
2.36878919697717
O -0.42747039674502 0.20758709325802 -
0.83893739471962
C -1.36339177288729 -0.26205504880901
0.00791232168719
C -2.70449754549178 0.14193493278736
0.03924424000069
C -3.58122395553506 -0.49412049368066
0.92160261962257
C -3.16815691714635 -1.50430567800275
1.78903110927708
C -1.81874820932532 -1.88102280892364
1.80854407318955
O -1.25579377267360 -2.78912192831638
2.60675795320690
C -1.84120594555052 -3.41627853407796
3.75407894821680
C -2.41592389833018 -2.45817636657527
4.79648993047345
C -0.67612999991790 -4.19831695376676
4.38262942457674
N 0.48673221516341 -3.51908713054127
4.40117763581995
C 1.68437728121806 -4.02321399334972
5.05684323418763
C 2.90044478456711 -3.79677680962776
4.14974149770439
C 1.87747073768258 -3.38698314843899
6.42638608192890
C 1.83308205005868 -1.99226136547097
6.56995108790121
C 2.02304021721835 -1.39970290650631
7.81903153854219
C 2.26420708555525 -2.19619776900681
8.94365386822082
C 2.31123056692185 -3.58621222839264
8.80969882071618
C 2.11630313066956 -4.17660813505016
7.55684101649964
O -0.84365262740352 -5.31823376881880
4.85649374488678
C -0.95524782267541 -1.26831412555329
0.88947229304457
I 1.07900298184709 -1.81212427442187
0.85187499833230
H -2.09874262357289 1.28627532046532 -
2.70184073755974
H -1.04812210218023 2.69113262478872 -
3.05453773486119
H -0.46951869820406 1.05270279878323 -
3.41399615396690
H -0.99762133217924 2.18209273069699 -
0.59203671184490
H 1.02640348878936 1.89534297282425
0.74382550108421
H 3.31951951684667 2.94636563368341 -
0.80530213051984
H 2.72571843038266 2.92384156354951
2.21983138386411
H 2.65613445742014 4.32085226012174
1.12502508123159
H 4.23148575740624 3.55697057005089
1.49041694813005
H 4.53955601725126 1.26166532195404 -
1.79555998061819
H 5.82303997548922 -0.86785171597869 -
1.65326565348437
H 5.76854408273060 -2.22232253830260
0.44331822451747
H 4.41446925801260 -1.42670518039504
2.38983845420033
H 3.11521962300222 0.67504434504865
2.21746906120578
H -3.06093300900084 0.92354086080484 -
0.63102591116372
H -4.63159576879036 -0.19246689546210
0.92814038459167
H -3.88047616533507 -1.98571947789608
2.45588790347795

H -2.59435570399530 -4.14870081511842
3.42621312308016
H -3.37184853527609 -2.01798783689394
4.48458318764833
H -1.71890580410365 -1.63400106670349
4.99395580639459
H -2.59007121975739 -3.03122705627868
5.72009715230478
H 0.52274741737551 -2.57294247600998
4.00771241904049
H 1.52327126618506 -5.10148330318189
5.19974791680119
H 3.03578284125340 -2.72106430967566
3.95445098728294
H 2.76276329070890 -4.31006317134929
3.18421821385374
H 3.81678795961935 -4.17781110654950
4.62567224733938
H 1.64248682390030 -1.36629195256497
5.69785085622947
H 1.97787513045936 -0.31237774426081
7.90857476799509
H 2.41039368936290 -1.73418804937701
9.92380915245372
H 2.49236119878946 -4.21597367242664
9.68505480394045
H 2.14075943361634 -5.26583821530114
7.45765266386948
C -2.72914374579159 -7.27107716516824
3.06522200557003
O -3.19538183475651 -6.33086202501743
2.10308696686097
H -2.09207979480283 -6.78577769683198
3.82212971786336
H -2.17782100920385 -8.09305007267364
2.57438572837456
H -3.62765275155355 -7.68197882053874
3.54480090248729
C -2.30932586670157 -5.72155550113895
1.28852190321480
C -0.91497126530221 -5.84320712769905
1.39428759709898
C -2.85822336295801 -4.89811972134935
0.28768228684191
C -0.09448729821465 -5.13950765962079
0.51127713413029
H -0.46736920988970 -6.45277649464402
2.17795080780429
C -0.63072078715026 -4.31720450688308 -
0.48656559727753
H 0.99031509462795 -5.23032732115893
0.60641733368200
C 0.22318515169547 -3.57542333310118 -
1.44808219627218
C -2.03020153453609 -4.21372348132869 -
0.58878564980560
C 1.08287617016000 -2.57601724979135 -
1.14657479853642
C 1.93854467558424 -1.81696923368453 -
2.10963949986495
H 2.97808021948887 -1.73337257774927 -
1.75598799371776
H 1.93468202067878 -2.33621558244089 -
3.07833708141429
H 1.56691420165160 -0.79118215114494 -
2.26211066414521
H -2.46592373468557 -3.57038178041308 -
1.35683738368785
H -3.94462659707569 -4.81071512088015
0.22843916983393
O -1.12082410069297 0.29092874161977
3.86878282966818
S 0.30717373430311 0.47267844406151
3.55144190034326
C 1.06834855112974 1.22313935353478
4.99400538422147
O 1.05273118310222 -0.84491475568902
3.36882143022019
O 0.58968836387170 1.38594164734652
2.40976545266535
C 0.24977418402347 1.70536650869319
6.01296234271431
C 2.45913719379982 1.33696322894001
5.08626714420324
C 0.83029388729168 2.31036618774511
7.13130119484062
H -0.83202594408212 1.59824253495469
5.92077057956005
C 2.22085172424867 2.43786824123826
7.24824623943011
H 0.18649219004060 2.68782409223589
7.93065160933908
C 2.85206563939109 3.06565933451278
8.46437357330918
C 3.02400640748756 1.94068659283726
6.20606423785246
H 3.58826883910257 3.83576847858127
8.18171922526834
H 2.09974297729201 3.53479758203897
9.11539998150312
H 3.39051568172835 2.31103947554556
9.06364288774316
H 4.11215331059730 2.02419562485484
6.27835755446731
H 3.09899650230620 0.94802396808467
4.29313576297968
O 0.05088418510882 -3.85121869097521 -
2.77287580364438
C -0.12159745880407 -5.21403442075638 -
3.17880644207213
H -0.41332991402280 -5.14781347187976 -
4.23746365156270
H -0.95730832799170 -5.67303041640484 -
2.62515450246176
C 1.15055108228480 -6.02786813219772 -
3.01310445421855
H 1.98798509735607 -5.56448129510586 -
3.55820217180407
H 1.00211171146132 -7.04684549468793 -
3.40542910646269
H 1.43214210487875 -6.11315757769193 -
1.95185442120254

113
INT-4D

C -0.84873063164761 2.22068746904271 -
2.21346827751925
C -0.30509317869137 1.64412511119295 -
0.92151541901664
C 1.22900176116030 1.80348024227343 -
0.84558782458352
N 1.70453834079160 2.05430960980849
0.39155663819117
C 3.13836157082802 2.13990150361166
0.66500400545287
C 3.39648631224172 3.22640094317552
1.70879243386130
C 3.68137320411332 0.76708933081375
1.04518894215493
C 4.13844264888943 -0.09832305147322
0.03943413049787
C 4.55799695841623 -1.39402375879429
0.35306645107585
C 4.52021701151251 -1.84527731139911
1.67861263543280
C 4.07079787503854 -0.98645938351363
2.68570041447282
C 3.66125800071048 0.31044424998887
2.36853200350298
O 1.92275478767140 1.68514820774593 -
1.85065878263146
O -0.52606403025820 0.21466538423869 -
0.86726590111550
C -1.46357657967554 -0.28081403208928 -
0.02734997050943
C -2.80396974137941 0.12380239073775 -
0.03189866272468
C -3.70501214734989 -0.49852882570144
0.83323450441959
C -3.31132301320142 -1.50399049106032
1.71651901671260
C -1.96616338185053 -1.89376563655927
1.76334678956004
O -1.43279357958551 -2.81152722073436
2.57315194549108
C -2.05525797859932 -3.44600976457181
3.69688099423217
C -2.68486880570734 -2.50657833433942
4.72457842903327
C -0.90202182081797 -4.22127177517292
4.35063098268696
N 0.24622051484165 -3.52055046510674
4.45013554527922
C 1.46387984420369 -4.11218313875909
4.98088473475341
C 2.41317663940557 -4.49428157811007
3.84134405577311
C 2.06823189009919 -3.20933803731589
6.05013896542446
C 1.22473063104803 -2.65054198339885
7.02470800923189
C 1.73892388543718 -1.86515231770567
8.05542897077271
C 3.11369284536108 -1.61827182090581
8.13303724743754
C 3.96119248362904 -2.15324866326514
7.16228542645534
C 3.44127613836291 -2.94098893995501
6.12827076718971
O -1.06012091921046 -5.36230496264884
4.77589981830638
C -1.07816109942453 -1.28591203896128
0.86235593326088
I 0.96070083399535 -1.82695753363805
0.86918098265474
H -0.66824709425328 3.30681507020830 -
2.24680000611930
H -0.32906806135181 1.76314873871545 -
3.06689952259046
H -1.93040927750420 2.04397677758392 -
2.30516424693225
H -0.77465215389283 2.09544958718875 -
0.03452778355221
H 1.07636088173389 1.90164321514640
1.19375434340482
H 3.60644386295184 2.42891550510163 -
0.28681611590146
H 4.46912354420040 3.29086393898777
1.94791540019995
H 2.84996522725845 3.03089778972474
2.64394564222841
H 3.06193964104234 4.20195569837701
1.32454113127270
H 4.13780238589769 0.24861226280267 -
0.99638234155277
H 4.92147733374518 -2.05540393120808 -
0.43827496747810
H 4.84909489464733 -2.85878193799402
1.92340571314817
H 4.02530026981954 -1.32433783292130
3.72310476616573
H 3.30586025999001 0.95994474888239
3.16685019757048
H -3.13268249162049 0.90304132688890 -
0.71945046142213
H -4.75443208643623 -0.19431435210798
0.81575278528522
H -4.04016044994214 -1.98051608799781
2.36876214566191
H -2.78388744743603 -4.18900909174149
3.33902755965242
H -3.65541784574909 -2.11208018709542
4.39611342621529
H -2.02829712513645 -1.65055291390327
4.93152530081129
H -2.85598353077180 -3.08491385411729
5.64602225404258
H 0.30532806000266 -2.57274892372794
4.05575135148165
H 1.13780294529640 -5.04204164901783
5.47589887976777
H 2.76521226737816 -3.60409059137551
3.29815068964853
H 1.88065440702183 -5.14256964498102
3.12992270765266
H 3.28974915035535 -5.04814908673147
4.21097955537183
H 0.14950329958444 -2.83179264073698
6.96486741994924

H 1.06296210190500 -1.43980601646141 8.80146473564973
H 3.51822193029925 -1.00608914824115 8.94304036314560
H 5.03652630504165 -1.96054612726608 7.20485883101475
H 4.12563753395706 -3.35463891947401 5.38641119508037
C -2.86470594780596 -7.37824822267405 2.99990558114080
O -3.32941577352144 -6.42944485869523 2.04656035870668
H -2.29243438086533 -8.18349343737694 2.50510008537929
H -3.76409909254064 -7.81104109720634 3.45827501098380
H -2.24764995267274 -6.89446374299009 3.77337549310897
C -2.43904915314540 -5.79191916743977 1.25855344152637
C -1.04490708424755 -5.89432734756010 1.38482253824389
C -2.98451777180461 -4.95978469747198 0.26317325832834
C -0.22111306422587 -5.16688416344909 0.52419306261693
H -0.60144482001650 -6.50949995194918 2.16631100268043
C -0.75403793563738 -4.33965719452359 -0.47091155401915
H 0.86344873271982 -5.24622874837844 0.63122700565075
C 0.10011097864709 -3.59440057508818 -1.42836760413465
C -2.15322305467032 -4.25238993174683 -0.59128709434388
C 0.96142668954899 -2.59417870501025 -1.13438400516878
C 1.80388396451283 -1.84527135100966 -2.11716246063078
H 1.64209131596292 -2.27121493338727 -3.11687223198035
H 1.55147923436861 -0.77452447231569 -2.14351440463938
H 2.87517413911655 -1.92289937763533 -1.87439082122438
H -2.58561774799019 -3.60608778912075 -1.35861379747509
H -4.07110280163551 -4.88717776992802 0.18861177250922
O -1.24851648105852 0.24899580719570 4.31977375720117
S 0.03381028836364 0.42867458801371 3.61863003904070
C 1.12449247027630 1.34529828450456 4.70703772133356
O 0.75269369648273 -0.88502887745066 3.35972762001133
O -0.05862447395125 1.25933978328209 2.38037398032910
C 1.00764878758615 2.73887896569931 4.74115180528767
C 2.05093980744680 0.68979718823905 5.51569722004840
C 1.83511233302200 3.47556249789415 5.58503806335908
H 0.28065442226958 3.23373695875499 4.09458555533495
C 2.78788887526331 2.84116262706292 6.40123333327045
H 1.74630014558536 4.56578139679472 5.60747441363412
C 3.68843635611821 3.65271114577483 7.29739033020240
C 2.87816769842133 1.44350585839594 6.35222553055240
H 3.10315304755553 4.29614963282985 7.97552417112991
H 4.33274772554445 3.00830564449062 7.91359242255768
H 4.34234894894345 4.31877354864667 6.70873038031331
H 3.60998977937779 0.92788729078338 6.97745338604259
H 2.13617918721380 -0.39575704643265 5.47866288956141
O -0.08152151253993 -3.87450715061556 -2.75287547330785
C -0.22482248927759 -5.24047923919131 -3.15565550746051
H -0.52409176339167 -5.18312984925045 -4.21278247148577
H -1.04652069180349 -5.71868696421067 -2.59697111015245
C 1.06687562519086 -6.02474353346736 -2.99662807979229
H 1.88991772559511 -5.54306466440448 -3.54782058607396
H 0.93996946049060 -7.04777869856935 -3.38605763066656
H 1.35732929278541 -6.10116180377048 -1.93700005362067

113
INT-4E
C 2.00989971701373 3.78342996486355 -0.47835740227003
C 1.63109804632042 2.61112229211492 0.40839341521257
C 2.83929362060176 2.13753069346474 1.24873239804164
N 2.48337233341863 1.43328929507245 2.34026085684276
C 3.44148317190537 0.74451887377694 3.19916858403074
C 3.04021416103197 0.93472400353411 4.66263241191070
C 3.55761446426181 -0.71341813639058 2.76438964324587
C 4.48676380726791 -1.06041585084665 1.77218482815493
C 4.56298817148966 -2.36810810198540 1.28712256236879
C 3.70151105119300 -3.35270380630514 1.78229246836694

C 2.77833413455157 -3.01915734902793
2.77928551133061
C 2.71018754062794 -1.71065885738857
3.26840824161665
O 3.99828360334004 2.38590107604543
0.92722659793076
O 1.25012914548550 1.42256792738847 -
0.32409529387526
C -0.02661074776013 1.21026822083214 -
0.70561373122457
C -0.84328451282409 2.17723359701009 -
1.30612666980909
C -2.12404374395988 1.83262749279256 -
1.72776715799411
C -2.63803539055080 0.54585309695452 -
1.57223852876592
C -1.85258968348803 -0.43112847847958 -
0.94809506889272
O -2.24258041226870 -1.69177991483144 -
0.65659297703801
C -3.50199833281345 -2.21789034777666 -
1.09621264019497
C -3.53358101142411 -2.47756139168917 -
2.59413501540800
C -4.70656741530526 -1.38884784343085 -
0.59697855977185
N -4.55875381534482 -0.82326726429445
0.61841496936933
C -5.56618418770711 0.11579550899464
1.12298198379650
C -5.59843040577560 0.07818449322188
2.65053401648838
C -5.32677159264290 1.50522453372337
0.54264673843809
C -6.10550896637788 1.95547446517630 -
0.53284062272210
C -5.86829820663094 3.20712900817313 -
1.11015325780725
C -4.84373336782776 4.02246237686316 -
0.61987342037198
C -4.05264665582092 3.57172029694485
0.44151898371463
C -4.29196170073828 2.32333973949678
1.01661592153486
O -5.71910907117654 -1.31119961071482 -
1.28268286123516
C -0.55480689357485 -0.07856818592638 -
0.54657078699326
I 0.62033051461470 -1.53690166613859
0.41960301759082
H 2.73379326459860 3.48281487406882 -
1.24426943909494
H 1.13360328491243 4.22535494194789 -
0.97029294255984
H 2.47711424212831 4.56376587490343
0.14124023560961
H 0.79516185167705 2.86431902319272
1.08054652342205
H 1.51244950679245 1.10245054141248
2.41776831053688
H 4.41271124233545 1.22699109023756
3.01785197552524
H 2.00934147433896 0.59286149804019
4.84383214465589
H 3.08433717577848 2.00237291575703
4.92580914349017
H 3.71403644451973 0.37864518021665
5.33274488638770
H 5.14514982316010 -0.28478668990109
1.37310693882654
H 5.30117095827393 -2.62269573815917
0.52175140109609
H 3.75941501344598 -4.37668843873763
1.40407160122869
H 2.10794752154872 -3.78310205431517
3.18275284537149
H 1.96640858105170 -1.46116679381123
4.02578229129313
H -0.47099676126492 3.18832020852025 -
1.44935979603315
H -2.75803794084668 2.59294464050120 -
2.18644088065440
H -3.65227577893907 0.33287491564365 -
1.90182812317802
H -3.55451606279307 -3.18283241804066 -
0.56496968991741
H -2.71478478558071 -3.15640125699686 -
2.87556412815609
H -3.43395676389953 -1.54963374728224 -
3.17400677961416
H -4.49680491328356 -2.93338112200413 -
2.85890106626664
H -3.64484057517293 -0.80547291330021
1.07950270511525
H -6.52907684312001 -0.24279992925653
0.73251419805643
H -4.62061324474423 0.34284707037382
3.07641511148129
H -5.85543530022746 -0.93489451826162
2.99859862970404
H -6.35307481663488 0.78263933445703
3.03222766765223
H -6.89204358820105 1.30839807240744 -
0.92874862414172
H -6.48746481365471 3.54606451639213 -
1.94551475952758
H -4.65935695895093 5.00282795079175 -
1.06854099494809
H -3.22948102513271 4.18143407623033
0.82218747564591
H -3.63477526922676 1.98346567869632
1.81408505599484
C 5.37380817411478 2.79649275352000 -
2.06647478491778
O 4.21252719183412 2.53273659027098 -
2.84343513468354
H 6.17894722343764 2.07825316949667 -
2.30468290640392
H 5.14397061537024 2.76339581165268 -
0.98950675034660
H 5.70396300516998 3.80551009397409 -
2.34729172927352
C 3.58318652659958 1.34723934043783 -
2.69605237941915

C 3.99904292706571 0.33843256918034 -
1.81604878854333
C 2.43287916925936 1.15147358026674 -
3.48305715905593
C 3.26737100425802 -0.84350642640529 -
1.73427327892535
H 4.86788663141638 0.47731785655288 -
1.17589498985976
C 2.12004723921438 -1.04871402993385 -
2.50855810541969
H 3.58935505280430 -1.62142167719022 -
1.04155963322126
C 1.36034952326280 -2.31610860484461 -
2.41303250584652
C 1.71471672865691 -0.03199870546867 -
3.39267824098023
C 0.78804598216756 -2.80951500350438 -
1.29011572068859
C 0.07940843529764 -4.12243234856714 -
1.16879331293237
H 0.23834305021471 -4.70829515506524 -
2.08493678538281
H 0.45136991481773 -4.70781527691433 -
0.31058915992235
H -1.00370566903585 -3.98071149900900 -
1.02516797545337
H 0.81496094471651 -0.16986578008088 -
3.99709150284812
H 2.11991903924406 1.95573390498570 -
4.15147672689784
O -2.31940142074133 -0.19322015043082
2.28811666944336
S -1.20823336330580 0.69276547412228
2.71755173742399
C -1.30642454962845 0.80716015462665
4.49992513474604
O 0.13795030372200 0.01806763881156
2.48825830556976
O -1.21826026661070 2.07465112001752
2.18907758918588
C -1.73120595645172 -0.30083303390652
5.23739320015513
C -0.88347456990958 1.97247430588408
5.13810557646326
C -1.72346396412112 -0.23463091442703
6.62928056091798
H -2.06888453164520 -1.19789395723936
4.71505541678803
C -1.29844948012562 0.92505884379430
7.30059692331316
H -2.05687033778932 -1.09950856859643
7.21034938178076
C -1.32245995613301 0.99185686813086
8.80632750002999
C -0.88138251385652 2.02311130430077
6.53337217387014
H -0.68237795059701 1.80214111807671
9.18640615906882
H -2.34616385880847 1.17829818085556
9.17610055535735
H -0.98400118473770 0.04559369842160
9.25750734213453
H -0.55139525020509 2.93615546772577
7.03700424196360
H -0.56872460558480 2.82802539720025
4.53825255361481
O 1.16146655238330 -3.02322282876758 -
3.56204807563954
C 2.24592148936554 -3.14871602549714 -
4.49122649192804
H 2.62701967305741 -2.15205693395165 -
4.76715771350543
H 1.78830144383187 -3.59653444511231 -
5.38579699866445
C 3.36247413899246 -4.02498314571365 -
3.94968886457280
H 3.82776790302287 -3.56955247437429 -
3.06122290499021
H 4.14688636499692 -4.15444404508817 -
4.71272470854545
H 2.98288337499186 -5.02084891415937 -
3.67109006557171

113
INT-4F
C -0.97947798015956 2.77504942680937 -
1.62645624281208
C -0.31970630979489 1.70913442406148 -
0.76965307875310
C 1.20618213192199 1.73859231719594 -
0.94814951609532
N 1.88589511515585 2.07003598472137
0.16707050278260
C 3.34508832456038 2.10624784112260
0.20208254054459
C 3.80096924946149 3.14820848998029
1.22287821163209
C 3.89377862522691 0.70621559353836
0.45487835280700
C 4.25952676206847 -0.09626884849952 -
0.63533340550196
C 4.68404718805622 -1.41439736638835 -
0.44291006569229
C 4.75574951862929 -1.94663775001280
0.84838260839685
C 4.40142432939166 -1.14994360648676
1.94201921976166
C 3.96494132490038 0.16294531942024
1.74582390710577
O 1.72090872297313 1.49905132871360 -
2.03561646601799
O -0.76518830298020 0.39368013810262 -
1.19715222236817
C -1.61113320452998 -0.24401157754550 -
0.34690865835095
C -2.99639604165015 -0.06147668057352 -
0.42227788128537
C -3.82742371722846 -0.74929693963620
0.46333755823474
C -3.31277106001898 -1.59522305909178
1.44504529772130
C -1.92719455687365 -1.77185907340188
1.55467746211633
O -1.30369289839870 -2.53534613799172
2.45659460942711

C -1.90365109255777 -3.08374850199648
3.63522915257701
C -2.62758465431710 -2.06656292215270
4.52128356067104
C -0.73731964170933 -3.72800097863291
4.39893820921451
N 0.41936710005976 -3.03121327392790
4.38614134960645
C 1.64262393115303 -3.60013327727114
4.94748706629596
C 2.52537277925474 -4.16710855299004
3.82433293166328
C 2.40414977097428 -2.61898980511655
5.82319762657567
C 2.64543233136779 -2.92219415151312
7.16871531058260
C 3.40120053172868 -2.06266173983017
7.97310085087317
C 3.92954747504986 -0.88708039181912
7.43568920393751
C 3.68220323565345 -0.56879446773983
6.09767089930625
C 2.92178448541935 -1.42380800718914
5.30043067055423
O -0.90389296545577 -4.78573265775835
4.99936145549045
C -1.10879319541375 -1.12375141438563
0.61642472640249
I 0.95863922388825 -1.56675127872132
0.56919638840228
H -0.62812389161103 3.77430563639928 -
1.32363835566890
H -0.71124617698235 2.61058142624701 -
2.68040358235852
H -2.07388855879212 2.74417622936134 -
1.51578748788020
H -0.57796476911236 1.83606556229746
0.29384947207664
H 1.39579374584409 2.09367878995912
1.07332551975440
H 3.67166897459552 2.40970174721564 -
0.80366873706138
H 3.44908241810253 4.14733630481040
0.92389385489767
H 4.89887613157791 3.16738855015034
1.29699819266279
H 3.38727131280731 2.93625572153661
2.22092302229453
H 4.17613730397172 0.31541558388505 -
1.64335558178788
H 4.96581332208853 -2.02674747500842 -
1.30374061178811
H 5.09577870720621 -2.97380639243884
1.00458986669450
H 4.46368379598917 -1.55351905362213
2.95410132592635
H 3.65252032467878 0.75606221167684
2.60672170660950
H -3.39772019460380 0.60956776465520 -
1.18254177912077
H -4.91009364691382 -0.61924713762083
0.39239499014737
H -3.98258020686601 -2.12406932207140
2.12042880794220
H -2.57672900087266 -3.90866570309944
3.35631724637321
H -3.65671162638129 -1.87906922861136
4.18772199695760
H -2.09971442816169 -1.10366808972174
4.51889630212003
H -2.67928248027386 -2.47014696027542
5.54386575909883
H 0.48671258291867 -2.17369988813064
3.82556278210382
H 1.30709071880894 -4.43396124605697
5.57905509418125
H 3.44021138883982 -4.61952407985662
4.23798071212217
H 2.82357977686307 -3.37634863322162
3.11858843656894
H 1.97610872991742 -4.93847624611185
3.26215886517365
H 2.24146153253677 -3.84663600365351
7.59172805914048
H 3.58154930433038 -2.31755316433892
9.02099562347098
H 4.52448652464484 -0.21507048600550
8.05961621013048
H 4.06728708793107 0.36266972969194
5.67651221800221
H 2.69313429734120 -1.13844797535805
4.27366653599060
C -2.28372498039353 -7.21740409902143
3.41472107628815
O -2.88104963962619 -6.41194492605160
2.40572424505041
H -1.70244295466404 -6.60285752715163
4.11933432196099
H -1.64449458091091 -7.99891578098827
2.96596074443587
H -3.11455492943185 -7.69979836489633
3.94685934787697
C -2.09533467155030 -5.76251887262083
1.52332875316164
C -0.69399581959847 -5.70891596268965
1.58793196887854
C -2.76507382798467 -5.08733236425500
0.48560983846993
C 0.01373627050174 -4.98940133582781
0.62291704460986
H -0.15621244448464 -6.20520704643856
2.39464319142140
C -0.64373101271027 -4.31957321035938 -
0.41503196842743
H 1.10420491853997 -4.95174471178724
0.68078166636324
C 0.07764947162110 -3.59739218583635 -
1.49170010703347
C -2.04845696878773 -4.38504694732911 -
0.47018011779771
C 0.86301467279412 -2.50406398876401 -
1.36042097376580
C 1.55112738393343 -1.77709070645140 -
2.47037208018920

H 1.19610733362478 -0.73914260481785 -2.55997366484955
H 2.63882064932611 -1.73195090127170 -2.30782418776796
H 1.35795294353226 -2.30895924364860 -3.41203186799875
H -2.57676473214677 -3.86233719939156 -1.27078808623725
H -3.85537725893420 -5.13340670532656 0.45884334696273
O -1.29772776237942 0.63901491216340 3.33960562598355
S 0.16780083040883 0.79712246025810 3.39538358628839
C 0.62295881832767 1.05548832810889 5.10882721438115
O 0.89584315472213 -0.48159523766909 2.99462183149598
O 0.69931652008161 1.97693814888196 2.65328954475623
C 1.38124057849622 2.16797835085626 5.46456397839066
C 0.22172847175925 0.12939623530242 6.07729945970254
C 1.74693647906234 2.34811925005672 6.80179211974814
H 1.67258530655147 2.88352759559325 4.69457570176590
C 1.36401221021987 1.43095741368495 7.78842270595449
H 2.34480075221557 3.22034997503731 7.08099889443001
C 1.77495198877534 1.60500234210805 9.22671720403091
C 0.59208865741936 0.32184946151776 7.40292930720777
H 0.89545311429296 1.67211673226330 9.88889151653559
H 2.37083617794643 0.74219692524439 9.56869313347017
H 2.37742918748329 2.51404777416663 9.37116378997147
H 0.29338100430382 -0.41395525656718 8.15399228359923
H -0.35840599147580 -0.74824538046702 5.79587945184033
O -0.16974200760290 -4.01805275306654 -2.76788624673453
C -0.17242592551301 -5.42458084877687 -3.03697585949940
H -0.53709838148639 -5.50353570036468 -4.07186040532662
H -0.89786336273184 -5.93631547117517 -2.38255995840402
C 1.21016882176180 -6.03864436248224 -2.89636812615913
H 1.56846476880823 -5.97547290556032 -1.85682462673004
H 1.93699296833532 -5.52461215656598 -3.54490071450241
H 1.18275500833742 -7.10308676429777 -3.17986928119178

113
INT-4G
C 2.67231379782773 0.58789260752246 -2.26595269086871
C 1.21339510842786 0.62819998993152 -1.84202597703053
C 0.85299718448556 1.99649351741098 -1.22412913682773
N 1.11665837004511 2.13290589100978 0.08676613886543
C 0.70107684420316 3.32179393209932 0.83277096864789
C -0.75583078663556 3.18604765902339 1.28317049679080
C 1.69989131760686 3.58661100605827 1.95292239813141
C 1.32417292745605 3.67693492062982 3.29783396541071
C 2.27386055861153 3.94662720105039 4.28869448931783
C 3.61545791559229 4.11952254425901 3.95200160872385
C 4.00537344757818 4.01769803189335 2.61201455400609
C 3.05506444428710 3.75545766743860 1.62633905400846
O 0.38392908845379 2.86580437207260 -1.95002951049163
O 1.01238465168913 -0.47206092524014 -0.94526900247180
C -0.19304912558976 -0.64749610149671 -0.36895275124236
C -1.36035622971734 0.04229356057353 -0.73150111860486
C -2.54248604824968 -0.19706687508386 -0.02944049754441
C -2.60662180258084 -1.09241214357793 1.03555421214241
C -1.45014855965941 -1.78678742921053 1.41496091301400
O -1.37591347068178 -2.68355720325103 2.40352536320550
C -2.27241414390574 -2.75846689680339 3.51910569237894
C -2.48268931570958 -1.42865173776288 4.24090039906201
C -1.62117307210579 -3.77755391247344 4.47059010204392
N -0.28470285997423 -3.66028593955784 4.56486227997365
C 0.55859273660674 -4.51908751553726 5.40139648084044
C 0.43561711081347 -5.99456691209880 5.00858220689025
C 1.96853280802825 -3.95863629746676 5.30707192698412
C 2.85368821972157 -4.35804207682150 4.29714651478241
C 4.09264154437593 -3.72948220081127 4.14544496255193
C 4.46286781954220 -2.69323174623844 5.00562491734387

C 3.59355775726721 -2.29742325164125
6.02410492361284
C 2.35683421308453 -2.92543668636566
6.16823226437192
O -2.30376213169417 -4.58772897443977
5.09248384740083
C -0.27723635526513 -1.58005654373730
0.67369367361255
I 1.41109770073207 -2.75692145565108
1.15415905162843
H 2.89874612434421 -0.34378239426658 -
2.80666305694322
H 2.88064378769790 1.43874069379397 -
2.93082791139241
H 3.32517285325833 0.65662287879146 -
1.38270991949786
H 0.55697618782453 0.51552259237187 -
2.72037243408106
H 1.44972960037991 1.34297696358431
0.65512863932449
H 0.76155089511482 4.15872622398649
0.11736352534511
H -1.39330899968971 3.04248764503335
0.39866655870776
H -1.09586609635735 4.09691316443520
1.80063717595383
H -0.87713108779413 2.32392891140880
1.95532398360509
H 0.28701806472277 3.51449273292297
3.58841229585458
H 1.96041244124949 4.00424080375760
5.33362240070442
H 4.35674859334723 4.32408070363374
4.72849124492125
H 5.05542103926364 4.14717573121345
2.33491045481762
H 3.36317521935092 3.67361566346956
0.58038968994564
H -1.34203823673360 0.78333393270891 -
1.52921443076454
H -3.44432628923379 0.34684950815159 -
0.32055574720205
H -3.54330377363469 -1.26494059985404
1.56347473659711
H -3.23022951394964 -3.19625649537874
3.19547487705952
H -3.13177092817147 -0.74490900825483
3.67800552388592
H -1.52547807364624 -0.91450035342593
4.40247746852924
H -2.96552712167581 -1.63585846154638
5.20825534604385
H 0.17234351490149 -2.86704051481606
4.10144319538160
H 0.21782348249843 -4.42489568974522
6.44863321848925
H 0.66506415825679 -6.14255024095860
3.94195866822239
H -0.59127637188955 -6.33784859564346
5.18907991978791
H 1.12925692914711 -6.60788444048030
5.60423967423815
H 2.57428091788582 -5.16473009667183
3.61603841589655
H 4.77325001546646 -4.05209950516701
3.35232995994867
H 5.42592461132067 -2.19182470136360
4.88322095503773
H 3.87029262081516 -1.48032735232730
6.69271432397910
H 1.66765983852240 -2.59332419353405
6.94978657798476
C -4.30181084812286 -6.30466224633671
3.20699655873768
O -4.27656342756394 -5.46272033015820
2.06019396076198
H -3.96823596195223 -7.32647783109350
2.95199897219429
H -5.35090222371611 -6.34333896674801
3.52956523186778
H -3.68138957591985 -5.89054072063111
4.01759252938185
C -3.12572254924629 -5.30711862568337
1.37564222368014
C -1.88443435055203 -5.83150721888841
1.76907141545528
C -3.21156911648329 -4.54645253053335
0.19447387332091
C -0.75405711964187 -5.58180211887821
0.98961543065841
H -1.78786121479846 -6.40606113160879
2.68889877138869
C -0.82914843963666 -4.82455419962292 -
0.18492939996698
H 0.21081664620366 -5.98403401312224
1.30787286185661
C 0.35490509339135 -4.56323146371409 -
1.04135790035687
C -2.08194112674337 -4.31596636911932 -
0.57483573356116
C 1.43685007781896 -3.82592186779913 -
0.69893514633840
C 2.61242765583735 -3.54100272507162 -
1.58144873540536
H 2.59904189064208 -2.49296611662857 -
1.92215289319514
H 3.57026931057823 -3.70277165046147 -
1.05883551297920
H 2.58050839139367 -4.19633431385742 -
2.46324410549197
H -2.15878166784834 -3.71423741184002 -
1.48355447360674
H -4.18459495488037 -4.14208133158128 -
0.09030021254238
O -0.01842708225671 0.65329469309756
3.47657518793477
S 1.29229438192019 0.00530481782829
3.26277592196673
C 2.38277889847802 0.55816717548847
4.56326458468795
O 1.21223688948934 -1.50618432988550
3.42095686760859
O 1.98891791241140 0.32516197421679
1.98471305455931

C 1.86275905102774 0.83787745885794
5.82574645680221
C 3.74456045076226 0.69167969818299
4.30251286704513
C 2.72466131286417 1.25640492961022
6.83902552730300
H 0.78965368706098 0.74558315421454
6.00163628501250
C 4.10147449413050 1.40518005701107
6.60477833109581
H 2.32096897979298 1.48107461782580
7.83048705150253
C 5.02161043363633 1.90757609705172
7.68795050491557
C 4.59411257739524 1.11286735135658
5.32357870598097
H 4.61999569898475 1.69943408790859
8.69134064772363
H 6.02094027839328 1.45079476127396
7.61537247102278
H 5.15834043988698 3.00092876055183
7.61052567726944
H 5.66210812534157 1.23072194074001
5.11902538730729
H 4.11901404529501 0.48744413037300
3.29925701766379
O 0.30392075842360 -5.01229744164573 -
2.32778067931712
C -0.27971325614308 -6.29313033428962 -
2.59722345068568
H -0.36606855809271 -6.32697206746135 -
3.69335705713094
H -1.29660934182878 -6.34362104164960 -
2.17532471534509
C 0.57992534692481 -7.43448265668238 -
2.08153782708660
H 0.64738287126811 -7.41565110623363 -
0.98259598104203
H 1.59974036942559 -7.37680931334712 -
2.49368506593977
H 0.14122809993148 -8.40154078326690 -
2.37571822936015

113
INT-4H
C 1.96399694236276 3.92511248691969 -
0.54596531075160
C 1.54227552777004 2.73674896807424
0.30124224896395
C 2.69385614555211 2.26772035237912
1.21568092305620
N 2.28038771175981 1.75735449060841
2.39397086323842
C 3.18424352608069 1.07578807028864
3.31750408871960
C 2.95882126158331 1.57550695164284
4.74463379644638
C 3.02417815133331 -0.43147499447550
3.15012169194831
C 3.82842788709791 -1.11172761379155
2.22369359136412
C 3.63641726576123 -2.47201198629860
1.97037887795935
C 2.62708091253896 -3.17478345899256
2.63748689313774
C 1.82912016085631 -2.50805153523590
3.57373784405716
C 2.03010219306724 -1.14910269527126
3.83132416748739
O 3.86775953168853 2.34822861856887
0.86610327439839
O 1.24144418025351 1.56145105131974 -
0.48226987304517
C -0.00363757115709 1.35118113588519 -
0.97026383035941
C -0.64825092378032 2.22977335696167 -
1.84834123296394
C -1.89796489559274 1.89101927793876 -
2.36344501274436
C -2.53433991299763 0.69504962522587 -
2.03821892623076
C -1.91279408654027 -0.19864391597502 -
1.15279437426586
O -2.42514180756140 -1.36982263805651 -
0.72238756850309
C -3.75636885109164 -1.79151275422282 -
1.05937284551321
C -3.83698645493566 -2.37163260595375 -
2.46153661252310
C -4.81035812838766 -0.68984392854347 -
0.79600543049538
N -4.63563734477080 0.00341602234018
0.34380254196682
C -5.45780562370525 1.17996205188208
0.64053881878147
C -5.59851646440466 1.35406468533631
2.15302424538399
C -4.89757509114194 2.40720342239835 -
0.07255530685774
C -5.47081015129649 2.82814144989616 -
1.28086096457896
C -4.95805941038736 3.93273562748700 -
1.96806112679274
C -3.85935499073060 4.62767794547674 -
1.45501492395578
C -3.27300337491310 4.20177909764608 -
0.25920511923162
C -3.78438286078613 3.09923943970765
0.42847018244757
O -5.72336885753351 -0.50695365213461 -
1.59436679292860
C -0.65058773846242 0.15041790740812 -
0.65001312024395
I 0.33935158431255 -1.19406232096986
0.63219356567796
H 2.72790131444173 3.62218445182896 -
1.27349740762180
H 1.10979890353785 4.36285053826312 -
1.08011246682383
H 2.39193168877462 4.70625778645015
0.10116095644219
H 0.65673765068054 2.97087995664819
0.91398322813901
H 1.27395535967118 1.57981172940240
2.51095270941593

H 4.19854985521379 1.34784811530788
2.99307235137113
H 3.62032963547298 1.05152935452808
5.45206331793278
H 1.91901920547198 1.41645060517487
5.06910047091452
H 3.16648550131248 2.65469827836947
4.80106824807205
H 4.60167613077385 -0.55759742599656
1.68576457345722
H 4.27692798785275 -2.98937452016521
1.25114956029244
H 2.47409747132390 -4.23900045668336
2.43975534496684
H 1.04247390964229 -3.04371486938501
4.10973587180877
H 1.39018968180831 -0.64842631842225
4.55732190802744
H -0.15968536380442 3.15662909742863 -
2.14036609985333
H -2.40419490078922 2.58394668281939 -
3.03697036058641
H -3.51947465072047 0.48397119419036 -
2.44808799996714
H -3.94430808932639 -2.59197947500261 -
0.32503927117169
H -3.12283756060827 -3.20234635734430 -
2.56557626730017
H -3.61598928858835 -1.61920833574691 -
3.23112706288280
H -4.85528180048510 -2.74091264648465 -
2.64371295696691
H -3.81382652593089 -0.15533140262087
0.93891273984504
H -6.44622064811693 0.97285668381771
0.20583051395549
H -6.24709375705594 2.21586919078087
2.37283537236805
H -4.62829838796864 1.52358534940896
2.64008119501369
H -6.05127235438191 0.45276397952210
2.59550553805316
H -6.31875676618880 2.27187297405606 -
1.68767387037773
H -5.42219907763487 4.25307168997205 -
2.90519865162850
H -3.46052400152740 5.49623127023475 -
1.98690709709187
H -2.40698054978274 4.73197064013229
0.14659053182755
H -3.30233190774768 2.78059173235487
1.35426180786274
C 5.34693048000330 2.64971402397694 -
2.07516518978031
O 4.20014700125554 2.31951984410986 -
2.84729460300306
H 6.15372879245666 1.91103082609281 -
2.23213577615269
H 5.09743955324190 2.71167815646084 -
1.00430705902107
H 5.68693061547630 3.62859702907101 -
2.43913675911868
C 3.56794997077955 1.14998679260591 -
2.60939796334316
C 3.94239600838315 0.24062228668968 -
1.61021471217291
C 2.45884847198703 0.86561284358375 -
3.42687756821778
C 3.18984187337363 -0.91524105767271 -
1.42002913311962
H 4.77951881430847 0.45178969835538 -
0.94824641569989
C 2.06942924333023 -1.19702205857605 -
2.20958289085881
H 3.46754085410181 -1.60897217190768 -
0.62607744275375
C 1.26583578153702 -2.42078837883102 -
1.98101721657501
C 1.72195908560900 -0.29323246143367 -
3.23015532392339
C 0.58554007819267 -2.71548851571816 -
0.84838202909890
C -0.19858490330569 -3.96508344594513 -
0.59539152748667
H -1.28218337984665 -3.76394862865583 -
0.61518740806562
H 0.03422524170163 -4.70665476941670 -
1.37250656223840
H 0.03910393660298 -4.40225199593644
0.38924625217689
H 0.84584795788313 -0.49206391044818 -
3.85204933871564
H 2.18397065106256 1.58883771410856 -
4.19686566710098
O -2.53031636031465 -0.20798805739915
2.21708964111001
S -1.65033945838073 0.75911931361819
2.92353920929186
C -1.32842753397376 0.04592314787957
4.53360670364599
O -0.27499579762038 0.79594799255908
2.27125660552282
O -2.18891857216056 2.11315423653156
3.14942981502062
C -1.45268708410660 -1.32965132691546
4.72090893165639
C -0.86507400686420 0.86890660423104
5.56306346775607
C -1.08568099709019 -1.88756825567368
5.94749347778242
H -1.83640136662717 -1.94668886499044
3.90679473471265
C -0.59476639646176 -1.08920652300528
6.99151016036098
H -1.18372411920715 -2.96649287394638
6.09878820528316
C -0.21092638679218 -1.69031924910087
8.31929328517584
C -0.49724859086104 0.29741569892848
6.77997924735788
H -0.11485268172342 -2.78442257381678
8.25616725375739
H 0.74551268946940 -1.28263573496644
8.68412616375988

H -0.97105171915490 -1.46666461479812 9.08796550234108
H -0.12995129737049 0.93932568681094 7.58595798841108
H -0.80226665232646 1.94694614020835 5.40202034332801
O 1.13179121560519 -3.29637935711618 -3.01771561567165
C 2.26325565376562 -3.55342716849925 -3.85874878559678
H 2.66277365921281 -2.60692985444980 -4.25753030102634
H 1.85187011106565 -4.13000583824103 -4.70047859618844
C 3.34669457867734 -4.33671673034753 -3.13672150909404
H 3.77073925754501 -3.75068625818474 -2.30651424206793
H 4.16628164045909 -4.57881228374279 -3.83233533928668
H 2.94690797993950 -5.27909350439622 -2.72996098361632

113
INT-4I
C 2.07490046326455 3.77162351045422 -0.40141343544858
C 1.67968425579172 2.58673952972089 0.46097815514739
C 2.88209409019729 2.07430471408018 1.28644414477710
N 2.51696732299888 1.35326395916201 2.36399635489182
C 3.46467940653979 0.62703518861798 3.20331494747670
C 3.08944487713968 0.81377227089590 4.67427278187016
C 3.53006403585721 -0.82894774681426 2.75177700156316
C 4.43454709716936 -1.19331650090392 1.74311725942667
C 4.46391454446188 -2.49714405933588 1.24291285146734
C 3.57907849655332 -3.46029694833092 1.73919444220504
C 2.68011235189258 -3.10959731448584 2.75234226199507
C 2.65901484952325 -1.80524140294576 3.25645476622304
O 4.04414441567547 2.30815652098183 0.96522830323968
O 1.27750631051574 1.42063909283086 -0.29585779217741
C -0.00283978728498 1.23842674479105 -0.68089225822584
C -0.80248546552247 2.23124456382762 -1.26183029081225
C -2.08843978173456 1.91711668029594 -1.69126115858341
C -2.62405495526010 0.63627892532609 -1.56258253670219
C -1.85592788531321 -0.36638347410931 -0.95795806017487
O -2.26731641643808 -1.62604597133462 -0.69266935275532
C -3.53441371789781 -2.12205717161388 -1.14498672314710
C -3.56782678672528 -2.34875630297457 -2.64823104609078
C -4.72555954703839 -1.28310021442435 -0.63025508703088
N -4.57008191630435 -0.74542731591996 0.59684161686798
C -5.56008056877986 0.20263949909822 1.11906196959287
C -5.60114147461443 0.12822949585018 2.64507309781501
C -5.28752752179128 1.60025360361364 0.57358436655462
C -6.04313392187884 2.08891558016586 -0.50175524119328
C -5.77417714228980 3.34808024244285 -1.04802432702203
C -4.74042383654360 4.13216291099806 -0.52685516486846
C -3.97227212318000 3.64312205080909 0.53437084552133
C -4.24416513289644 2.38792904025320 1.07919918335371
O -5.73493695889427 -1.17285357679245 -1.31615933589276
C -0.55298200697913 -0.04409763487044 -0.54815413794647
I 0.59553250677723 -1.54239032087030 0.38871802621655
H 1.20412477200100 4.23853701262991 -0.87979941449894
H 2.55755172959685 4.53042416144681 0.23294439317393
H 2.79041177168237 3.47545408348415 -1.17688311123989
H 0.84983418168966 2.83887725762929 1.14100800739375
H 1.53955418201702 1.04124409584461 2.43894482376408
H 4.44750464760308 1.08182697786278 3.01336196738707
H 3.75414593515595 0.22949491835729 5.32937222309226
H 2.05078708387672 0.50258049792411 4.86594004148033
H 3.17100311834387 1.87666870820197 4.94762126966685
H 5.11117887093810 -0.43421152921827 1.34276839063630
H 5.18378118286435 -2.76584917391813 0.46504738437564
H 3.60052599663389 -4.48136576235092 1.34942349906347
H 1.99134307021876 -3.85671128571369 3.15624172318857
H 1.93334284747648 -1.54173148636939 4.02646724510525
H -0.41304639817982 3.23850098488219 -1.38464695183983

H -2.70905656079321 2.69718478691528 -
2.13476169810974
H -3.64123395365562 0.44706888578558 -
1.89739724364768
H -3.60380579961776 -3.09710983974111 -
0.63454204555107
H -3.46063254269104 -1.40912102171288 -
3.20736785280931
H -4.53472246434450 -2.79053352012893 -
2.92328635730938
H -2.75459427006111 -3.02811402821240 -
2.94415834654976
H -3.65722648048839 -0.75518925429650
1.06018424056723
H -6.52803443994019 -0.12558454607486
0.71478250406263
H -4.61981747786426 0.36069743804374
3.08165734525877
H -5.88233614998266 -0.88712885083659
2.96666678757304
H -6.34183037454998 0.83971845865221
3.04082572325725
H -6.83644730829851 1.46575587668372 -
0.92174369139063
H -6.37563855613498 3.71739740805741 -
1.88345163939442
H -4.53083657086357 5.11801647048385 -
0.95181868012369
H -3.14200049602623 4.22753929306203
0.93842665107592
H -3.60598089262513 2.01755286822789
1.87820578660322
C 5.40793525326767 2.76219810658296 -
2.03162241090382
O 4.23928671237043 2.53301524013859 -
2.80854282502107
H 5.75313066034832 3.77130947160310 -
2.29333931324592
H 6.20032776247465 2.03608267893568 -
2.28797823411196
H 5.18224435371261 2.71077386290170 -
0.95445427098509
C 3.59167690084054 1.35493809879795 -
2.68235249594126
C 3.99477038374810 0.32247903312366 -
1.82413812655468
C 2.43552787586521 1.19284756214728 -
3.46842751445762
C 3.24480338701822 -0.84913536580190 -
1.76250596363123
H 4.86819041837151 0.43549647664999 -
1.18504120400852
C 2.09147103874528 -1.02107646922543 -
2.53611203227278
H 3.55685214232996 -1.64550941685086 -
1.08641512308622
C 1.31156132320457 -2.27759226380583 -
2.46185499310275
C 1.69905514742255 0.01927928969594 -
3.39829132732777
C 0.73692183782225 -2.78383966695128 -
1.34592969036379
C 0.00975741509030 -4.08841181082994 -
1.24653622520743
H -1.07097050939645 -3.93434823886983 -
1.09746909905668
H 0.15820560461282 -4.65969288353303 -
2.17357397632785
H 0.37556710190430 -4.69444529707239 -
0.40008580624652
H 0.79470232592462 -0.09225301156065 -
4.00126308003565
H 2.13250770738802 2.01493841626550 -
4.11951323815680
O -2.31842470957819 -0.18205748531599
2.28011733797577
S -1.19268429597949 0.67545730025884
2.72919868926217
C -1.28983613727615 0.75108559025561
4.51379645981255
O 0.14212176471446 -0.01624531634375
2.48506055284394
O -1.17899332449567 2.06888606549177
2.23228253735493
C -1.73557567472300 -0.36576082316209
5.22581238058442
C -0.84755644662724 1.89380656892030
5.17843895741603
C -1.72863196805868 -0.33119276261812
6.61847641215666
H -2.08943506173182 -1.24403189770611
4.68266476614896
C -1.28321660860641 0.80534232876111
7.31645161910908
H -2.07941267967058 -1.20242171792308
7.17961424101515
C -1.30190650563434 0.83254709367410
8.82354459701152
C -0.84640634075025 1.91280113248012
6.57490014087207
H -2.33583075848858 0.89904865942555
9.20459042527616
H -0.86147803066016 -0.08455208817407
9.24739743428309
H -0.74535695827938 1.69439077144739
9.22063621345442
H -0.50210122332151 2.80857322992328
7.09941574797783
H -0.51804507526640 2.75753869754984
4.59856404754037
O 1.09516322604061 -2.95858396935085 -
3.62352551957934
C 2.17239515330178 -3.08314531129808 -
4.56111545788858
H 2.56638124931909 -2.08731316575403 -
4.82160339823828
H 1.70324385390788 -3.50823759504721 -
5.46079267989566
C 3.27922093752673 -3.98508004352901 -
4.04195892557355
H 2.88686615897671 -4.98036214996377 -
3.77943400154941
H 3.75566207246466 -3.55253516321485 -
3.14804036770763

H 4.05756832185830 -4.11173758021859 -
4.81163596201249

113
INT-4J
C 3.07206585967121 3.82053961363346 -
0.71585488394380
C 2.22049461713892 2.79460123083850
0.00927172267974
C 3.05221325579938 2.00856557577654
1.04665005205399
N 2.30793914838948 1.43714293334116
2.01689115776114
C 2.86456727702999 0.52332318253197
3.01117362642671
C 2.49857600516264 0.98903675405317
4.42188678496147
C 2.42061367116424 -0.89999243908843
2.69155001399546
C 3.20817075975035 -1.69688518868321
1.84797501787017
C 2.77733460201962 -2.96524914524860
1.45113896611928
C 1.54501576988050 -3.45903977046836
1.89333868723464
C 0.76077671586103 -2.68094714820743
2.75187134820243
C 1.19891753354845 -1.41428855702515
3.14836499182769
O 4.27370739516266 1.91610670188383
0.96677854382878
O 1.70854033090040 1.75450386549191 -
0.86051472200495
C 0.52978112456877 1.88927114647597 -
1.50326584190807
C 0.18871057357468 2.99063153080890 -
2.29971965469744
C -1.02672513375587 2.99893006630801 -
2.97884676109052
C -1.93460500281928 1.94185854224650 -
2.89269561995293
C -1.63503857703957 0.84575684535263 -
2.07522780126908
O -2.40314731456748 -0.24041238584282 -
1.89884149863416
C -3.84583633768654 -0.30254436212104 -
1.89928063658940
C -4.59360270529506 0.98998676892331 -
1.58437528307907
C -4.17367463821803 -1.40578577395766 -
0.87274109133024
N -3.73779987045877 -1.17224785971688
0.38609951134763
C -3.97718125970555 -2.15394746310080
1.44394459004884
C -3.08659039377609 -3.39487113297569
1.26628185049201
C -3.79551628980116 -1.53618422804419
2.81732654059928
C -4.84695455105514 -1.54192040641632
3.74281005961867
C -4.66591024086329 -1.04644787549320
5.03825431327065
C -3.42619326866181 -0.53260383353083
5.42370248518633
C -2.37655780743718 -0.50581367452851
4.50196872405864
C -2.55850256757458 -1.00435228132249
3.21235264503935
O -4.78845795203774 -2.40898094566138 -
1.21263465474383
C -0.39121817777040 0.83801504120036 -
1.42129434270303
I 0.08151005585458 -0.82563364691990 -
0.21826768319372
H 3.82279366721148 3.33379895035284 -
1.35005859178688
H 2.45791278439701 4.48954503521607 -
1.33369088228208
H 3.60043758975488 4.43989463981540
0.02469150021463
H 1.35562558118990 3.26507428092930
0.50282898401900
H 1.28168066742256 1.44056528136736
1.92682736264967
H 3.95513272041360 0.57784242210022
2.88450455270414
H 1.40765028359974 1.07163040418129
4.54773975930497
H 2.93248714312655 1.98255962069443
4.61137192199318
H 2.88020307809559 0.28778534350190
5.18032617439721
H 4.16314306105367 -1.30491988552803
1.48856719464534
H 3.40553882925522 -3.57518270619879
0.79637559932682
H 1.20467280739545 -4.45044141038623
1.58345854672024
H -0.19794370482246 -3.05807588313547
3.11557389076968
H 0.56498820846492 -0.81496674350051
3.80081233120243
H 0.88203481286855 3.82270213210725 -
2.39748593293009
H -1.27348662167066 3.85527924115671 -
3.61130804807202
H -2.86310435081992 1.96428468554951 -
3.45920736580549
H -4.15978281365221 -0.70961098773992 -
2.87301309902735
H -4.63917940403296 1.67084134581167 -
2.44428097026336
H -4.14135451995311 1.51533507901646 -
0.73245511372818
H -5.63028183906470 0.71978130266411 -
1.32869703767433
H -3.27754591618412 -0.28288592422729
0.61155912578390
H -5.02304561953757 -2.48781994101663
1.34389343926427
H -3.27377508281176 -3.84792315143780
0.28268767033512
H -3.30869328932072 -4.13957080834044
2.04663878220591

H -2.02225511914427 -3.12332536781057 1.34149735287531
H -5.81875886356560 -1.94744500157923 3.44697850262195
H -5.49823279451628 -1.06374218155921 5.74710262086349
H -3.28107442698440 -0.13828166269242 6.43230467808718
H -1.41545562720250 -0.07003829716088 4.78303968689805
H -1.73123812035844 -0.94604167808251 2.50634726441972
C 6.28066147824670 1.87751980036014 -1.66642715019338
O 5.25462362224345 1.88766639609816 -2.65052663276373
H 6.89083124118364 0.95946289199838 -1.74330195717412
H 5.85760442659398 1.96499624134230 -0.65318236793156
H 6.91713924751780 2.74596309649073 -1.88272434602686
C 4.31712094177431 0.91576993287965 -2.62805142422620
C 4.26405613808816 -0.10708685051700 -1.67097157832012
C 3.34021743773807 0.97121307650763 -3.63917318142319
C 3.23770669414201 -1.04707601337540 -1.72846129785303
H 4.99151044559246 -0.15294726992257 -0.86286251805253
C 2.25386367337531 -0.99577496805952 -2.72171568399530
H 3.19305508671089 -1.83406983775438 -0.97583286402467
C 1.16844656262032 -2.00344204556216 -2.78096718673325
C 2.32595805952661 0.02601781906749 -3.68621548518500
C 0.23200674009488 -2.22277991887429 -1.82825605004830
C -0.84899446992377 -3.25640711536283 -1.87088334779561
H -0.90755023086455 -3.82607024262320 -0.92934430983418
H -1.83811692682917 -2.80262189285473 -2.04042962356281
H -0.64319661977438 -3.95760631328681 -2.69173298574098
H 1.56403899152128 0.08480592700869 -4.46723517106212
H 3.39669380969999 1.77989013243420 -4.37028630877842
O -2.66321085551241 1.45439154237364 0.84342778514738
S -1.47318046666726 2.05338014756562 1.50962042992372
C -2.00583761721994 2.53024459173524 3.14890963296538
O -0.40525863636999 0.99103269886885 1.74882014996932
O -0.91042504689351 3.25637089487259 0.86826093374475
C -1.05552578558115 2.97577017444707 4.07188431945097
C -3.35276243434887 2.45590963147645 3.49205276058420
C -1.46548537829451 3.32520782467311 5.35649222285005
H -0.00289288490271 3.03992128730851 3.78891315142977
C -2.81897626391783 3.25376736927274 5.73201582875380
H -0.72209724201915 3.66221160601647 6.08488032971329
C -3.25493602083429 3.65549246722060 7.11805321703847
C -3.75045744437112 2.81984430350666 4.77927676777327
H -3.39054278306291 4.74934122127383 7.18814647794557
H -4.21339975279277 3.18834418841352 7.39069063291234
H -2.50636727081611 3.37361514608888 7.87525204886286
H -4.80606908845448 2.74420480061266 5.05355764397633
H -4.07168178374115 2.08542382165222 2.76093920307422
O 1.04879353183947 -2.72990072101859 -3.92830271567663
C 2.23758607869940 -3.21874582284672 -4.56177332361186
H 2.93171497783752 -2.38622261583231 -4.76206861721357
H 1.88937697466300 -3.61022581271837 -5.52904601217798
C 2.91538745122012 -4.30502251153705 -3.74396119157909
H 3.78197536370242 -4.70742686193213 -4.29286094095854
H 2.21998570150650 -5.13373645624394 -3.53692353615750
H 3.27909710749292 -3.90794334382599 -2.78323808823073

113
INT-4K
C 2.34263326104389 0.69910271220217 -2.61640370942406
C 0.93014812514707 0.70821754022221 -2.05557708543679
C 0.62196903666153 2.04128784534882 -1.34196945010445
N 1.01716557839919 2.12772937883534 -0.05939031054333
C 0.65679159265134 3.28083808524591 0.76793515338409
C -0.75967157064221 3.11598278710148 1.32461075239728
C 1.73858124840259 3.51109943947846 1.81549932688750
C 3.06995407437899 3.66351537841751 1.39650588108834

C 4.09030799360103 3.90025553834084
2.31610688233873
C 3.79654130128163 3.99441894447565
3.68099216896660
C 2.47914537136062 3.83943724355737
4.10892095255583
C 1.45936863659488 3.59259676720800
3.18415163734196
O 0.06854114297821 2.93337537481917 -
1.97467540647231
O 0.81825590536787 -0.43369550152746 -
1.19616844239744
C -0.32106458321468 -0.63616518706956 -
0.50525664884835
C -1.52412222867822 0.05266602567923 -
0.72096459396151
C -2.62227153368302 -0.22009607154330
0.09644960297897
C -2.56580381074425 -1.14365514410688
1.13755046265549
C -1.37096071974332 -1.83736637218040
1.37185139943241
O -1.17405492396475 -2.74961894703219
2.32760917571029
C -1.98049674409665 -2.89545552428581
3.50484691325125
C -2.15661138146588 -1.60662644134249
4.30430489989177
C -1.22956498913845 -3.94605995116740
4.33942667378223
N 0.08777695206257 -3.70117687410016
4.47359106648044
C 0.97950876149172 -4.62280820449365
5.16907332353766
C 1.80628737682032 -5.43134912665101
4.15782020134623
C 1.85420940156975 -3.89733390026732
6.17864064854426
C 1.75983623034110 -4.20469064757637
7.54211134776972
C 2.57276171369825 -3.55976580014668
8.47930660419525
C 3.49307701234012 -2.59598646300759
8.05981438287580
C 3.59256683422810 -2.28039783005499
6.70137599886906
C 2.78010711600918 -2.92533555778115
5.76805331651547
O -1.82231423835323 -4.89400705398063
4.84869321048645
C -0.29110531670315 -1.60145597463643
0.50927882872775
I 1.43308670354208 -2.79726961681583
0.73447232668608
H 2.51825769867570 -0.20264867917837 -
3.22281998049215
H 2.48567353198746 1.58358267580462 -
3.25407882900479
H 3.07634983452182 0.72701686037838 -
1.79668838140183
H 0.19444093607041 0.63033476200100 -
2.87274713853388
H 1.42737505423713 1.32602339289749
0.43697723495835
H 0.65507767197590 4.14719193963343
0.08552086048133
H -1.46222485029310 2.99684756856074
0.48721135852846
H -1.06593603567971 4.00636775134806
1.89593603062549
H -0.82184404763308 2.23018171548267
1.97344843779710
H 3.30366666017495 3.58767343905718
0.33116513696491
H 5.12056678981270 4.01551001420440
1.96774249218463
H 4.59324121668957 4.17935768528336
4.40560043570291
H 2.24033233661977 3.89350638722895
5.17332915153989
H 0.44129974331045 3.44619946476995
3.54354743989867
H -1.59566650248411 0.81739226664557 -
1.49288764868319
H -3.55423002922760 0.32220154731695 -
0.08041880628182
H -3.43941552038626 -1.33601415802212
1.75812564250512
H -2.95151313191950 -3.33726809835880
3.23179745224020
H -1.19484050972411 -1.09399160134199
4.43578541221096
H -2.57857344250810 -1.86579974128320
5.28760317519211
H -2.84028821433665 -0.90101754744154
3.81466044799076
H 0.50325740511904 -2.89996458078648
3.98266656319402
H 0.31992163897992 -5.31457289081088
5.71120058725956
H 2.44425859883627 -4.76783379061896
3.55238400646112
H 1.13910844980200 -5.98528589100544
3.47821805236070
H 2.45977153046652 -6.15139128009699
4.67486081405760
H 1.03878494357305 -4.95736279586636
7.87408480268164
H 2.48530726843637 -3.81175850182127
9.53970170810804
H 4.13014471673128 -2.08943250061565
8.79010338819640
H 4.29982568995839 -1.52130004414884
6.36241732949921
H 2.84874291708423 -2.64603657334239
4.71531908085520
C -4.12674175484198 -6.38002198151724
3.15536679766718
O -4.19898596606858 -5.47130293252229
2.06162431162982
H -3.40690175236762 -6.03301927138958
3.91318504396543
H -3.85495209751353 -7.39207675657776
2.80540150724014

H -5.13586168185635 -6.41188315126300
3.58753124641605
C -3.11885122149836 -5.29356497964446
1.27540617940739
C -1.84988156927052 -5.83994896419375
1.52590142414900
C -3.31081211533366 -4.48296598138163
0.14063720336869
C -0.79715995813769 -5.56338934841611
0.65246840333462
H -1.67132288537015 -6.44557899920663
2.41325948175176
C -0.97821219534891 -4.75597282048167 -
0.47683439343581
H 0.18995460163141 -5.98342394779017
0.86044221456919
C 0.11951987451596 -4.46432904237071 -
1.43192952367236
C -2.25789446126591 -4.22510092705327 -
0.72299542593887
C 1.24762714610748 -3.76676597313609 -
1.16244433214450
C 2.33786367562452 -3.45703642141530 -
2.14088509990896
H 3.33744723667015 -3.68189591518956 -
1.73202122517389
H 2.19182524721019 -4.05329100896113 -
3.05233549286300
H 2.32725203400950 -2.38940075476451 -
2.41362487747546
H -2.41508026299538 -3.58368445367904 -
1.59338098702705
H -4.30245450081069 -4.06058708661976 -
0.03096622340081
O 0.16831096565230 0.43812399923718
3.23988969653675
S 1.51049498448831 -0.11961697103176
2.97619569665574
C 2.58883920219347 0.43849279068603
4.28501076914095
O 1.52484908488556 -1.64407372476956
3.07940814121133
O 2.15205492069925 0.28089595008260
1.69355614336120
C 2.05059906434620 0.74151683390568
5.53560841062000
C 3.95564613830688 0.55803526562663
4.04337325448505
C 2.89838397928726 1.16686226412474
6.55535114989526
H 0.97446608552272 0.66050530520103
5.69456460052851
C 4.28086566392509 1.29368863503288
6.34444006516777
H 2.47936160206098 1.40661408095479
7.53660709700814
C 5.18977113793027 1.72741847228367
7.46562964965618
C 4.79237859409676 0.98424735492528
5.07521247228365
H 5.39086982240506 0.88906501057883
8.15611617258295
H 6.16017123481885 2.08281010872930
7.08777108400611
H 4.73642985554414 2.53417268937712
8.06296014565650
H 5.86373251533036 1.09506310964319
4.88600208270336
H 4.34584210173338 0.35086231249243
3.04606203044925
O -0.06952016940927 -4.83699156544845 -
2.72958578137067
C -0.69689401782504 -6.09536211792412 -
3.00944724354459
H -0.89406040498697 -6.06566142598273 -
4.09123449632465
H -1.66635226298217 -6.15960316971629 -
2.48945068134983
C 0.19286692401966 -7.27203482385736 -
2.64760292438892
H 1.16537802895605 -7.20362807479326 -
3.16027788844992
H -0.28991326779604 -8.21680527504537 -
2.94507049447963
H 0.37427988329147 -7.31355102677592 -
1.56222590710628

121
(R)-4-TS
C 2.79917387109285 0.33688032391567 -
2.02828710678134
C 1.39576192764994 0.37112282208019 -
1.44586022323618
C 1.12442495781780 1.70393372288558 -
0.71681536338343
N 1.58181196855896 1.78515331997478
0.54479219243185
C 1.28000020558061 2.94225417070433
1.39027081513991
C -0.11926129817475 2.81249822030369
1.99698510472642
C 2.40520611922132 3.13405477588697
2.39907213573190
C 3.71970909653628 3.28512891117770
1.92915197630294
C 4.78007486767760 3.48334036771781
2.81198867613538
C 4.54373908852662 3.53934532458521
4.19007849744863
C 3.24350680198739 3.38538813924061
4.66808541468637
C 2.18343908600678 3.17711561277667
3.77987258421436
O 0.54266016543079 2.59932160932907 -
1.31901824465463
O 1.27872113941695 -0.77647241807949 -
0.59237946317112
C 0.16149279091447 -0.96208561971995
0.13421336348357
C -1.02727267756596 -0.23247252661298 -
0.02019750913236
C -2.10051505930420 -0.48851562243264
0.83457159072422
C -2.03442761982542 -1.44067948072770
1.84962116874013

C -0.85556874806288 -2.17903065170843
2.01870139558238
O -0.65903072467150 -3.12943534018656
2.93729766932399
C -1.38706398259768 -3.25179988858013
4.16939965175665
C -1.47172074648857 -1.96053473482984
4.97805942798850
C -0.61329760634809 -4.33146520813738
4.94521994371318
N 0.71165887253036 -4.10964593702042
5.02324666852407
C 1.62590778307530 -5.06554704247943
5.64219782872791
C 2.37860188338811 -5.86048394690064
4.56558299424787
C 2.56527244109688 -4.37408650813208
6.61711484057242
C 2.51569439381064 -4.68095150223964
7.98300289800565
C 3.38287388917194 -4.06122929434181
8.88808335376486
C 4.31303739099379 -3.12305718566432
8.43383041540884
C 4.36846669747130 -2.80816422569560
7.07276691160000
C 3.50211322951388 -3.42814963384727
6.17173372819881
O -1.19907456674042 -5.28297020753312
5.45536236282091
C 0.20106396737899 -1.95610589885804
1.12241994855350
I 1.89944149977475 -3.19191208149665
1.28096738234296
H 2.95301217501783 -0.57259570912344 -
2.62833155913182
H 2.94674282083323 1.21509497405671 -
2.67361900126069
H 3.54494262418484 0.35816414630420 -
1.21947536818439
H 0.64296336328376 0.31100080987211 -
2.24900182795450
H 2.01270843956604 0.97897865499249
1.01630045922464
H 1.27718778806981 3.81367338886963
0.71423984419617
H -0.85585296552205 2.72768772005320
1.18502804949946
H -0.37562692485112 3.70383713655624
2.59098057656633
H -0.18493350903645 1.92035633247813
2.63658430401833
H 3.90761372466143 3.24033528497603
0.85297321510618
H 5.79622371844645 3.59889714641369
2.42462387628875
H 5.37171640935071 3.69447474256475
4.88604643825218
H 3.04991785968428 3.41003363780589
5.74270085858172
H 1.17950736391175 3.03021183887226
4.17675430736718
H -1.10316851521706 0.55276443339059 -
0.77087599924000
H -3.01901879389381 0.09001313984156
0.70901151098902
H -2.88855245554088 -1.61999503298301
2.50072874582709
H -2.38573908189923 -3.66885541111287
3.96456890080092
H -0.49028449358854 -1.47316500985890
5.04222814807956
H -1.82968260641605 -2.20999517409137
5.98883783181424
H -2.16926573974158 -1.23529265471907
4.53917087311676
H 1.11736647761624 -3.30124961098788
4.53730096384788
H 0.98604387979324 -5.75891520530384
6.20547167784136
H 3.00043758659633 -5.19568778551884
3.94565748053816
H 1.66692339173486 -6.37907955773707
3.90394465047452
H 3.04094723384701 -6.61022873020425
5.02561596043367
H 1.78747317973061 -5.41379085830326
8.34248698763843
H 3.33035640251356 -4.31322348157559
9.95076885994327
H 4.99237813144577 -2.63628157228894
9.13899606696884
H 5.08340918957380 -2.06914303020697
6.70662772636238
H 3.53888759574714 -3.14942141463960
5.11732210623565
C -3.29332767172536 -7.11734261310293
3.98720084166774
O -3.55746987141492 -6.13286153784391
2.98840976279537
H -2.51094281497428 -6.76661376665133
4.67601387243257
H -3.00899169058723 -8.07643040840857
3.52092237084317
H -4.23705495865699 -7.24884916925607
4.53202698048644
C -2.61060111719857 -5.80719804224162
2.10295043741953
C -1.29464382399178 -6.31229166228587
2.11180770536793
C -2.99019522208383 -4.87985325472161
1.11052116799702
C -0.38844425154240 -5.89748055809854
1.14638212022855
H -0.97501886425683 -7.01099728437318
2.88344439954752
C -0.76935835852166 -4.99522486917353
0.13203086073775
H 0.62407282234643 -6.30419337245057
1.15193835836165
C 0.17597798009986 -4.52549108775087 -
0.86659482700734
C -2.08929370006498 -4.49153658941107
0.13848556339927

C 1.57702353205746 -4.27953258614843 -0.59953909636218
C 2.40958809384420 -3.74235118958572 -1.74540085908137
H 3.46458847050119 -3.65500916017755 -1.44846851465255
H 2.35041671620950 -4.46311845579916 -2.57593208745148
H 2.05985394080008 -2.76444073053035 -2.10315725272752
H -2.39162526238367 -3.74608941421596 -0.59783712811336
H -4.00547036299108 -4.48086562480237 1.13775555171669
O 0.82759706016398 0.07131622609336 3.82663909803334
S 2.14245112551889 -0.51925050046701 3.50526895491694
C 3.28877869299865 -0.01449724751507 4.77604614265993
O 2.11243403750247 -2.04691677966464 3.59185195621375
O 2.74434745817644 -0.12629134330051 2.20191917363369
C 2.81540169966254 0.26032039074537 6.05838694482224
C 4.64477213928384 0.09119243345320 4.47167330460177
C 3.71838565653458 0.64321422548222 7.04801780018705
H 1.74712546748408 0.19097384914551 6.26810636023605
C 5.09048745612679 0.75517598497387 6.77432149651411
H 3.35088304335558 0.86035716009593 8.05468769878069
C 6.06444018141406 1.13551739474155 7.85957712514764
C 5.53590286903993 0.47540977745398 5.47297286741805
H 6.41651464017755 0.23992204256669 8.40218818535992
H 6.95364792975222 1.63791346684558 7.44899990182791
H 5.60268076019278 1.80367463473104 8.60264409225956
H 6.59857528330037 0.57578315084742 5.23495778761580
H 4.98350741880953 -0.09363230827593 3.45147760372366
O -0.21441958786292 -4.19528096978847 -2.06556394439164
C -1.28130185663262 -4.88239132185872 -2.78097951690881
H -2.10508773633808 -4.16028506902674 -2.88792486106503
H -1.61181836881232 -5.73694722230507 -2.17808496160368
C -0.73208709844302 -5.32451026811346 -4.11907564215999
H -0.33692959279303 -4.46994593926355 -4.68978226795222
H -1.54165006902620 -5.78731828340100 -4.70646602947306
H 0.06175461556060 -6.06687926108519 -3.95916888722506
C 1.71037985076901 -7.36983826297728 -1.11582673598310
C 2.06330801203113 -8.82792197564573 -1.36453102783596
H 1.78308254792390 -9.42148951265130 -0.47844613868559
H 3.15088171368318 -8.93664813879971 -1.49441272049540
H 1.52856801663209 -9.21547613222573 -2.24230666489804
O 0.77060960673644 -6.84150836062518 -1.74484078284643
H 1.99551539040066 -5.40294319167313 -0.31209134181982
O 2.41094067646444 -6.76603878522861 -0.22833889299534

120
(R)-4-TS-CF3
C 2.80759455224409 0.35033969484217 -2.03809588981925
C 1.40450142022094 0.40877712883268 -1.45691529253212
C 1.15290336992498 1.74945721780096 -0.73545932568203
N 1.60617672383520 1.83014731662110 0.52742471051974
C 1.32835042630349 3.00189573890395 1.36143460745848
C -0.08138925709663 2.91847890022837 1.95167265044865
C 2.44691152377283 3.16815356859644 2.38214032497651
C 3.77088229815708 3.27631948192839 1.92721948748967
C 4.82590068337634 3.45107305752206 2.82132224954120
C 4.57483426331248 3.52594758433967 4.19591332198273
C 3.26501696493135 3.41406405910695 4.65926228421776
C 2.21001872317134 3.22955398097959 3.75979265121671
O 0.58957176328878 2.65094408871315 -1.34590941673441
O 1.26998329835679 -0.73422482396465 -0.59890932450004
C 0.14764526744409 -0.90661874306270 0.12203276758028
C -1.03000093684026 -0.15795271615957 -0.02954636843045
C -2.10886459155824 -0.40185848505982 0.82223886534570
C -2.06169594790915 -1.36227898308738 1.83133306178096
C -0.89525792240677 -2.12117552758418 1.99551999574620
O -0.71464022794949 -3.08358016773088 2.90264194045350

C -1.41744514042578 -3.17889065251410 4.15668209855969
C -1.41438138506130 -1.88957309447603 4.96882991739323
C -0.67057716174947 -4.31395517327728 4.88029579769378
N 0.65327568120035 -4.08544416655908 5.02339258584132
C 1.55182433696450 -5.07604126241903 5.60750965007689
C 2.28047919424926 -5.86686695806748 4.51077699285103
C 2.51678090468312 -4.42404083016565 6.58405550507087
C 2.52120202144011 -4.80036753159328 7.93325891284615
C 3.41510393711630 -4.21661532431045 8.83631189859089
C 4.31758165465502 -3.24468779554591 8.39720608263496
C 4.31933871812275 -2.86036738997571 7.05281447041165
C 3.42715319369321 -3.44568227878244 6.15353383194381
O -1.25945829009620 -5.31248274513096 5.27338671710813
C 0.16664026494698 -1.90809949885895 1.10321801131532
I 1.85489299502222 -3.15212249843232 1.30403995377005
H 2.94745914220360 -0.56356764951643 -2.63492318277648
H 2.96938437067257 1.22370852889161 -2.68653836824014
H 3.55279029622182 0.36307974191557 -1.22857704079222
H 0.65113426457625 0.35614499045863 -2.26005645693189
H 2.02302580426418 1.01905107262720 1.00294953081641
H 1.35917234706677 3.86817715233833 0.67935903980389
H -0.81023226907467 2.84919275222998 1.13130851406119
H -0.31818552144902 3.82184909401209 2.53541567203740
H -0.17982043768725 2.03383725621873 2.59714363108391
H 3.97063587546017 3.21687808185056 0.85390279910321
H 5.84957249153439 3.53351507257901 2.44555921249267
H 5.39875127076150 3.66285258141397 4.90043996291487
H 3.05904641137950 3.45413418743684 5.73116955103633
H 1.19729434406207 3.11699170846985 4.14522063250627
H -1.09215208928963 0.63318894372938 -0.77538867499317
H -3.01688097051549 0.19353585172810 0.69947147280579
H -2.91684628911997 -1.52771241849820 2.48551267724878
H -2.43966224660426 -3.53878653308338 3.97407337858174
H -0.40521300717461 -1.46544946932049 5.03855071305347
H -1.78897799196242 -2.11762181876369 5.97870096508597
H -2.06147404333626 -1.11778560756455 4.53124650318405
H 1.06820925876719 -3.25785733681319 4.58435183406313
H 0.90353780731801 -5.77029996658847 6.16051963735088
H 2.91315538194040 -5.20410139034291 3.89932071857644
H 1.55271493086349 -6.35909694800842 3.84711862806978
H 2.92946145954307 -6.63935843875170 4.95185410197514
H 1.81402072579703 -5.55880853974878 8.28136439346678
H 3.40443738122717 -4.52258241038180 9.88598167717001
H 5.01792287856052 -2.78648092990138 9.10098309713743
H 5.01319528775916 -2.09568362202831 6.69902230362998
H 3.42546313060285 -3.11641235338735 5.11286053061952
C -2.56139551970834 -5.87724692304343 2.18848040276319
C -1.25563084048635 -6.37479210059438 2.19332195583347
C -2.95521949812839 -4.94286256311151 1.22517432375861
C -0.34975053957804 -5.96287746059619 1.21960526133139
H -0.95313814338634 -7.07828054042655 2.96868587675464
C -0.75109851579223 -5.05900408945653 0.22134926206121
H 0.66451555860615 -6.36336646518219 1.21122242316074
C 0.17870037618141 -4.59360819229229 -0.82293774132149
C -2.05645806402070 -4.53769129025711 0.24548090784060
C 1.55673192136555 -4.24974662055434 -0.57141606087128
C 2.34400375061476 -3.68438902283855 -1.73625218600925
H 3.39358616054191 -3.52544429149868 -1.45094886329061
H 2.32289501133311 -4.41503123608226 -2.56023556816401
H 1.93205381091739 -2.73413749752428 -2.10216721426162
H -2.36122688583793 -3.78826922652280 -0.48547873519835
H -3.96778253150815 -4.53490333674528 1.23838498748669

O 0.82979039020869 0.16999086279088
3.75193413166073
S 2.13187716524461 -0.46151015676886
3.46395898420530
C 3.26857358263537 0.01363651468294
4.75306234251349
O 2.05014128019275 -1.98979102963012
3.56575183792106
O 2.77029591921308 -0.10463728457963
2.16725425660741
C 2.77966071467306 0.28715486134555
6.03147709514213
C 4.62976107978623 0.09225687806184
4.47084173186623
C 3.67390255623369 0.64084461488065
7.03747698511776
H 1.70711213464022 0.23606160863100
6.22424083068888
C 5.05355694180559 0.72813576353504
6.78539161929977
H 3.29579614391746 0.85261647367648
8.04153550135823
C 6.00513491571706 1.10903237080041
7.89015651449552
C 5.51361749354012 0.44905452705518
5.49066631297053
H 5.94216619065330 0.39595065044068
8.72946135243854
H 7.04779178676155 1.12915144896273
7.54039488867753
H 5.76488997431385 2.10535558722134
8.29782489722729
H 6.58236276655480 0.52360928389143
5.27215572354994
H 4.98150082537124 -0.09571959039787
3.45571358943509
O -0.25262106782444 -4.31229024261447 -
2.01640461438618
C -1.32444749768175 -5.04152533654161 -
2.68573235612141
H -2.15268483382060 -4.32760666656101 -
2.80937440010624
H -1.64161234685060 -5.87177841545679 -
2.04369717859510
C -0.79080294361242 -5.53353628856975 -
4.01243985323771
H -0.41528696156237 -4.69930183657285 -
4.62479984468608
H -1.60346945478601 -6.03082580426581 -
4.56607771456696
H 0.01513987589839 -6.25831337197663 -
3.83428900386186
C 1.78311041346441 -7.32022332752560 -
1.04503181593795
C 2.26537240520459 -8.70050563380041 -
1.45250414026052
H 2.64408364390609 -9.24353969991254 -
0.57378514819801
H 3.10695054496298 -8.59135552348676 -
2.15688766528942
H 1.46093006772824 -9.26603664546513 -
1.94214154209970
O 0.74056525776068 -6.85338234377984 -
1.55780330391347
H 2.03253724945572 -5.35003047594959 -
0.27081092069182
O 2.49840419005186 -6.69088358146054 -
0.19171484245201
C -3.58723012083201 -6.34729923668299
3.19401145559600
F -4.07286517860654 -5.31897031305478
3.91651919850001
F -4.64563152794180 -6.89157902057493
2.55346397376792
F -3.11060248719958 -7.26520108447918
4.03153667418188

121
(S)-4-TS
C 1.91968988919811 3.94812264331805 -
0.40223618588286
C 1.59032084683853 2.75743302312494
0.48092380776736
C 2.79676839565353 2.37828787972163
1.36674108343790
N 2.44484901869784 1.77434113436090
2.51772638792636
C 3.40873953598654 1.14449564280055
3.41944523305419
C 2.99242847307904 1.40741626358177
4.86636913655500
C 3.54138853352827 -0.32966069052830
3.05215084916499
C 4.41557497845756 -0.69471463243559
2.01498734764339
C 4.47432189277995 -2.01180933380015
1.56001465629693
C 3.66026468355057 -2.99335620136111
2.13774999727891
C 2.80573365677940 -2.64477834344682
3.18840240022285
C 2.74661614030135 -1.32139418279307
3.64179304239663
O 3.95281081108181 2.60641629677465
1.01885705236906
O 1.33656700836832 1.53622712199289 -
0.25540742657501
C 0.09878185974673 1.22009111388210 -
0.68983560253623
C -0.73186023150964 2.10496857201402 -
1.38853773864941
C -1.95297044685449 1.65835630016088 -
1.88409000810423
C -2.38786199225562 0.34582352663521 -
1.70818187902815
C -1.59018567640459 -0.54800131731272 -
0.98183028151220
O -1.92293553657878 -1.81749541913231 -
0.66707288291688
C -3.12105240304429 -2.43306863960537 -
1.16086929980774
C -3.04591042570704 -2.75037109173147 -
2.64689564335319
C -4.39856972321476 -1.65703402623173 -
0.76952522542803

N -4.35747986942958 -1.03212092671532
0.42466569714484
C -5.44420186939361 -0.13138427483941
0.82495598147678
C -5.56916926137621 -0.10908738540485
2.34791432003071
C -5.24202159972736 1.24352405593693
0.19686680740772
C -5.95063270325025 1.59043911530649 -
0.96245005923574
C -5.74203519562353 2.82668051256931 -
1.58226222819740
C -4.81662345642700 3.73037806359505 -
1.05106612049908
C -4.09416401914437 3.38291683036913
0.09465377616461
C -4.30523764648651 2.14972776237174
0.71239332346666
O -5.36940755789930 -1.66741441432198 -
1.51709426868594
C -0.35047243584278 -0.09569006808653 -
0.50143817413526
I 0.84963595134631 -1.41329094404322
0.62643825709053
H 2.69441177317274 3.69191903089913 -
1.13413541763920
H 1.03505045398952 4.32471523169550 -
0.93243966397775
H 2.30449826693317 4.76519548689258
0.22696815782872
H 0.70805527170632 2.95267881226871
1.11072716751893
H 1.48534405983566 1.41223458183419
2.58972760179280
H 4.37410273111223 1.63157679979981
3.21955690002390
H 1.96616841323754 1.05690605510847
5.05885398273515
H 3.01687226618666 2.48813980825743
5.07261913644450
H 3.66950793533354 0.89900619443592
5.56995493051493
H 5.03086577165556 0.07960520796359
1.54999349019969
H 5.13872043609728 -2.29378799515097
0.74157108565331
H 3.69871551361412 -4.01307217063313
1.75038440752411
H 2.17300177360472 -3.40434264241057
3.65616653026280
H 2.04844631928328 -1.06118864651570
4.43825762053676
H -0.41253194671793 3.13074751132070 -
1.55351577050659
H -2.60140329765971 2.35403473376245 -
2.41885682185635
H -3.35869000957339 0.04921760256529 -
2.09805151128442
H -3.15091447698271 -3.37915095506353 -
0.59561260343582
H -2.16933475833847 -3.38141934698562 -
2.85206926498592
H -2.97146344266092 -1.84096835000828 -
3.25910350830217
H -3.96114042555240 -3.27797749699569 -
2.94596961239934
H -3.47691469164986 -0.93924390216918
0.93839865055336
H -6.35993276870629 -0.55919605715010
0.39316321469025
H -4.63146590833338 0.21505979307122
2.82056648343630
H -5.80436502925040 -1.11788969131004
2.72253227575606
H -6.37462722128067 0.57566092910179
2.65392384734614
H -6.65877936627124 0.87521595510852 -
1.38788244829643
H -6.30718297883405 3.08503372208391 -
2.48226044381996
H -4.65622182122227 4.69935251770833 -
1.53250738718592
H -3.34694900165187 4.06311050013647
0.51091439947116
H -3.70054832830364 1.89262332911802
1.57922730705731
C 5.37661587630733 2.98056622156594 -
1.92819921284269
O 4.21963307147394 2.70769003218703 -
2.71752454409558
H 6.19721529556308 2.28888798609132 -
2.18544185096037
H 5.13653030110144 2.91531904453759 -
0.85533290624986
H 5.67880047642852 4.00414263393592 -
2.18438652033827
C 3.61050307253062 1.51978195052020 -
2.60478430842451
C 4.06536106883964 0.47406875479354 -
1.78148114612417
C 2.43265986507551 1.34977679178195 -
3.36145687380639
C 3.34373733824992 -0.70533346311085 -
1.70983673029513
H 4.96229109813377 0.58885806491808 -
1.17633189966169
C 2.16518996237681 -0.88977581649268 -
2.46099895255390
H 3.70585328094127 -1.51142891601437 -
1.07429236752603
C 1.37053161746333 -2.09060864538501 -
2.28525819150284
C 1.72537914994109 0.16482734822855 -
3.29326702066581
C 1.29819385721813 -2.79336278359974 -
1.02864882557699
C 0.46097549152011 -4.05365955523522 -
0.97033480384590
H 0.82503999580743 -4.73820500880762 -
1.75238906764215
H 0.59204296082368 -4.55321601091401
0.00071079223775
H -0.60852848035033 -3.86950914355484 -
1.13136201470460

H 0.78523316923199 0.07629151718689 -
3.83891767328832
H 2.08462220340395 2.18414282188367 -
3.97231506240060
O -2.24870894369877 -0.20063447746122
2.17905444434858
S -1.24904912280645 0.78228990382300
2.66783175001689
C -1.46547064080383 0.89633245752980
4.43881642534613
O 0.16371509253986 0.22558415279683
2.52909107387650
O -1.34294628419157 2.15428434516134
2.12215323680197
C -1.81878122006000 -0.24698607756350
5.16089268551001
C -1.20240441376515 2.10125148711725
5.08815666298083
C -1.90245697494665 -0.17423388949277
6.54933803038136
H -2.03019111396119 -1.17624719171290
4.62864844775027
C -1.63855679712833 1.02609389772933
7.23290102057683
H -2.18095073944154 -1.06608480588947
7.11842336742654
C -1.75244110530804 1.09026550251544
8.73459778055543
C -1.29022145443858 2.15763011903613
6.48099045516612
H -1.33442866409988 2.02629827992957
9.13356668606138
H -2.80775420017933 1.03311798393659
9.05330179219374
H -1.22619444908544 0.24847423484440
9.21331069265613
H -1.08650642830054 3.10174122982455
6.99395338785681
H -0.94098123857290 2.98199415234132
4.49920264490453
O 0.62450919794395 -2.57695287293079 -
3.23853760675837
C 0.96600446259828 -2.49507519797420 -
4.65043906024210
H 1.98029293597770 -2.08712971898604 -
4.74211971731315
H 0.24347837471267 -1.80436302915217 -
5.11165419121944
C 0.87355841745599 -3.88860107134399 -
5.23146299590920
H 1.63253329651797 -4.52938670665722 -
4.76230533535299
H 1.06491916252027 -3.84196607477082 -
6.31572395087388
H -0.12775983608596 -4.31957799981953 -
5.07685318472332
C 5.23640831170690 -4.89446593876374 -
2.35787969193594
C 3.95112832588357 -4.12164868487611 -
2.10806665250111
H 5.31652804656272 -5.73791665539664 -
1.65531576431501
H 6.09771373593822 -4.23220635350059 -
2.16882152019684
H 5.27870288322747 -5.25178488856503 -
3.39559115294427
O 3.21961122663595 -3.79239387862734 -
3.05585848142397
O 3.71269847503243 -3.82886838933549 -
0.87633427105687
H 2.49075487154149 -3.18273218374768 -
0.85597969538039

121
(R)-INT-6
C 2.20847125932652 1.35755621689053 -
2.62121639606715
C 0.85541674692141 1.45218706154756 -
1.93545685082323
C 0.74969154570143 2.72867029281561 -
1.07701988504395
N 1.24832975751978 2.63564381094146
0.16752195869354
C 1.11430148698173 3.73386745151477
1.12404480733593
C -0.25974646953336 3.69138637774652
1.79837756101140
C 2.30214036490561 3.71879275978371
2.07832128924179
C 2.15463248659048 3.81543124795203
3.46610121885651
C 3.27325040354779 3.84013585375579
4.30563657317346
C 4.55746907927669 3.75269608909150
3.77161062143493
C 4.71758653560329 3.64191573433888
2.38601102507601
C 3.60131480230340 3.62752675866314
1.55242692726321
O 0.25365120424360 3.73455282605373 -
1.57164363089839
O 0.69960903292164 0.24737817697396 -
1.17590197778685
C -0.41606553843908 0.04693399917604 -
0.45185306644488
C -1.54702165900504 0.87579824707861 -
0.47924688984627
C -2.63557988497468 0.56937718141658
0.33817990565993
C -2.62967524211658 -0.51571038019778
1.21146872269846
C -1.49651406887171 -1.33799236163417
1.27706437045135
O -1.34171273016504 -2.37020124659618
2.10937061274495
C -2.03919742618565 -2.51700512721632
3.35850662103070
C -2.02241417245242 -1.26967639903275
4.24091199658859
C -1.31300483670340 -3.66279192013650
4.08090662648992
N 0.02674637136932 -3.60725867548397
3.98135774030732
C 0.90620999501254 -4.59510993391161
4.58721668404698

C 2.03020979340864 -4.94065784208831
3.60048167150992
C 1.46037367832830 -4.12839195984029
5.92546603470668
C 2.12473466922891 -2.89665306107609
6.03314094540250
C 2.66492602436455 -2.49030458967519
7.25436482563726
C 2.54489112563623 -3.30575998720312
8.38484162299870
C 1.87735579887377 -4.52875583046345
8.28740034001772
C 1.33863466384663 -4.93502735197199
7.06266476630626
O -1.93947619420899 -4.51423246091316
4.70614913081602
C -0.44175973313380 -1.08174523851706
0.38364180834150
I 1.18091883965713 -2.41786468957146
0.34601677313430
H 2.38617381453496 2.27277231267699 -
3.20432807916780
H 3.00627150160967 1.24994584302323 -
1.87101393018306
H 2.24198658976377 0.49215634484236 -
3.30105241855516
H 0.04934332589649 1.52558665072627 -
2.68413143094995
H 1.60183360401616 1.74381298824028
0.54050290544046
H 1.16969349757207 4.65925348826305
0.52548769750777
H -0.40749743658565 4.56468252702928
2.45277106380273
H -0.37201876211431 2.77025679576113
2.38867208889841
H -1.03959330452160 3.71967168548063
1.02370461964818
H 1.15966592640039 3.86265703859109
3.90858527462666
H 3.13424305401128 3.91401190126310
5.38646962404685
H 5.43010984507023 3.76295449656192
4.42921043327662
H 5.71945823251451 3.56668743997119
1.95422964218454
H 3.73114775894095 3.53611439698052
0.47131778441066
H -1.57224073203130 1.76157760417182 -
1.11221375508077
H -3.51644052326501 1.21493570970043
0.30148643487984
H -3.48954857146235 -0.72322149252583
1.84648139746020
H -3.06696937807237 -2.85719479037761
3.15958343609118
H -2.70415471246934 -0.48798205386720
3.88212246456262
H -1.01489242338992 -0.83510724890519
4.28512342888816
H -2.34354558900900 -1.56537223032583
5.25137945298039
H 0.46292914838002 -2.80818397404782
3.50842476535772
H 0.28694657855246 -5.48522182171324
4.77100990365792
H 2.63207176191963 -4.04534755964544
3.37757497286340
H 1.60822867392004 -5.31754825960897
2.65487908295881
H 2.69739149305492 -5.70587611771740
4.02569098310538
H 2.21375415587449 -2.24929193611153
5.15793932224118
H 3.18029217552379 -1.52993375014772
7.31810504325239
H 2.96750290705672 -2.98529361157537
9.34098921336718
H 1.77023263313065 -5.16811384259357
9.16792999687729
H 0.80853931678836 -5.88918299916861
6.99017453636239
C -4.74718388862551 -5.42937861600519
3.16946047426109
O -4.63318627280524 -4.70844000209486
1.94799090349823
H -3.98805596857182 -5.11091575019678
3.90252667301172
H -4.65220367704561 -6.51605303114040
2.99371490829038
H -5.75363445292635 -5.21699728215733
3.55429897828607
C -3.49219336078899 -4.80958397071678
1.23177903590693
C -2.33560315813009 -5.46507083088282
1.67349722655512
C -3.49110382229952 -4.19825050425518 -
0.03431535732000
C -1.18340672015804 -5.44368185891793
0.88760510021124
H -2.30543194158287 -5.94340252999419
2.65004375357511
C -1.16126886224252 -4.81705617285562 -
0.36163560173004
H -0.28467865580953 -5.92978160738956
1.26173958481143
C 0.07501866493652 -4.81093109700763 -
1.25232584520314
C -2.34776139522688 -4.21928536053110 -
0.82171588795091
C 0.66220591701507 -3.41152068388455 -
1.57688719989108
C -0.14313150406480 -2.52181338699375 -
2.49929279099636
H -0.40490483612572 -3.11658369863385 -
3.38747930474893
H -1.06647780637232 -2.14511331533263 -
2.04229953652542
H 0.45805490824224 -1.65969605881311 -
2.81310117563853
H -2.38963809857659 -3.78019271801821 -
1.81804601425004
H -4.40872843411464 -3.72048727193617 -
0.38264684201430

O 0.41779851886716 0.66223232467445 3.19920446959704
S 1.69146536144893 0.01007632767675 2.83016955625362
C 2.87875866226281 0.38608701216120 4.10933909567361
O 1.58716255081145 -1.51139183470237 2.83378972351638
O 2.29748046528297 0.44739414828520 1.54047126066660
C 4.24145671773491 0.27731717236871 3.83029521214900
C 2.43216004915435 0.74831909031134 5.37797106737413
C 5.16309006154774 0.52884679831194 4.84332561328192
H 4.56596516083127 0.01720950210262 2.82166791588623
C 4.74450286803142 0.89809291809969 6.13228978537198
H 6.23259519768712 0.45021966111451 4.62801111802673
C 5.75481550070232 1.20750124648239 7.20695910222723
C 3.36835304830335 1.00382830545762 6.38065338857526
H 5.30138440626762 1.18462425519668 8.20929308602213
H 6.59221648232123 0.49192941303436 7.19207947675496
H 6.18705098877907 2.21358535294209 7.06292485017385
H 3.02202731505827 1.29464934023982 7.37644134715464
H 1.36180054445810 0.84159529628712 5.56630471615590
O -0.15658438682189 -5.38020773599655 -2.49786626438015
C -0.69849561847273 -6.70143298950177 -2.53581914291926
H -1.73319589332775 -6.69339475724208 -2.15142942213850
H -0.10978939662972 -7.36598356399438 -1.87863972288470
C -0.64025805033956 -7.17469357124644 -3.97333973287144
H -1.23107134077320 -6.51243815927147 -4.62541509092195
H -1.04681284584569 -8.19486688593232 -4.05631437008115
H 0.39910421103584 -7.17769648225024 -4.33592107291518
O 1.07579557431961 -5.56810155687641 -0.51386845425159
C 2.27804272472023 -5.92315576182255 -1.04213048672118
O 2.70958131676887 -5.52862318085880 -2.09353002893156
C 2.98672932970898 -6.86802904518915 -0.10457421118725
H 3.96654304307102 -7.13177523977475 -0.52044908170766
H 2.38078604523413 -7.77567695939350 0.04197881600283
H 3.10854566561608 -6.39789278011037 0.88368936158265
H 1.65745082906792 -3.58089074898724 -2.00416158555299

120
(R)-INT-6-CF3
C 2.31085185197515 1.31172556659709 -2.43775162706674
C 0.91282188811481 1.35540429559477 -1.84326124235783
C 0.69892347761837 2.63204744114145 -1.00398966019576
N 1.14870437072112 2.57874973332723 0.26127876927462
C 0.90504708805495 3.67404156859647 1.20225554482762
C -0.49655281382999 3.56037883113903 1.80677310579743
C 2.04324491692273 3.72890320021338 2.21311429294174
C 1.83561809643801 3.64050158644807 3.59395209594726
C 2.91006937511207 3.72701653361891 4.48510735556815
C 4.20972261054431 3.89164821115868 4.00869522225123
C 4.43159618975664 3.96644024776909 2.62913127497596
C 3.35742099613705 3.88660124595017 1.74429037562820
O 0.16648231916203 3.60309836142389 -1.52961928896863
O 0.75660166749494 0.14857334259438 -1.08538805882635
C -0.38311479068098 -0.08138360743535 -0.40977358065566
C -1.54236492070085 0.70364063123695 -0.50104640545379
C -2.64859019896531 0.38330801822496 0.28773883888509
C -2.63322832666277 -0.66706304155919 1.20279304606699
C -1.47269189512684 -1.44263165754971 1.32991112944435
O -1.29449748361682 -2.42353493860865 2.21644581114889
C -2.02848763243084 -2.53898156900803 3.45073900690607
C -2.02423208904578 -1.27068275294380 4.29744374301065
C -1.32783951672262 -3.70249475170908 4.16934344827812
N -0.00119877034271 -3.52406683955362 4.34658545713041
C 0.82866492207584 -4.56633920517828 4.94320963683045
C 1.37935582493409 -5.50814237814985 3.86087877098986
C 1.92430809675387 -3.96850149138318 5.80894969052633

C 2.95428227931171 -3.19605924918130 5.24942387030839
C 3.96417178912990 -2.66830101392596 6.05424251171769
C 3.96238284045606 -2.90348856010681 7.43203906326186
C 2.94034777695054 -3.66718983768387 8.00074364199620
C 1.92940845609479 -4.19488674173490 7.19162630645344
O -1.95627569980566 -4.68829300084129 4.54147785702864
C -0.40587761087268 -1.19115454193818 0.45047131616087
I 1.24435165222846 -2.49489623706250 0.50834835199766
H 2.48947606671765 2.22918672838637 -3.01717124225015
H 3.06060354679037 1.24307519462475 -1.63533082944183
H 2.42521435214863 0.44416103391906 -3.10603616528105
H 0.15426052914190 1.39238521869730 -2.64257625535172
H 1.54276367304001 1.71326109712662 0.65434163730786
H 0.94092960644896 4.59741473295432 0.60019024012210
H -0.70755108778715 4.41061427282360 2.47434722071274
H -0.59972945971228 2.62274779021813 2.37151197013786
H -1.23960640775640 3.57553973950769 0.99629976545367
H 0.83135723405982 3.48429702274881 3.98612343958250
H 2.72744110623940 3.64792624761676 5.55916262083265
H 5.04839176077749 3.95165632194029 4.70654564805397
H 5.44701431855000 4.09132701470255 2.24268758592183
H 3.53346247447508 3.94429124305778 0.66664799000729
H -1.57431069031148 1.57124801671786 -1.15856599219064
H -3.54999401342537 0.99439112338468 0.19843897729205
H -3.50559670372777 -0.89062159088697 1.81460180074326
H -3.05373948486937 -2.86934640507762 3.23674010843610
H -2.68299447866015 -0.49468592685190 3.88576416839815
H -1.01712571319749 -0.84071244087924 4.36736402872921
H -2.39033428998671 -1.52716180018837 5.30366254391482
H 0.46494159717319 -2.71882416639374 3.91111516006252
H 0.15464968023379 -5.15179591115174 5.58460681399988
H 2.00796445646047 -4.95560100279645 3.14408106943283
H 0.54621091339065 -5.97293376817096 3.31128799744907
H 1.99005711952559 -6.30721371329754 4.31007767910995
H 2.95063467141228 -2.96885954308253 4.18230454380663
H 4.74720334841502 -2.05629564433084 5.60269914199271
H 4.75419134837331 -2.48759696703833 8.06101600742143
H 2.92774047642796 -3.85455578794976 9.07787204551089
H 1.13000825488724 -4.79185729865563 7.64013054334739
C -3.79466448835356 -4.41877275971517 0.60575456665367
C -2.76241831751403 -4.97413143534731 1.36450246402983
C -3.56920281924695 -4.05144132074710 -0.72481820999728
C -1.49175090777428 -5.11246529057354 0.80640038080454
H -2.92690201042570 -5.26524486836332 2.40409586943555
C -1.24800906270238 -4.73045809286559 -0.51668597494671
H -0.68258294384829 -5.51574970418627 1.41074607899810
C 0.12961062432560 -4.86707800057520 -1.16586292200318
C -2.30663239517200 -4.22374603782961 -1.28544859643300
C 0.85342264109120 -3.51944400511729 -1.42862324799004
C 0.21886265736394 -2.60995910874885 -2.45904697957126
H 0.05640179360088 -3.20015096749580 -3.37377784495831
H -0.74321899128433 -2.19478651406466 -2.13162317692064
H 0.88980984596267 -1.77444456109195 -2.69113956366524
H -2.14949009149942 -3.97959440875716 -2.33556949076657
H -4.38418310258827 -3.64314174073937 -1.32393570132103
O 0.39509964522569 0.65980051144043 3.30954479074992
S 1.66180665408953 -0.00300374071126 2.93815862288137
C 2.84757395581942 0.33290970721027 4.22820010505096
O 1.51970732207544 -1.52371814675972 2.92464687384994
O 2.28161658293435 0.43783865077829 1.65699480810783
C 4.19552703128116 0.46121348353338 3.90523943124426
C 2.40919265972804 0.44487809233651 5.54867779567001

C 5.11784105048398 0.70115151214119
4.92546171077841
H 4.50509223728843 0.40326182938672
2.86111599492347
C 4.70899076659630 0.81262988917597
6.26137669839618
H 6.17616742318901 0.81603619249789
4.67538155282709
C 5.70297041120256 1.05262850810012
7.36835244436298
C 3.34086424348878 0.68240780662614
6.55432961194418
H 5.72159360326134 0.20206257433885
8.07126578083776
H 6.72227497513507 1.18941825535682
6.97802372354015
H 5.44049218875135 1.94758556667081
7.95669882089692
H 3.00259090954773 0.76371563078782
7.59103812275396
H 1.34640020506963 0.35526541146849
5.77823890722069
O 0.05657124114225 -5.46450569523366 -
2.41822895304336
C -0.45332701554555 -6.79685843414008 -
2.49053283932498
H -1.37043237320438 -6.88445052251030 -
1.88259434718503
H 0.29046272600589 -7.49993189522923 -
2.07416876736010
C -0.72530460195468 -7.10840754930310 -
3.94720460660851
H -1.49450390120376 -6.43397389404894 -
4.35482745715617
H -1.07953951744507 -8.14545647851606 -
4.05549174417728
H 0.19179433025324 -6.98884519743748 -
4.54442868423593
O 0.90144661191935 -5.67510804714894 -
0.24674457390260
C 2.15837275853563 -6.11981425110289 -
0.52899644215448
O 2.81641920199408 -5.75169605786835 -
1.46613561356844
C 2.59349946074173 -7.11569378404824
0.51347663715463
H 3.59280327424793 -7.49321084175021
0.26629949618035
H 1.87213555476022 -7.94519054339380
0.56832516207162
H 2.60618040121931 -6.63521094235241
1.50375829922647
H 1.87597168153986 -3.77566854814772 -
1.73111726326828
C -5.13677515116165 -4.17842640957956
1.24438438803904
F -5.11825250922964 -3.05757653681464
2.01204026742198
F -6.10960894889090 -4.00330691652400
0.33551310612010
F -5.50209683126535 -5.18653378916607
2.04728243229204

121
(S)-INT-6
C -1.09840253867716 4.56189644711165 -
3.35909142490887
C -0.52184772017553 3.52864004514679 -
2.40606447310771
C 0.78063721407186 4.04158937753808 -
1.77692434922988
N 0.68499687421868 4.31181714086854 -
0.45986046339488
C 1.83092623565946 4.78407644256612
0.30805750972246
C 1.34252490227309 5.65590514901285
1.46765397978229
C 2.69179606486143 3.61877809979033
0.77888161015111
C 2.16243911123525 2.64438879643058
1.63992926580983
C 2.94526101021154 1.56571445080070
2.05844819340239
C 4.27107614555228 1.44738318843783
1.62595475710323
C 4.80281019720629 2.41018370915658
0.76404608992616
C 4.01347913228024 3.48468938929797
0.33939276229289
O 1.78774778858111 4.21459565776047 -
2.45435754310271
O -0.19000327587298 2.31862662943036 -
3.13942048485793
C -1.05495653881978 1.28479898998156 -
2.96449793872131
C -2.13315533184680 1.08471118770735 -
3.83312966759127
C -2.98268388923091 -0.00414906528170 -
3.62946473867039
C -2.80141685527300 -0.88441159789978 -
2.56344942113366
C -1.73948763713028 -0.68696583074264 -
1.66909240236844
O -1.46415154015836 -1.44898060871967 -
0.60994845346747
C -2.38142956328913 -2.39051213852813 -
0.04274135524556
C -3.70408771736227 -1.76111975360797
0.39891658246354
C -1.65848666582598 -2.96305377530666
1.18290162964503
N -0.97144107638830 -2.04914823193794
1.89671834287849
C -0.32815827790923 -2.37822352564527
3.15372839482699
C 0.91856870110177 -1.50062946907360
3.31734984057416
C -1.27437750825528 -2.26598182378593
4.34888636332545
C -0.86169371406108 -2.74540453052022
5.60147576719090
C -1.70101582627133 -2.66426076628972
6.71326240151260
C -2.97906245524694 -2.10937977538239
6.58679109585713

C -3.39913987725928 -1.63274649702607
5.34396091935142
C -2.55039974482277 -1.70441713756927
4.23614411069088
O -1.77480990983651 -4.14461816602911
1.48943268468445
C -0.85520230025167 0.37268952617665 -
1.92239040398923
I 0.90809279383433 0.54112927606903 -
0.77353776145794
H -1.33279505577225 5.48970207633567 -
2.81365887735183
H -0.35663828452241 4.78659117571454 -
4.13954614685602
H -2.02375025832364 4.19097327379366 -
3.82506313938392
H -1.24715498928540 3.26929762463956 -
1.61763882983745
H -0.12874809666111 3.96943565915581
0.06973635456459
H 2.43949153353537 5.38812662610825 -
0.38088637616990
H 2.19288972662726 6.01123510292879
2.06919192252470
H 0.66258914586928 5.09110805216709
2.12461429431673
H 0.79295038263526 6.52878181990506
1.08202789444673
H 1.11891133301124 2.71045406679961
1.95288109842942
H 2.51690751289908 0.81199416960975
2.72199091594670
H 4.88427117584572 0.60634241089427
1.95968044518218
H 5.83647056507085 2.32548996056658
0.41741420355391
H 4.42390259336938 4.22513295661952 -
0.35207010384659
H -2.27848438912277 1.78222249919860 -
4.65884896767529
H -3.81492577259579 -0.17108428877220 -
4.31768938450049
H -3.47243035230964 -1.73162331899276 -
2.42993789975390
H -2.54330874549653 -3.21926620008616 -
0.74806580350087
H -3.51855564682305 -0.81724231487919
0.92882139763737
H -4.22168842341302 -2.46882684963301
1.06377313910741
H -4.36721723188579 -1.53784930326791 -
0.44718906753032
H -0.94158760650126 -1.07304404763088
1.58108529808800
H -0.02591925911492 -3.43637237478305
3.08650241792821
H 0.63332568284864 -0.43831657061531
3.32269269708328
H 1.61370026580606 -1.66634388468933
2.47908246458254
H 1.44197881031180 -1.72595395942037
4.25783382251061
H 0.12696484547954 -3.20254392542577
5.70627551337471
H -1.36282226997065 -3.04902893717152
7.67928085191355
H -3.64631497785482 -2.06069738560989
7.45151017582799
H -4.39448036442363 -1.19853964526446
5.23001659684540
H -2.88833508912785 -1.32310264485506
3.27327216649223
C -1.63468787579161 -6.05074241070253 -
1.21798243873605
O -1.52215121855206 -5.03910729567319 -
2.21228391794379
H -0.73939856812150 -6.69778518536907 -
1.21286094903667
H -2.50897325562348 -6.65417462963372 -
1.49768784189384
H -1.78714409773357 -5.61869875778763 -
0.21540223249561
C -0.46104144825154 -4.19921057847143 -
2.17994766027607
C 0.45657515502309 -4.12098893267472 -
1.12221644902507
C -0.31252468343813 -3.34536705613891 -
3.28602631248638
C 1.48988116066888 -3.18523613799859 -
1.16954653685670
H 0.35128771298388 -4.76114710866429 -
0.24755936760422
C 1.63384370101966 -2.32169618668247 -
2.25635960142257
H 2.20090362406172 -3.11960954834235 -
0.34765368310120
C 2.75337123811090 -1.29002325969906 -
2.31639265613040
C 0.72415200657860 -2.42191916032525 -
3.32019122564033
C 2.20498631762099 0.14227131019407 -
2.53650128016261
C 3.21638530507397 1.25850447222547 -
2.69821315863745
H 3.81693637922802 1.42038693713644 -
1.79309872249428
H 3.89788471538969 0.98859018536625 -
3.52052759527021
H 2.71345524442346 2.20403264818570 -
2.94434691877116
H 0.82353548373697 -1.77316602365654 -
4.19380506303023
H -1.02915486901554 -3.42436118704908 -
4.10520140181187
O -2.92111611710158 1.48317065429896
0.46744130526445
S -1.79962292683665 1.82087853466560
1.36643531415945
C -2.39845083622316 1.74636061094187
3.05002752618556
O -0.68317163325103 0.78615756819253
1.33530247812218
O -1.24815252214870 3.19606877821411
1.18770141955490

C -3.77028291584756 1.74284364570902
3.29065433563404
C -1.48655713191925 1.72723902998277
4.11015409307163
C -4.23119830741168 1.71774504728976
4.60976925855125
H -4.46108124156634 1.75780928983535
2.44576532291011
C -3.33961087695999 1.69088482013064
5.69016771950087
H -5.30796466290056 1.71305582111082
4.80135256628838
C -3.82886738872581 1.62385506213700
7.11256976396418
C -1.96093323467408 1.69762907737045
5.41699728620720
H -3.38914362312812 2.42728419722922
7.72623809392395
H -4.92398444684068 1.71014132020640
7.17176611289463
H -3.53723977593599 0.66567249635368
7.57483939503254
H -1.24922650898306 1.66557663740260
6.24654837420743
H -0.41373741704765 1.72800718340031
3.91064178159929
O 3.48737793430804 -1.36623510451405 -
1.13417764297175
C 4.86810558549718 -1.00831593870327 -
1.06072027458885
H 4.95456890425876 0.00771690530715 -
0.64513312061921
H 5.31732457946694 -0.99240748841392 -
2.06470122554846
C 5.56815120874750 -2.01670199088273 -
0.17042259974230
H 6.63373212164528 -1.75660798820037 -
0.06164681358884
H 5.48653476607316 -3.02492910008284 -
0.60177670266515
H 5.11264814708761 -2.02601569084639
0.83277891015979
O 3.54750722194143 -1.50245133871735 -
3.50658019528017
C 4.14628538811443 -2.70905042892482 -
3.72913454985619
O 4.24692502485925 -3.57224852374859 -
2.90163807356258
C 4.64964104996235 -2.77914068114923 -
5.14758411266906
H 5.27866668827501 -3.66888787330224 -
5.27191458170210
H 5.20967846203522 -1.86840158027025 -
5.40667411621639
H 3.78679430151312 -2.83891184041768 -
5.83058204689406
H 1.47740179209631 0.14797966187928 -
3.35516903839417

121
(R)-6-TS
C 3.35291108020680 -0.05263003695227 -
2.58555412477587
C 2.10603293967693 0.71434977061283 -
2.17265130078495
C 2.48314210148317 2.01093203943152 -
1.42884011584775
N 2.66375540249249 1.91389101965821 -
0.10122257531940
C 2.90474979312216 3.11291066981436
0.70247050223522
C 1.57891166501467 3.75664293622622
1.11766788648708
C 3.85221782063076 2.78802475257642
1.84954575270949
C 3.57982047800792 3.14097027253294
3.17564416685708
C 4.51022271509316 2.88979410863069
4.18885368570876
C 5.72087712023872 2.26549861053696
3.89495500590373
C 5.99551367877247 1.88694286160477
2.57617135065327
C 5.07012580840399 2.14834263907607
1.56735022625299
O 2.62401152296589 3.03459929247084 -
2.09223126404653
O 1.30752459300659 -0.19115360920041 -
1.41279212330405
C 0.14834001371367 0.22444964481063 -
0.85363721286218
C -0.44100798895370 1.47263164133881 -
1.10421440819122
C -1.62337926626316 1.80939018199968 -
0.44645583822514
C -2.23630827129774 0.93376396050792
0.44644175468594
C -1.66026026880276 -0.32011621791199
0.69648069142165
O -2.20286591871507 -1.25236478116862
1.50329096140627
C -3.01840532541938 -0.96156720273618
2.65004948368372
C -2.57524390069557 0.23936070215896
3.48461253729462
C -2.96480986831520 -2.24866685791239
3.49413251026109
N -1.71283857260020 -2.61563612369262
3.81696481954563
C -1.40550121652582 -3.67164713299812
4.77009038008086
C -0.18632520126508 -4.46457885802288
4.28227915948958
C -1.17486412547760 -3.10260477075383
6.16510248700560
C -1.87368862682658 -3.60986770486207
7.26636898421957
C -1.64267448953024 -3.10882956546367
8.55183203170006
C -0.70632443680629 -2.09091972019513
8.74664634071967
C -0.00732886042577 -1.57482820459001
7.64955779471861
C -0.24006057128681 -2.07361276976517
6.36709128371011

O -3.99501480133396 -2.83082752557940
3.82272362573478
C -0.48553838851546 -0.67688174448524
0.01645436918182
I 0.33991069477171 -2.59401878624569
0.28807148061332
H 3.08690247317523 -0.92950167585352 -
3.19626429604754
H 4.01009108246525 0.60595339052796 -
3.17172408476236
H 3.89378513759808 -0.39797501331752 -
1.69169666391326
H 1.54930127486145 1.04616217064834 -
3.06623716611034
H 2.45562939866260 1.04969171980298
0.42498058448423
H 3.42605201862275 3.81382862356729
0.02952210168134
H 1.75208484049845 4.70393267260514
1.65241828720861
H 1.00080260928922 3.07543369742328
1.75898610117376
H 0.99357152519060 3.98567811644373
0.21511332162985
H 2.62696528490998 3.60318356876802
3.43232693276269
H 4.27321679536189 3.16591138473248
5.21860471182902
H 6.44250535402652 2.06072934477097
4.68976417609544
H 6.93753964965628 1.38659145117024
2.33423447200462
H 5.28716062405162 1.85145604177720
0.53791937728225
H 0.03329659787487 2.18943967293521 -
1.77287981977083
H -2.06948486739153 2.79059337815841 -
0.62658192398362
H -3.15271206866568 1.22353499712010
0.95734833437244
H -4.07131087542798 -0.85585832500701
2.33248439648720
H -3.02084639473018 0.14648975748833
4.48709604002317
H -2.90539283310019 1.19588615676104
3.05909474205935
H -1.48153073308968 0.28088147793156
3.57778384851295
H -0.92212457460881 -2.03988832195098
3.49154722598976
H -2.28915640740982 -4.32542621451455
4.81105237229445
H -0.39524987267413 -4.94233703406474
3.31171087299451
H 0.08248325675455 -5.24544800893710
5.00998585406991
H 0.67608855726005 -3.79324586508209
4.15055519483666
H -2.61319035918506 -4.40189151996227
7.11494482450336
H -2.19991115756811 -3.51279589987725
9.40176549078588
H -0.52354222763384 -1.69739002650071
9.75044510774354
H 0.72894389738121 -0.77928852412679
7.78849580408600
H 0.29947362491976 -1.65809097711322
5.51270244630628
C -5.57063711071292 -5.69787148920076 -
6.88183415087261
O -4.88431011649280 -6.27949245900865 -
5.78088555740362
H -4.89822461351166 -5.05798146636617 -
7.47725373917250
H -6.43858066677041 -5.10865789360036 -
6.54245865886506
H -5.91797484653405 -6.53427095813477 -
7.50047065241294
C -4.35311520719608 -5.51043941142710 -
4.83369757206904
C -4.39636355903147 -4.09731610114992 -
4.84145125348198
C -3.71400275058623 -6.19203063845157 -
3.76964905521370
C -3.81985247361721 -3.39163661334945 -
3.80282055270349
H -4.88741226098406 -3.55651600692656 -
5.64935383779209
C -3.17997746385204 -4.05951426474900 -
2.73149750717725
H -3.85948236458811 -2.30235436989389 -
3.81543839581491
C -2.77274684641029 -3.31757568077973 -
1.38506763855158
C -3.14154274885819 -5.48127892261216 -
2.74072697137978
C -1.58447664936776 -3.26336841099549 -
2.21477137480930
C -1.15693437034912 -2.10605787890402 -
3.05495647299378
H -1.03563278508229 -2.39548319187276 -
4.10990989859913
H -1.84358011320812 -1.25592283276071 -
2.96713389232649
H -0.17214166520293 -1.77924640471848 -
2.68693952544827
H -2.66943185620703 -5.99718240787515 -
1.90273256169347
H -3.70256364465718 -7.28296706940951 -
3.78919342389183
O 0.55389817851631 1.20992084828976
3.00389443317943
S 1.39602182335640 -0.01133575350568
2.94824815831399
C 2.55309654463012 0.08227690584584
4.31435741030548
O 0.62175476181031 -1.26601465800673
3.23620657915219
O 2.22540599924073 -0.14495725846531
1.71414074120755
C 2.17476700474371 0.72816238295418
5.49046492267072
C 3.77807086796164 -0.57713507862349
4.22504372959212

C 3.03584809491112 0.70932509985158 6.58840008820195
H 1.21555593379204 1.24741367106101 5.53175415241989
C 4.27371235388536 0.05132210718651 6.52706606964079
H 2.74478642368830 1.22122738238963 7.51073058008728
C 5.22126017521064 0.06367290849650 7.69986640508506
C 4.62744795585130 -0.58986353186188 5.32972584527897
H 6.02222640588246 0.81045960549132 7.55374810030667
H 4.70359157710463 0.31628063688986 8.63776246228023
H 5.71263734444471 -0.91352127560002 7.83262131183495
H 5.59296424020491 -1.09971631388353 5.25927792728162
H 4.05708504008854 -1.05747624807641 3.28612498766319
O -2.65848900861590 -4.12792148313558 -0.31099012867559
C -3.74900255869799 -4.29811404878571 0.62145989872541
H -4.62816960970628 -4.69267592971816 0.08706906717377
H -4.00457080178493 -3.31869573750778 1.05057409372823
C -3.26130740870140 -5.22397506079274 1.71025575372082
H -4.01495843929358 -5.27504392671198 2.50856312828310
H -2.33798078698762 -4.82172495854840 2.14867755401969
H -3.06564368837342 -6.23667581752686 1.32281220470872
O -3.40336298316953 -2.09052709214403 -1.19154240430376
C -4.77878880764201 -1.99227248582414 -1.14423159573925
O -5.50821936220805 -2.90164911204712 -1.42661243926316
C -5.18047476750666 -0.61989958778651 -0.69924164796284
H -5.01682607998640 -0.55116161275059 0.38770184806490
H -6.24329419252355 -0.46079431141555 -0.91694349843288
H -4.54807050503947 0.14845536800991 -1.16427073923091
H -0.90856257148621 -4.10872086933713 -2.09576428219967

120
(R)-6-TS-CF3
C 3.41680755227120 -0.07408162597641 -2.54867518311150
C 2.15884417727160 0.68883561592531 -2.16366822196567
C 2.51298361407945 1.99559472193673 -1.42651207092609
N 2.68767384326939 1.90949955620011 -0.09749677329247
C 2.90969293890492 3.11733610386800 0.69895727581086
C 1.57428710488416 3.76101241257426 1.08260454965386
C 3.83648488191510 2.80642114485496 1.86639954321292
C 3.54143795163318 3.17871323281408 3.18225917932996
C 4.45421364734093 2.94239228126428 4.21493737744218
C 5.66941053011016 2.31352093694650 3.95114686134371
C 5.96638300033666 1.91545047354050 2.64298687023726
C 5.05872101288528 2.16221300521848 1.61465013065515
O 2.64028536332292 3.01738425653210 -2.09516890815665
O 1.35364784959697 -0.21560369851732 -1.40635175061894
C 0.18834464753737 0.19768240654856 -0.86224129405190
C -0.40553469459136 1.44135382662839 -1.12477447744064
C -1.59924638524884 1.77297424923552 -0.48534126834649
C -2.22001739228278 0.89685963012741 0.40214676726751
C -1.63903052177412 -0.35149860579544 0.66577666674421
O -2.19149896890359 -1.28524512869654 1.46564869539061
C -2.99645805424004 -0.99030617787857 2.62113684164130
C -2.55050444017786 0.21765854930346 3.44265394737878
C -2.93374206649285 -2.27505856292537 3.46846115410728
N -1.68123230428220 -2.63093010396525 3.79880925496642
C -1.36910610223231 -3.68725692421790 4.75108340679264
C -0.14306612788612 -4.47066396552088 4.26507578381284
C -1.14686598871040 -3.11860823397010 6.14743696895874
C -1.84970672804704 -3.62910337202930 7.24462389988454
C -1.62689513139505 -3.12816888488255 8.53153503080198
C -0.69507731285907 -2.10716319798087 8.73167455990194
C 0.00785207430903 -1.58788722783632 7.63863820577214
C -0.21646918144424 -2.08672431581866 6.35469446750758
O -3.96111222065813 -2.86808277229550 3.78784641854982
C -0.45362269202900 -0.70390483768713 0.00291006517822

I 0.37542323695612 -2.61749625983505
0.28786598938295
H 3.16647854997605 -0.95936697263765 -
3.15381114940707
H 4.07827691203599 0.58239863934341 -
3.13236013899287
H 3.94659385249131 -0.40547618191685 -
1.64302084777044
H 1.61256917997347 1.00676030953975 -
3.06851794640407
H 2.49374005465556 1.04360648625984
0.43156736272231
H 3.44080536892820 3.81423370674968
0.02935230606861
H 1.73419256898350 4.71448189476068
1.61026882533288
H 0.98721080187988 3.08390628867439
1.71994533901322
H 1.00582671386556 3.97915661830755
0.16670010716535
H 2.58530651258109 3.64637240865271
3.41562768946792
H 4.20052540496358 3.23628642667145
5.23579989150364
H 6.37749708101031 2.12086799497242
4.76092725916570
H 6.91214756197690 1.41139480640206
2.42473233032168
H 5.29380219145269 1.85055081063416
0.59363397207788
H 0.07314688894647 2.15829890428820 -
1.79016352723751
H -2.04896704617827 2.75039260843536 -
0.67655074721967
H -3.14536287092878 1.18245142201947
0.89941031286947
H -4.05224410257963 -0.89043410269086
2.31133101036924
H -2.98413720415777 0.12850494433673
4.45065853504157
H -2.89162826965310 1.16933349928580
3.01487809922897
H -1.45609923452454 0.26650138539402
3.52391734908384
H -0.89171030647050 -2.05353692116648
3.47252799170125
H -2.24822354795387 -4.34726299787491
4.78836921614486
H -0.34502203186592 -4.94921325788567
3.29320063511529
H 0.12907187609690 -5.25067376342112
4.99231792547958
H 0.71515255527266 -3.79334702288019
4.13693863540341
H -2.58560703157336 -4.42364543624048
7.08905597452316
H -2.18686688811288 -3.53472183275488
9.37835512568571
H -0.51874631909108 -1.71375784433218
9.73662267290648
H 0.74055436219629 -0.78989160313219
7.78188637959174
H 0.32652737981865 -1.66888848509993
5.50369083803582
C -4.44929765757671 -5.46942400329645 -
4.80576363051197
C -4.44629900791103 -4.06731958573129 -
4.83496972385652
C -3.79000974266576 -6.16493295509465 -
3.78479160139532
C -3.78726170351183 -3.35879627594567 -
3.84159877751754
H -4.96033688442887 -3.53695666380955 -
5.63841378075943
C -3.12685325830768 -4.05059443484055 -
2.80675241507066
H -3.78387924767532 -2.26906795219108 -
3.86530650872958
C -2.72308832736042 -3.29305742118370 -
1.36275666336060
C -3.13048383100704 -5.45886954588866 -
2.78674484342227
C -1.56517755406793 -3.24536995725750 -
2.21043855519092
C -1.14212425322340 -2.08076865564341 -
3.04388508913671
H -1.04116406103874 -2.35959142877620 -
4.10333693606792
H -1.81813076275492 -1.22515736075413 -
2.93337664518246
H -0.14794636950416 -1.77115604786108 -
2.68709861693326
H -2.63451577031069 -5.98286317431525 -
1.96785528779188
H -3.79759361795995 -7.25557005254975 -
3.77569389637678
O 0.57151831918637 1.19596503357854
2.94932001424387
S 1.41844201467591 -0.02265670301992
2.92229669551923
C 2.55342819744541 0.08873496854936
4.30446392249847
O 0.64128154870733 -1.27608657940645
3.21204852989327
O 2.26387976000822 -0.17116308035567
1.70038896833761
C 2.14915848848091 0.73708336432813
5.47127373411913
C 3.78411614627203 -0.56157168435222
4.23963339430178
C 2.98993849200529 0.72876481681896
6.58398478924408
H 1.18528383530759 1.24875216634963
5.49378716597565
C 4.23399822484430 0.07917842345632
6.54785376783256
H 2.67795420378618 1.24169860997013
7.49891413775789
C 5.15800329312540 0.10850510228418
7.73887389708201
C 4.61374427202890 -0.56375498018979
5.36019936250829
H 5.86253657371031 0.95716199612435
7.67234497135536

H 4.60105399447686 0.22098819329273
8.68192044065503
H 5.76237016991258 -0.80972117774127
7.80497435097752
H 5.58351738190199 -1.06760118365383
5.30964639412169
H 4.08338047113447 -1.04499185162811
3.30859179365340
O -2.64553953431111 -4.14375730371064 -
0.33703584896446
C -3.71650981581776 -4.31031339914142
0.62845925815216
H -4.60397311711565 -4.70120261136343
0.10758573354985
H -3.95528966984224 -3.33007669109899
1.06276208868254
C -3.20523635631926 -5.24091138425038
1.70053902770557
H -3.95119042334466 -5.30156350701818
2.50537880054892
H -2.28128048289495 -4.83572251104608
2.13445400275598
H -3.00954730617062 -6.24929130407381
1.30282995063188
O -3.39313268796627 -2.10182323650690 -
1.18920690530918
C -4.78011571603710 -2.04192741785293 -
1.13539600999886
O -5.47366316670167 -2.97603392867562 -
1.41616748950934
C -5.21348775497730 -0.68094399988446 -
0.69228335188962
H -5.03685239055377 -0.60082351656526
0.39205641444567
H -6.28277384809934 -0.55260264160884 -
0.89764976236976
H -4.60842455354498 0.10112398971065 -
1.17099414407268
H -0.89017490074437 -4.09339123004426 -
2.10334697761628
C -5.21181841111050 -6.22261997408826 -
5.87465284123214
F -5.03092436064784 -5.66113673896159 -
7.07970218213242
F -4.82458464154819 -7.50252585161470 -
5.95221427512440
F -6.52812974834264 -6.20764753485425 -
5.61667245983016

121
(S)-6-TS
C -0.62058125310668 3.19512454553231 -
3.13422884987416
C -0.10474873950340 2.12928964490635 -
2.17866677209636
C 1.34205576312844 2.43268476609596 -
1.77313822758892
N 1.47802697036341 2.96632236738423 -
0.55076129783867
C 2.77940180944157 3.30565761184050
0.01257585118462
C 2.61931183787024 4.48881900625702
0.97380235475373
C 3.42481609761622 2.10840392667040
0.70076257994376
C 2.69466829243118 1.32760712298246
1.61145237305615
C 3.29025809810901 0.22778575385648
2.23489114814582
C 4.62371357892793 -0.10214669921499
1.96593619920306
C 5.36186840916139 0.68196569714695
1.07493627559553
C 4.76143645484205 1.77895524025423
0.44696900226330
O 2.26964307650074 2.23123220966299 -
2.56211133332133
O -0.10560905360677 0.83544043438886 -
2.84335307324354
C -1.13799581553488 0.01406871904573 -
2.46087106106287
C -2.33474730217797 0.02532697310240 -
3.18628959081754
C -3.39680793184432 -0.77728707922646 -
2.77145491798539
C -3.29691869567731 -1.55435183101239 -
1.61695309626737
C -2.11357077831466 -1.53825191330541 -
0.86345516364127
O -1.94047146888541 -2.19029626552126
0.29855240176632
C -3.03018909335533 -2.53205606324732
1.16479317299744
C -3.93565424602375 -1.34804312091255
1.50411413131744
C -2.39709632414952 -3.04534522779915
2.46755151296527
N -1.40699460536706 -2.26074555880196
2.94868481827107
C -0.91883642904274 -2.43366517386530
4.31194132651310
C 0.48404168748501 -1.83283199883842
4.43373815703523
C -1.90138803445559 -1.83966516792850
5.31505707468874
C -2.49860414795402 -2.64576441542663
6.29027774256016
C -3.41686148558438 -2.10719529029279
7.19744111724715
C -3.75413831670510 -0.75332306707296
7.12983437153440
C -3.16933312383313 0.05755418275515
6.15058122741226
C -2.24908779451986 -0.48061484406920
5.24970227735593
O -2.83606505812620 -4.04021399123721
3.02711784543785
C -1.01064027174517 -0.81088721238640 -
1.34248617402723
I 0.79470338519750 -0.90312039849632 -
0.26607218189938
H -0.60754004424474 4.18025238893950 -
2.64189792343809
H 0.02325742294830 3.23828335411578 -
4.02659069681377

H -1.65563312606572 2.97605903069639 -3.43661062103238
H -0.75017187422012 2.05927319577991 -1.28928299282730
H 0.68281037799920 2.95608494226420 0.11224846076270
H 3.42531615690014 3.59548123188416 -0.83034679588080
H 3.58966865961446 4.75750453304469 1.41817397401589
H 1.92069409576941 4.23080772643424 1.78446705374863
H 2.21845801166016 5.36557048213720 0.44051644245261
H 1.65133653429254 1.56857639297647 1.82375861928093
H 2.70182772249593 -0.37274453246241 2.93293252139400
H 5.08702725613081 -0.96294496679949 2.45564242827736
H 6.40678764471319 0.43745721793145 0.86357669234918
H 5.33735513378317 2.38248313667911 -0.26144225291140
H -2.41271442778610 0.66802722097339 -4.06471797742909
H -4.32743116420016 -0.78569197333354 -3.34421053404774
H -4.14103417308253 -2.15967839921838 -1.28620106247809
H -3.60085888638142 -3.37407578463214 0.73847267927476
H -3.33271350400349 -0.51648146787829 1.88734786673826
H -4.64191602033743 -1.66476027960698 2.28625095673137
H -4.50733273479530 -0.98528265621563 0.63994023105222
H -1.19333350675890 -1.36922899032831 2.49080982759367
H -0.87317485926309 -3.51749893094698 4.49598573187129
H 0.46853150792572 -0.76014799663352 4.18779682131456
H 1.17427142993657 -2.33630705195801 3.73744728952127
H 0.86531306796363 -1.95363038788187 5.45915168351735
H -2.25434491614236 -3.71095154732863 6.32671529816548
H -3.87788770968074 -2.75099509618040 7.95187064222695
H -4.47659096453555 -0.33134154537067 7.83429935037081
H -3.43107597287165 1.11552223274962 6.07970639733521
H -1.80515785961864 0.15080925700353 4.47721386406779
C 2.80134712039762 2.51253461553146 -8.51980901878105
O 3.73266658276293 1.60740754344933 -7.94381172612988
H 1.93683049844455 1.97694555728191 -8.94659428804842
H 3.33798085448439 3.03420405044564 -9.32171713978441
H 2.44920825377767 3.24724612026591 -7.77673519285165
C 3.36590683648879 0.83005995068038 -6.92476343765209
C 2.07526826003852 0.81690916749233 -6.36036625317359
C 4.37787650964954 -0.01176094888684 -6.39872550416128
C 1.80543909845266 -0.02298510394230 -5.29387273573058
H 1.29083309454517 1.47577939563878 -6.72966349744415
C 2.80439128801001 -0.87837518852280 -4.76876790612654
H 0.82570323745631 0.00326771770779 -4.81564289271285
C 2.41440577071855 -1.84004283817301 -3.60635085349789
C 4.10518221242977 -0.84207609371549 -5.33789292474675
C 2.75002048011839 -0.63145016805535 -2.86907676107414
C 4.05402847236588 -0.32878301406418 -2.24374423792313
H 4.83460981453059 -1.06498567813663 -2.46746611544276
H 4.35707896175944 0.69495250588843 -2.50280771628464
H 3.89116830050335 -0.31219412527369 -1.15303822081638
H 4.89864540253345 -1.47027207199471 -4.93426635960733
H 5.36790625331842 0.02725277583144 -6.85554657876849
O -2.06687891476727 1.40584656046556 0.44166710707771
S -1.34735718376494 1.73229240241882 1.69922575298441
C -2.55691022897633 2.44978236251972 2.81455754748656
O -0.81220691992959 0.53381954825901 2.42020016173918
O -0.30480246091217 2.79913893027672 1.53139182325367
C -3.91758137008572 2.34267558218819 2.53300063340199
C -2.11960970554105 3.07321514519343 3.98599624103543
C -4.84802534067988 2.84851585185277 3.44355520245019
H -4.23088376843915 1.87291509016094 1.59948618427430
C -4.43727049859443 3.46723732734276 4.63287609055058
H -5.91624011475116 2.76293803335392 3.22358782488255
C -5.44487586921318 3.97001340350131 5.63530183713156

C -3.05850840843472 3.57889648761802
4.88287629296587
H -5.06807610151863 4.84803588664691
6.18260267639929
H -6.39474057057225 4.24659335120444
5.15250962556808
H -5.67416602348955 3.19203076465910
6.38543373896324
H -2.71684324987006 4.06940880924599
5.79913143138222
H -1.04980127423787 3.16458948759735
4.18316922155282
O 1.10272838018046 -2.18886939005067 -
3.53618194669286
C 0.55342431903108 -3.09460856025319 -
4.50777252603474
H 0.93490940930712 -4.10955953150293 -
4.30737421433638
H 0.89357938005760 -2.80864880930924 -
5.51674258012759
C -0.95312814010398 -3.05455505653564 -
4.38089608611015
H -1.40118063670626 -3.80017677666183 -
5.05637901066892
H -1.35018686803003 -2.06282108355351 -
4.63761572365732
H -1.26616133867205 -3.28091300806427 -
3.35097175073583
O 3.32606642257856 -2.89578460996694 -
3.36336975207279
C 3.66552795235745 -3.80780556893231 -
4.32620915051966
O 3.30909251015367 -3.73645708253127 -
5.47029273927952
C 4.54353960999861 -4.87236897652193 -
3.72917231504774
H 4.84337441019363 -5.58222604696353 -
4.50896977634107
H 3.99597674175834 -5.39452397636570 -
2.92906248223134
H 5.43098713587611 -4.41472061858950 -
3.26536321776655
H 1.95486884830479 0.10791562178769 -
2.77770497750690

37
(R)-INT-7
C -3.02677094059035 -1.30438619698576
2.59074143271970
C -1.61217213004952 -0.72895617339780
2.54077426946294
C -1.34076232905791 0.32132615441597
1.45419086557921
C -0.45534591265932 1.38450408902308
1.72369271066973
C -0.18041365525873 2.34117953516659
0.76086007981301
C -0.77519417271315 2.25664148960368 -
0.51660243518508
O -0.45699475888811 3.22878878524897 -
1.37886076067944
C -1.03242484312954 3.24568709127854 -
2.67613882521308
C -1.64374946261387 1.18639235190744 -
0.80163154474756
C -1.90867547018957 0.22819846920978
0.17616799831449
C -0.55870075662333 -1.73059842644313
2.21833561469900
O -0.80631027817724 -2.62410492555998
1.29194345479053
C -0.47054834616915 -4.04825700777023
1.40042408938083
C -0.80651121501897 -4.71279051123452
0.11424642228309
O -0.04730413301328 -4.46738011213592
2.41985316204957
H -3.12456624568370 -2.03560678753596
3.40770873173715
H -3.73989644980711 -0.48799970465786
2.77244210885759
H -3.30678444592063 -1.79909117147834
1.65052731876311
H -1.33725229676853 -0.29314188246186
3.51196080012457
H 0.00442910534527 1.47360158893429
2.71186931725454
H 0.48207799810317 3.18363063260740
0.96758961073147
H -2.13229877863850 3.31744895022915 -
2.62539008857667
H -0.74921246780201 2.34860582165902 -
3.25314915453830
H -0.63499738186758 4.13692466676648 -
3.17718783578484
H -2.12035460855665 1.09692721100441 -
1.77728433069632
H -2.58506229022707 -0.59234371137617 -
0.07093466250836
H -0.26674417293708 -4.22607150268065 -
0.71300623551552
H -1.88387256456927 -4.59984535324514 -
0.08587239827429
H -0.54314074063439 -5.77532894422636
0.17767434578447
O 0.57273333650358 -1.61476070535575
2.78634381500007
C 1.84919219980801 -2.21407604899449
2.33442364698326
H 1.74520273313427 -2.44742455590696
1.26451941615965
H 1.95717542075779 -3.14392798601845
2.90714987077459
C 2.93706171163608 -1.20611840094481
2.60836217842452
H 2.99319863927007 -0.96564488792634
3.68004269252023
H 2.77827206031374 -0.27996823291612
2.03642074521966
H 3.90215509269261 -1.64030790680221
2.30366145462247

36
(R)-INT-7-CF3
C -2.99536504762872 -1.24883433800756
2.58138183318731

C -1.56759330057482 -0.70221964181010
2.50922816075216
C -1.32531579923224 0.37152886422806
1.43655347397580
C -0.58995185777261 1.51912628825776
1.76441632845538
C -0.34749312966267 2.49907561740029
0.80233489386416
C -0.82656328285711 2.32890252868205 -
0.50004129139816
C -1.55664622028318 1.18435843705918 -
0.83620029914366
C -1.79861832639195 0.20544473577975
0.12660694223919
C -0.54255346904487 -1.74737617084192
2.20045955831019
O -0.84256853276380 -2.67163584494193
1.33664958000721
C -0.47605860557205 -4.11261934579002
1.43555316685087
C -0.82342840702236 -4.76832231011841
0.15107016661462
O -0.04213936899764 -4.51550732195709
2.45283223073345
H -3.09086127778636 -1.99869731355517
3.38160390049215
H -3.68396847004225 -0.42169505986403
2.80377952135831
H -3.30982353293193 -1.70936696989197
1.63488650202172
H -1.26972496255644 -0.27521635881279
3.47797993635681
H -0.22025939551156 1.66082730117544
2.78311807322649
H 0.20470452319006 3.40267981095444
1.06570754479954
H -1.94193188511641 1.06322059143031 -
1.85023868479483
H -2.36995890235510 -0.68249615051718 -
0.15055307707811
H -0.29409294901491 -4.27466462922353 -
0.67902246953553
H -1.90355049223328 -4.65686292736661 -
0.03549389727663
H -0.55594042620818 -5.83053292820387
0.20583942877926
O 0.60154300328268 -1.62014491549776
2.73337941325044
C 1.86408628793894 -2.28549923227630
2.32059652590399
H 1.75719890251216 -2.56077641448224
1.26084783181576
H 1.93396212556129 -3.19186618681416
2.93520776961468
C 2.98171524946926 -1.30305496370324
2.56081611141232
H 3.04119104203863 -1.02209326060267
3.62234533541792
H 2.85826403093120 -0.39621344394459
1.95065266940011
H 3.93267826138395 -1.78210387577060
2.27958452999661

C -0.51513885456854 3.35851252090293 -
1.56516525001231
F -0.29646155052327 4.56637505448693 -
1.03117156812895
F 0.58867556266354 3.00903367050430 -
2.24794902793270
F -1.51759905631943 3.46490951313228 -
2.44726527753561

19
p-TsOH
O 3.17468659307347 -0.55126625246987 -
1.78924144713584
S 2.86529102393875 -0.37635877244625 -
0.38122159876412
C 1.10201846403584 -0.22429181206292 -
0.19528453093003
C 0.50158950823422 -0.62800041243213
0.99692933983240
C -0.87976224253223 -0.48549731668047
1.13797106207417
C -1.66346195427914 0.05196528695451
0.10585608919276
C -3.15658049772042 0.19106707665433
0.25182039305946
C -1.02647917947739 0.44297904973831 -
1.08581214430752
C 0.34949396663281 0.30602703444261 -
1.24725344169650
O 3.38085676906650 1.13953127166699 -
0.00536259496426
O 3.37876397279011 -1.26530368908309
0.65962633529179
H 1.11111480013607 -1.05893454365112
1.79311124479417
H -1.35872110743788 -0.80252017689672
2.06819634657531
H -3.68341078029792 -0.52740633010805 -
0.39949602977343
H -3.48276755962323 0.00689990631541
1.28583059585536
H -3.49456710342535 1.19828850156897 -
0.04100336959330
H -1.62287049724389 0.85793265258938 -
1.90317359491196
H 0.84268082515013 0.59554487873922 -
2.17678605244702
H 3.66202599897950 1.12944464716092
0.92539239784856

18
p-TsO-
O -3.55174047479573 -1.36783587305708 -
0.37856260394505
S -3.15922374928475 -0.07240431472717
0.23750012006190
C -1.33587493286624 -0.06838096084149
0.12796160398641
C -0.69286480143267 -0.73190340388876 -
0.91974119615870
C 0.69722638000605 -0.67443583008292 -
1.03786638467302

C 1.47481881238276 0.04326782102668 -0.11468870617009
C 2.97463042058811 0.13240308018350 -0.26660979652320
C 0.81379597846748 0.69214492339429 0.94028448576204
C -0.57644451282149 0.63765386150052 1.06337662489412
O -3.54815804009888 1.12614854952938 -0.54999162175444
O -3.43035859708742 0.03466306751124 1.69580844487701
H -1.30620985189537 -1.30410259876614 -1.61977664109071
H 1.19404588057847 -1.20322119989510 -1.85959559744341
H 3.47066507596738 0.30841586630103 0.70200136445799
H 3.39503207186876 -0.79062342606167 -0.69950559904364
H 3.26754180415564 0.96321409448889 -0.93566263550106
H 1.40280977916729 1.24355407709436 1.68228054073694
H -1.09899424289938 1.12080226629044 1.89234759752693

8
AcOH
C -0.96078607902924 -0.09987347752548 0.01025296768704
C 0.48962060670616 0.22519778660126 -0.23940862739801
O 1.29598642898418 -0.37432315151582 0.66456850922553
O 0.91577887492726 0.92683896336874 -1.12151600740280
H -1.25033571050197 0.23521104222026 1.01860619440328
H -1.11173878241866 -1.19010271783206 -0.02449666294314
H -1.58432157961937 0.39308735998010 -0.74477196697957
H 2.20589624095164 -0.11613580529699 0.43676559340768

21
Alkyne (15)
C -3.16154726145356 1.63485982840823 2.19663247500808
O -2.98503075976878 0.42557308253391 1.49570797031011
C -1.79866649000609 0.18427759110946 0.88571925470579
C -1.69262043359611 -1.03714131524843 0.19715470470505
C -0.51813801086232 -1.37358268974061 -0.45993836693176
C 0.59525702306504 -0.50293871727928 -0.45392608461446
C 1.80544095610283 -0.85033278651566 -1.13307654401790
C 2.83308103085086 -1.14878123490981 -1.71038184443299
C 4.06490655165135 -1.50546515933971 -2.40435236002947
C 0.47461735150728 0.71448621168383 0.23938399606573
C -0.70340851446016 1.06127702878594 0.90330397381200
H -4.18369764887383 1.61883665675669 2.59873591041312
H -2.44844207336274 1.73236874859257 3.03576616745723
H -3.05055924344469 2.51348143524352 1.53499320653753
H -2.55571403347287 -1.70601641256228 0.19502237542490
H -0.44611038498056 -2.32431644163531 -0.99237226216366
H 4.95363167644177 -1.18396802582110 -1.83518322608183
H 4.11917918467443 -1.03222446729984 -3.39999125766345
H 4.13985422550994 -2.59620322230399 -2.55064661730076
H 1.32322207126454 1.40174511362143 0.25671501908632
H -0.75486221678632 2.01491977592043 1.42874950971041

9
EtOH
C 0.97133346346819 0.22146234427500 -0.13027713661883
C -0.38339234680605 -0.35118074420520 0.24411429767897
O -1.35514927936015 0.66329273753623 0.08031486038536
H 0.96434624648464 0.57145456177685 -1.17484056046479
H 1.21597218686175 1.08111950791721 0.51375486179605
H 1.76303133105259 -0.53661411032381 -0.01934081839980
H -0.60433984462776 -1.23159979906291 -0.39611710449387
H -0.35437460947291 -0.71972431842560 1.29156803741478
H -2.21742814760031 0.30178982051225 0.32012256270214

66
Ph*I
C 3.59502211673931 -3.72602829901082 3.14288378404487
C 3.03906407131729 -3.23603874885175 1.81663545109921
C 3.92543392768982 -2.18217537314208 1.12023034972456
N 3.34974410718959 -1.65128747386267 0.00915328731215
C 3.85710747089820 -0.44490280031705 -0.64223989879165

C 4.40226379015643 -0.75249572633695 -
2.04177463396787
C 2.78465940908762 0.63318813249717 -
0.65466062535816
C 2.98560492634624 1.85046340792234
0.00683436669306
C 2.01221804632565 2.85344170767647 -
0.02854595318695
C 0.81577349770943 2.64649687455570 -
0.71877942414199
C 0.59558451542631 1.42553076860359 -
1.36309580943495
C 1.57382035332042 0.43207992267419 -
1.33473663294278
O 5.02514016168183 -1.87123004851462
1.54497023243770
O 1.67977310606058 -2.78019884585459
1.88412526810391
C 1.37678944483556 -1.50058889244585
2.22852304699758
C 2.18167591754637 -0.68352090163878
3.03766735038823
C 1.77558333048408 0.61969844589643
3.31448045039176
C 0.58041777510496 1.13615649474782
2.81773745042849
C -0.22560480242520 0.33288068920492
2.00047089966249
O -1.39226137184959 0.73704134062358
1.45198892675460
C -1.81780118024159 2.09586054697129
1.48124684674345
C -2.63633411822513 2.40487668336903
2.72895618678784
C -2.64707492997086 2.36577153407753
0.21483995729579
N -2.79573293197410 1.31214980191928 -
0.61804659540882
C -3.65331471289421 1.35627096715104 -
1.80481570995274
C -3.05921083089731 2.23651807588860 -
2.91191873554372
C -3.91974366587740 -0.07102242110442 -
2.24721449103750
C -5.19517384946528 -0.63394997611970 -
2.11669770746929
C -5.43388971083606 -1.96150123349787 -
2.48415355029222
C -4.39272220872716 -2.74808941642633 -
2.98260435605707
C -3.11436118802138 -2.19722148337154 -
3.11713511671620
C -2.88220086340729 -0.86895301899713 -
2.75471646964095
O -3.12538060473276 3.47538844511986
0.02583475708550
C 0.18606587806099 -0.97742849692824
1.70247083952581
I -0.95641866480997 -2.12544114537906
0.34376218618457
H 2.90960563889259 -4.46977820410554
3.57525155164290
H 3.74334608487319 -2.91657315934373
3.86888349091406
H 4.57330486926960 -4.19775262257386
2.97202902876077
H 2.97163373065630 -4.09920556740051
1.13236636779544
H 2.40003162322017 -1.93773020723867 -
0.20601492452826
H 4.69126770643024 -0.10889892520520 -
0.01006176740421
H 5.20128287504362 -1.50719345635855 -
1.97955052907610
H 4.81224889902209 0.15866068110923 -
2.50460800285990
H 3.61095004962707 -1.14070247662131 -
2.70351502761501
H 3.91761687427847 2.01498177073674
0.55418931042957
H 2.19097441130588 3.80095373580331
0.48651073127022
H 0.05601829155935 3.43124147439364 -
0.75986532747759
H -0.34520983551814 1.25085458979487 -
1.88486280552374
H 1.38480471302731 -0.51200545943689 -
1.85344936775536
H 3.12648766505655 -1.04947836327095
3.43171398350296
H 2.40957066560081 1.25085629439274
3.94163124417202
H 0.28385282997327 2.15578936814924
3.05861441773991
H -0.94040079140806 2.75935293845441
1.41690985092864
H -3.01740642713125 3.43355618245675
2.66284598403759
H -3.49075997249686 1.71400200698542
2.79823885586962
H -2.02961032866194 2.29964099392547
3.64027690725239
H -2.39939326955642 0.42375045597158 -
0.32502743199625
H -4.60898262571049 1.81551112318019 -
1.49985991872531
H -2.10266464317272 1.83526502086504 -
3.28155329924486
H -3.75538108605154 2.29412148989484 -
3.76314652525331
H -2.89186174218359 3.25109882286869 -
2.52417299585888
H -6.01084411317906 -0.02486095302411 -
1.71710828644674
H -6.43643775869604 -2.38337057482707 -
2.37591306580580
H -4.57625112868973 -3.78730022312292 -
3.26738935695567
H -2.29414184423136 -2.80457863555081 -
3.50905218265128
H -1.87816757177475 -0.45244765700162 -
2.86378683685653

NMR spectroscopic data

2-Iodobenzene-1,3-diol (S2)

^{1}H NMR (300 MHz, $CDCl_3$)

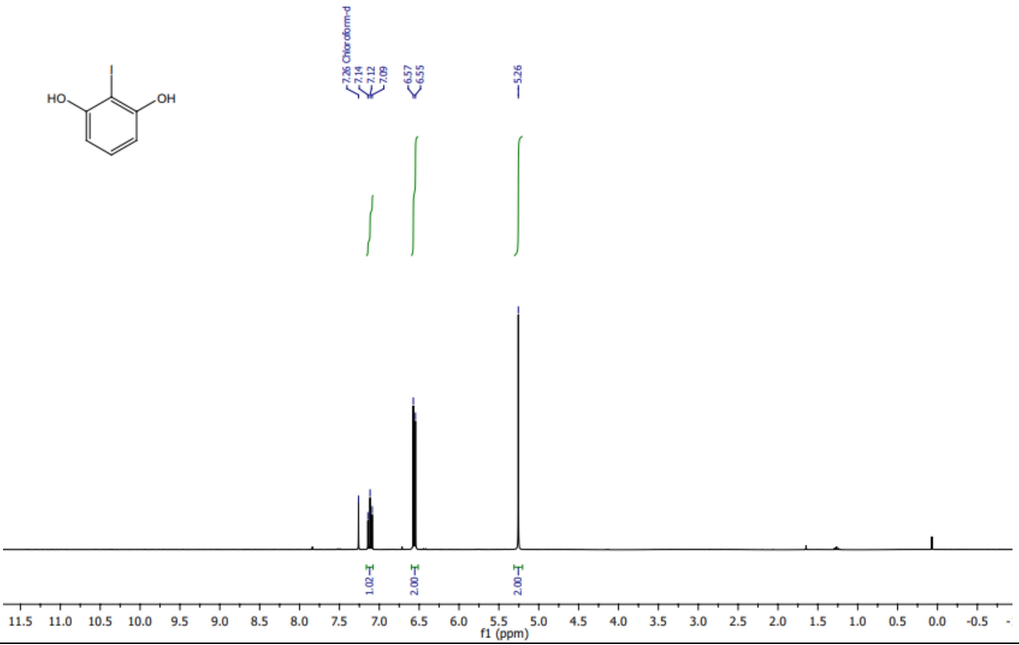

^{13}C NMR (75 MHz, $CDCl_3$)

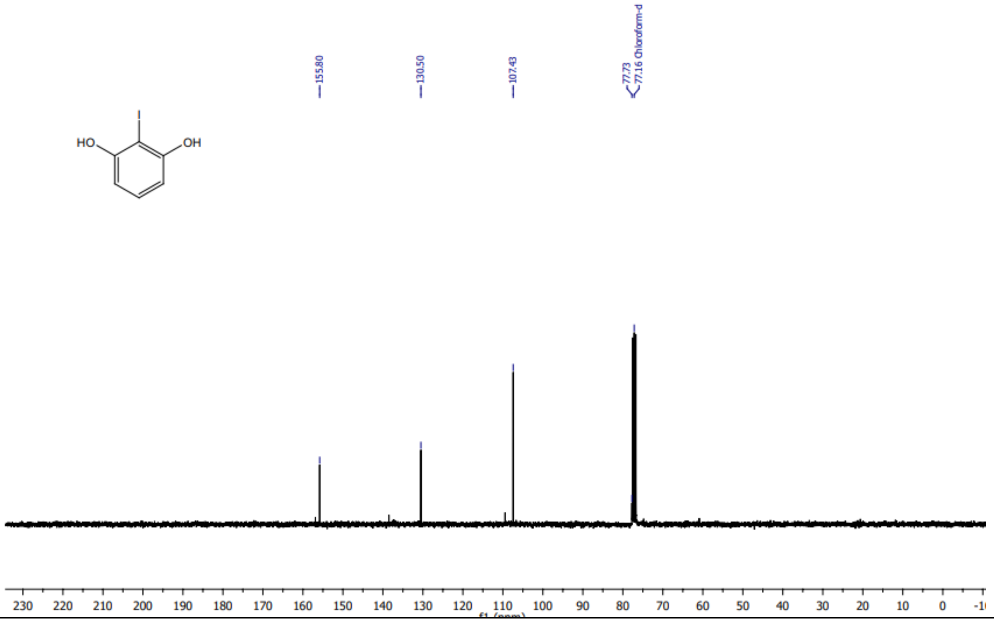

Dimethyl 2,2'-((2-iodo-1,3-phenylene)bis(oxy))(2*R*,2'*R*)-dipropionate (S3)

^{1}H NMR (500 MHz, $CDCl_3$)

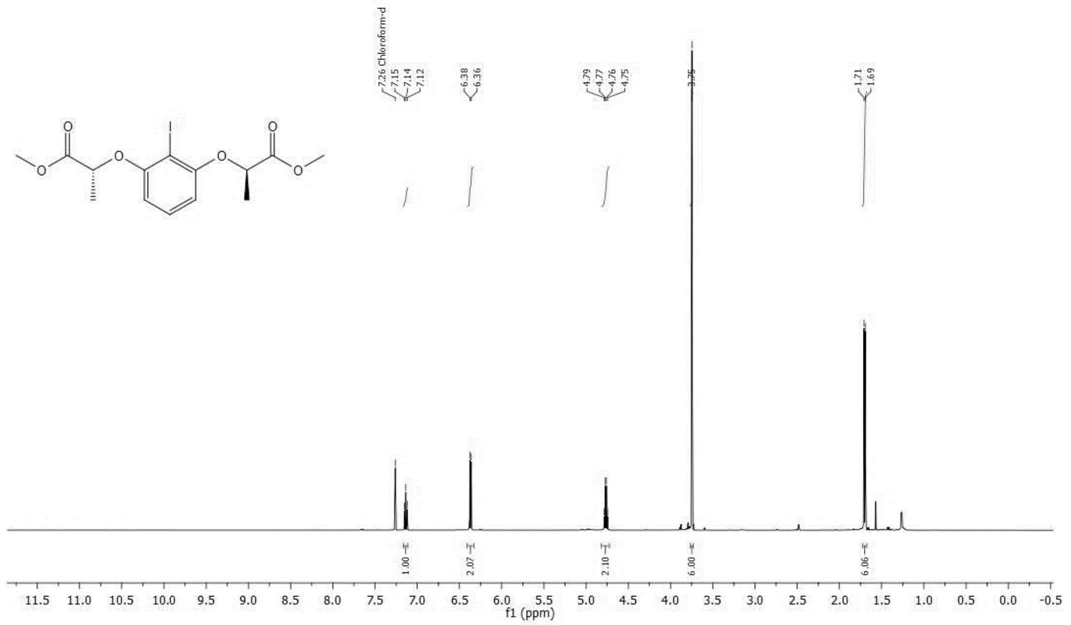

^{13}C NMR (126 MHz, $CDCl_3$)

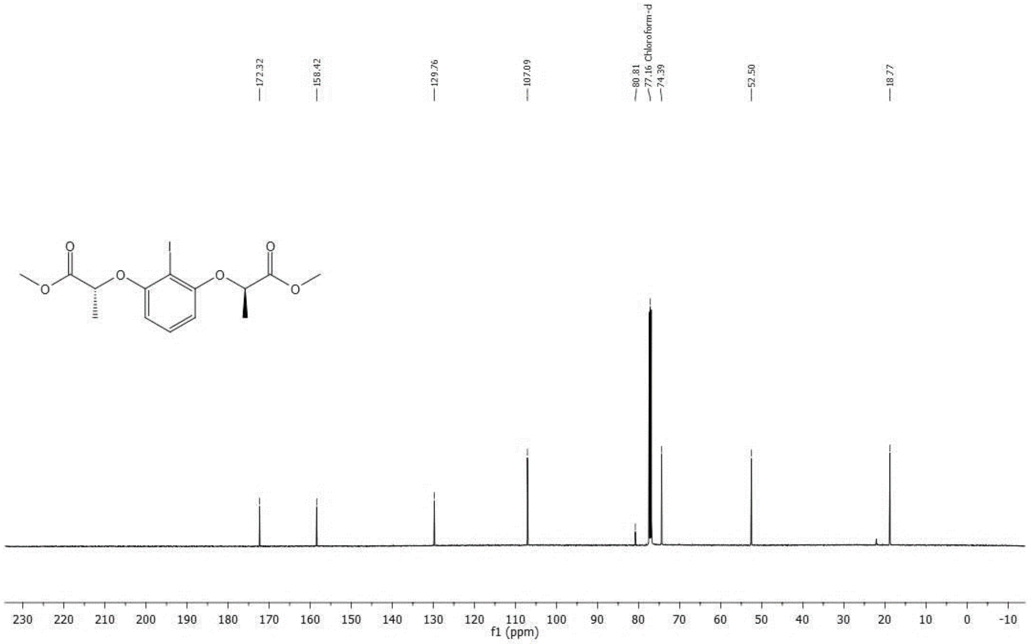

Diethyl 2,2'-((2-iodo-1,3-phenylene)bis(oxy))(2*R*,2'*R*)-dipropionate (S4)

^{1}H NMR (500 MHz, $CDCl_3$)

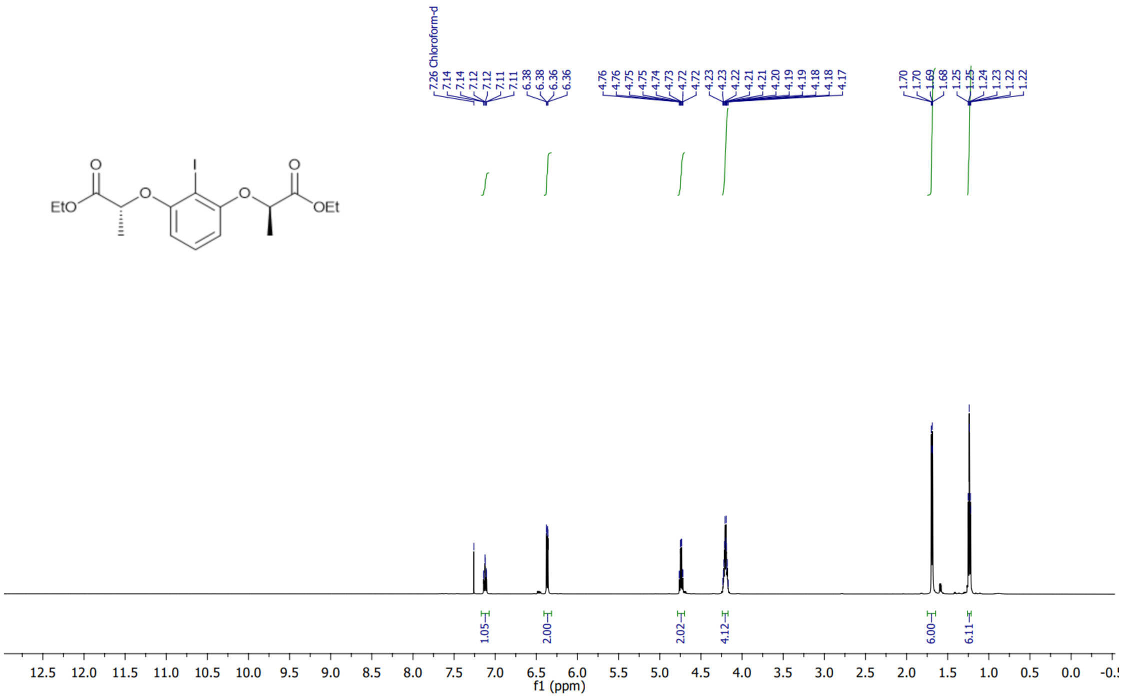

^{13}C NMR (126 MHz, $CDCl_3$)

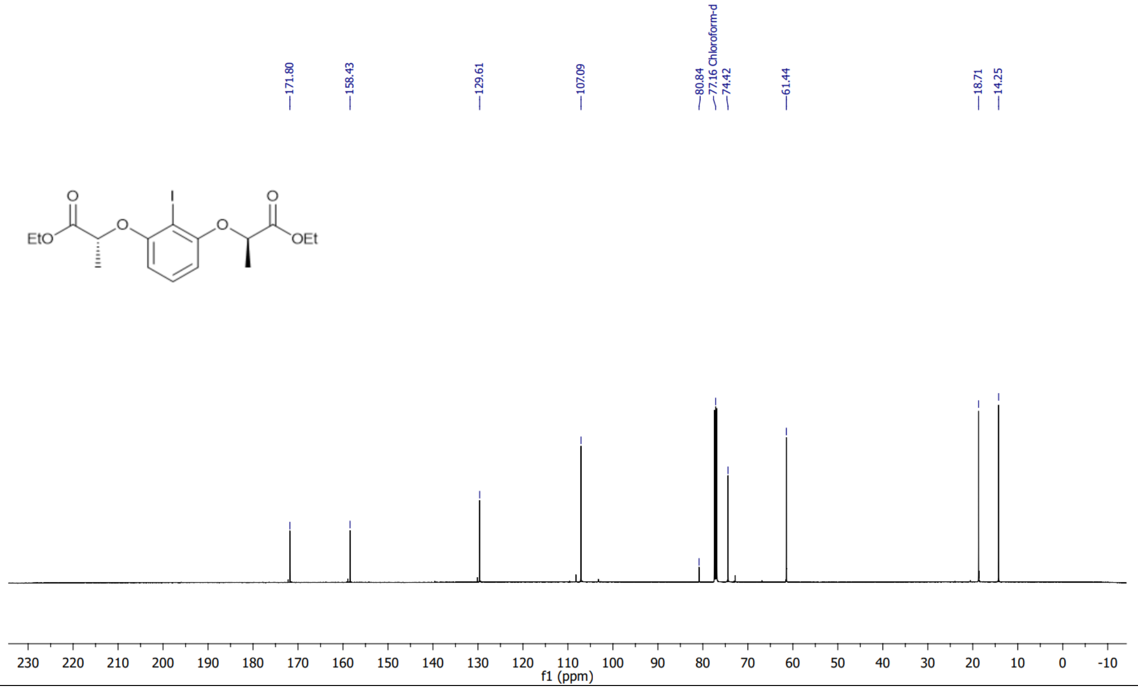

Diisopropyl 2,2'-((2-iodo-1,3-phenylene)bis(oxy))(2*R*,2'*R*)-dipropionate (S5)

^{1}H NMR (500 MHz, CDCl$_3$)

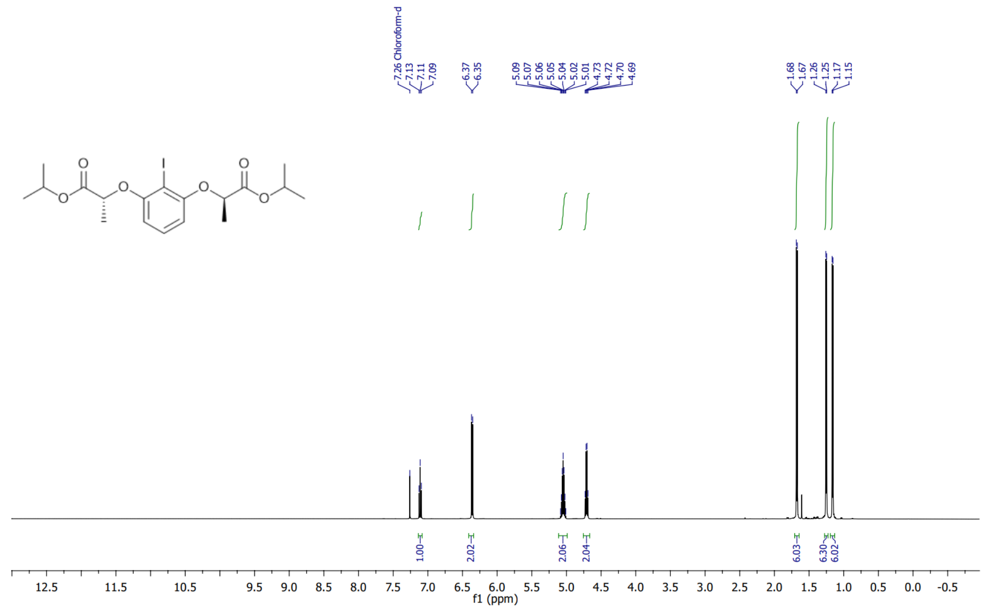

^{13}C NMR (126 MHz, CDCl$_3$)

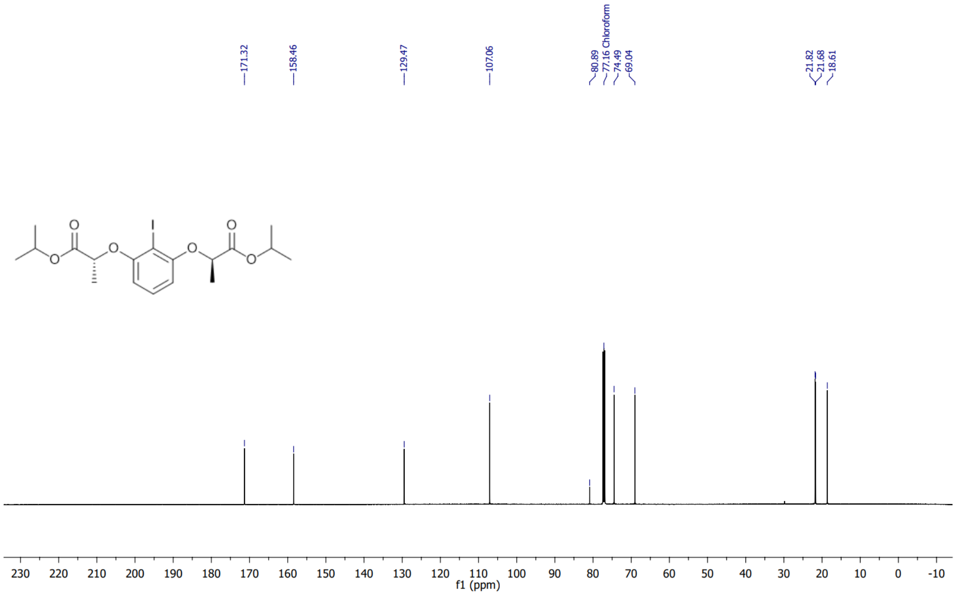

(2*R*,2'*R*)-2,2'-((2-Iodo-1,3-phenylene)bis(oxy))dipropionic acid (S6)

^{1}H NMR (500 MHz, DMSO)

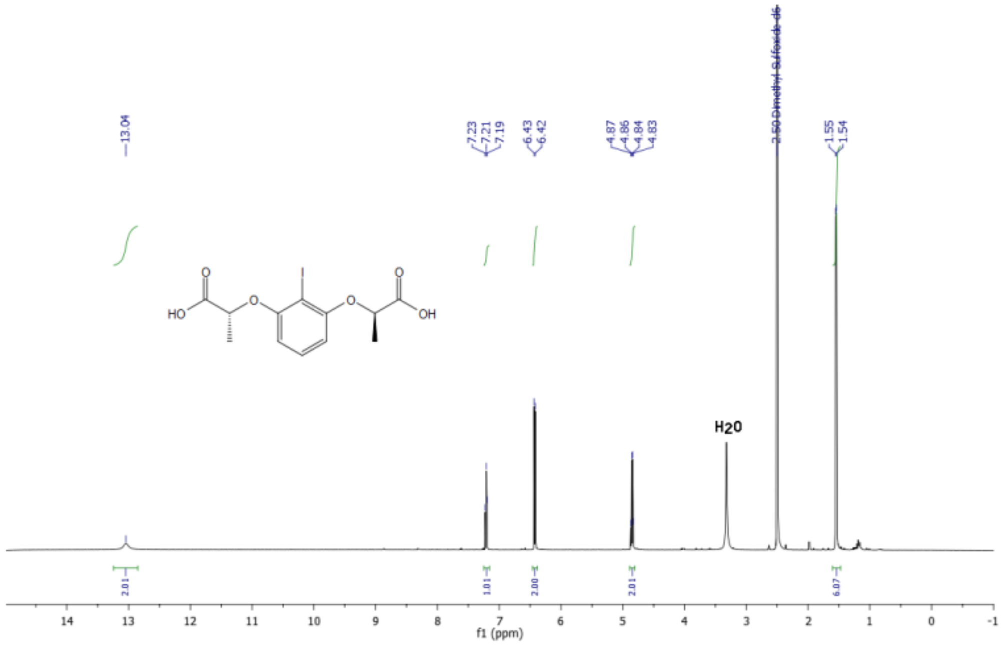

^{13}C NMR (126 MHz, DMSO)

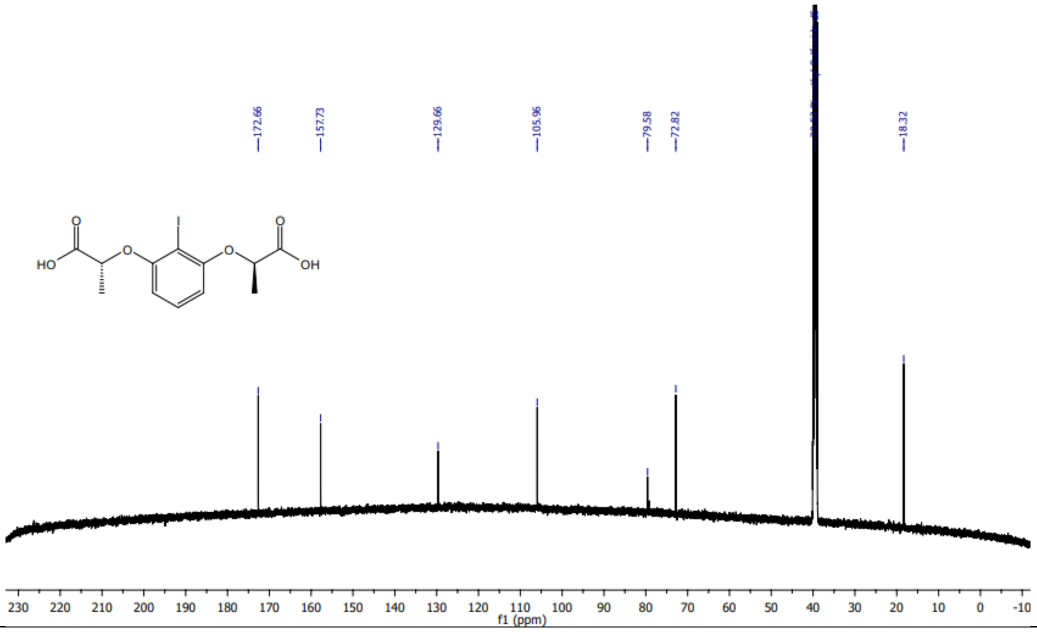

Bis((1*S*,2*R*,5*S*)-2-isopropyl-5-methylcyclohexyl) 2,2'-((2-iodo-1,3-phenylene) bis(oxy)) (2*R*,2'*R*)-dipropionate (S7)

^{1}H NMR (300 MHz, $CDCl_3$)

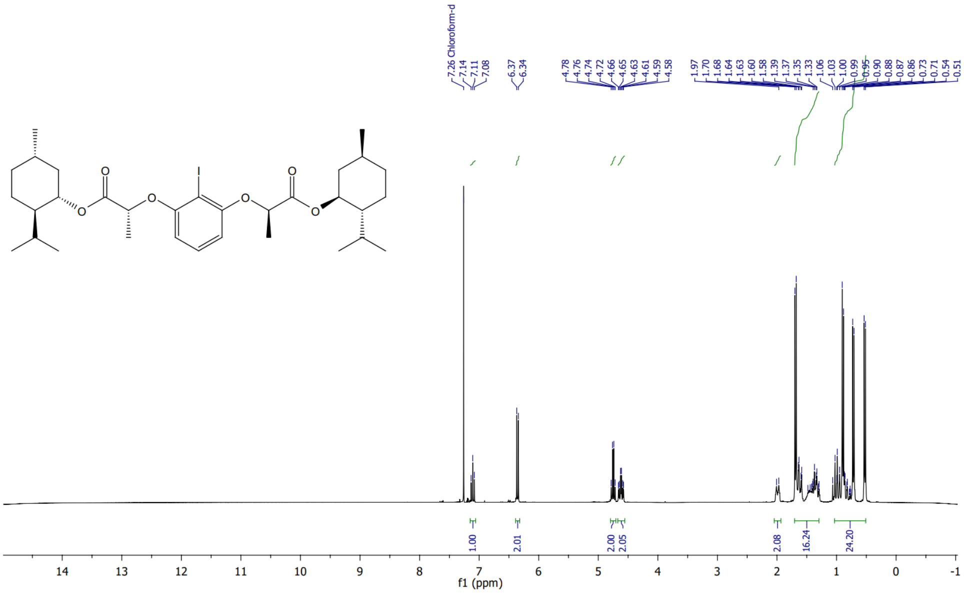

^{13}C NMR (75 MHz, $CDCl_3$)

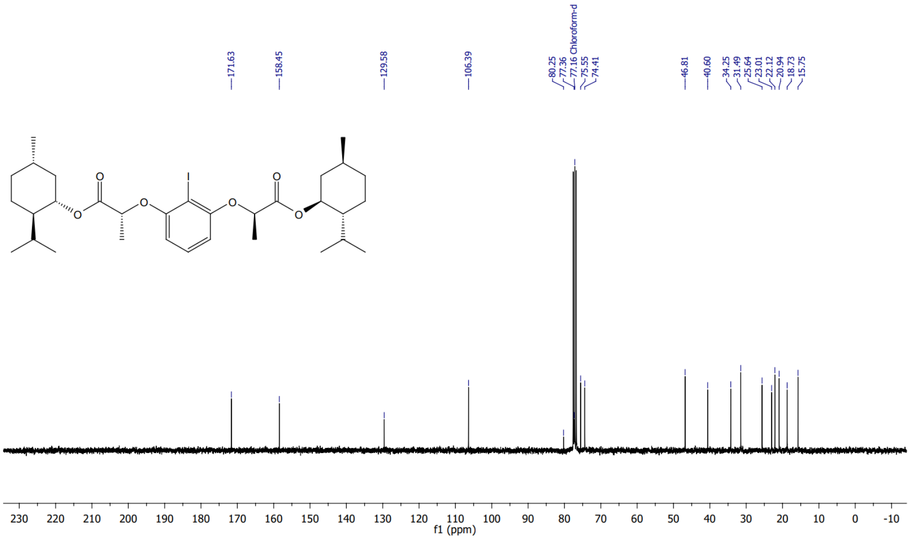

(2*R*,2'*R*)-2,2'-((2-Iodo-1,3-phenylene)bis(oxy))bis(*N*-tosylpropanamide) (S8)

^{1}H NMR (500 MHz, $CDCl_3$)

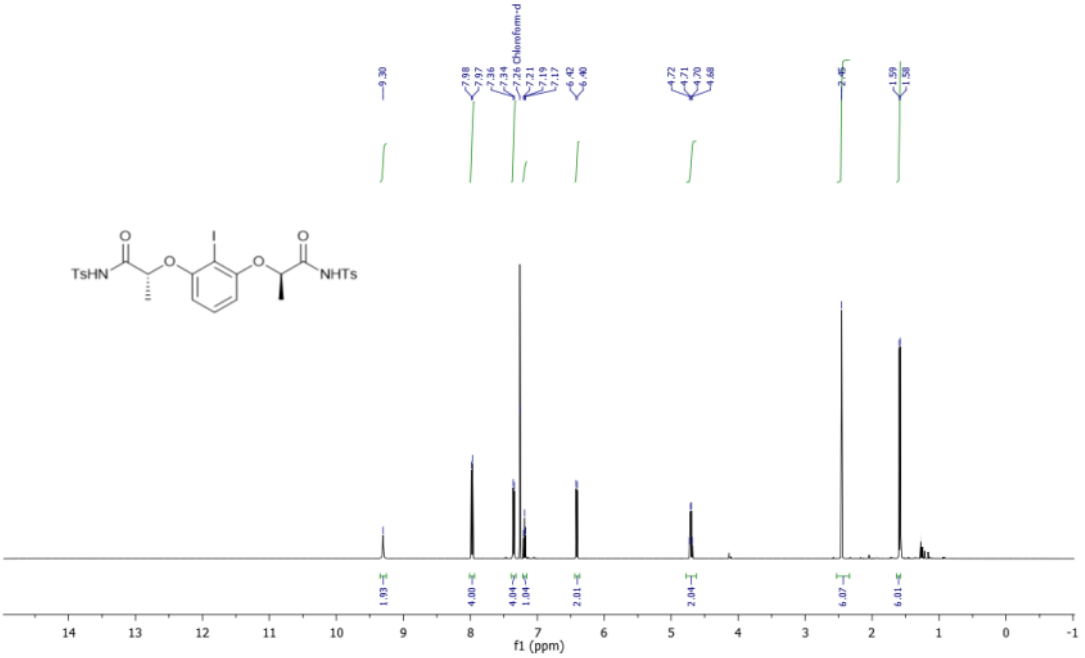

^{13}C NMR (126 MHz, $CDCl_3$)

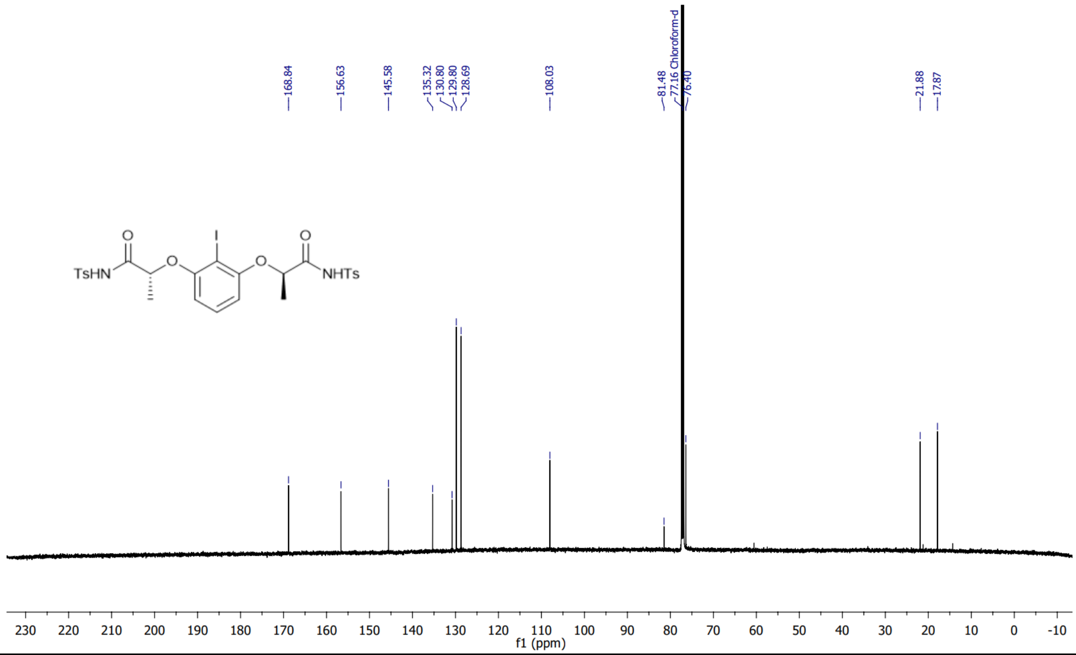

(2*R*,2'*R*)-2,2'-((2-Iodo-1,3-phenylene)bis(oxy))bis(*N*-(3,5-bis(trifluoromethyl)phenyl)-propanamide) (S9)

^{1}H NMR (300 MHz, $CDCl_3$)

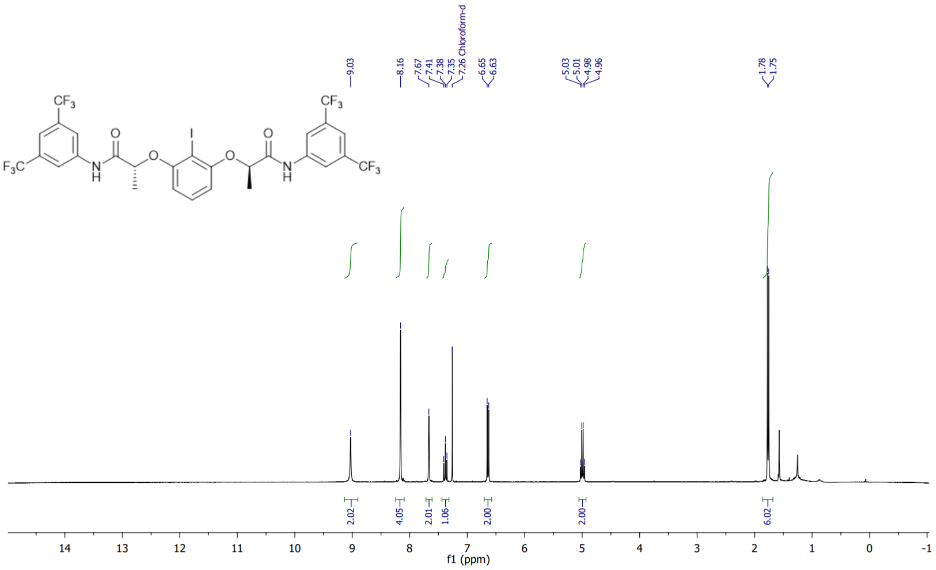

^{13}C NMR (75 MHz, $CDCl_3$)

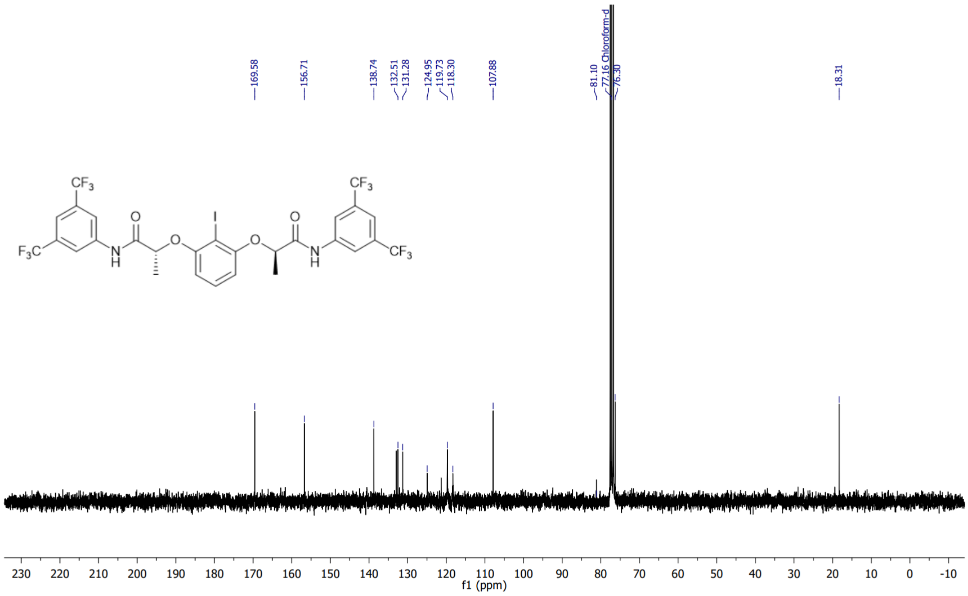

(2*R*,2'*R*)-2,2'-((2-Iodo-1,3-phenylene)bis(oxy))bis(*N*-(perfluorophenyl)propanamide) (S10)

^{1}H NMR (300 MHz, $CDCl_3$)

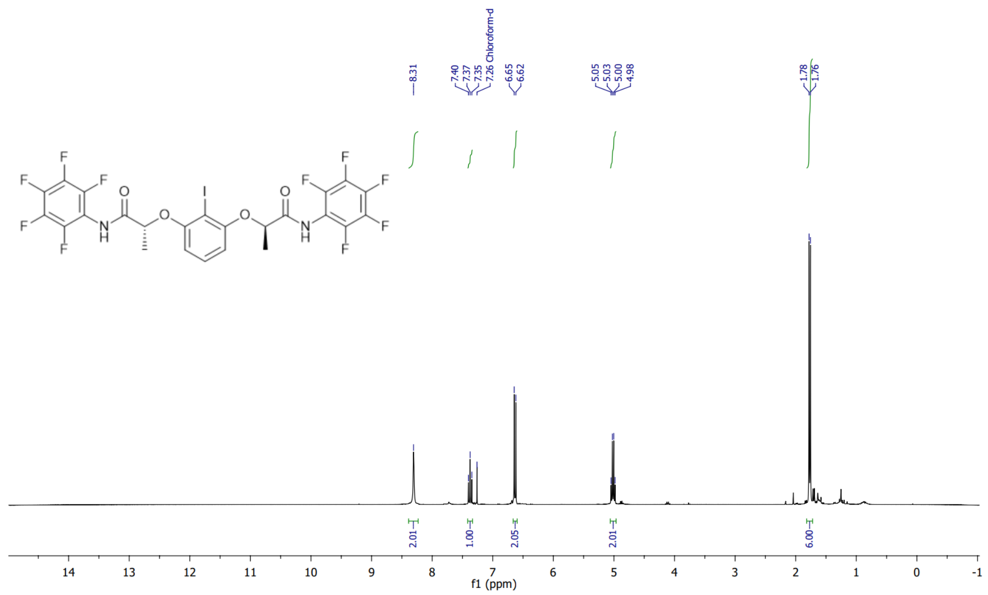

^{13}C NMR (75 MHz, $CDCl_3$)

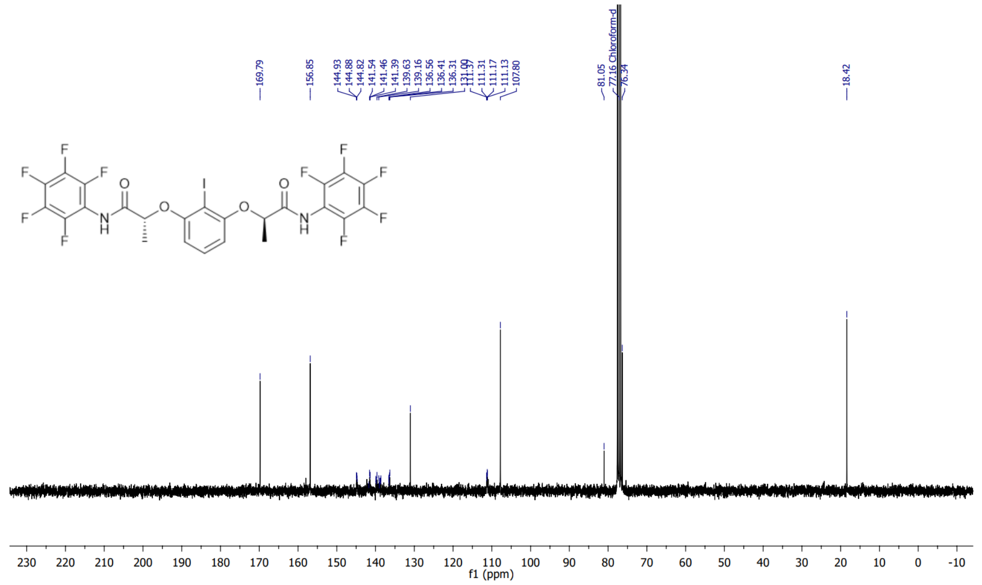

(2*R*,2'*R*)-2,2'-((2-Iodo-1,3-phenylene)bis(oxy))bis(*N*-(2,6-diisopropylphenyl)-propanamide) (S11)

^{1}H NMR (500 MHz, $CDCl_3$)

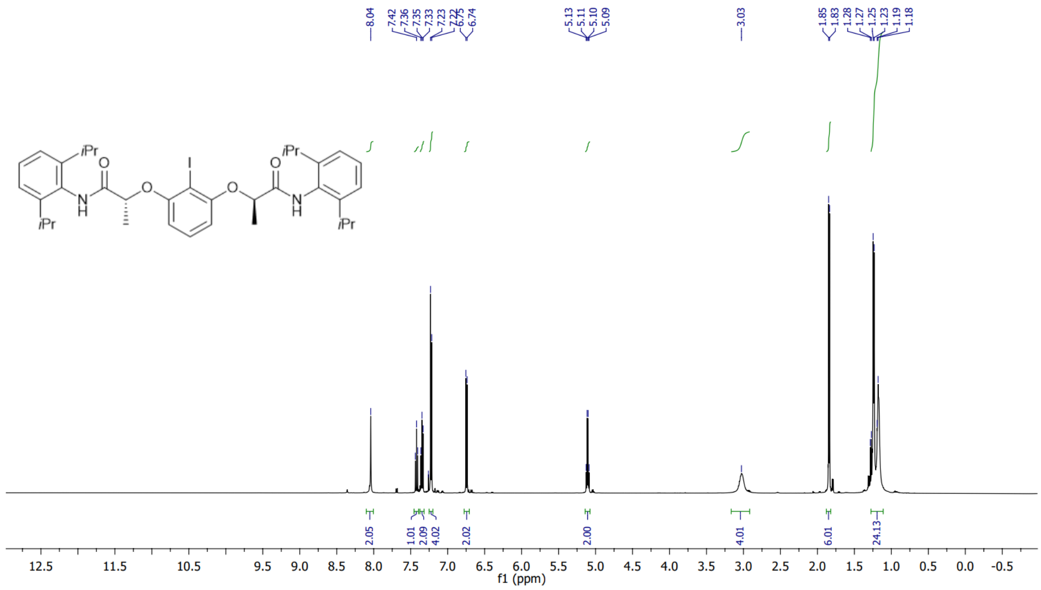

^{13}C NMR (126 MHz, $CDCl_3$)

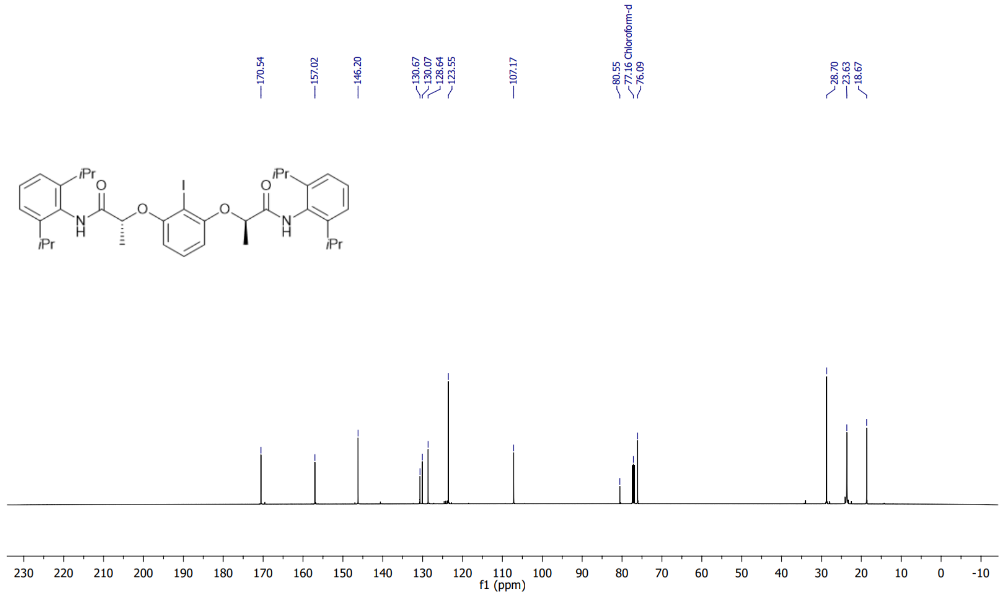

(2*R*,2'*R*)-2,2'-((2-Iodo-1,3-phenylene)bis(oxy))bis(*N*-((*R*)-1-phenylethyl)propanamide) (S12)

^{1}H NMR (300 MHz, $CDCl_3$)

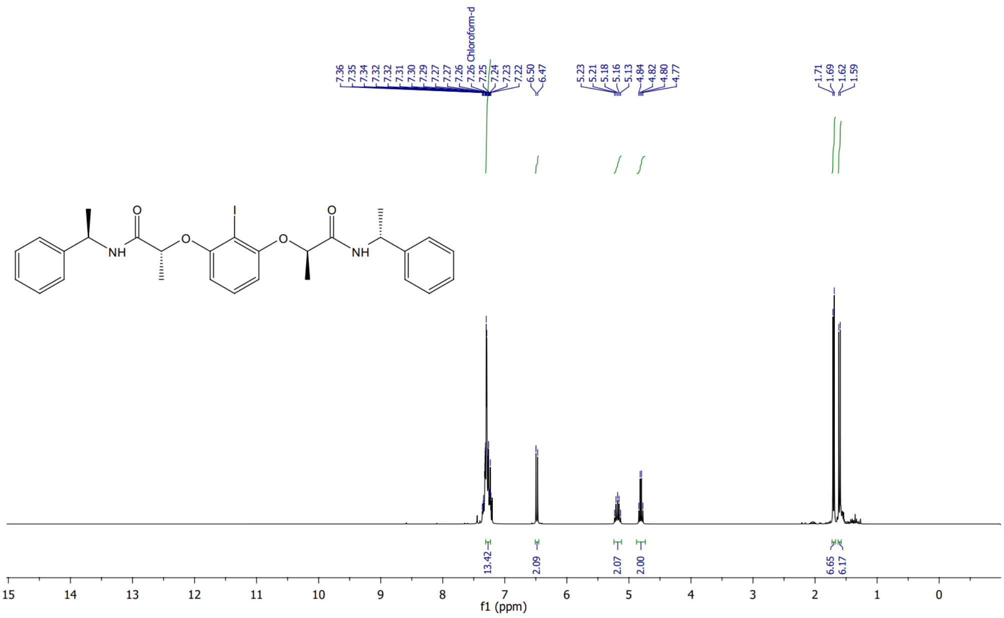

^{13}C NMR (75 MHz, $CDCl_3$)

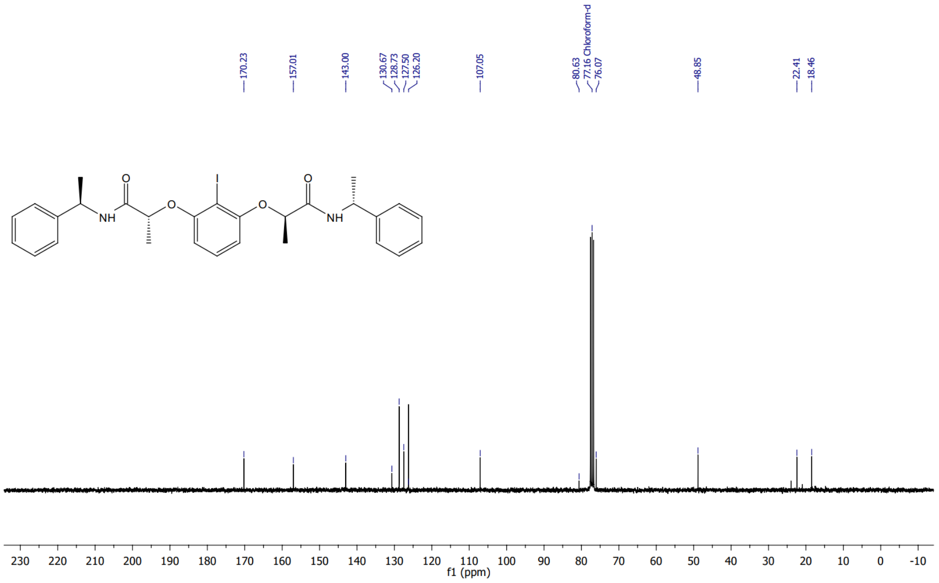

(2*R*,2'*R*)-2,2'-((2-Iodo-1,3-phenylene)bis(oxy))bis(*N*-((*S*)-1-phenylethyl)propanamide) (S13)

^{1}H NMR (75 MHz, CDCl$_3$)

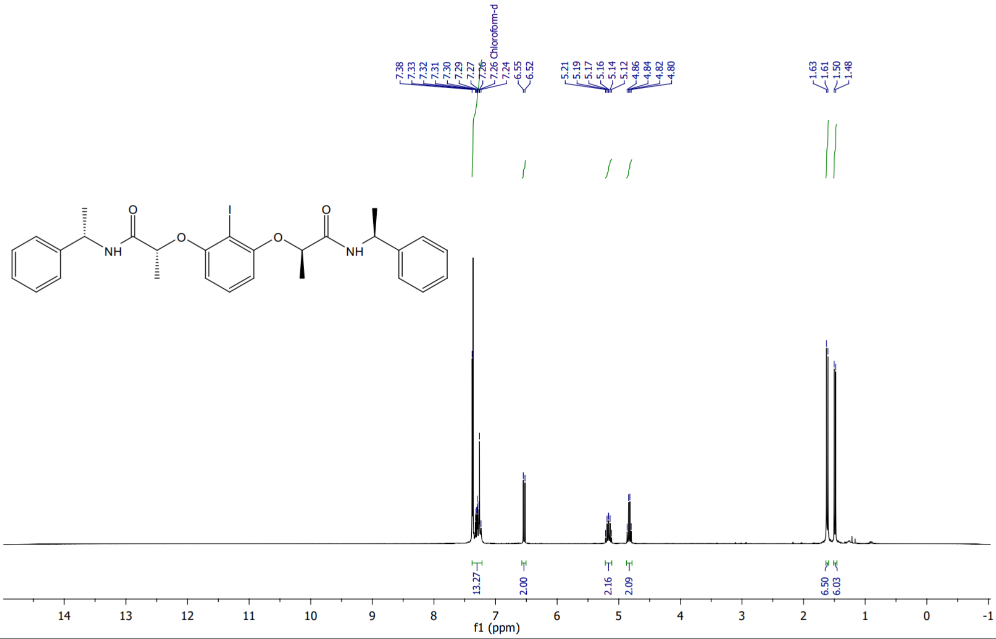

^{13}C NMR (75 MHz, CDCl$_3$)

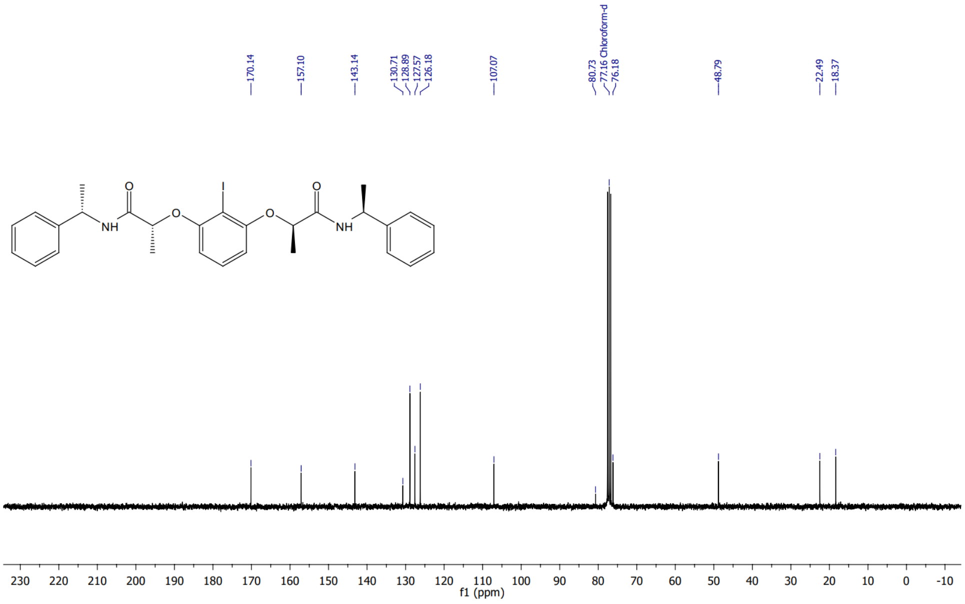

1-Methoxy-2-methyl-4-(prop-1-yn-1-yl)benzene (S14a)

^{1}H NMR (300 MHz, $CDCl_3$)

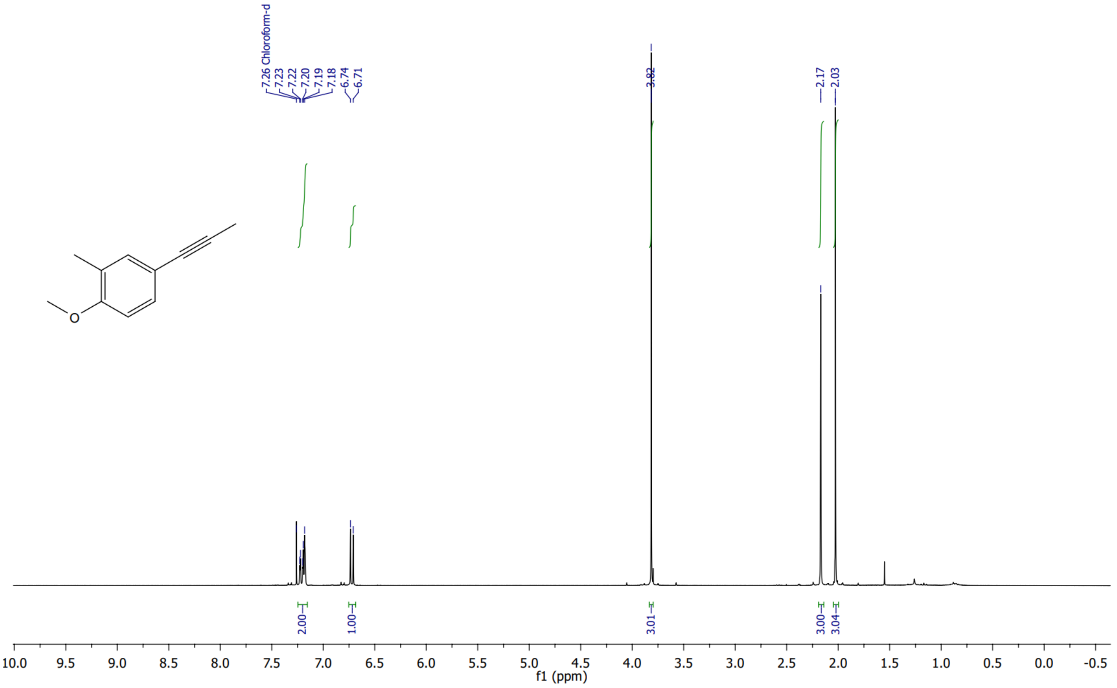

^{13}C NMR (75 MHz, $CDCl_3$)

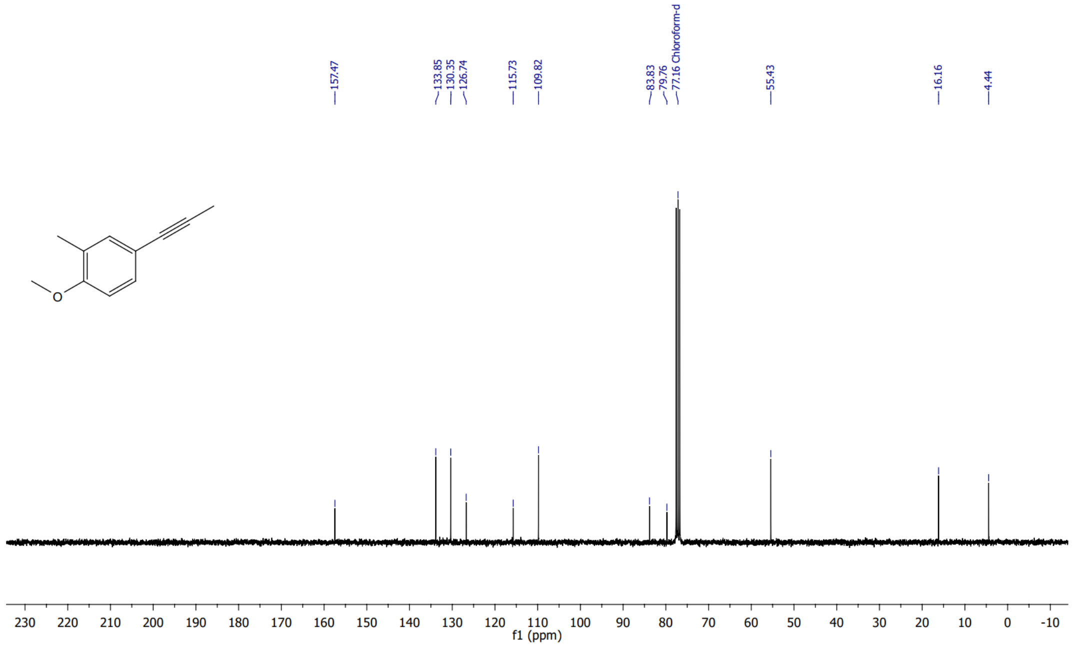

1-Methoxy-2-(prop-1-yn-1-yl)benzene (S14b)

^{1}H NMR (300 MHz, $CDCl_3$)

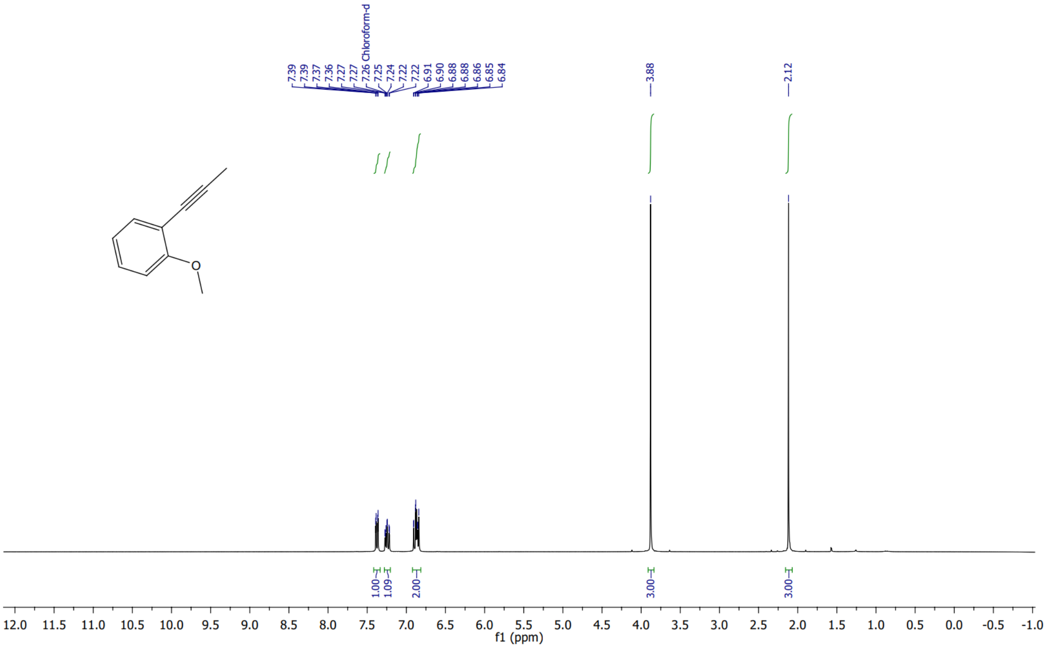

^{13}C NMR (75 MHz, $CDCl_3$)

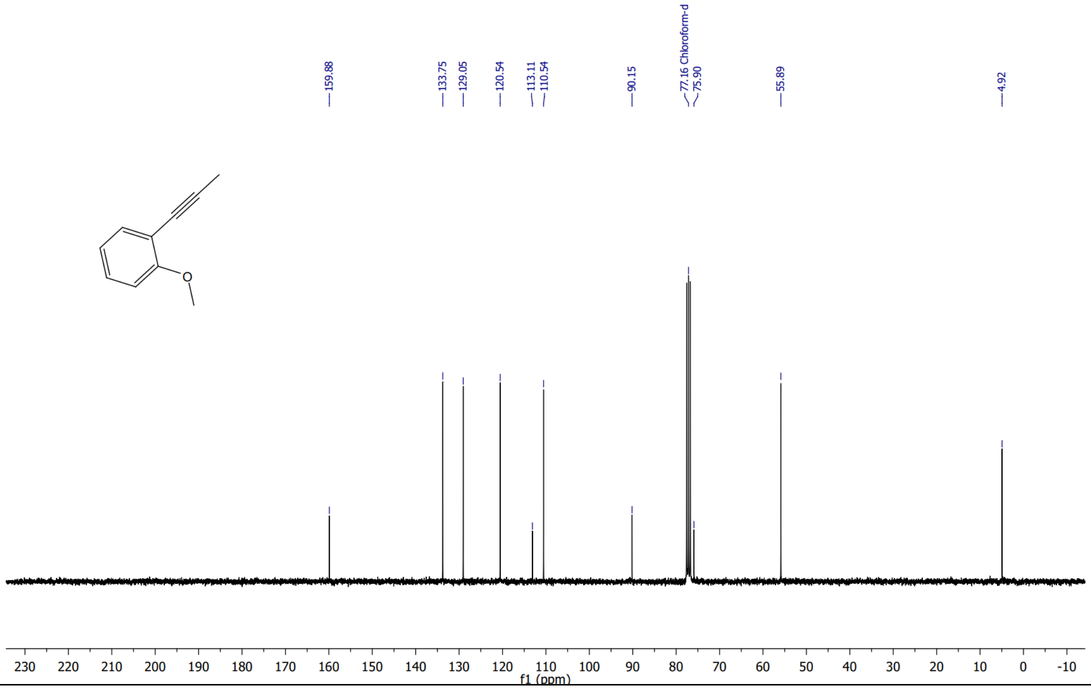

1-(But-1-yn-1-yl)-4-methoxybenzene (S14c)

^{1}H NMR (400 MHz, $CDCl_3$)

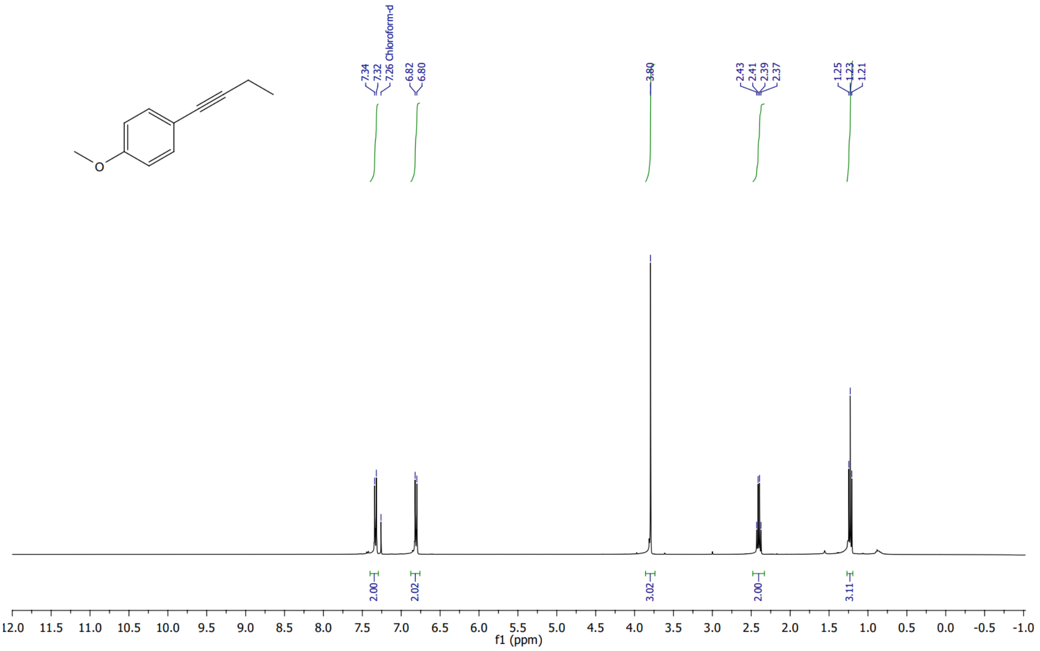

^{13}C NMR (101 MHz, $CDCl_3$)

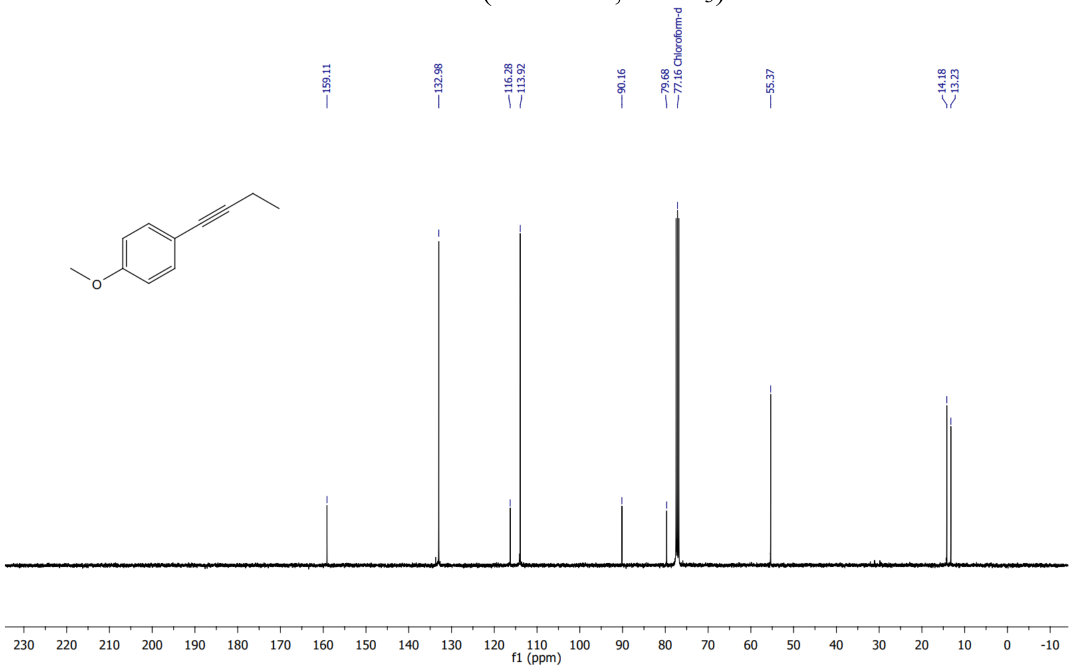

1,3-Dimethoxy-2-methyl-4-(prop-1-yn-1-yl)benzene (S14d)

^{1}H NMR (500 MHz, $CDCl_3$)

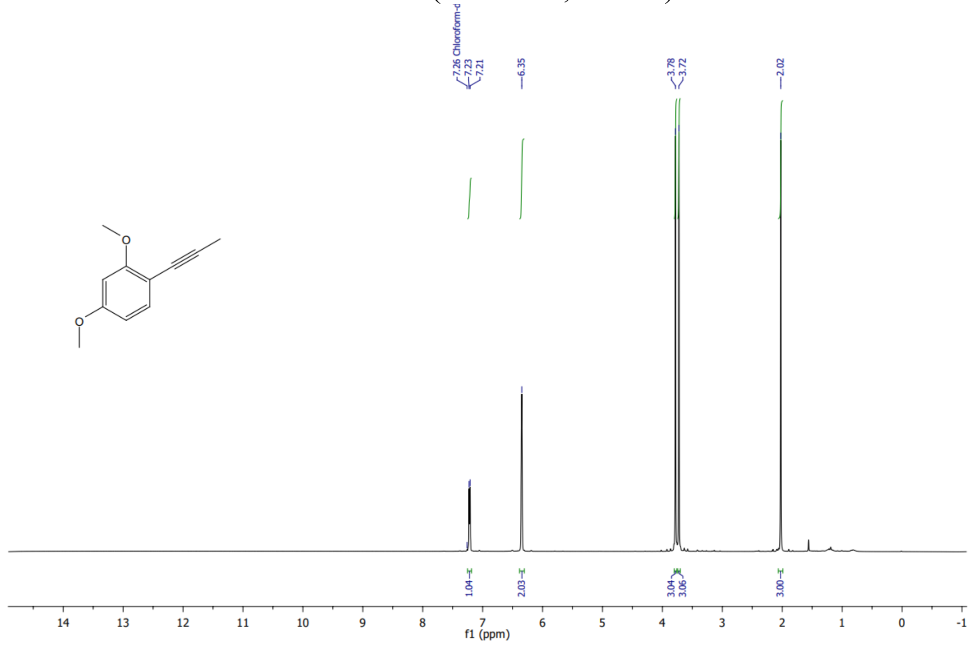

^{13}C NMR (126 MHz, $CDCl_3$)

^{13}C NMR (126 MHz, $CDCl_3$) δ 161.01, 160.58, 134.29, 105.69, 104.70, 98.42, 88.28, 77.16, 75.66, 55.85, 55.44, 4.80.

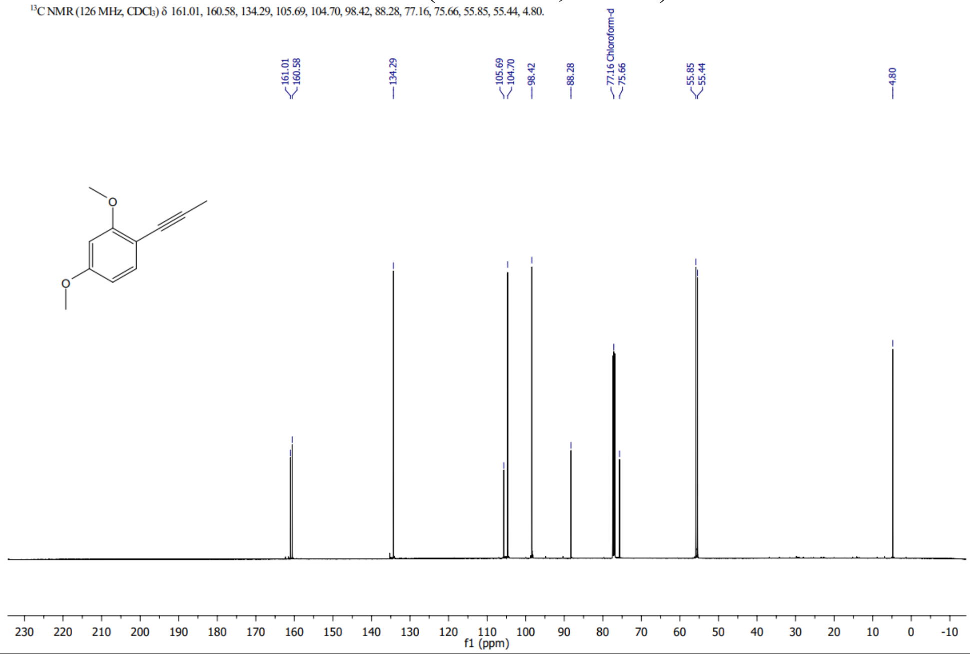

1,3-Dimethoxy-2-methyl-4-(prop-1-yn-1-yl)benzene (S14e)

^{1}H NMR (400 MHz, $CDCl_3$)

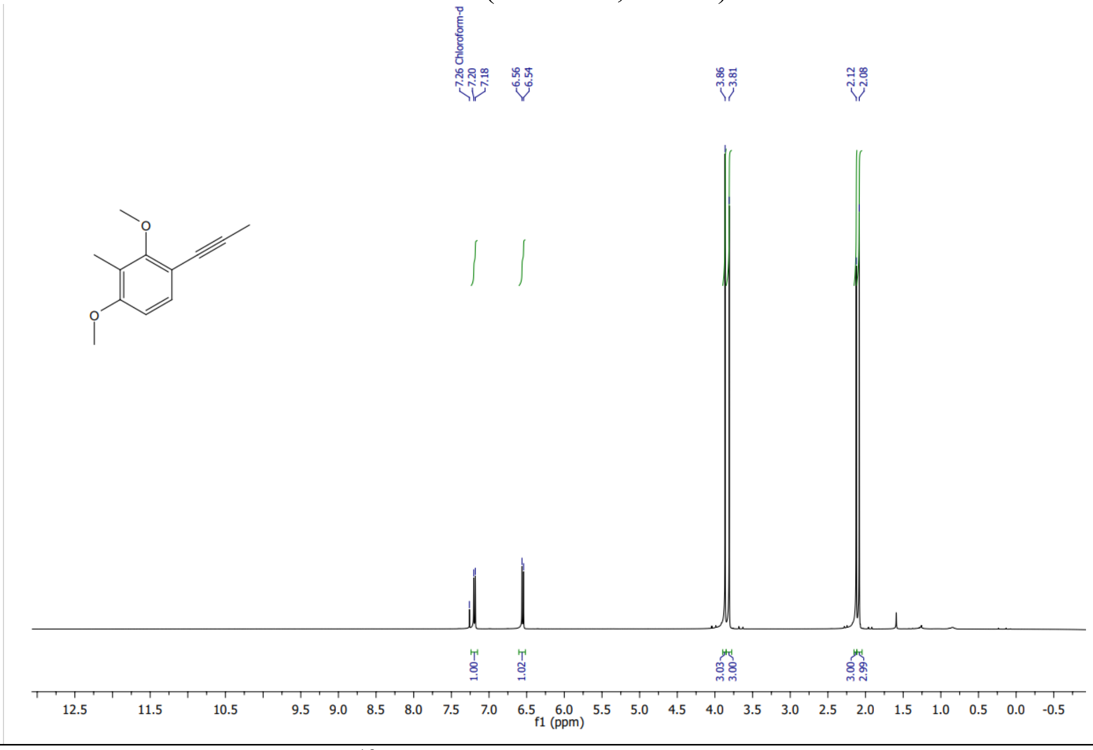

^{13}C NMR (101 MHz, $CDCl_3$)

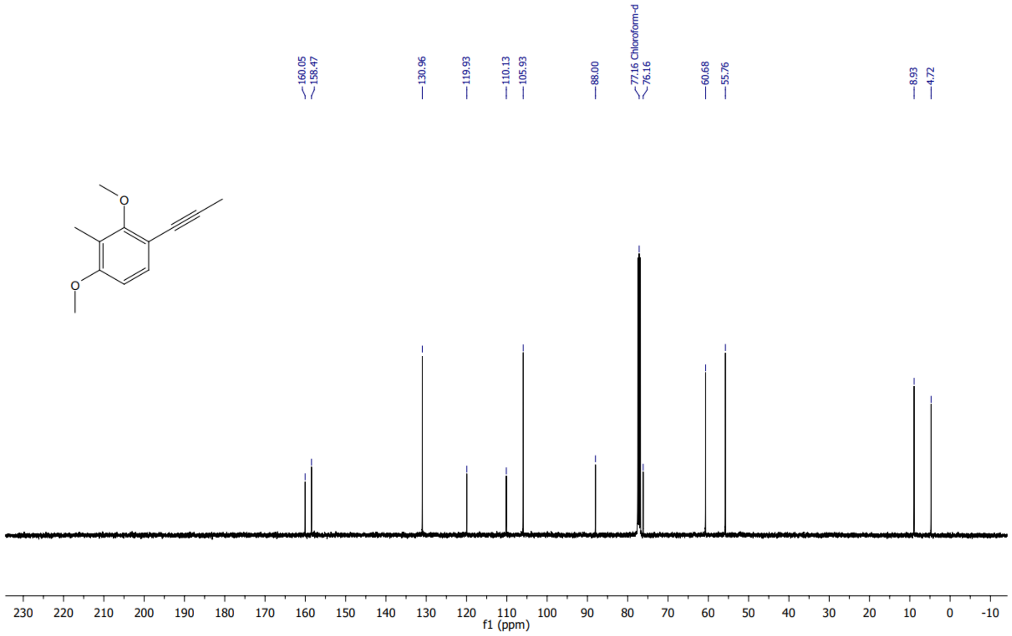

((2,4-Dimethoxyphenyl)ethynyl)trimethylsilane (S15)

^{1}H NMR (400 MHz, $CDCl_3$)

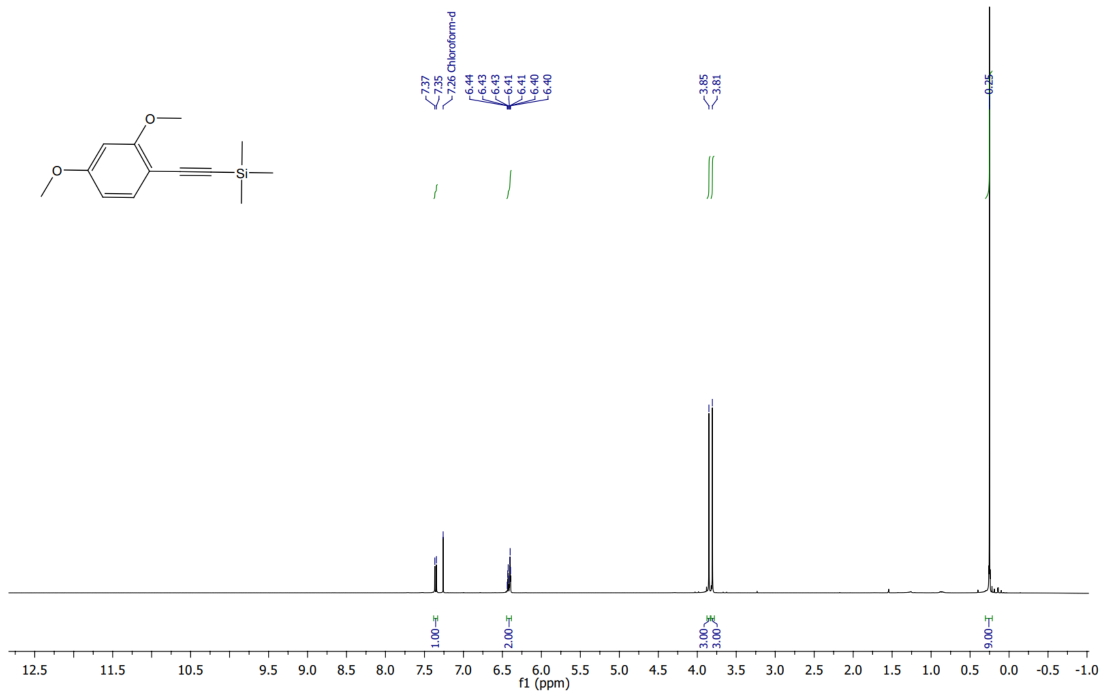

^{13}C NMR (101 MHz, $CDCl_3$)

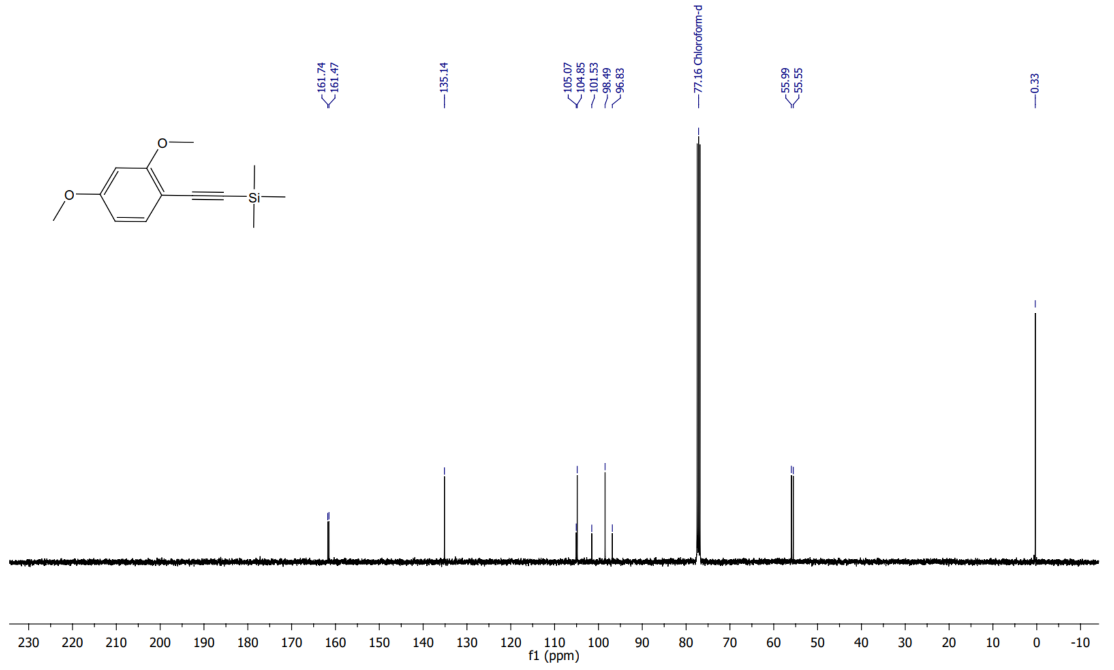

1-Ethynyl-2,4-dimethoxybenzene (S16)

^{1}H NMR (400 MHz, $CDCl_3$)

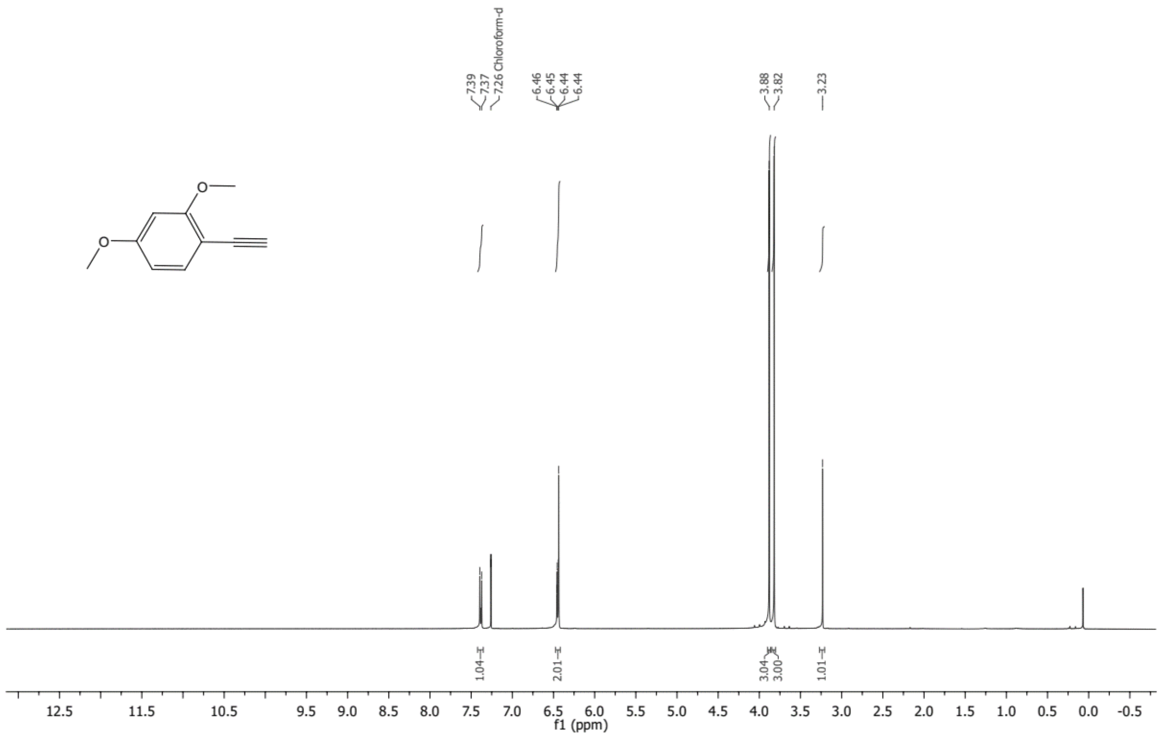

^{13}C NMR (101 MHz, $CDCl_3$)

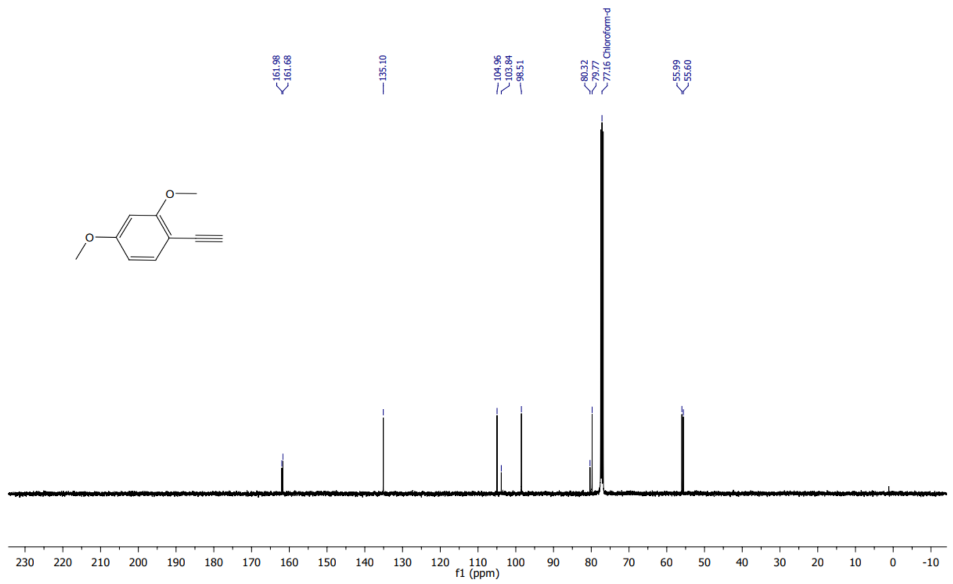

(*S*)-2-(6-Methoxynaphthalen-2-yl)propanoic acid [naproxen, (*S*)-2]

^{1}H NMR (400 MHz, $CDCl_3$)

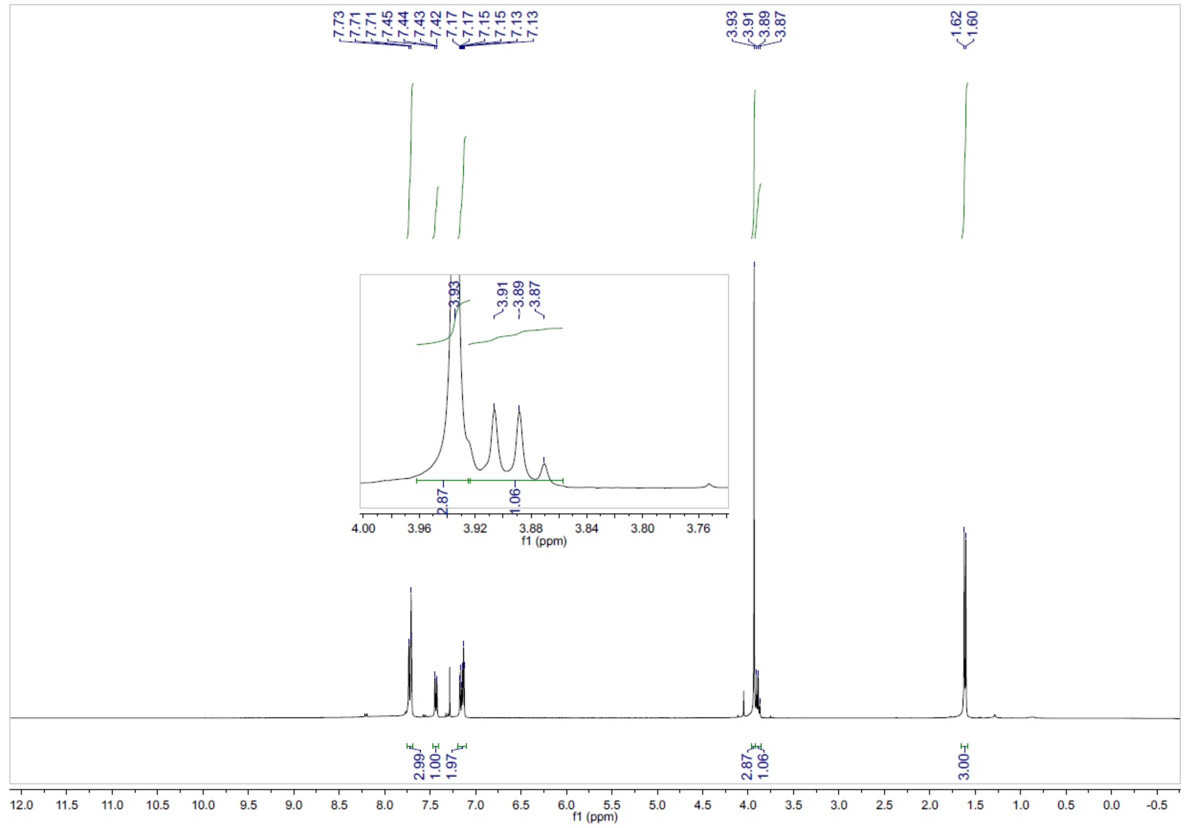

^{13}C NMR (75 MHz, $CDCl_3$)

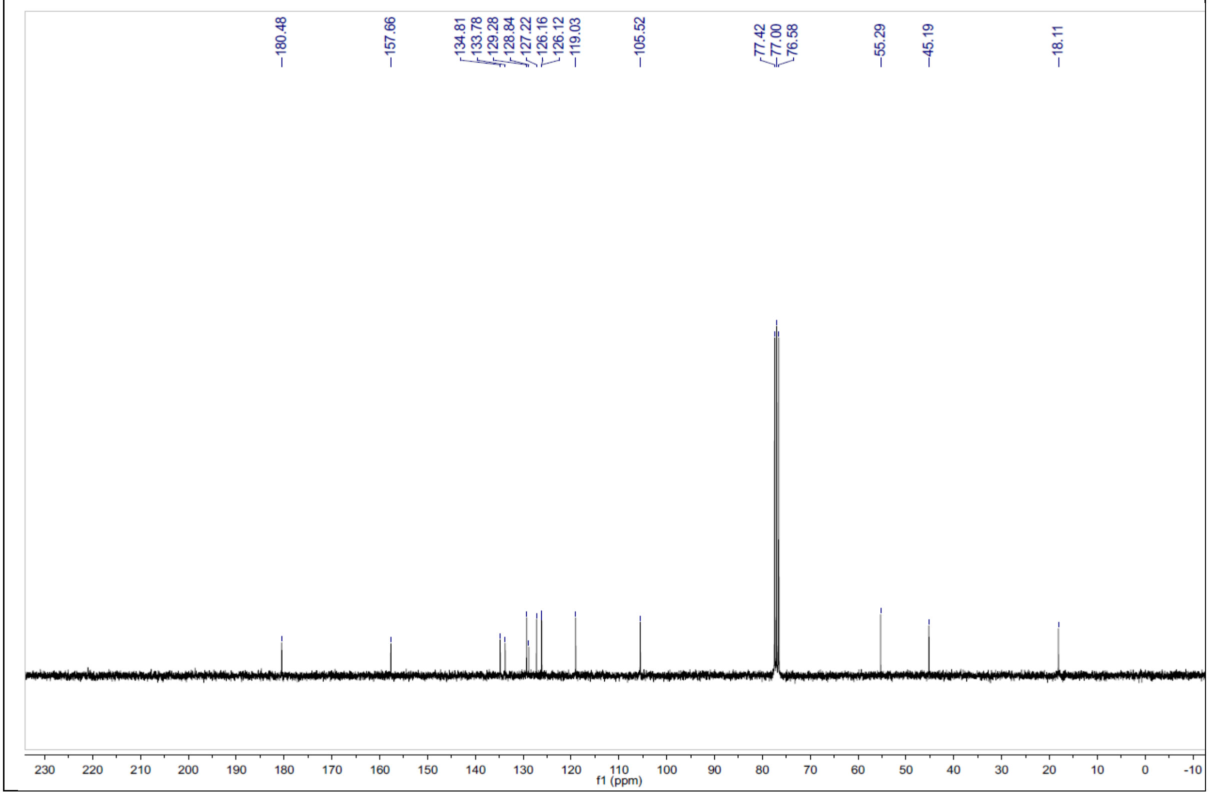

1-Methoxy-4-(prop-1-yn-1-yl)benzene (15)

^{1}H NMR (300 MHz, $CDCl_3$)

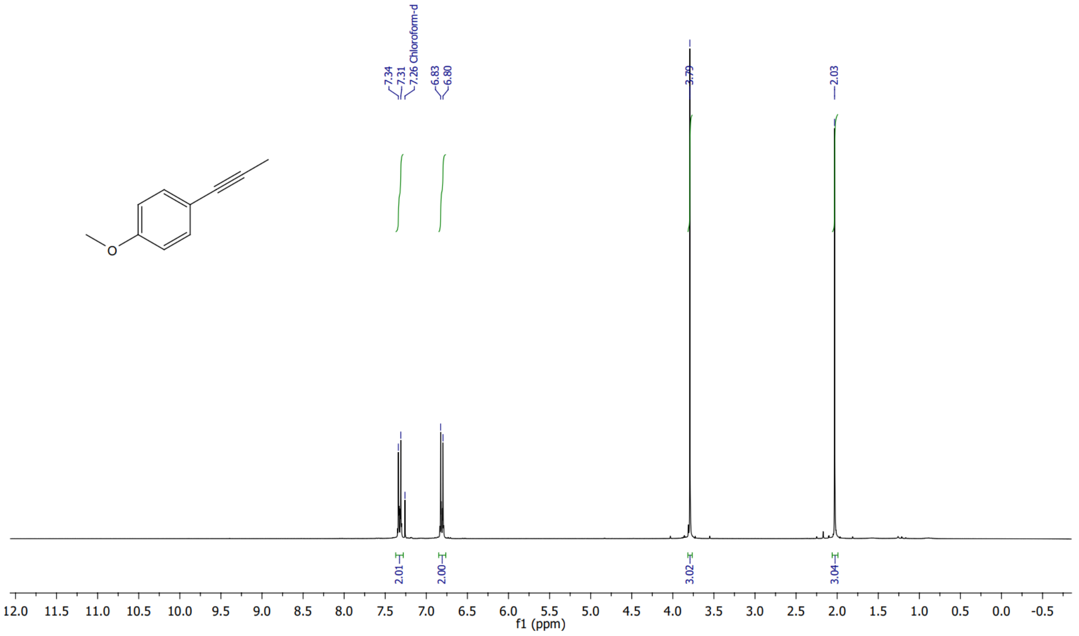

^{13}C NMR (75 MHz, $CDCl_3$)

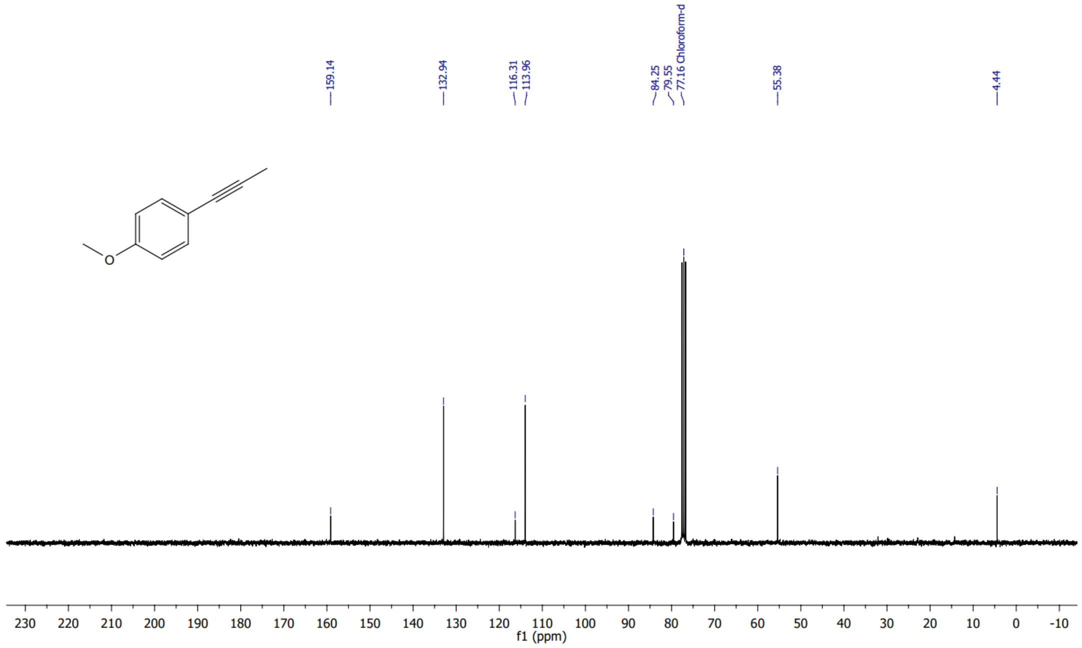

Methyl (*R*)-2-(4-methoxyphenyl)propanoate [(*R*)-16a]

^{1}H NMR (400 MHz, $CDCl_3$)

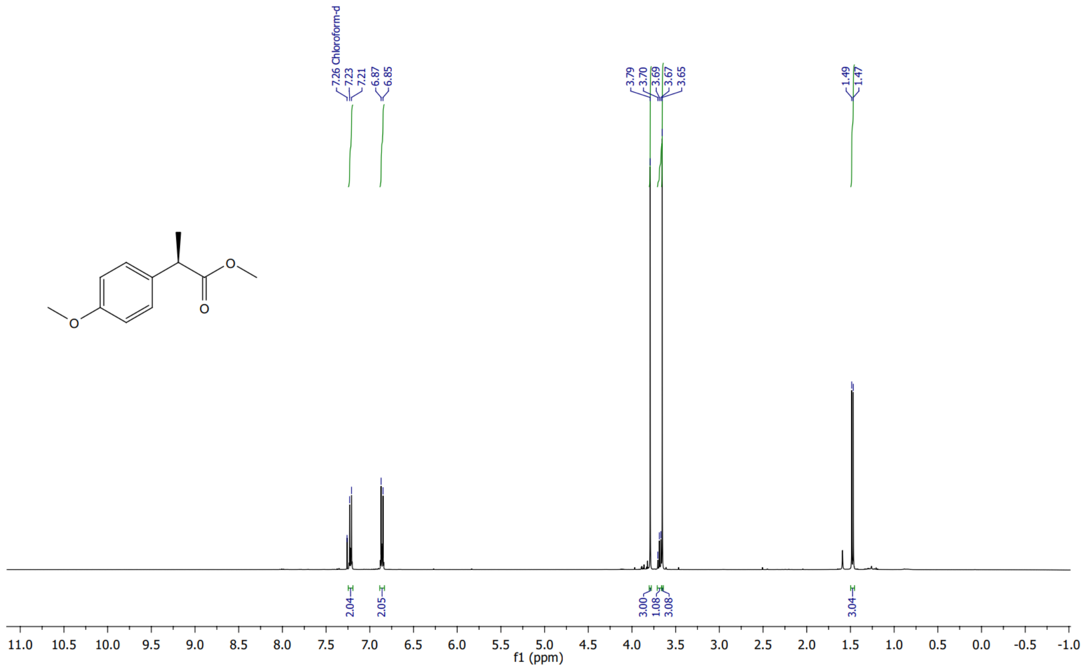

^{13}C NMR (101 MHz, $CDCl_3$)

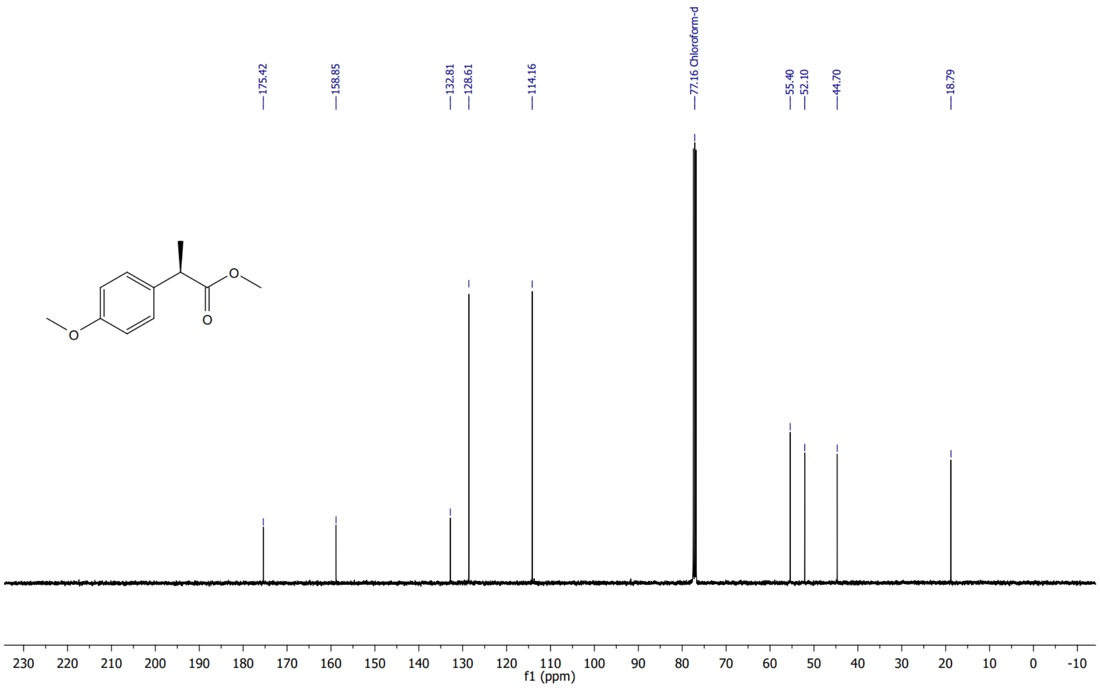

2-Ethoxy-1-(4-methoxyphenyl)propan-1-one (16b)

^{1}H NMR (500 MHz, $CDCl_3$)

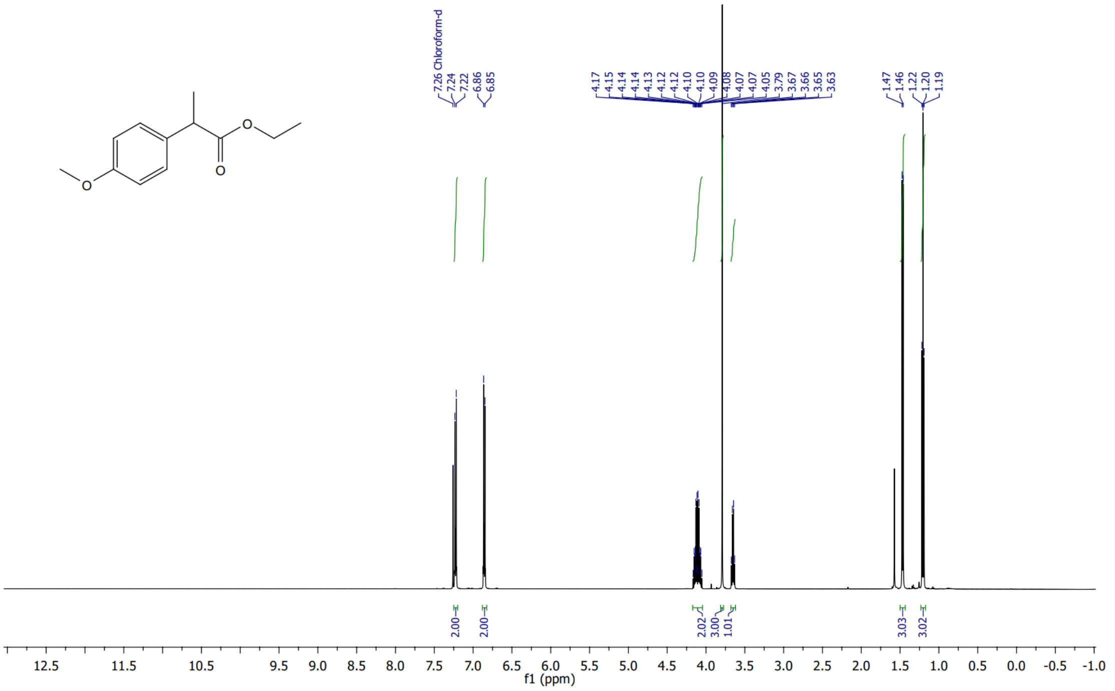

^{13}C NMR (126 MHz, $CDCl_3$)

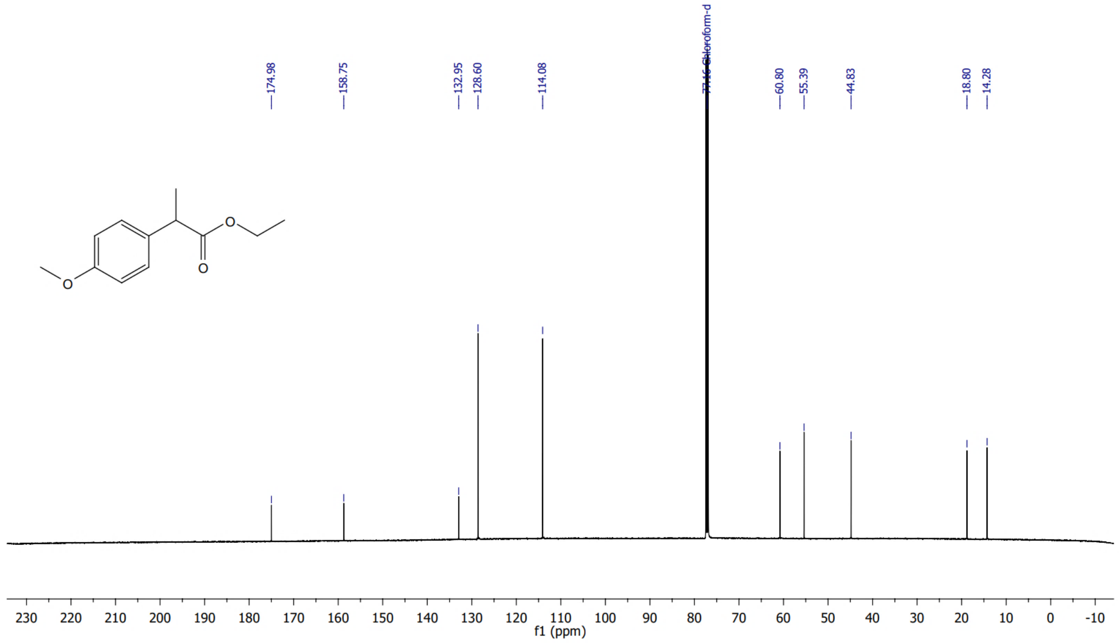

Isopropyl (*R*)-2-(4-methoxyphenyl)propanoate [(*R*)-16c]

^{1}H NMR (500 MHz, $CDCl_3$)

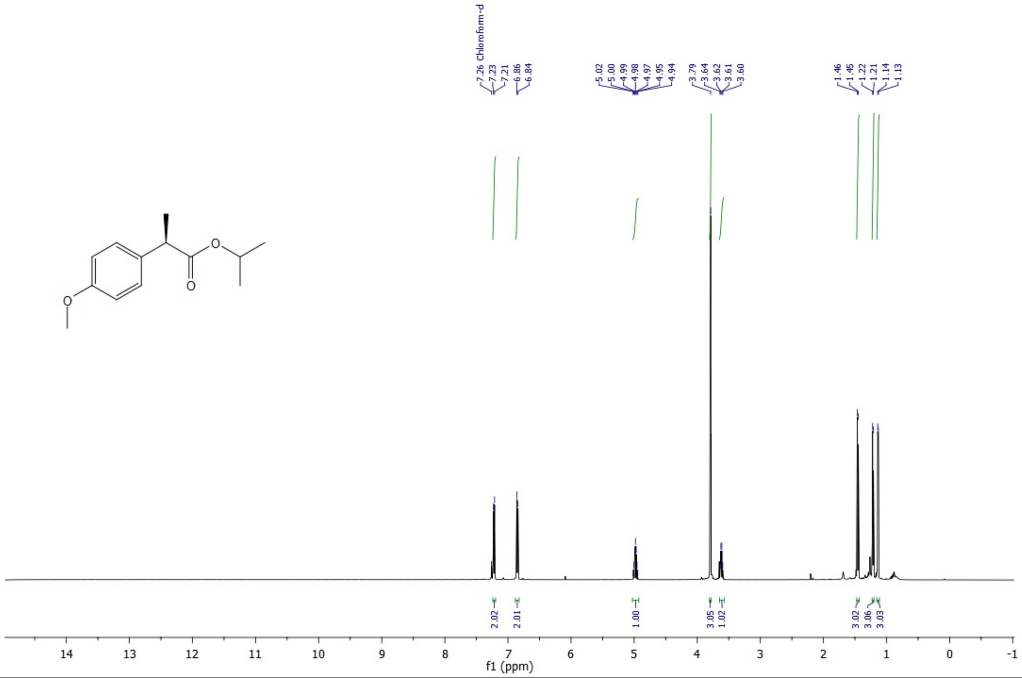

^{13}C NMR (126 MHz, $CDCl_3$)

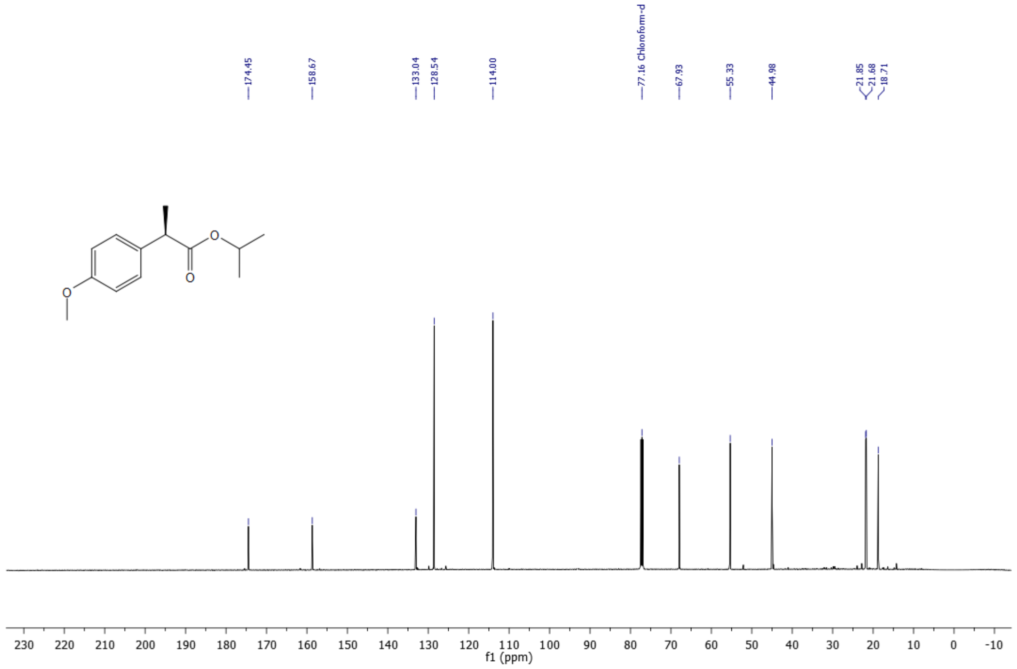

Propyl 2-(4-methoxyphenyl)propanoate (16d)

^{1}H NMR (300 MHz, CDCl$_3$)

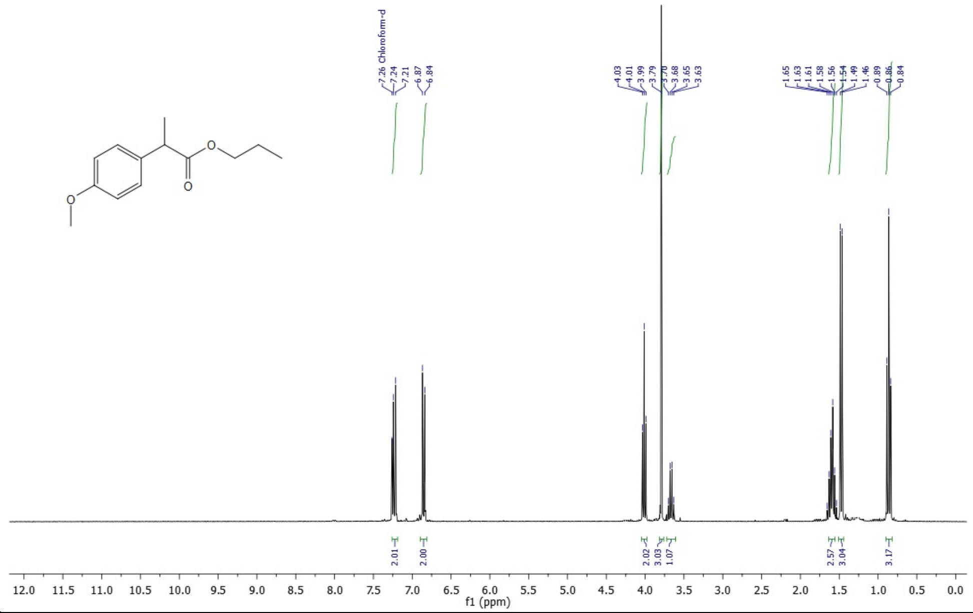

^{13}C NMR (75 MHz, CDCl$_3$)

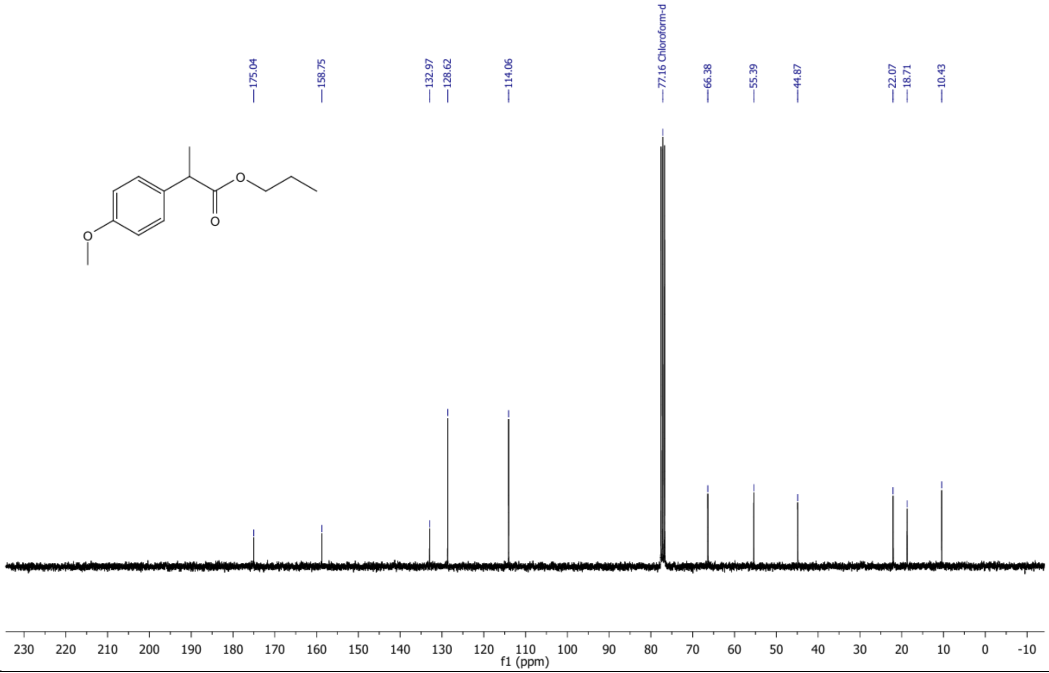

Dimethyl 2,2'-((2-(diacetoxy-λ[3]-iodanyl)-1,3-phenylene)bis(oxy))(2*R*,2'*R*)-dipropionate (19a)

^{1}H NMR (300 MHz, $CDCl_3$)

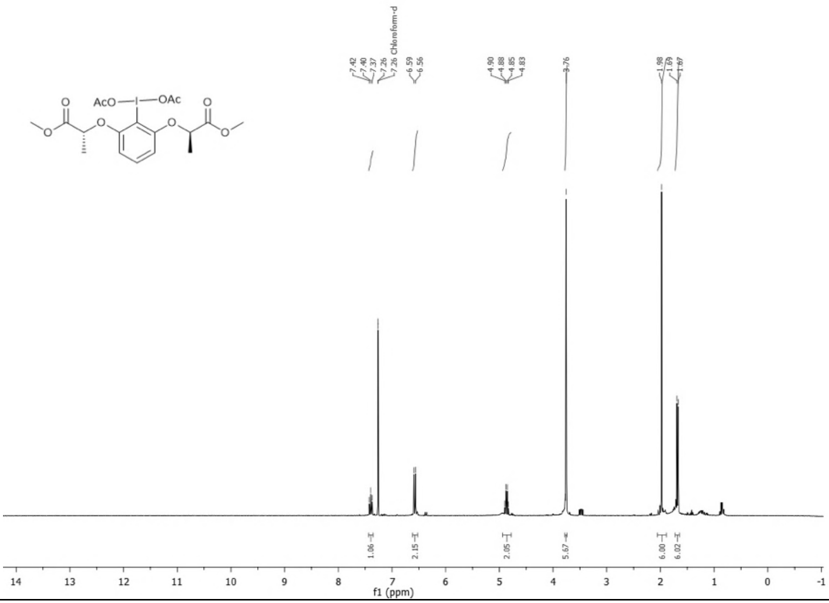

^{13}C NMR (75 MHz, $CDCl_3$)

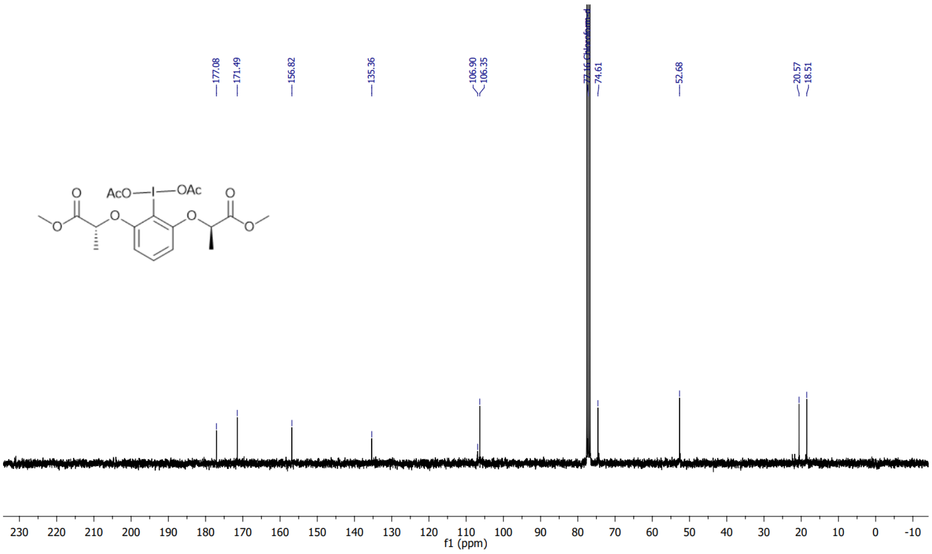

Diethyl 2,2'-((2-(diacetoxy-λ^3-iodaneyl)-1,3-phenylene)bis(oxy))(2R,2'R)-dipropionate (19b)

^{1}H NMR (500 MHz, CDCl$_3$)

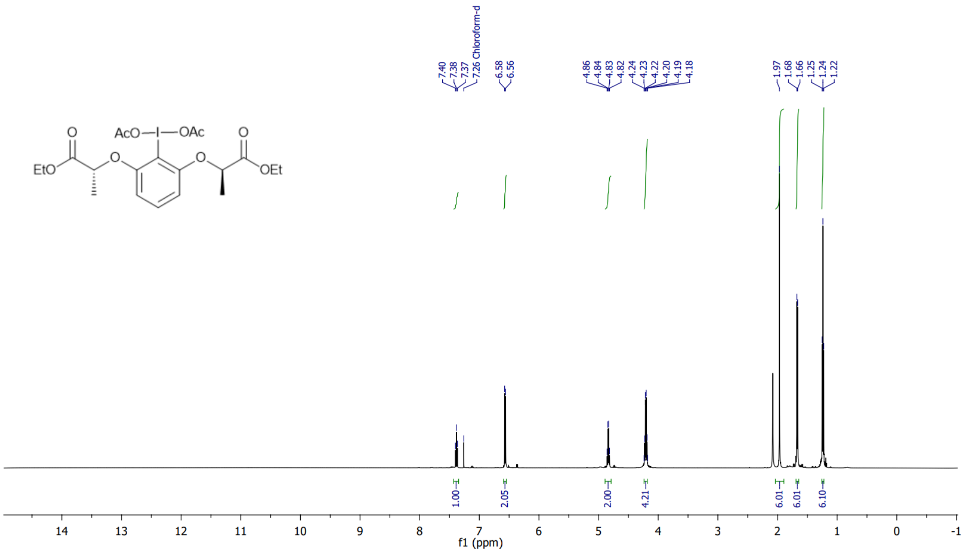

^{13}C NMR (126 MHz, CDCl$_3$)

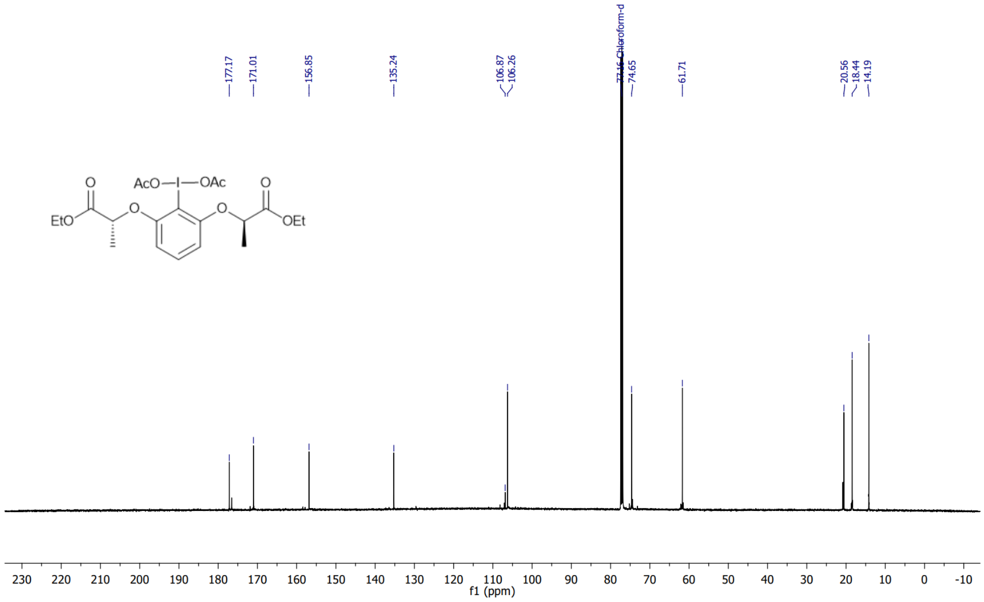

Diisopropyl 2,2'-((2-(diacetoxy-λ^3-iodaneyl)-1,3-phenylene)bis(oxy))(2*R*,2'*R*)-dipropionate (19c)

^{1}H NMR (500 MHz, $CDCl_3$)

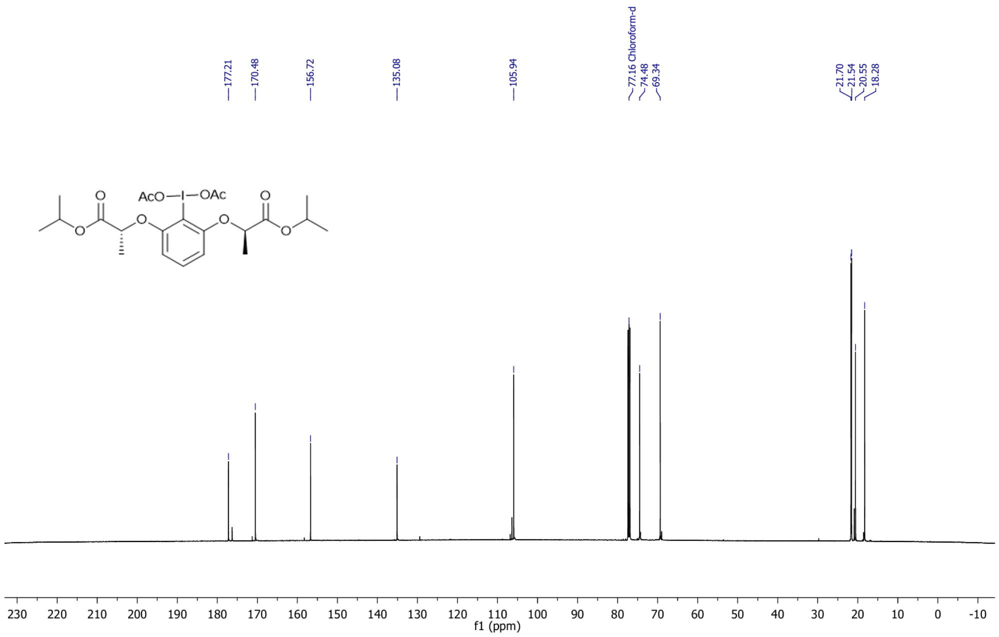

^{13}C NMR (126 MHz, $CDCl_3$)

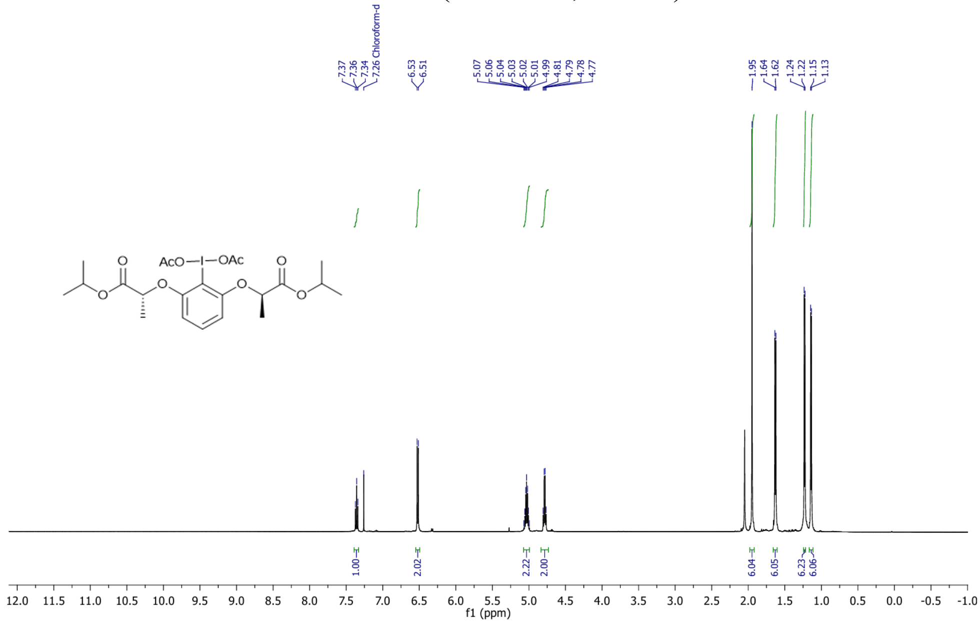

Bis((1*S*,2*R*,5*S*)-2-isopropyl-5-methylcyclohexyl) 2,2'-((2-(diacetoxy-λ^3-iodaneyl)-1,3-phenylene)bis(oxy))(2*R*,2'*R*)-dipropionate (19d)

^{1}H NMR (300 MHz, $CDCl_3$)

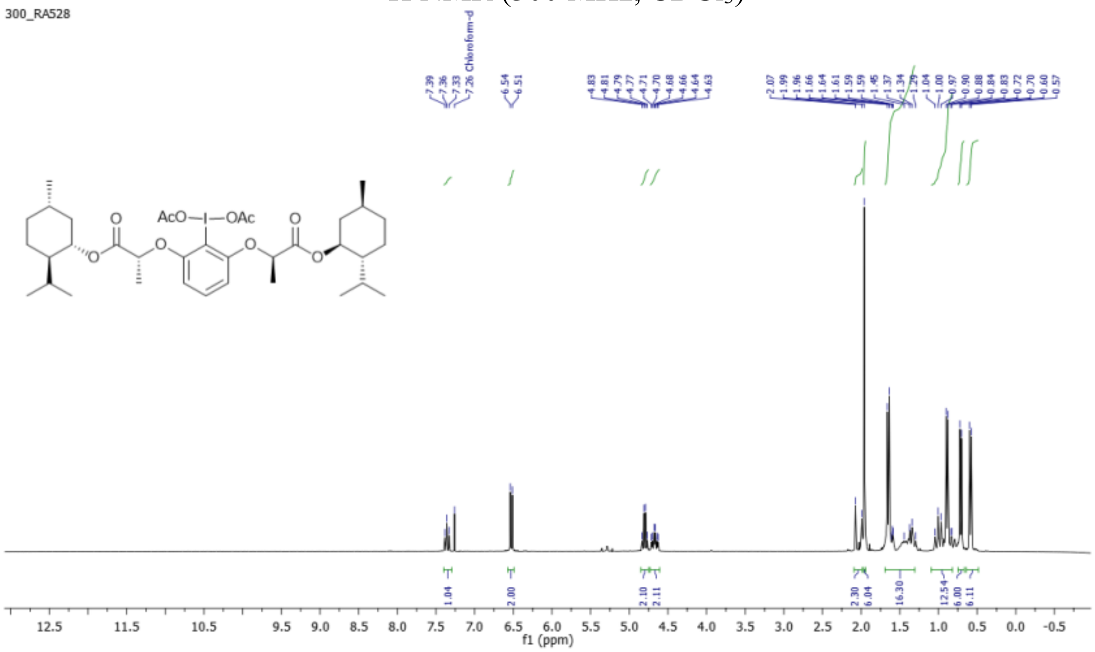

^{13}C NMR (75 MHz, $CDCl_3$)

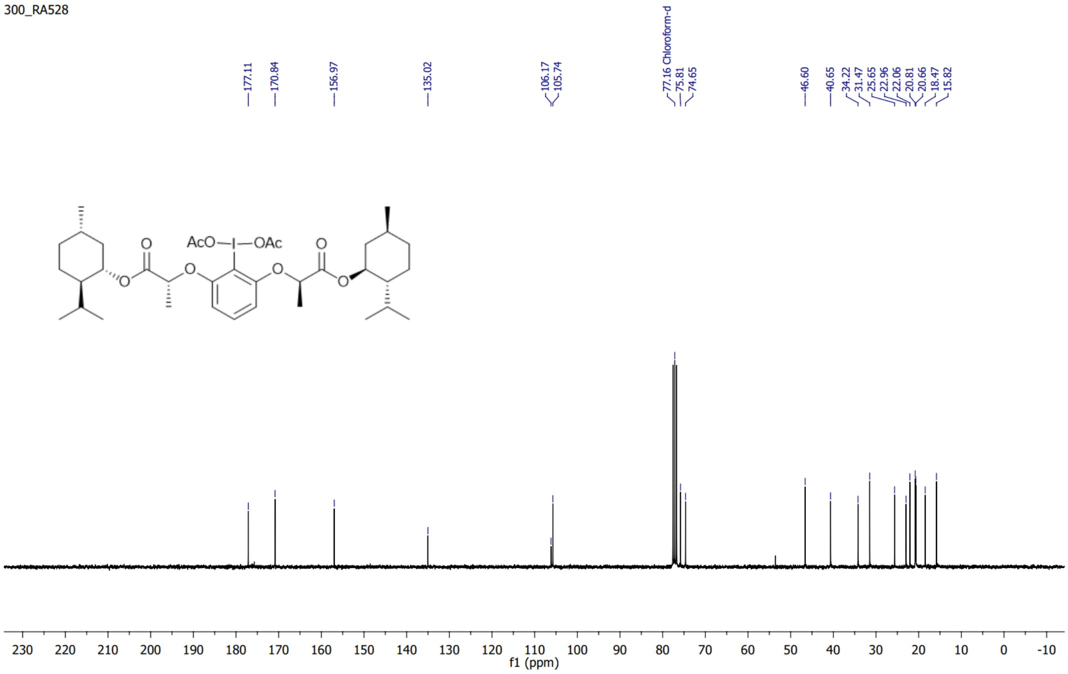

(2,6-Bis(((*R*)-1-((4-methylphenyl)sulfonamido)-1-oxopropan-2-yl)oxy)phenyl)-λ³-iodanediyl diacetate (19e)

^{1}H NMR (400 MHz, $CDCl_3$)

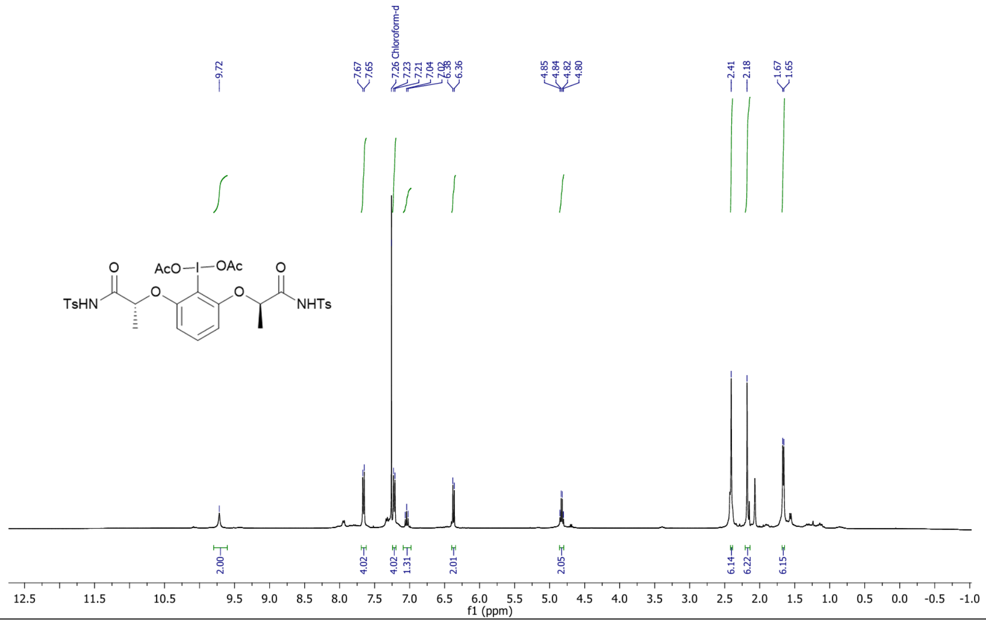

^{13}C NMR (101 MHz, $CDCl_3$)

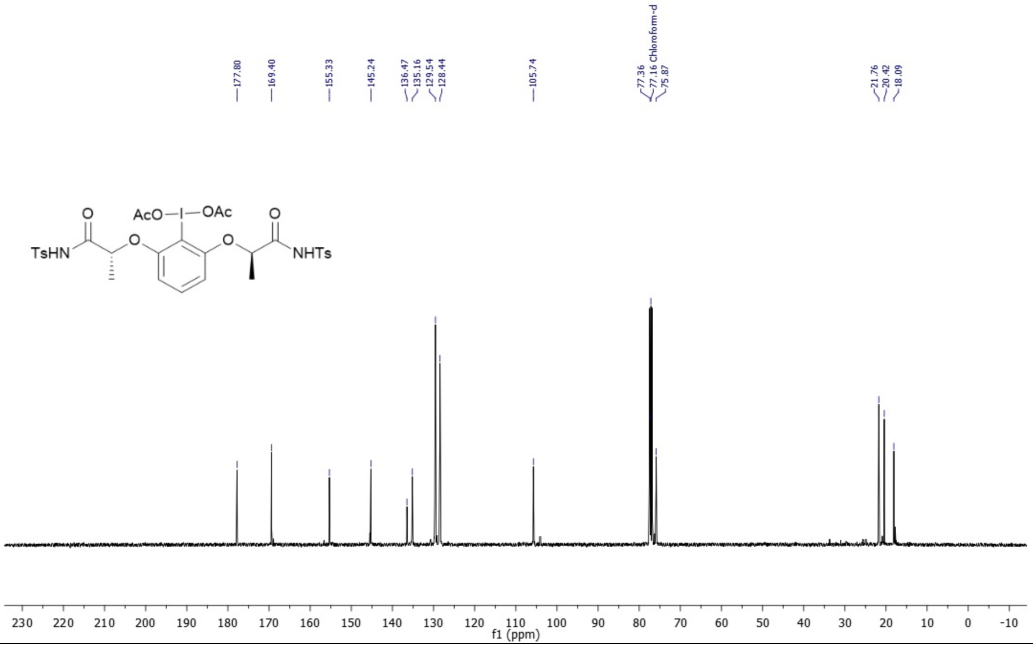

(2,6-Bis(((*R*)-1-oxo-1-((perfluorophenyl)amino)propan-2-yl)oxy)phenyl)-λ^3-iodanediyl diacetate (19f)

^{1}H NMR (400 MHz, $CDCl_3$)

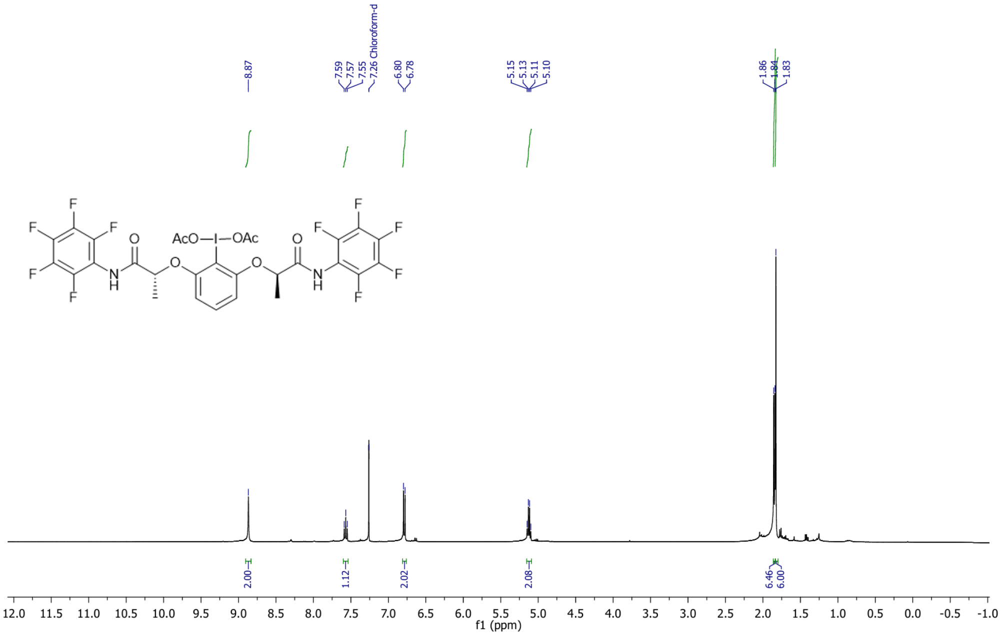

^{13}C NMR (126 MHz, $CDCl_3$)

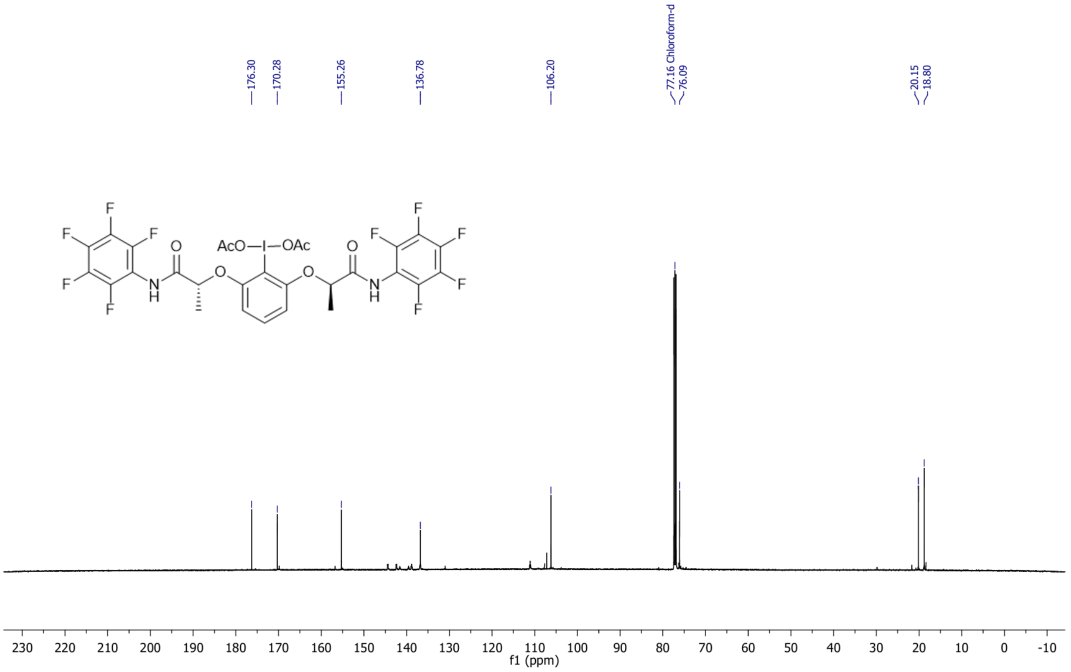

(2,6-Bis(((*R*)-1-((3,5-bis(trifluoromethyl)phenyl)amino)-1-oxopropan-2-yl)oxy)phenyl)-λ^3-iodanediyl diacetate (19g)

^{1}H NMR (500 MHz, $CDCl_3$)

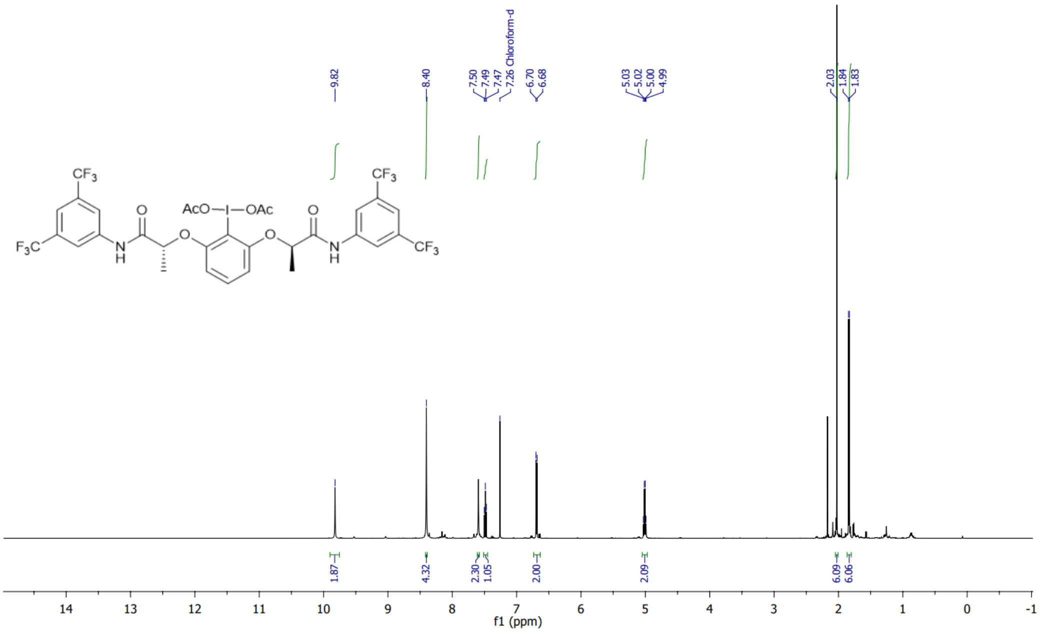

^{13}C NMR (126 MHz, $CDCl_3$)

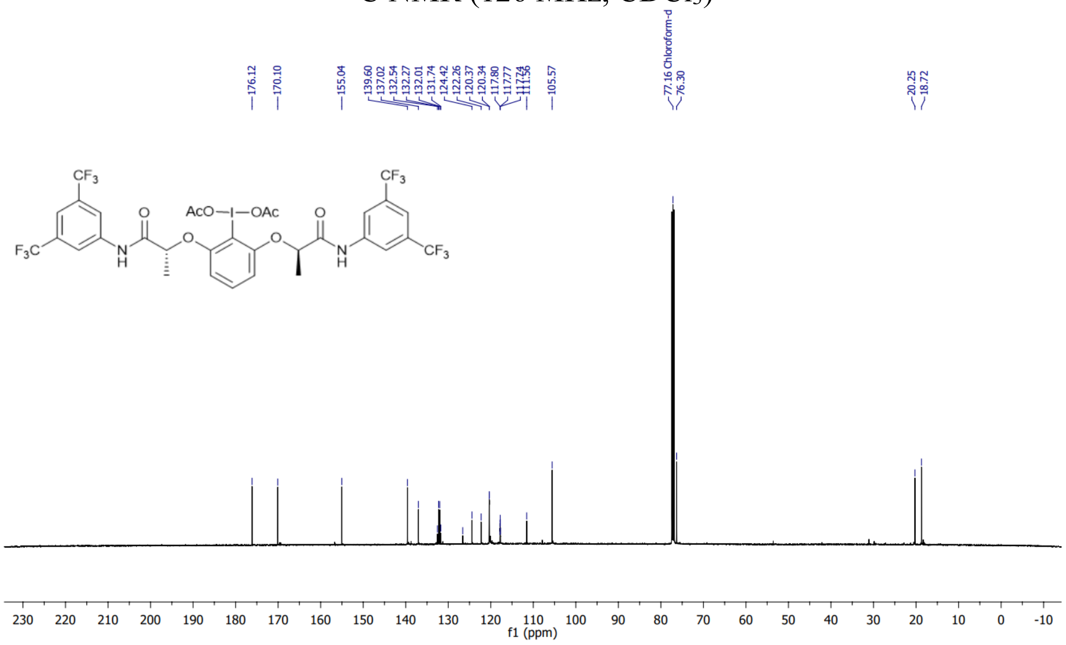

(2,6-Bis(((*R*)-1-((2,6-diisopropylphenyl)amino)-1-oxopropan-2-yl)oxy)phenyl)-λ³-iodanediyl diacetate (19h)

^{1}H NMR (300 MHz, $CDCl_3$)

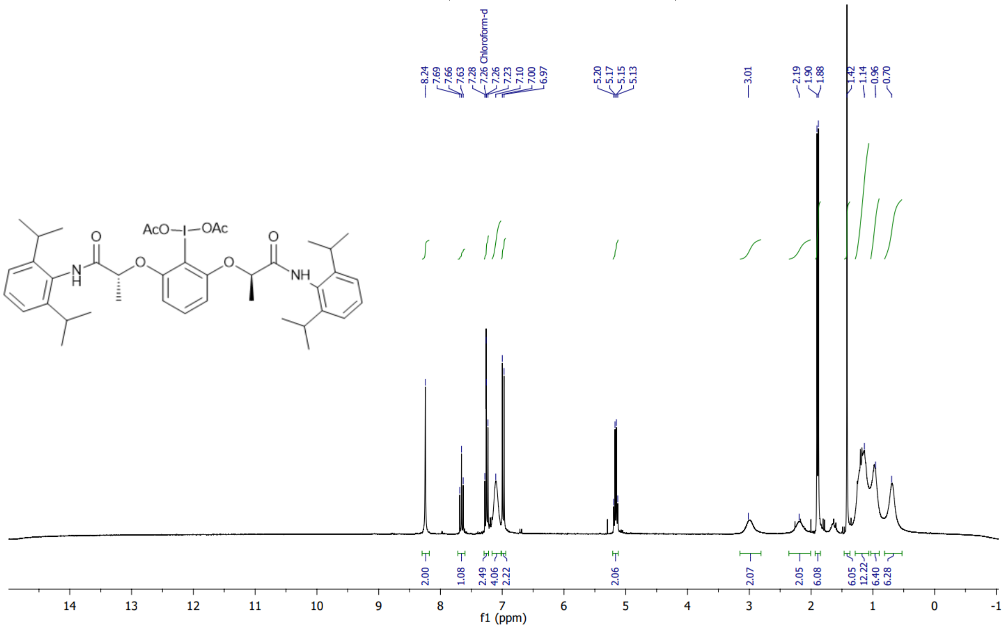

^{13}C NMR (75 MHz, $CDCl_3$)

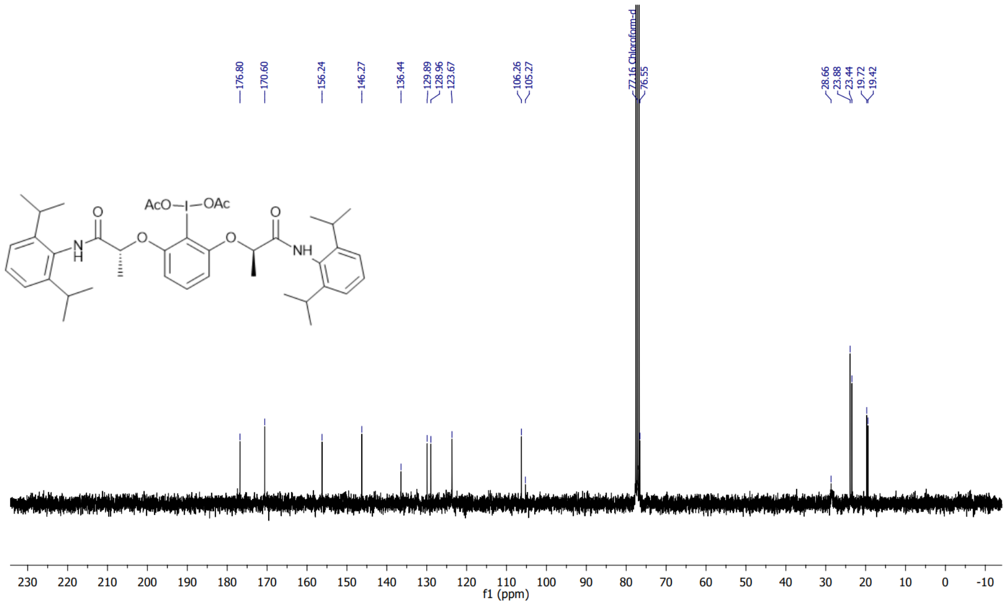

(2,6-Bis(((*R*)-1-oxo-1-(((*S*)-1-phenylethyl)amino)propan-2-yl)oxy)phenyl)-λ^3-iodanediyl diacetate (19i)

^{1}H NMR (400 MHz, CDCl$_3$)

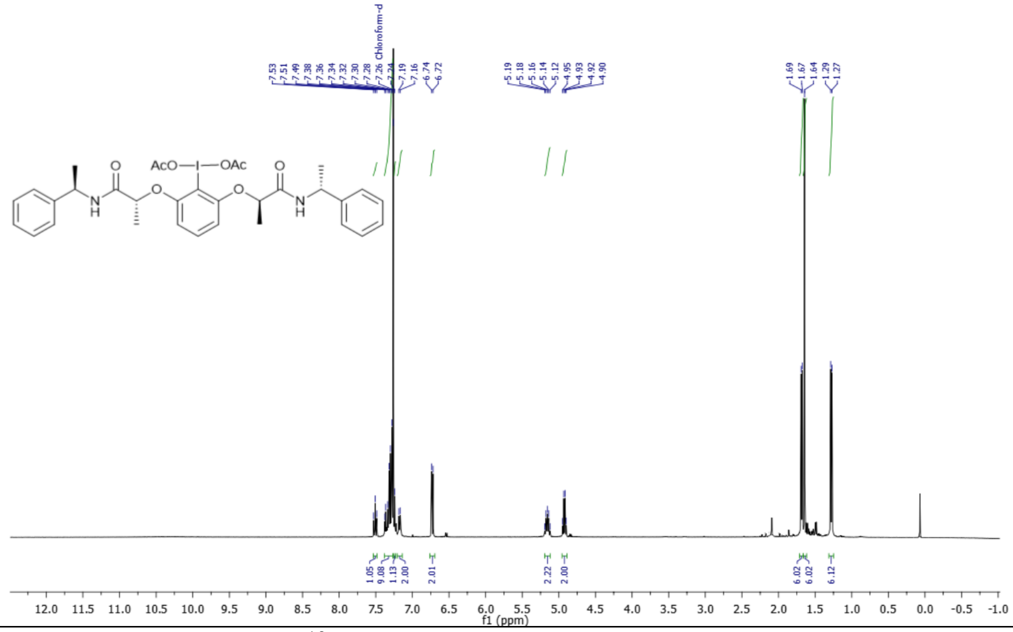

^{13}C NMR (126 MHz, CDCl$_3$)

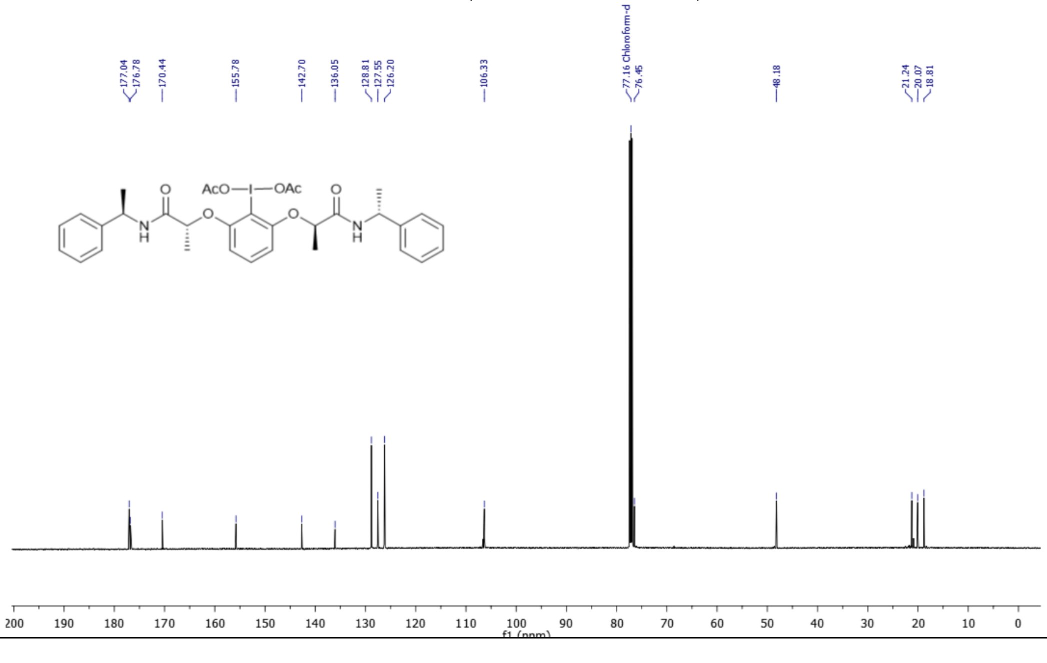

(2,6-Bis(((*R*)-1-oxo-1-(((*S*)-1-phenylethyl)amino)propan-2-yl)oxy)phenyl)-λ³-iodanediyl diacetate (19j)

^{1}H NMR (400 MHz, $CDCl_3$)

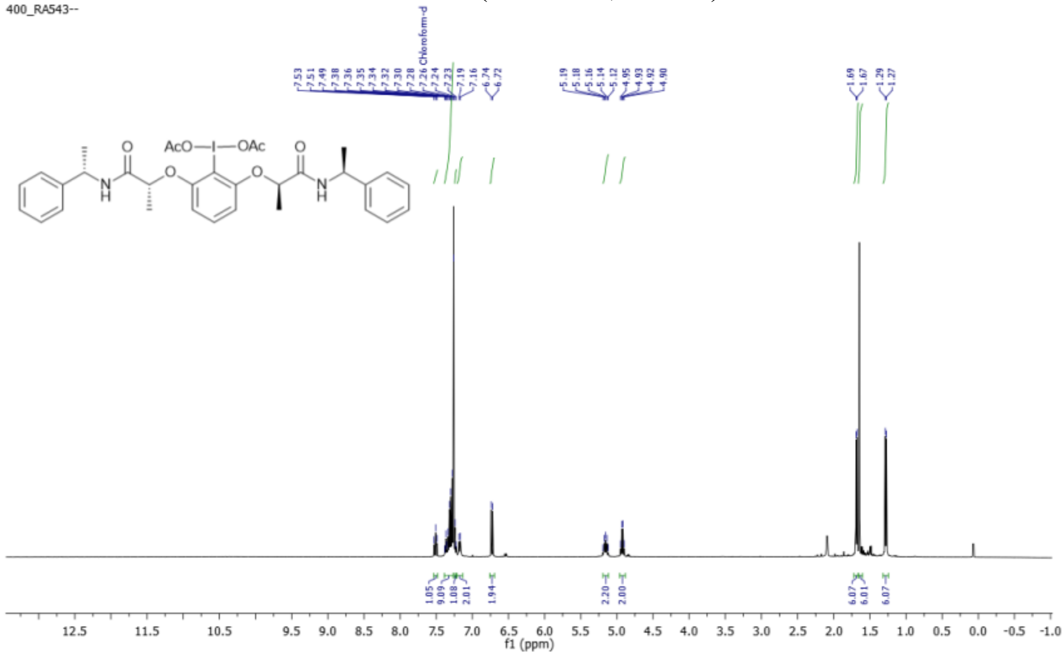

^{13}C NMR (126 MHz, $CDCl_3$)

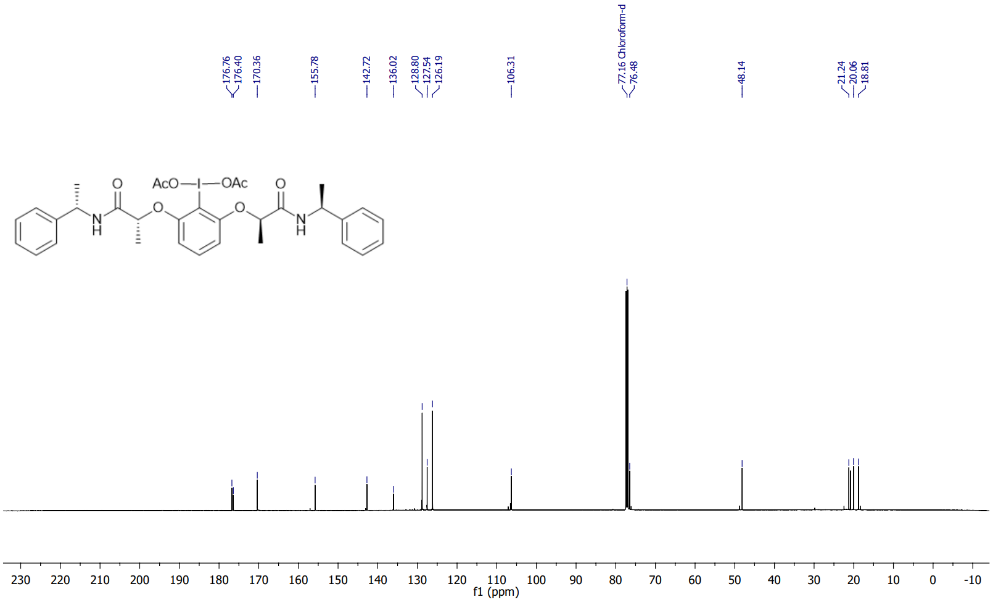

Methyl 2-(4-methoxy-3-methylphenyl)propanoate (20a)

^{1}H NMR (300 MHz, $CDCl_3$)

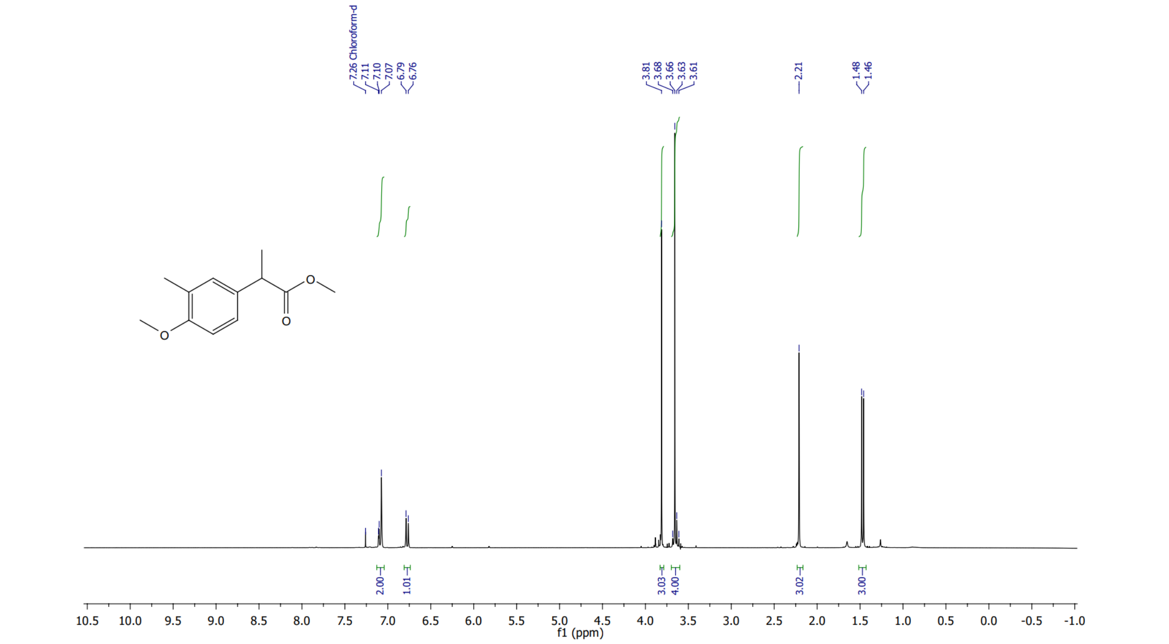

^{13}C NMR (75 MHz, $CDCl_3$)

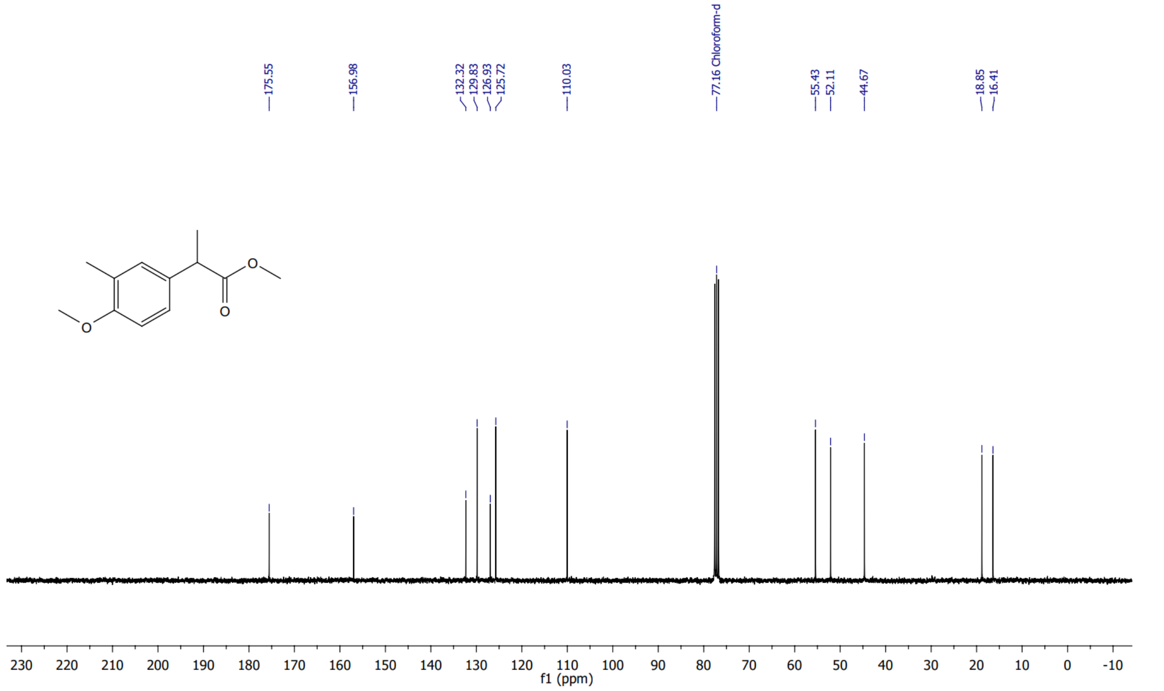

Ethyl 2-(4-methoxy-3-methylphenyl)propanoate (20b)

^{1}H NMR (500 MHz, $CDCl_3$)

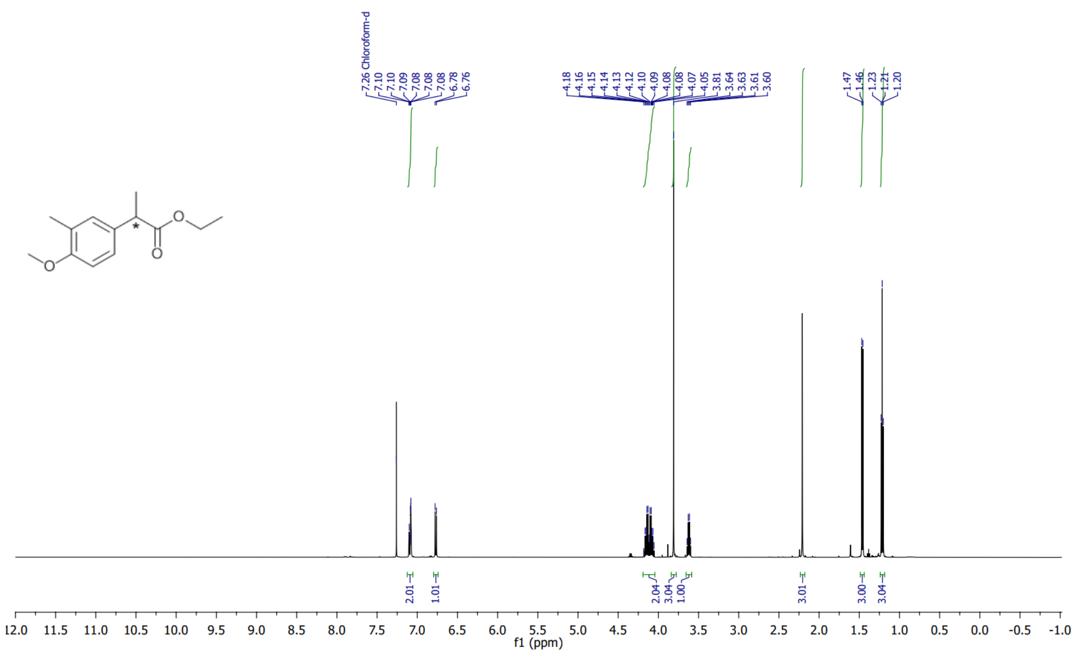

^{13}C NMR (126 MHz, $CDCl_3$)

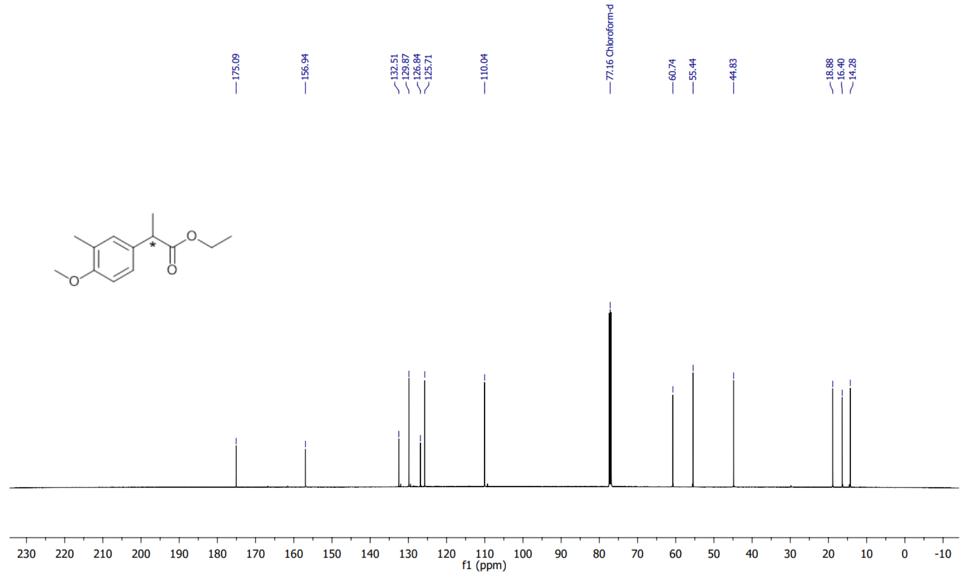

Isopropyl (*R*)-2-(4-methoxy-3-methylphenyl)propanoate [(*R*)-20c]

^{1}H NMR (300 MHz, $CDCl_3$)

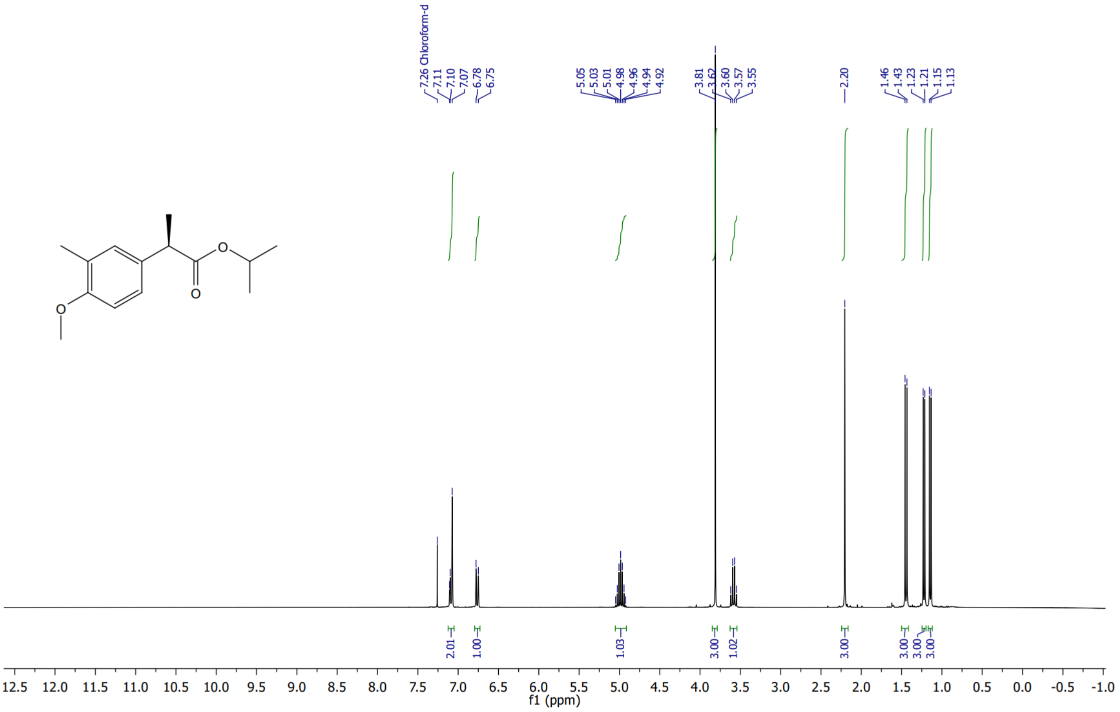

^{13}C NMR (101 MHz, $CDCl_3$)

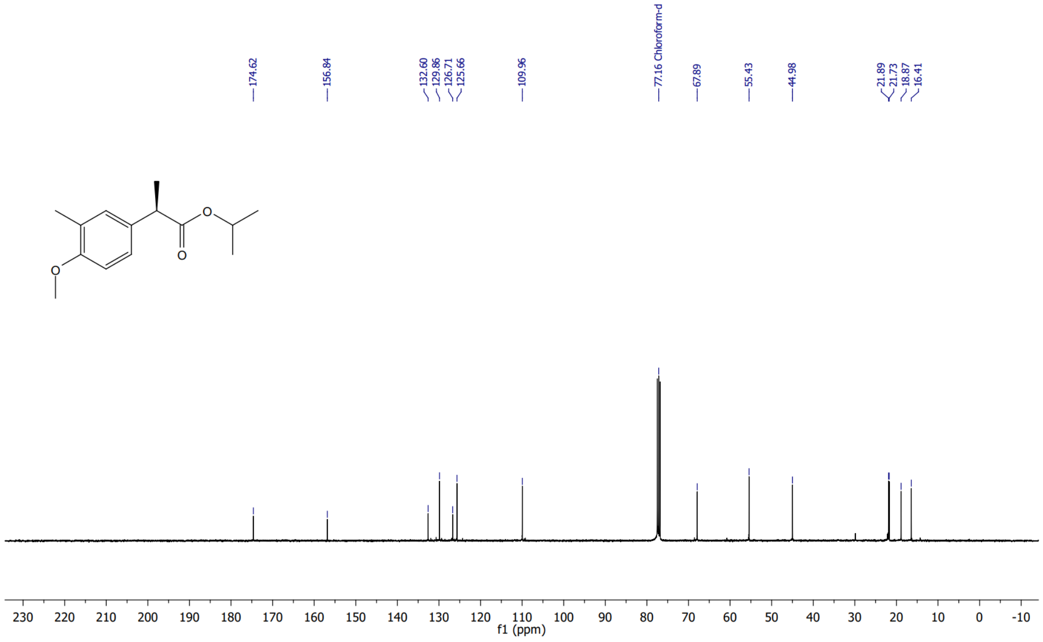

Methyl (*R*)-2-(2-methoxyphenyl)propanoate [(*R*)-21a]

^{1}H NMR (500 MHz, $CDCl_3$)

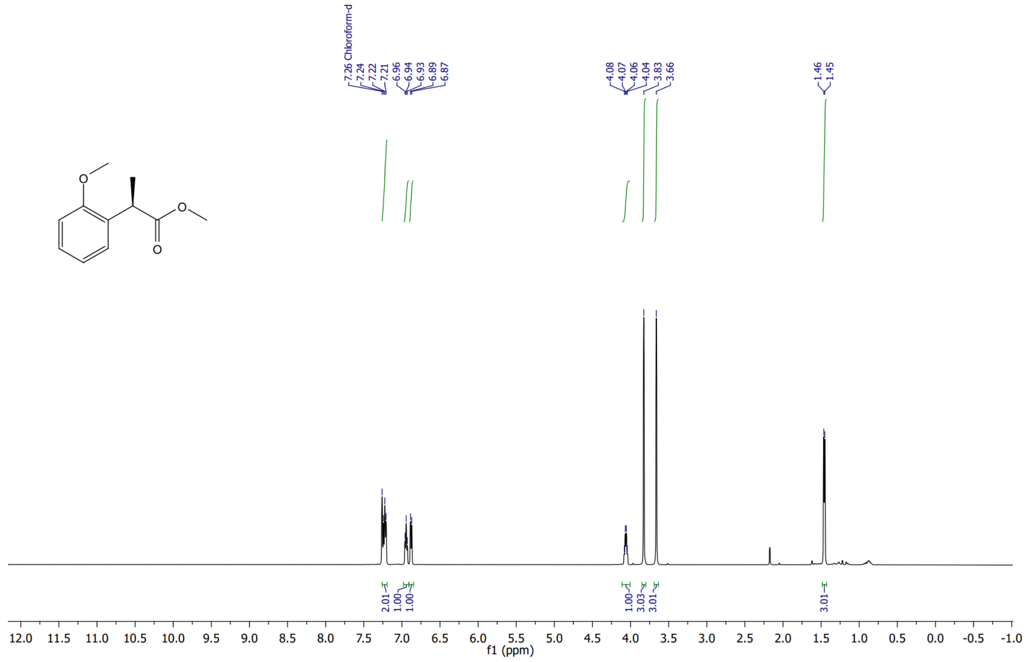

^{13}C NMR (126 MHz, $CDCl_3$)

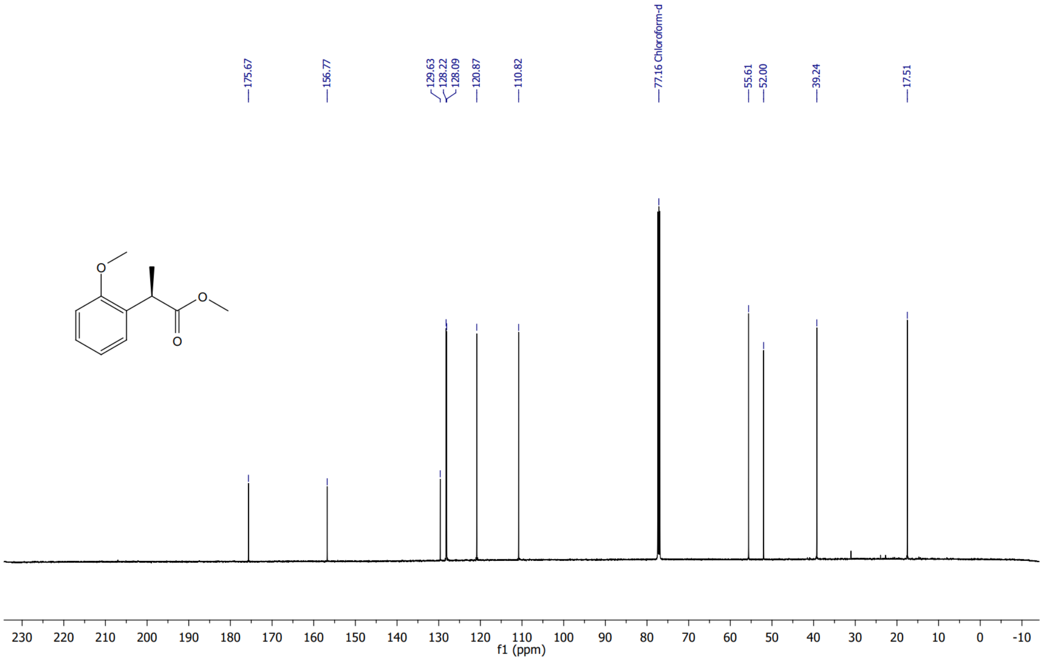

Ethyl (*R*)-2-(2-methoxyphenyl)propanoate [(*R*)-21b]

^{1}H NMR (500 MHz, $CDCl_3$)

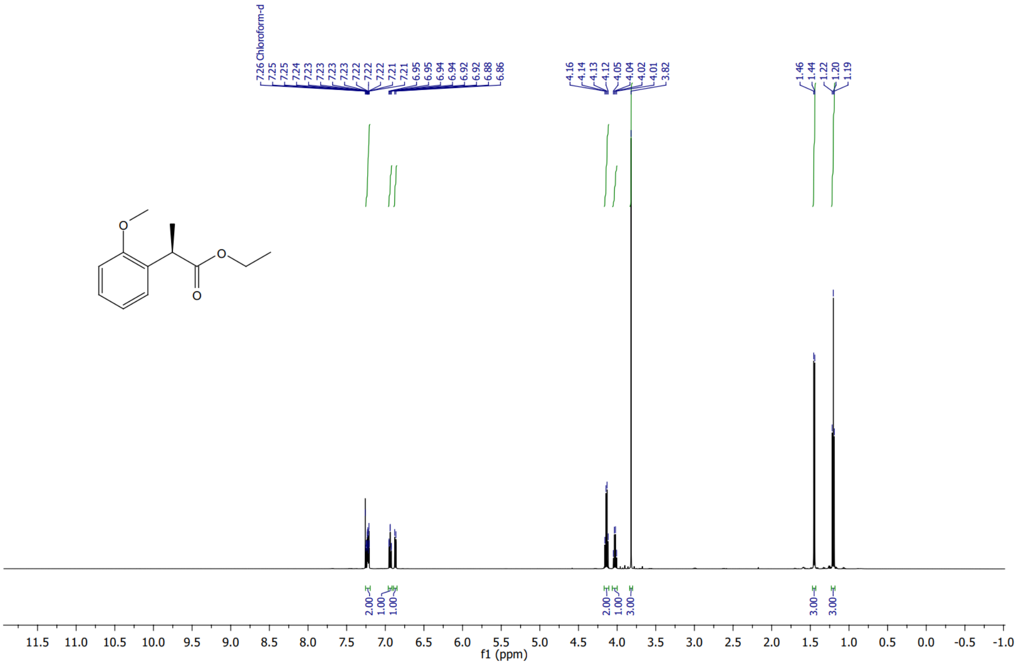

^{13}C NMR (126 MHz, $CDCl_3$)

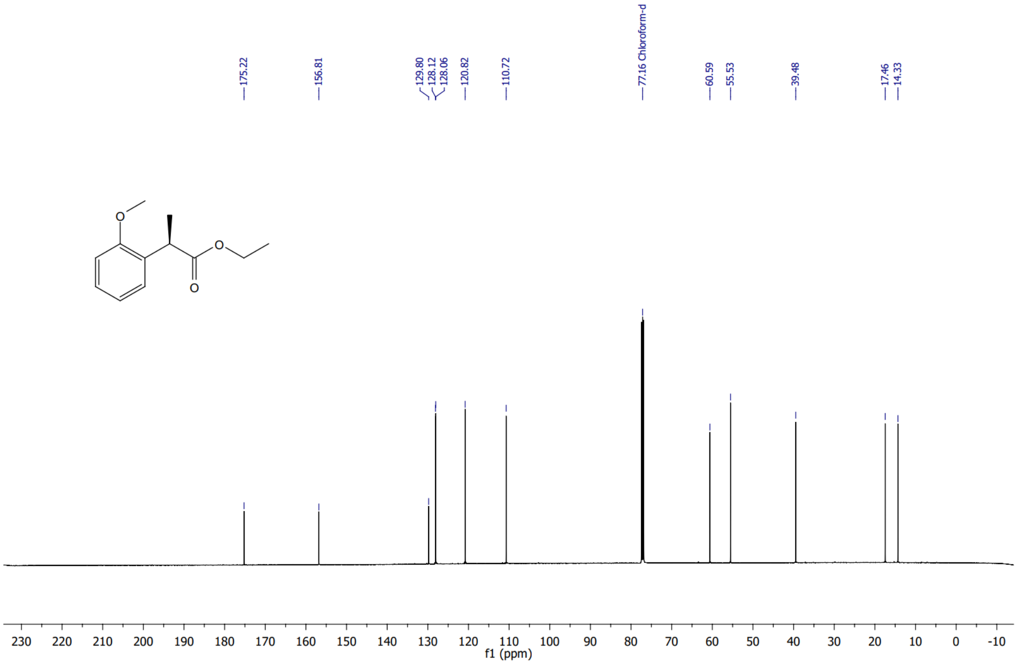

Isopropyl (*R*)-2-(2-methoxyphenyl)propanoate [(*R*)-21c]

^{1}H NMR (500 MHz, $CDCl_3$)

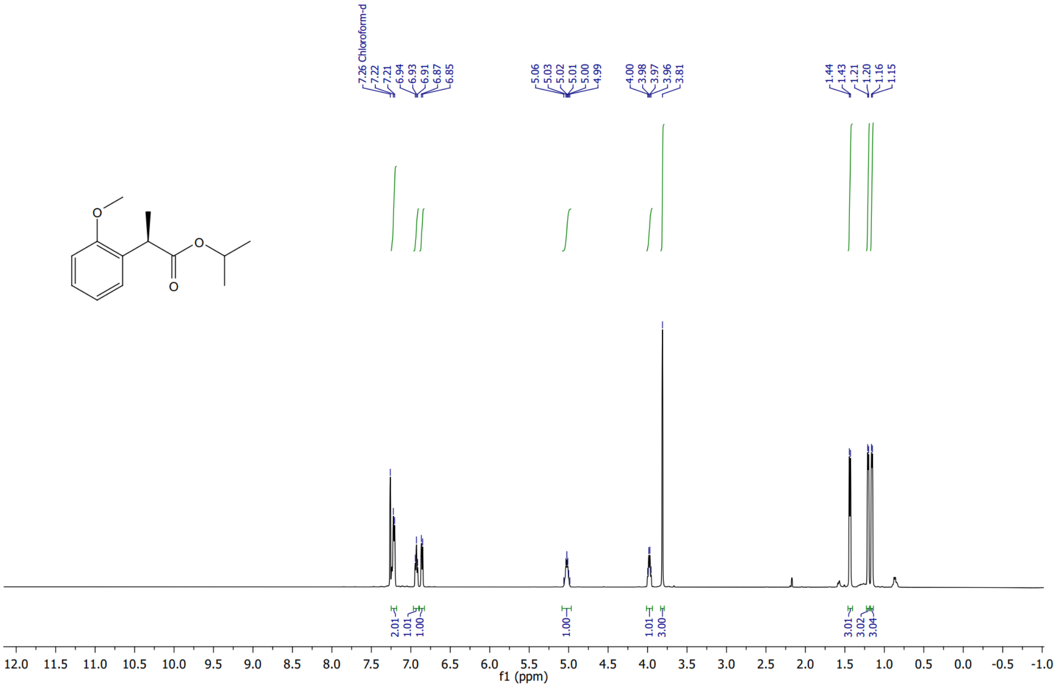

^{13}C NMR (126 MHz, $CDCl_3$)

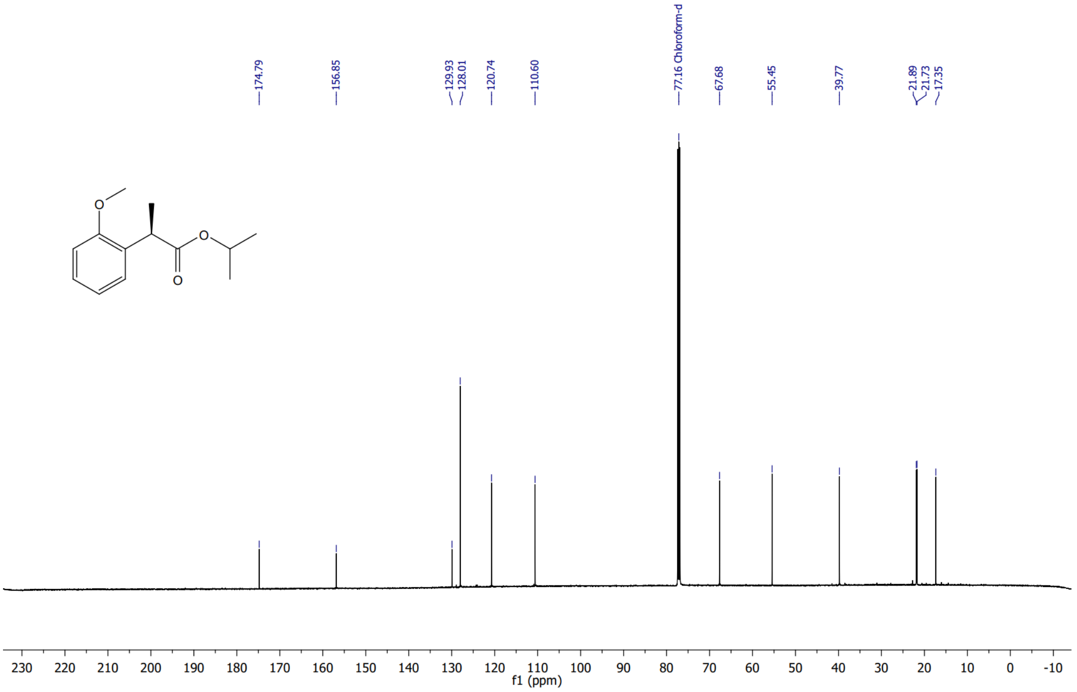

Methyl (*R*)-2-(4-methoxyphenyl)butanoate [(*R*)-22a]

^{1}H NMR (400 MHz, $CDCl_3$)

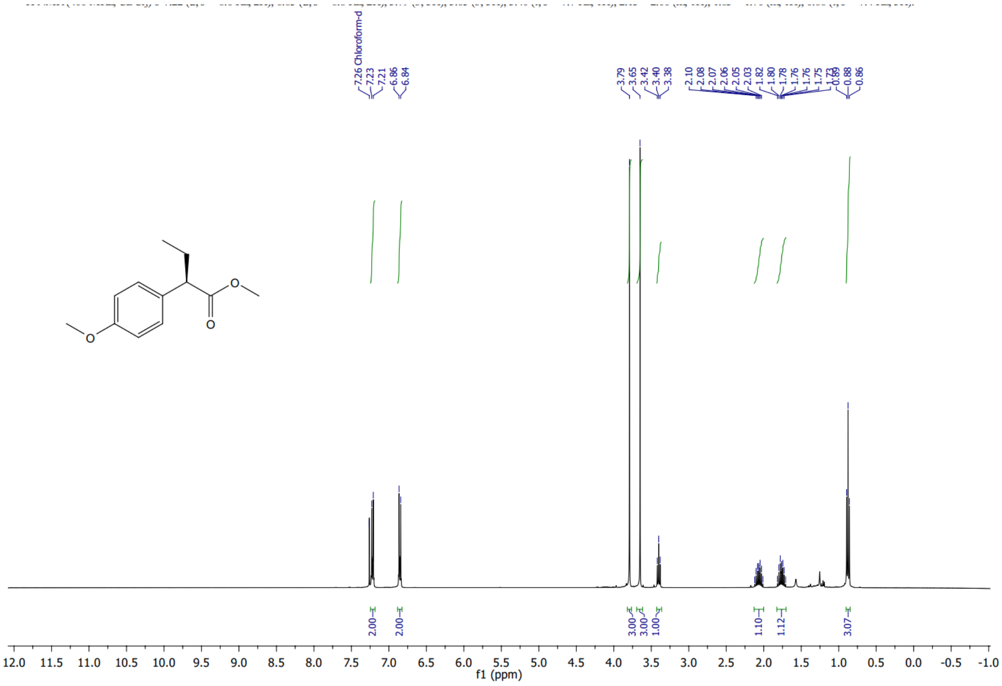

^{13}C NMR (101 MHz, $CDCl_3$)

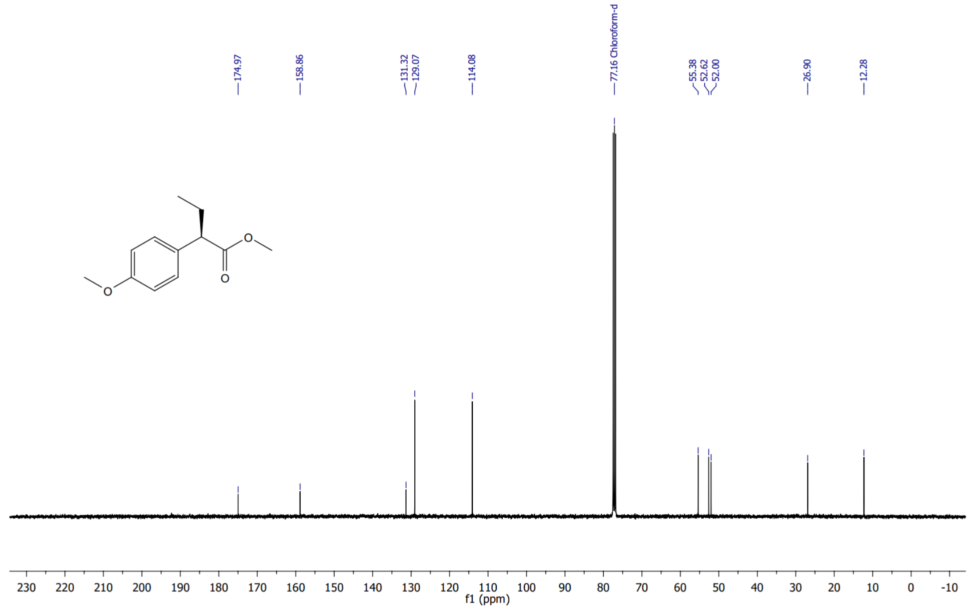

Ethyl (*R*)-2-(4-methoxyphenyl)butanoate [(*R*)-22b]

^{1}H NMR (400 MHz, $CDCl_3$)

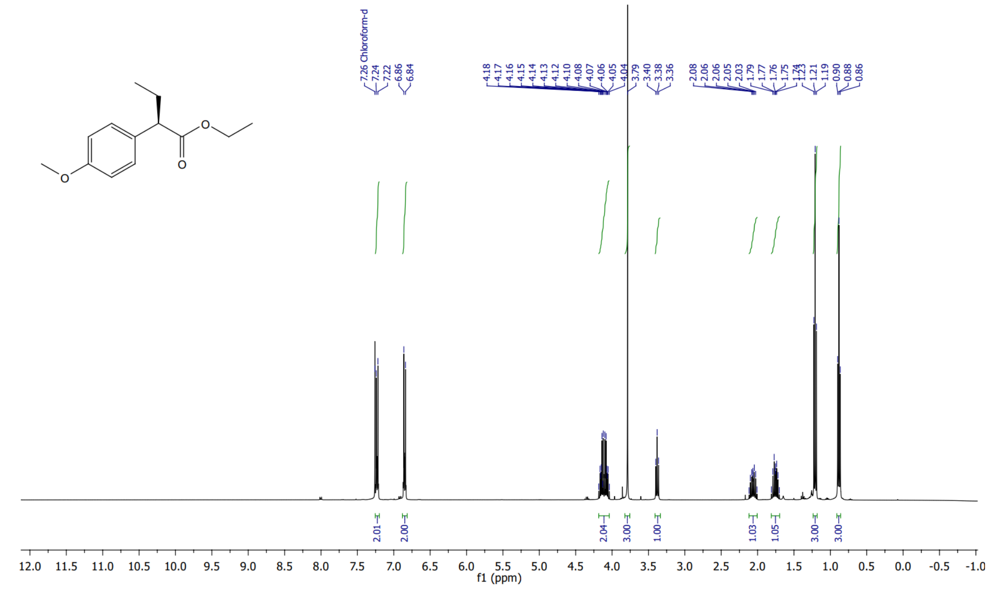

^{13}C NMR (101 MHz, $CDCl_3$)

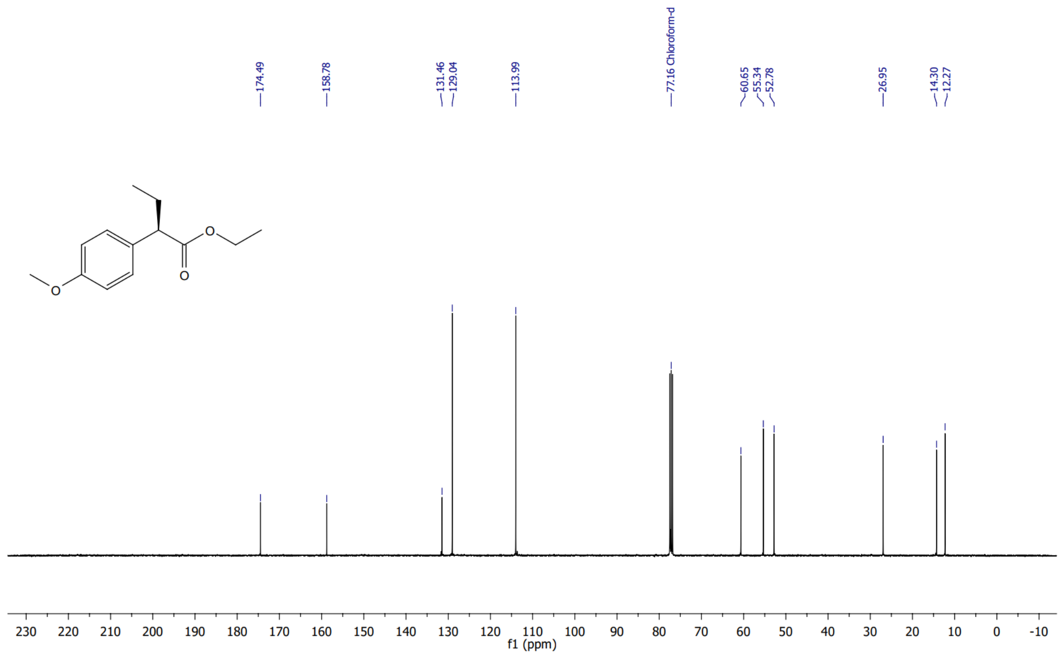

Isopropyl (*R*)-2-(4-methoxyphenyl)butanoate [(*R*)-22c]

^{1}H NMR (300 MHz, $CDCl_3$)

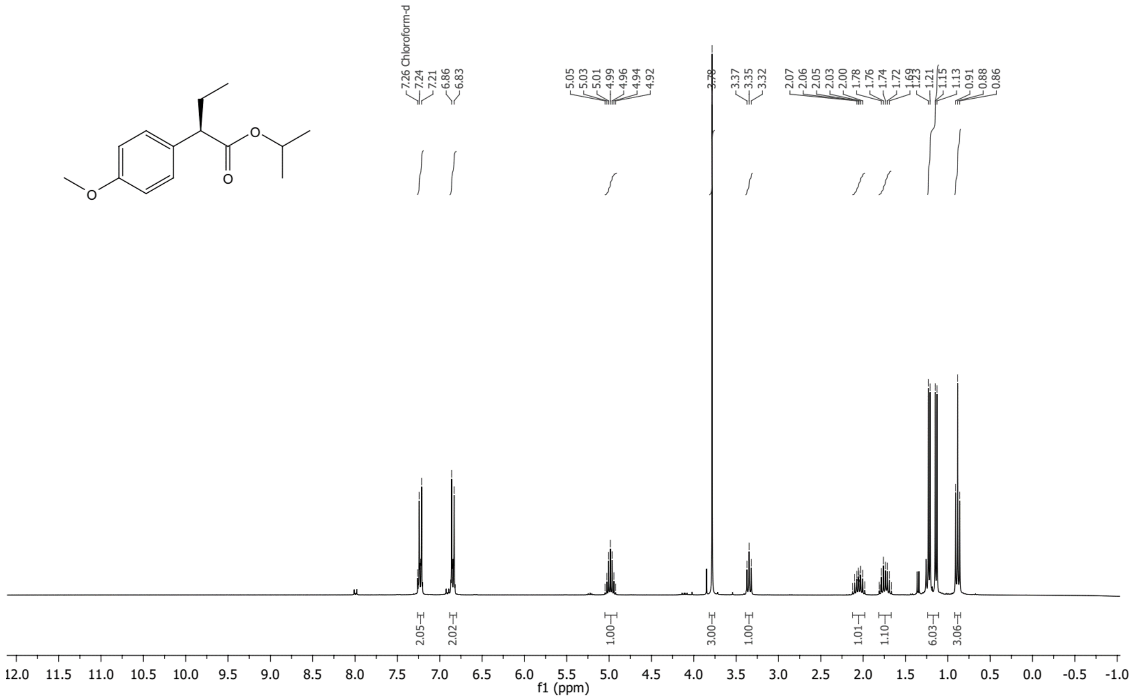

^{13}C NMR (101 MHz, $CDCl_3$)

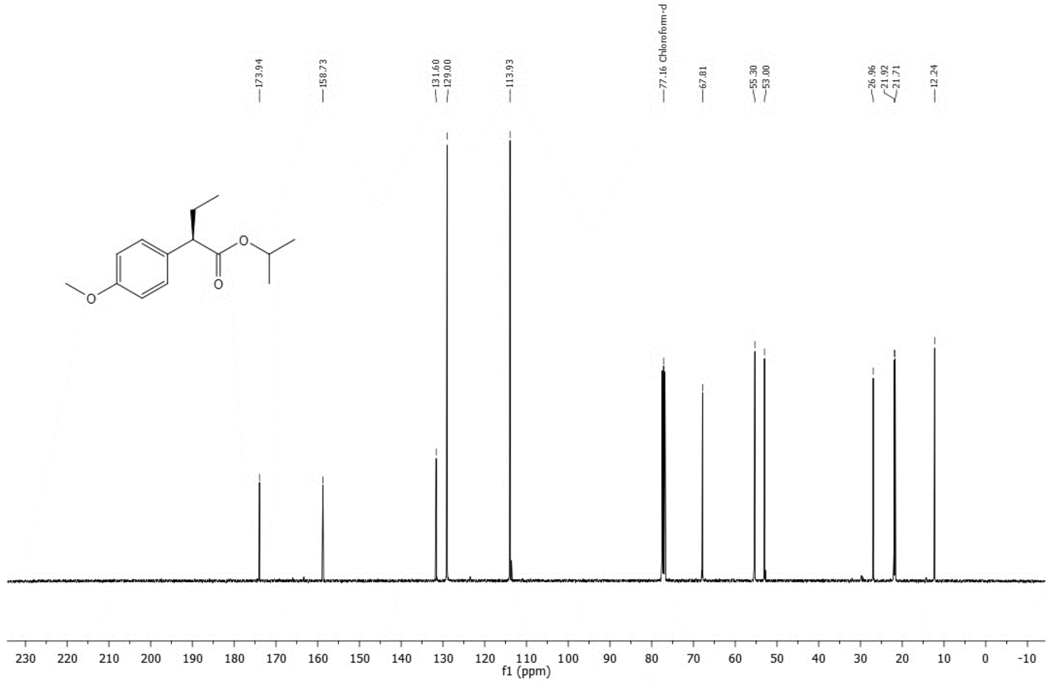

Methyl (*R*)-2-(2,4-dimethoxyphenyl)propanoate [(*R*)-23a]

^{1}H NMR (300 MHz, $CDCl_3$)

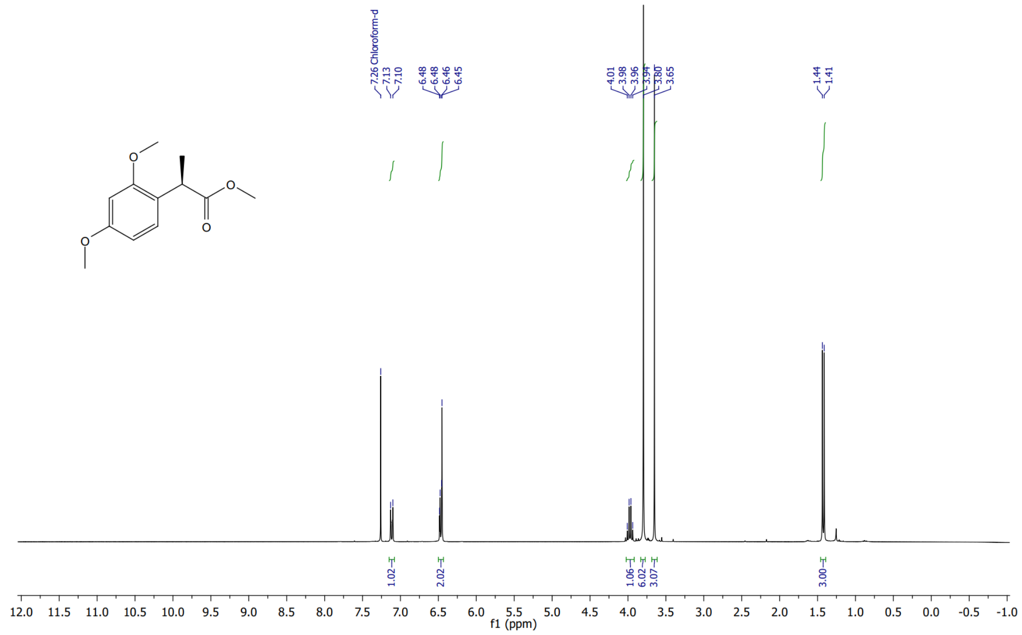

^{13}C NMR (126 MHz, $CDCl_3$)

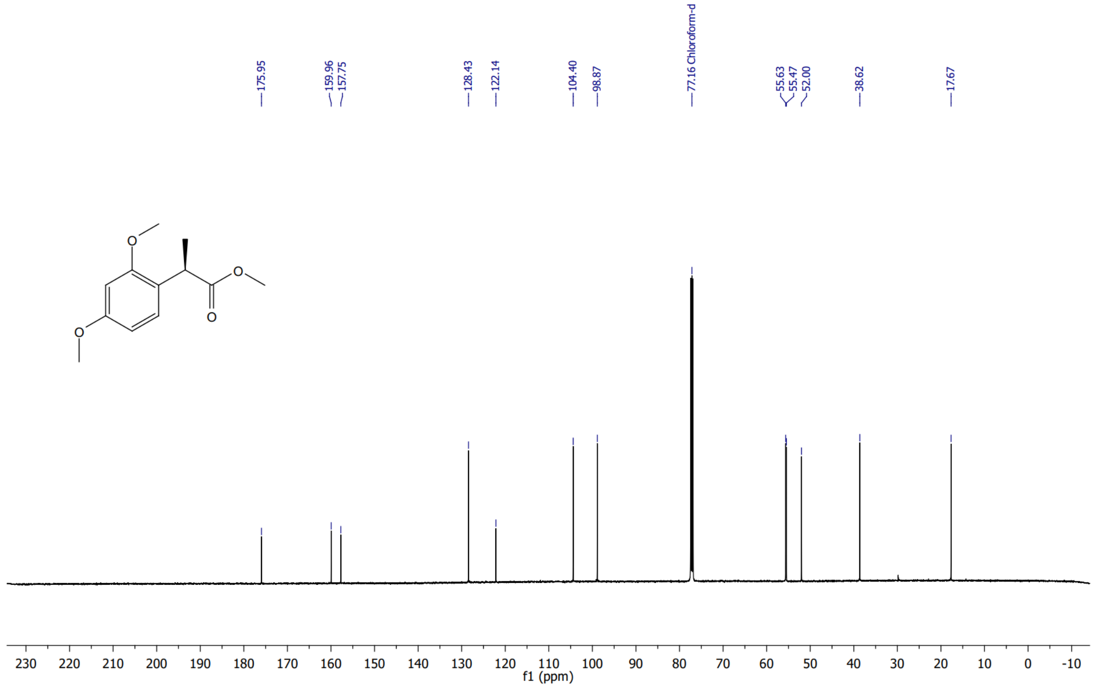

Ethyl (*R*)-2-(2,4-dimethoxyphenyl)propanoate [(*R*)-23b]

^{1}H NMR (400 MHz, $CDCl_3$)

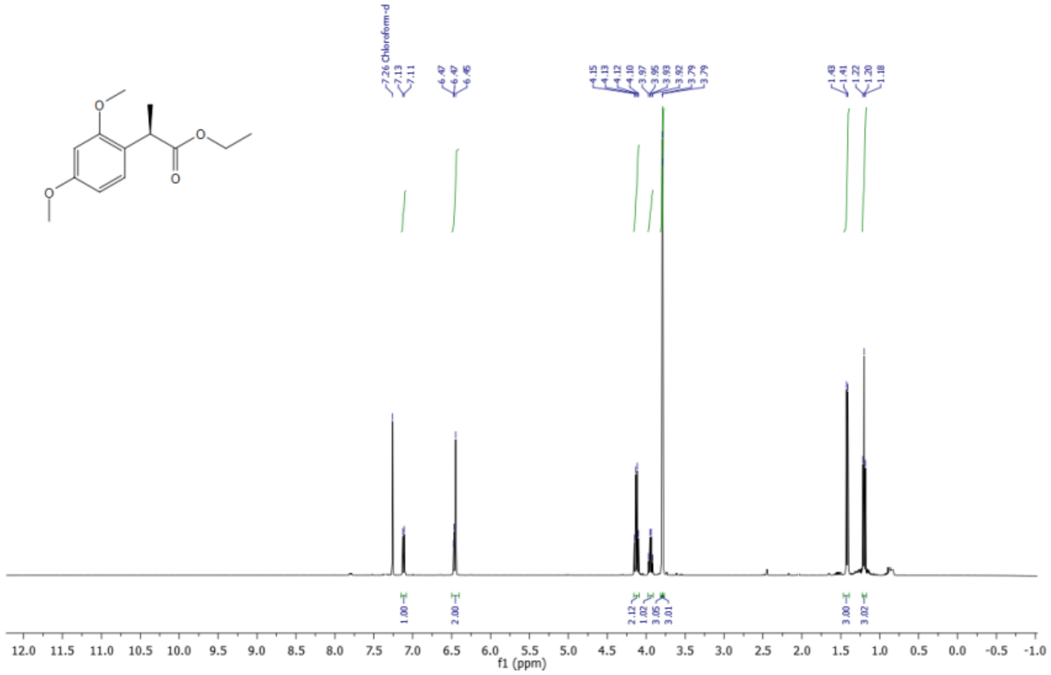

^{13}C NMR (101 MHz, $CDCl_3$)

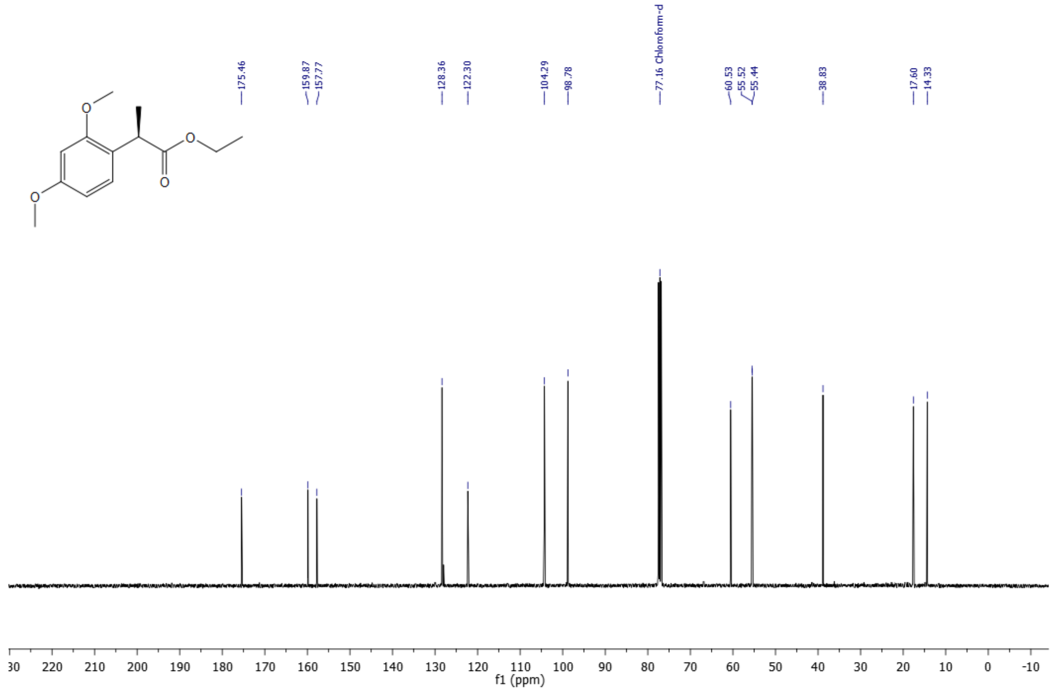

Isopropyl (*R*)-2-(2,4-dimethoxyphenyl)propanoate [(*R*)-23c]

^{1}H NMR (400 MHz, $CDCl_3$)

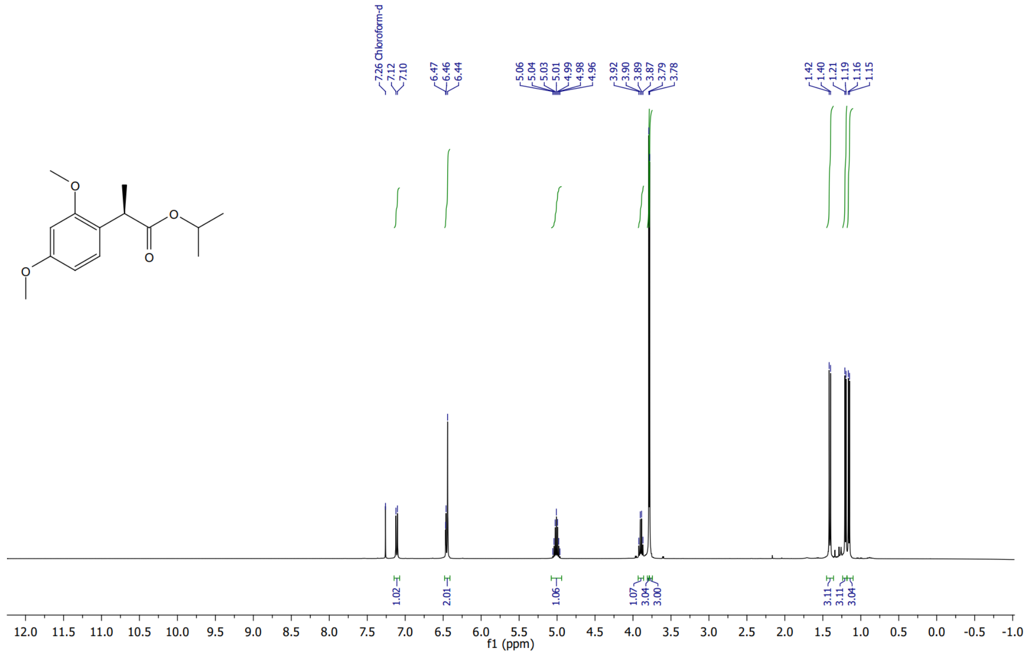

^{13}C NMR (101 MHz, $CDCl_3$)

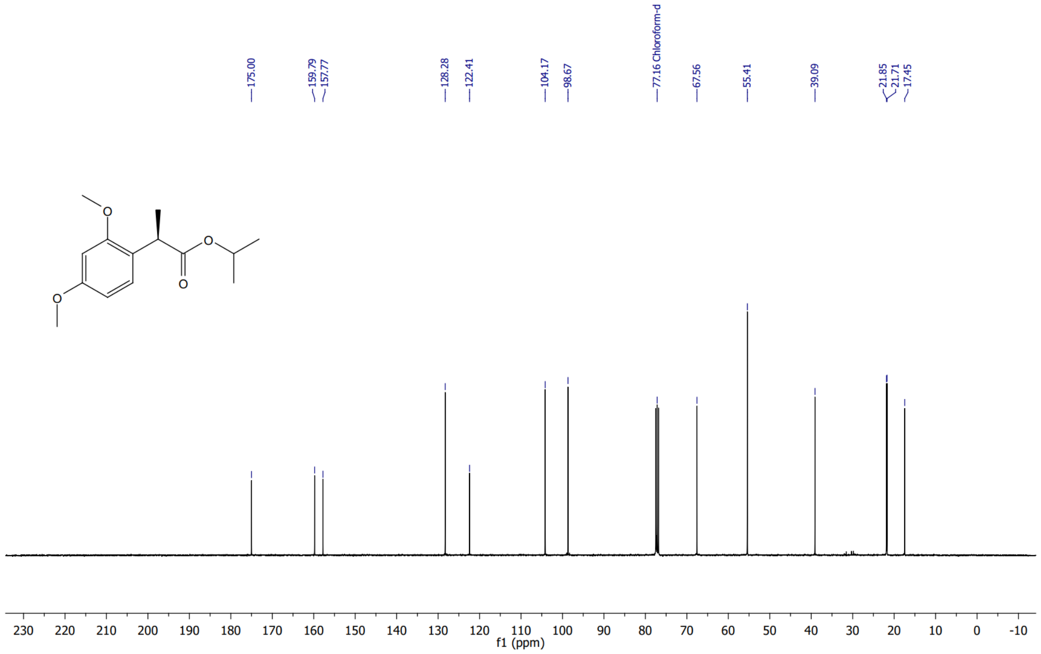

Methyl (*R*)-2-(2,4-dimethoxy-3-methylphenyl)propanoate [(*R*)-24a]

^{1}H NMR (300 MHz, $CDCl_3$)

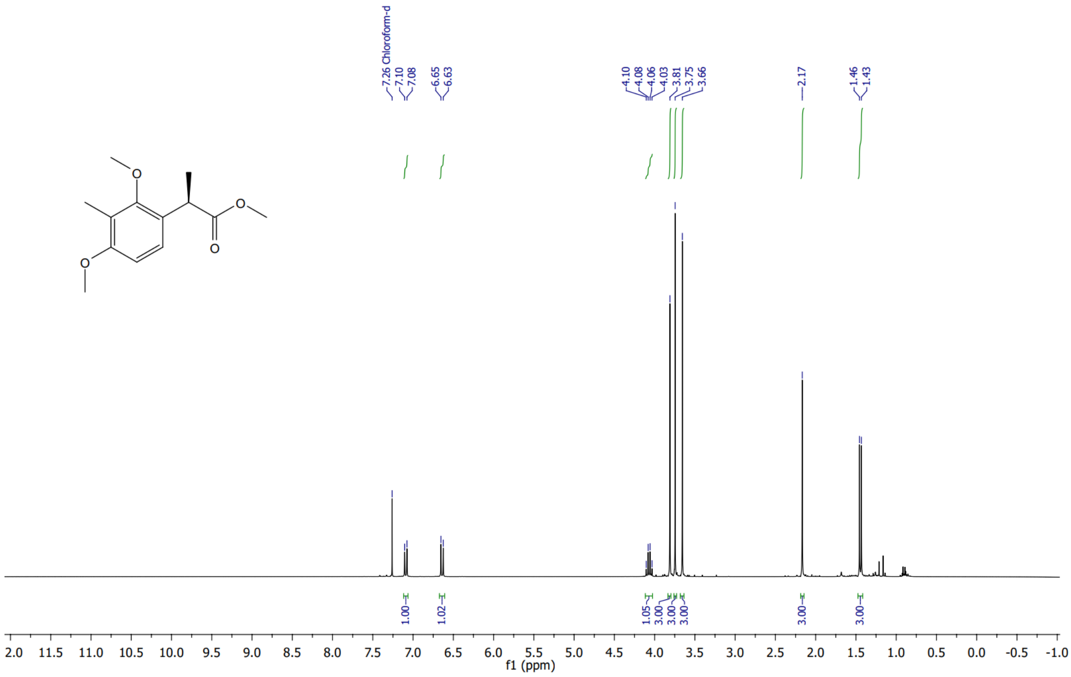

^{13}C NMR (126 MHz, $CDCl_3$)

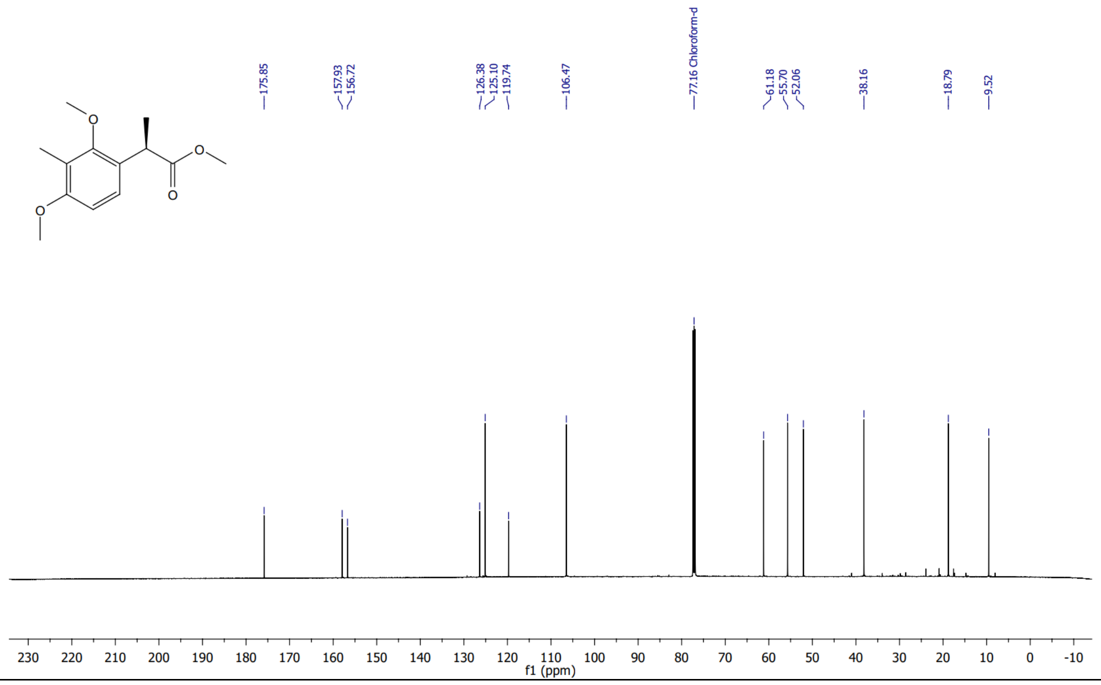

Ethyl (*R*)-2-(2,4-dimethoxy-3-methylphenyl)propanoate [(*R*)-24b]

^{1}H NMR (500 MHz, $CDCl_3$)

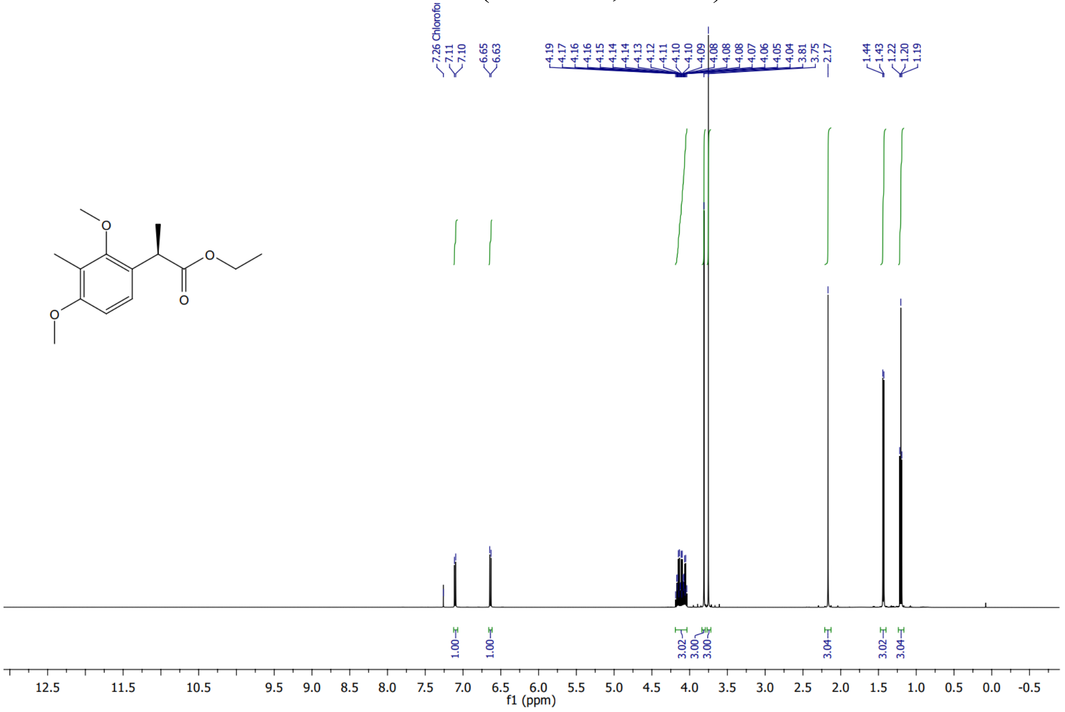

^{13}C NMR (126 MHz, $CDCl_3$)

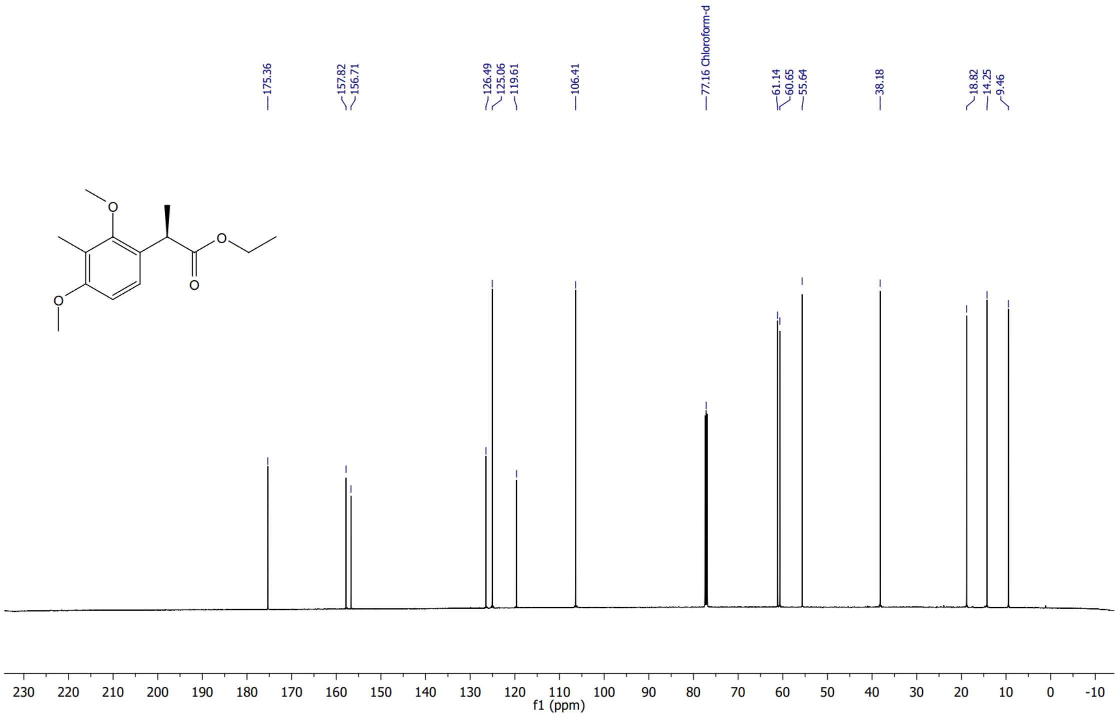

2-Methoxy-6-(prop-1-yn-1-yl)naphthalene (25)

^{1}H NMR (400 MHz, $CDCl_3$)

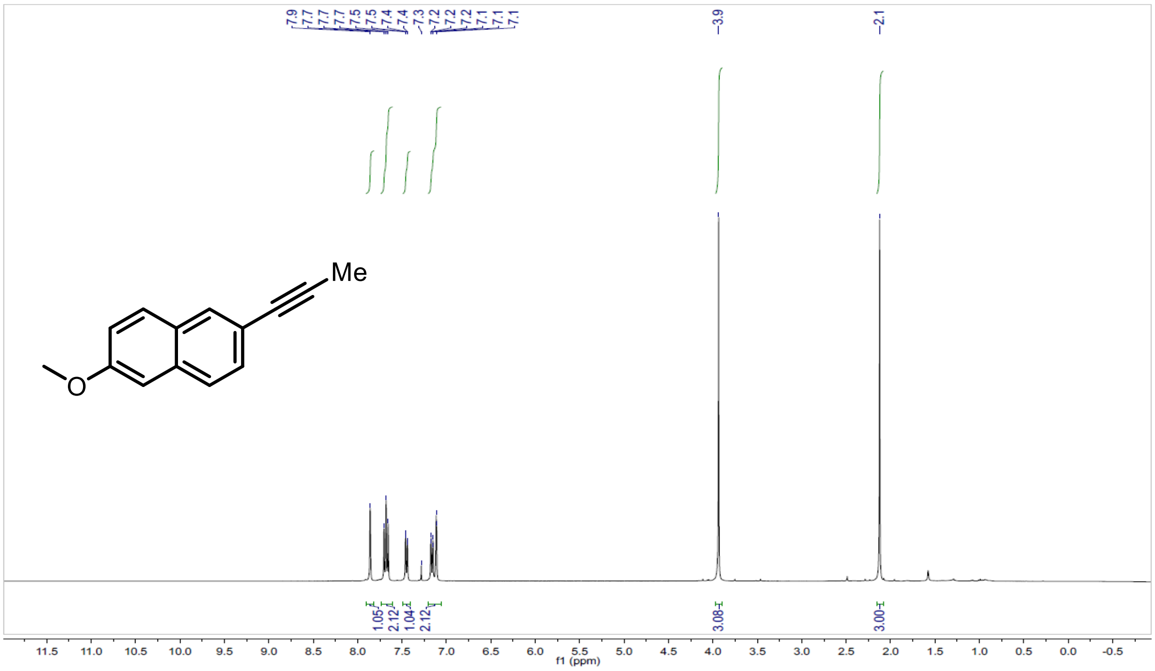

^{13}C NMR (101 MHz, $CDCl_3$)

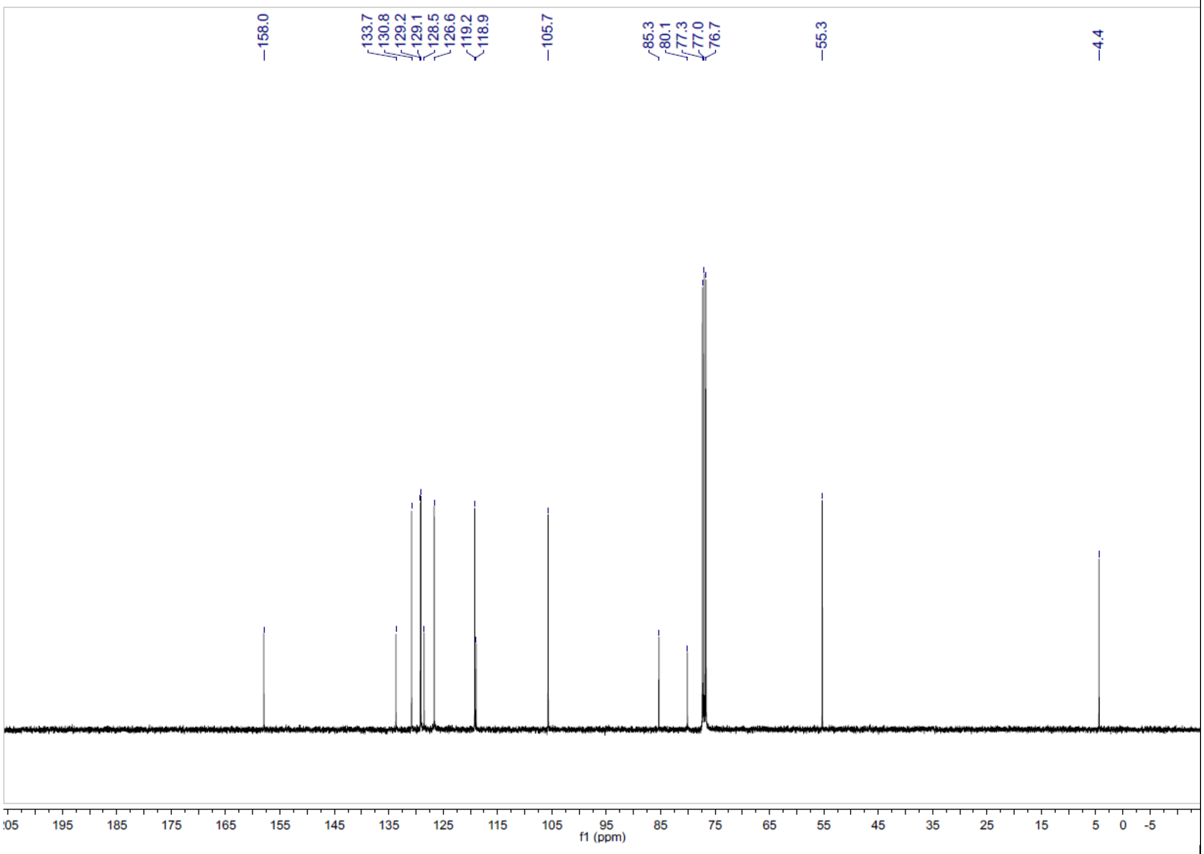

Ethyl 2-(6-methoxynaphthalen-2-yl)propanoate (26)

^{1}H NMR (300 MHz, $CDCl_3$)

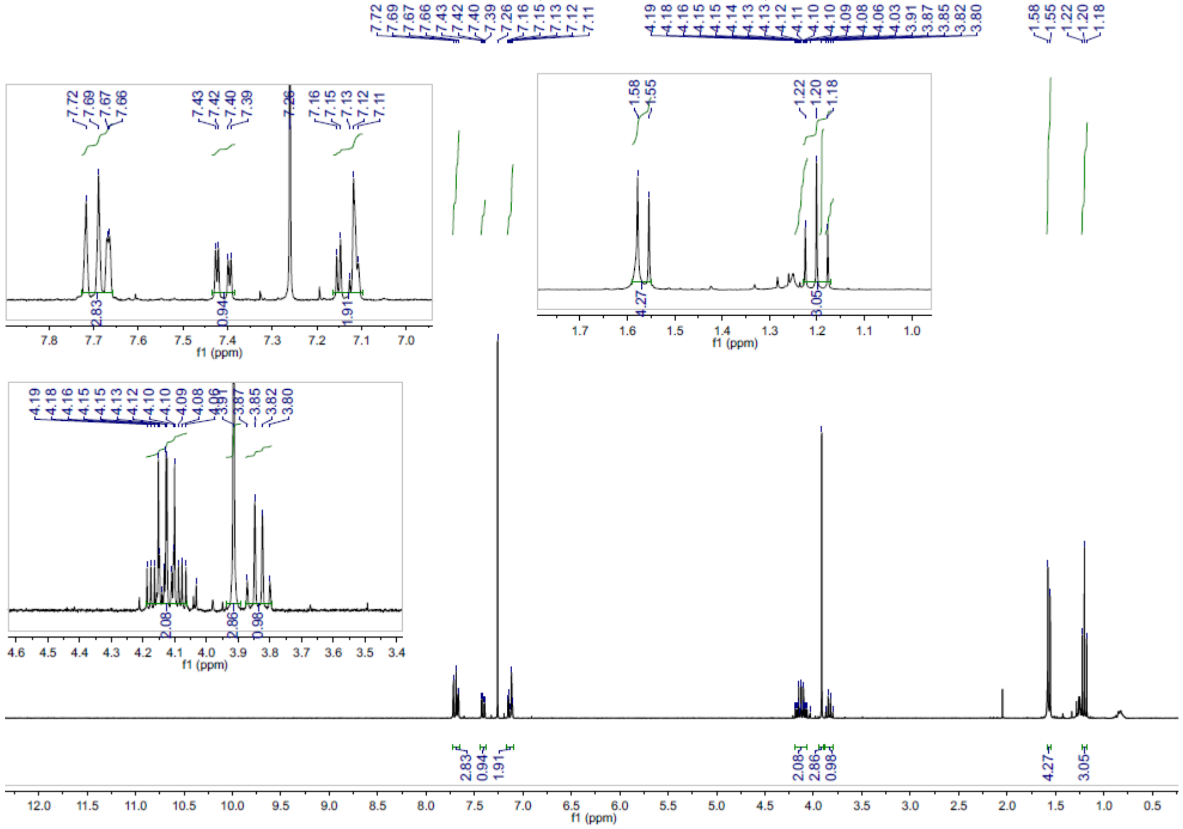

^{13}C NMR (126 MHz, $CDCl_3$)

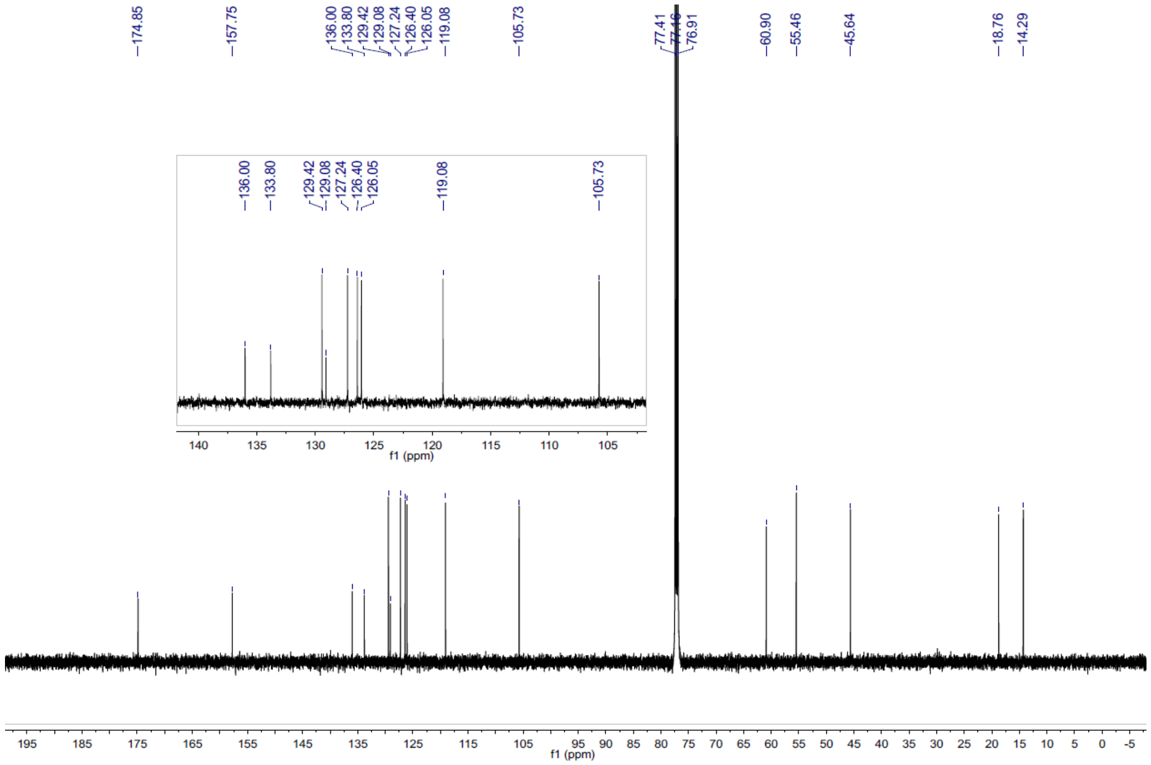

1-Methyl-4-(prop-1-yn-1-yl)benzene (S26b)

^{1}H NMR (400 MHz, $CDCl_3$)

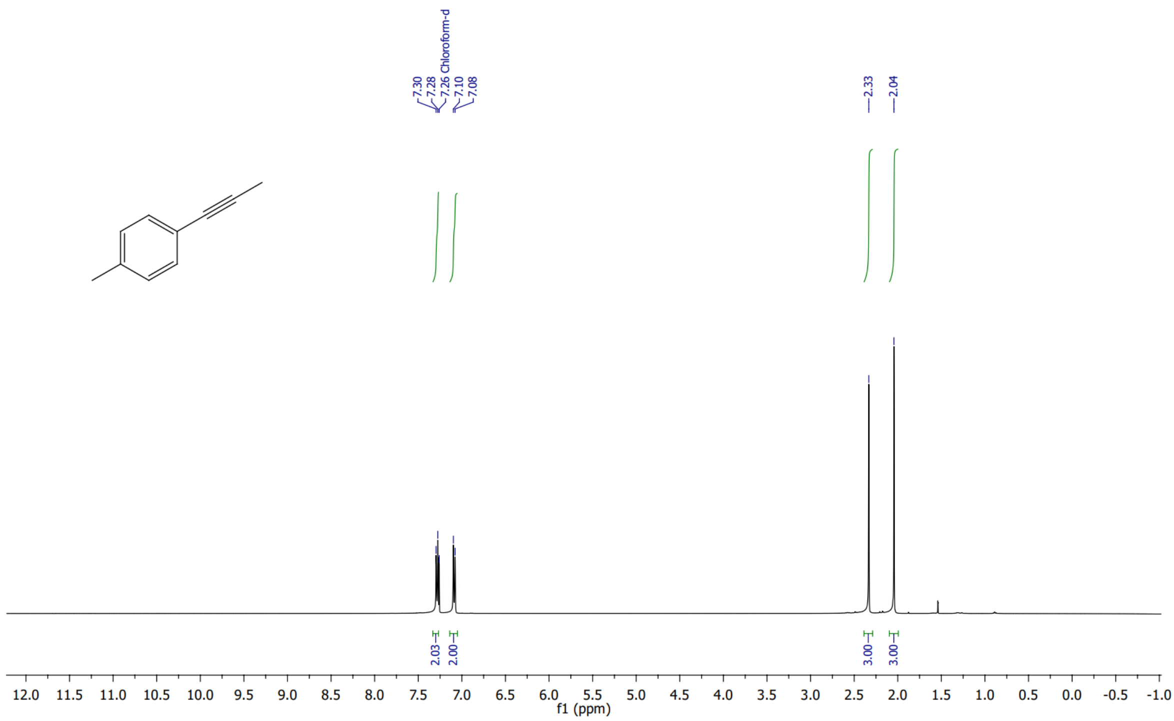

^{13}C NMR (101 MHz, $CDCl_3$)

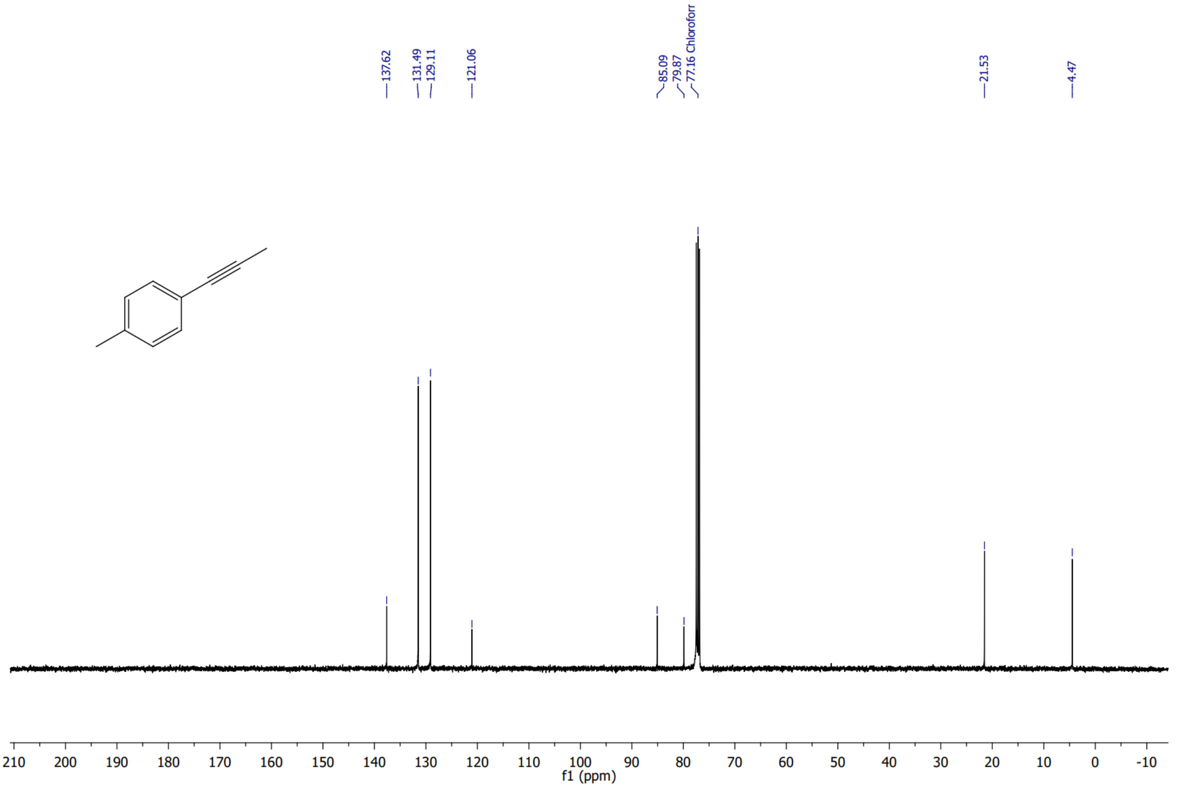

1-Fluoro-4-(prop-1-yn-1-yl)benzene (S26c)

^{1}H NMR (400 MHz, $CDCl_3$)

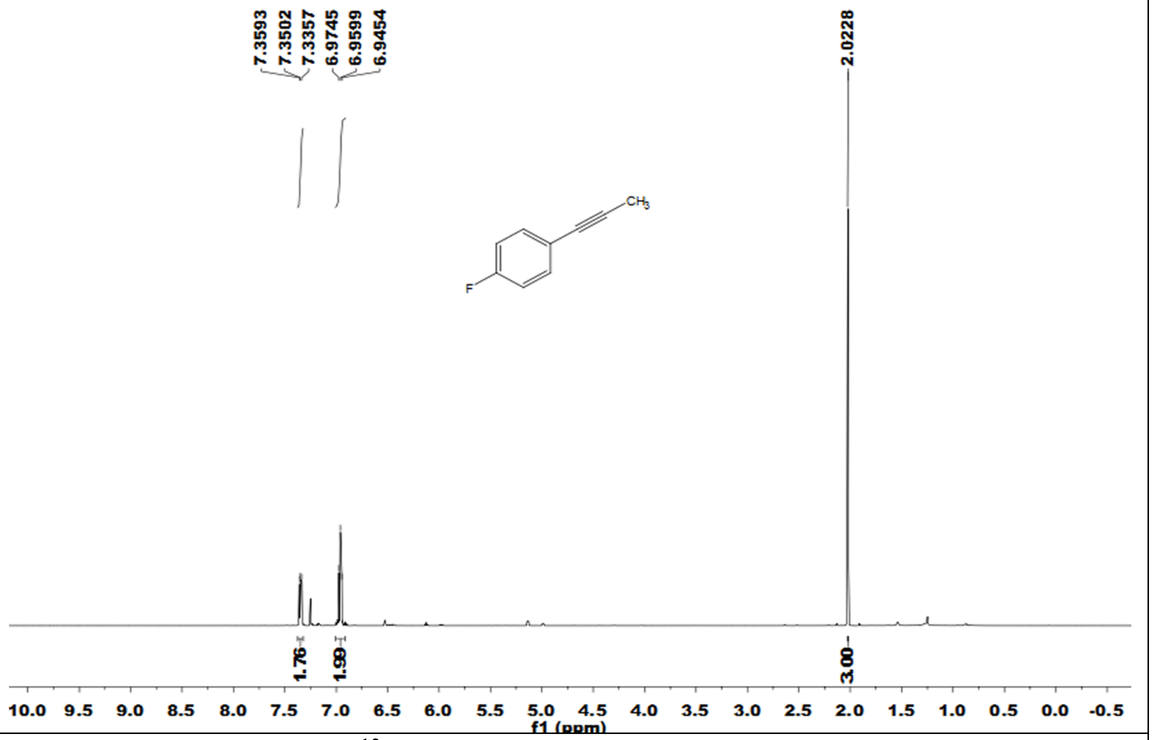

^{13}C NMR (101 MHz, $CDCl_3$)

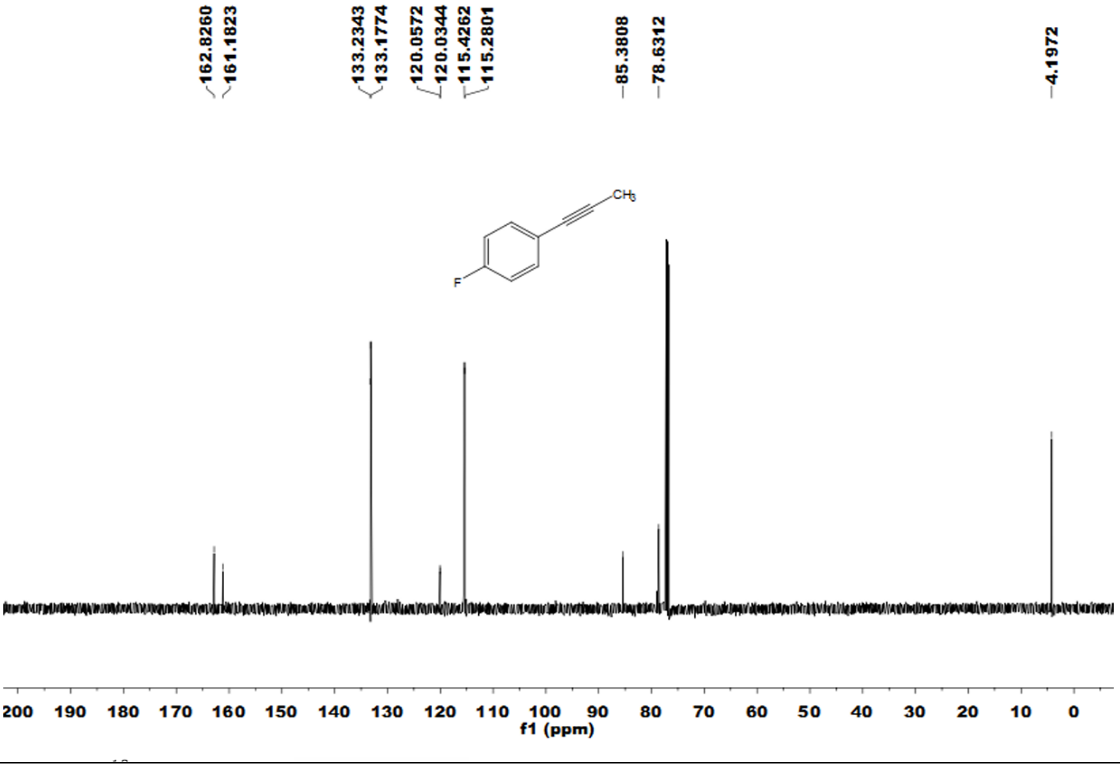

1-(Prop-1-yn-1-yl)-4-(trifluoromethyl)benzene (S26d)

^{1}H NMR (500 MHz, $CDCl_3$)

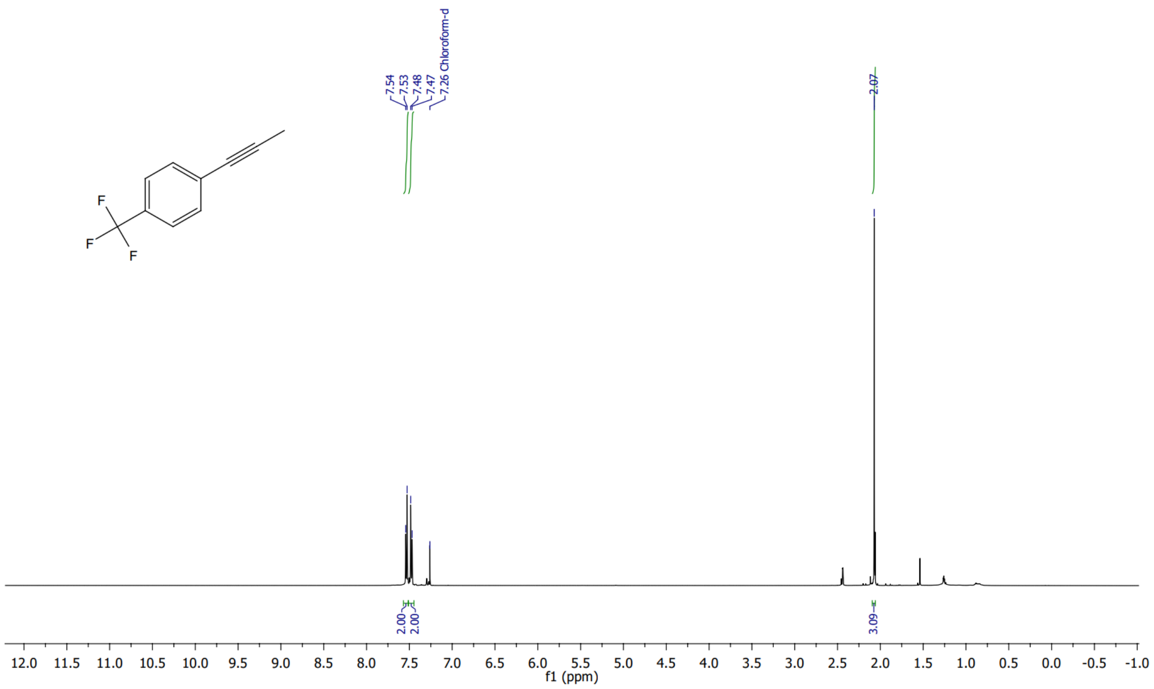

^{13}C NMR (126 MHz, $CDCl_3$)

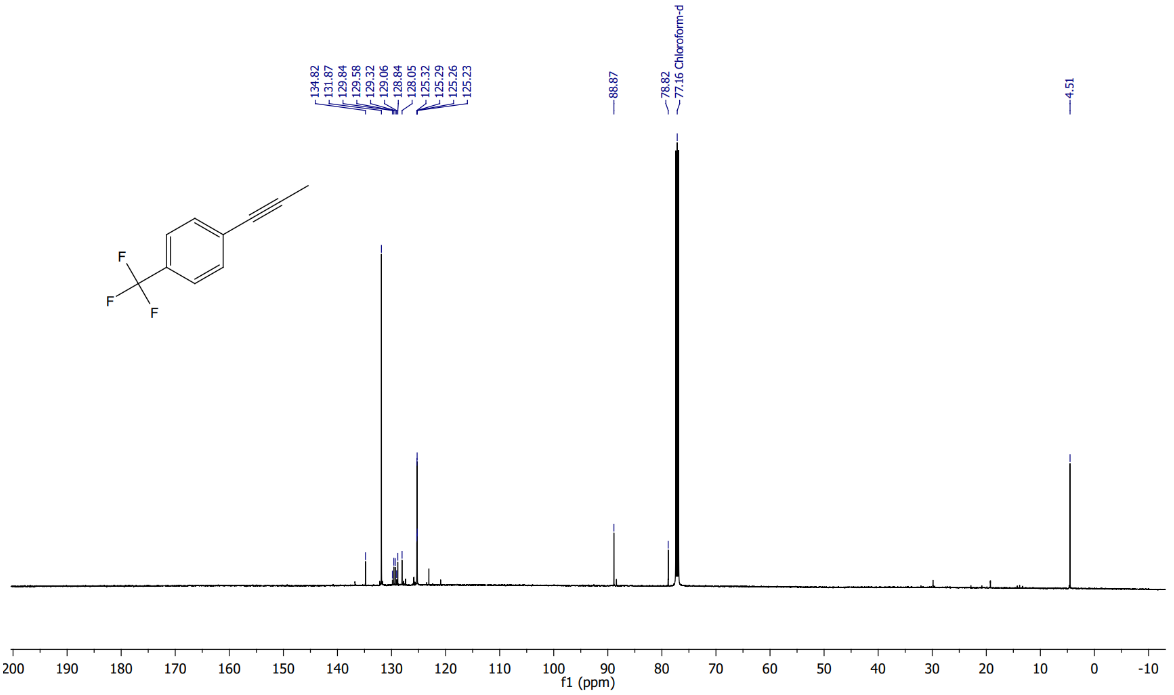

***N*,*N*-Dimethyl-4-(prop-1-yn-1-yl)aniline (S26e)**

^{1}H NMR (500 MHz, $CDCl_3$)

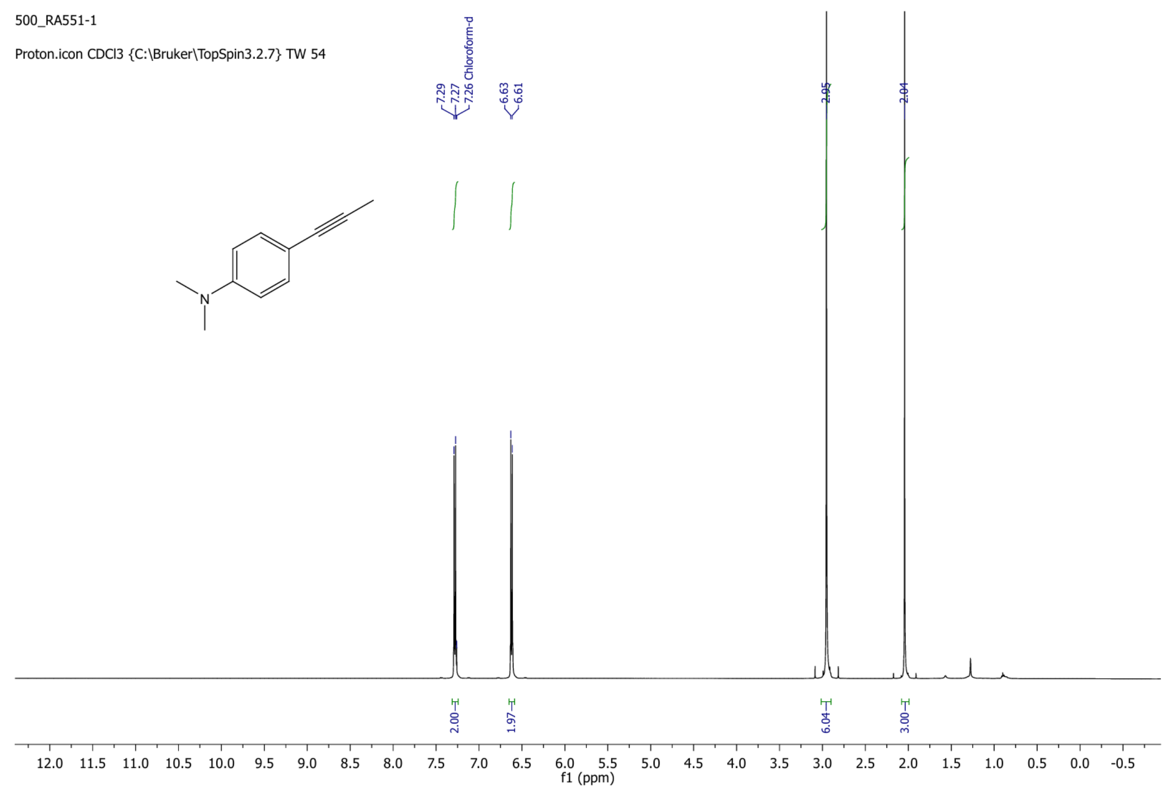

^{13}C NMR (126 MHz, $CDCl_3$)

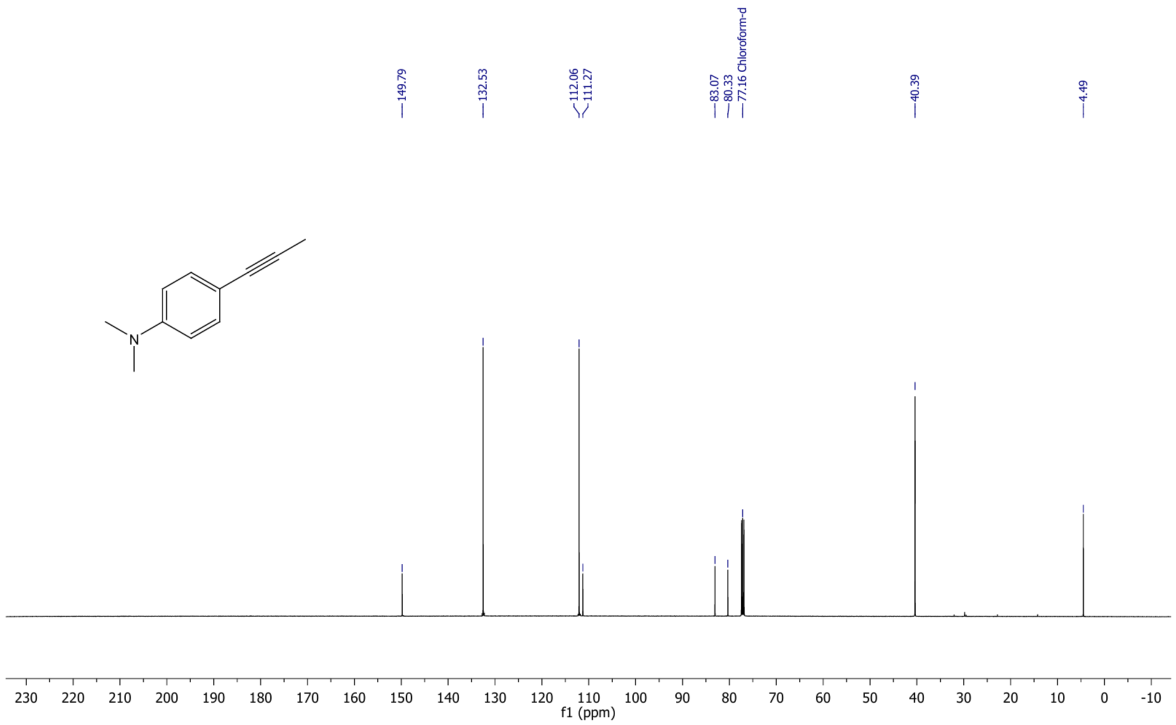

1-Chloro-3-(prop-1-yn-1-yl)benzene (S26f)

^{1}H NMR (400 MHz, $CDCl_3$)

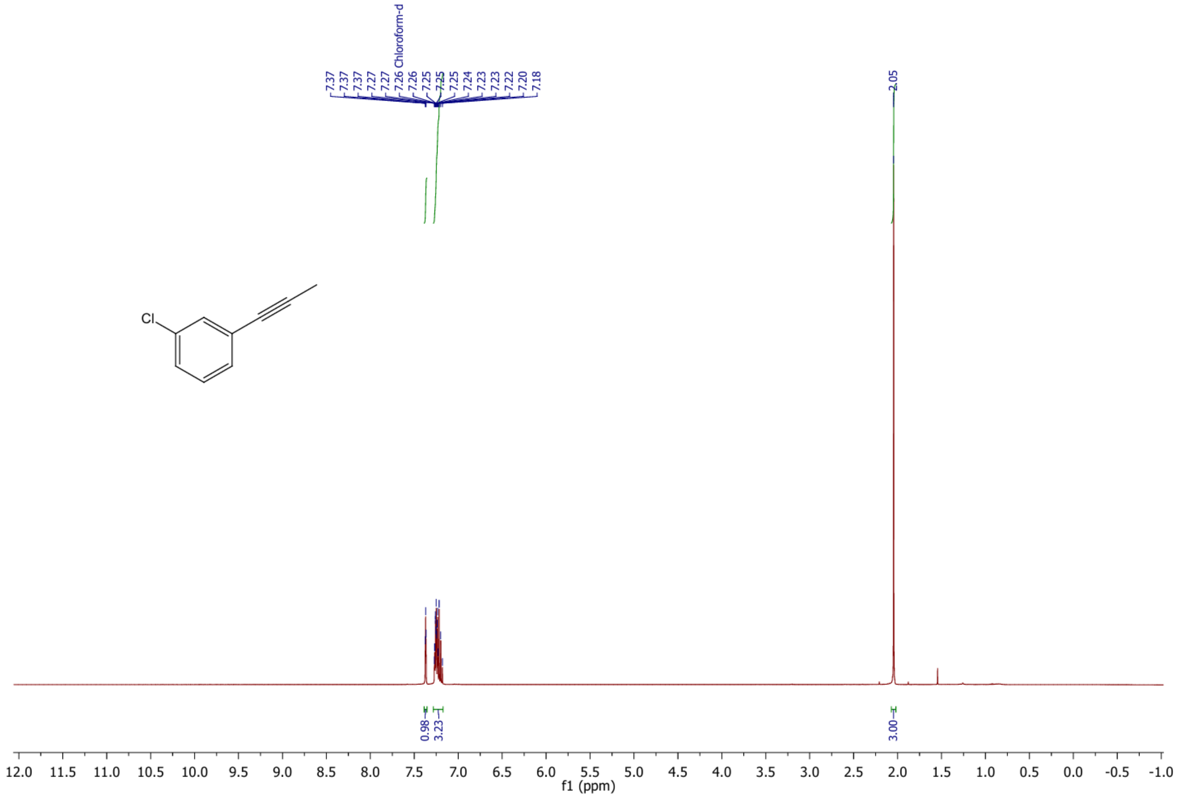

^{13}C NMR (101 MHz, $CDCl_3$)

^{13}C NMR (101 MHz, $CDCl_3$) δ 134.12, 131.57, 129.77, 129.55, 127.96, 125.89, 87.45, 78.62, 4.46.

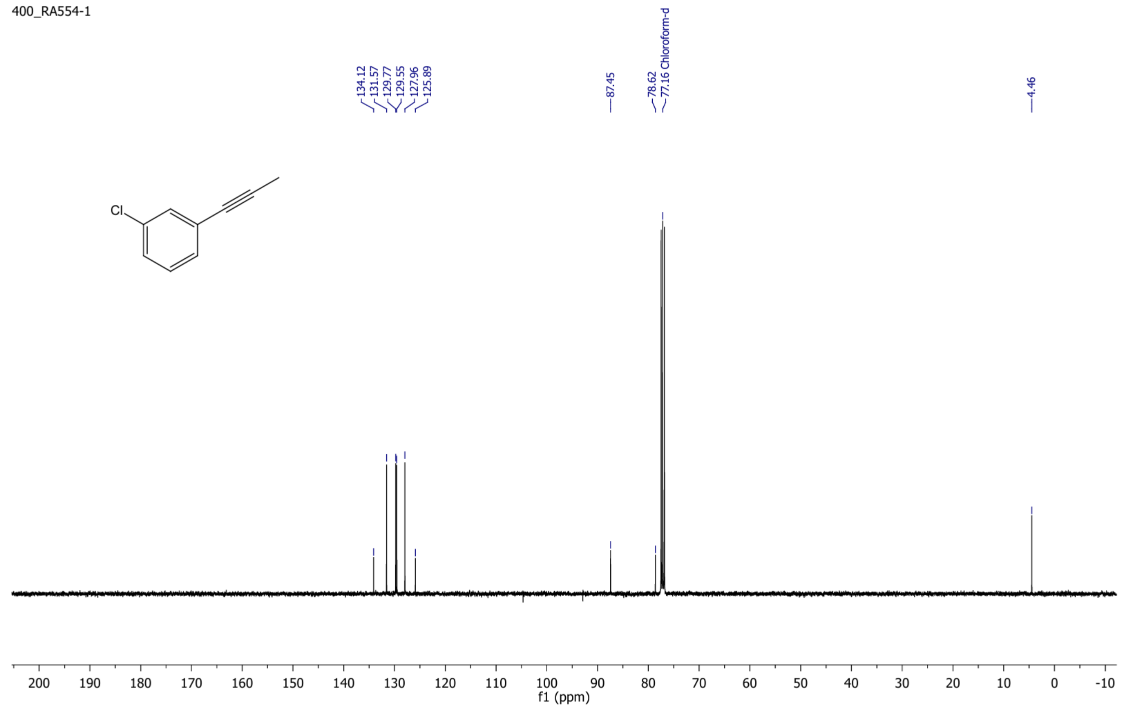

1-Methoxy-3-(prop-1-yn-1-yl)benzene (S26g)

^{1}H NMR (300 MHz, $CDCl_3$)

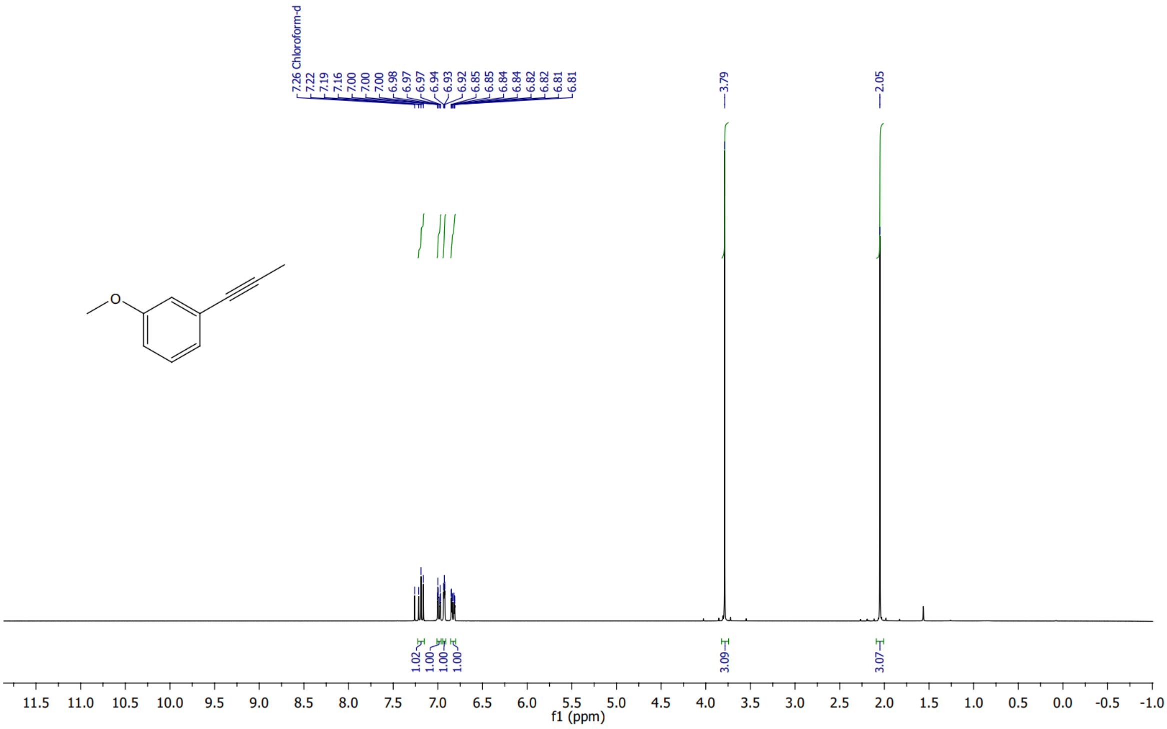

^{13}C NMR (75 MHz, $CDCl_3$)

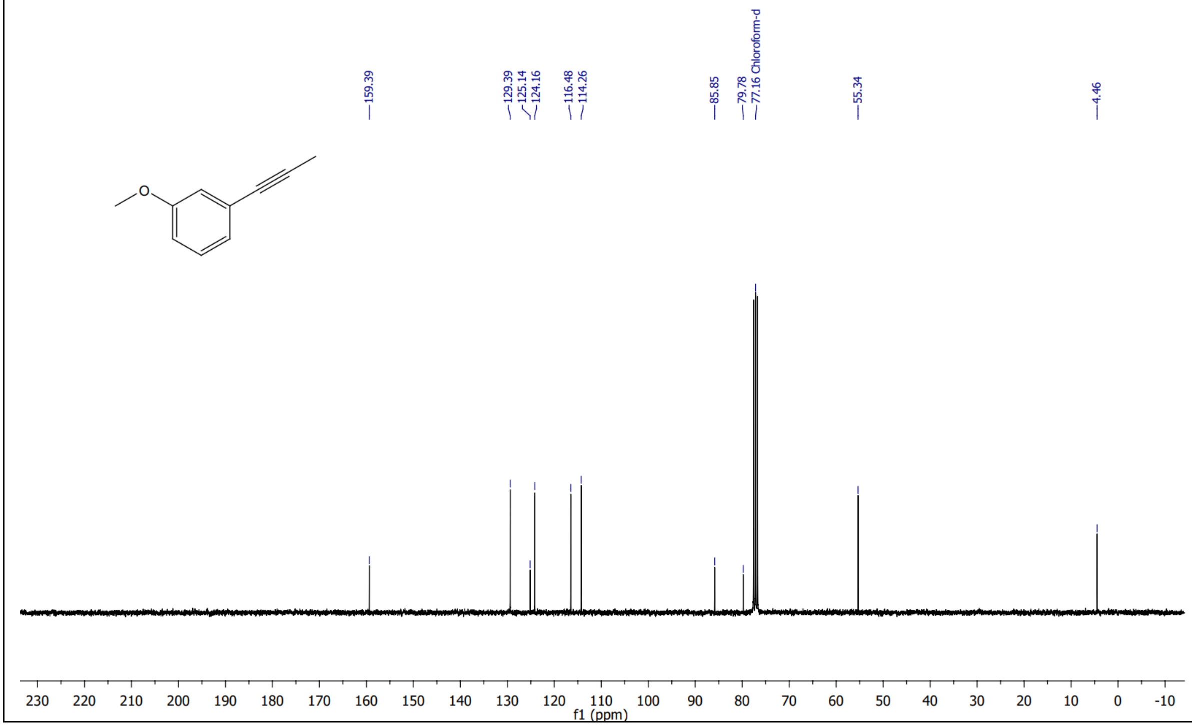

1-Methoxy-3-methyl-5-(prop-1-yn-1-yl)benzene (S26h)

^{1}H NMR (300 MHz, $CDCl_3$)

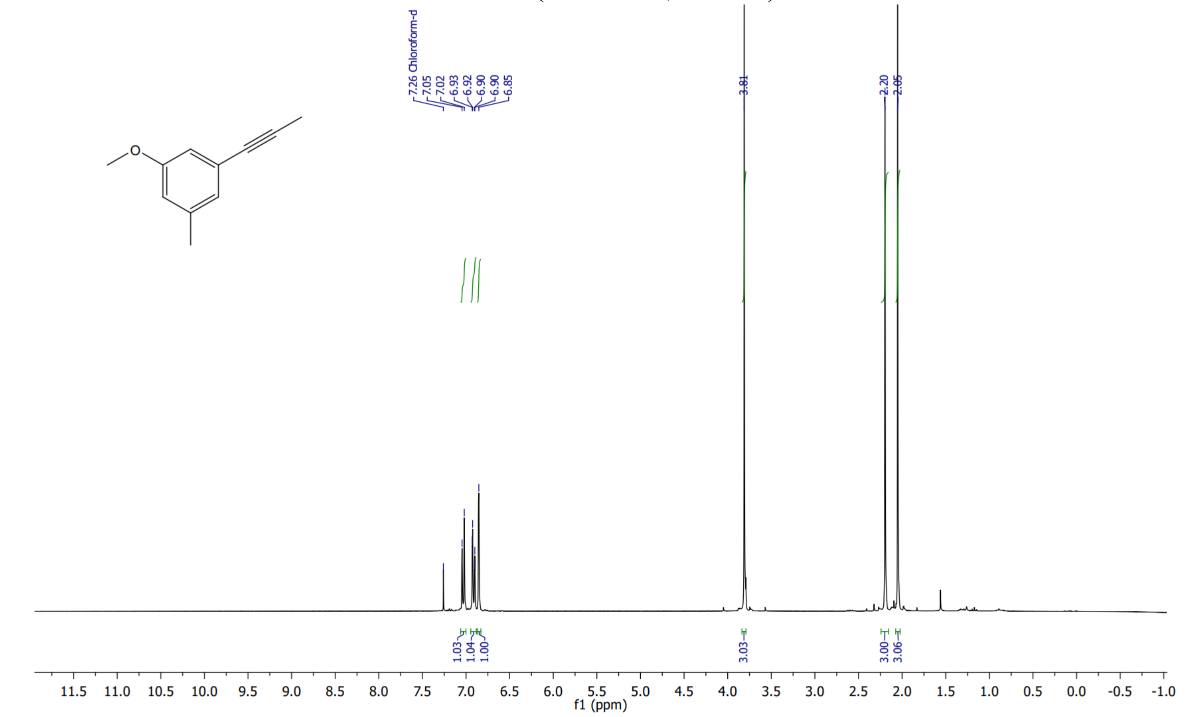

^{13}C NMR (75 MHz, $CDCl_3$)

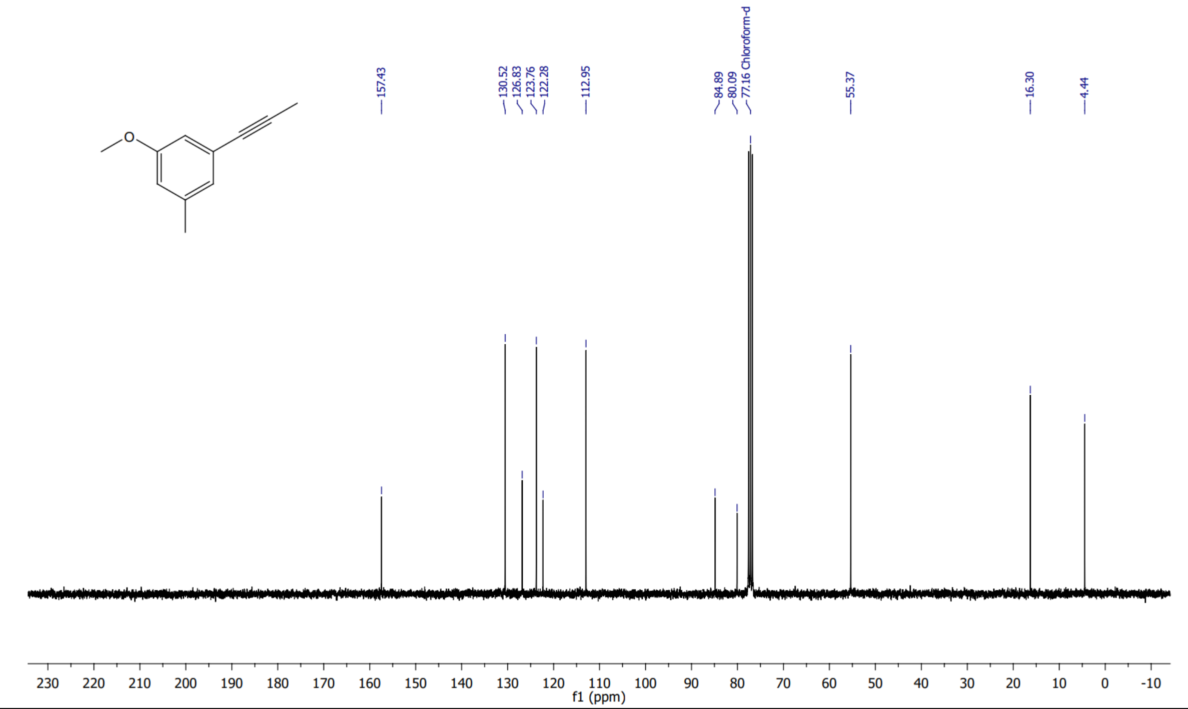

References

1. R. Alkahtani and T. Wirth, *ACS Org. Inorg. Au*, 2023, **3**, 209–216.
2. T. Lussier and C. Y. Legault, *Org. Lett.*, 2023, **25**, 2825–2829.
3. J. Renner, A. Thakur, P. M. Rutz, J. M. Cowley, J. L. Evangelista, P. Kumar, M. B. Prater, R. M. Stolley and J. Louie, *Org. Lett.,* 2020, **22**, 924–928.
4. W. Zhu, X. Zhen, J. Wu, Y. Cheng, J. An, X. Ma, J. Liu, Y. Qin, H. Zhu, J. Xue and X. Jiang, *Nat. Commun.* 2021, **12**, 3957.
5. Y. Yoshida, A. Magara, T. Mino and M. Sakamoto, *Tetrahedron Lett.*, 2016, **57**, 5103–5107.
6. T. Hokamp and T. Wirth, *Chem. Eur. J.*, 2020, **26**, 10417–10421.
7. M. Shimogaki, M. Fujita and T. Sugimura, *Eur. J. Org. Chem.*, 2013, **2013**, 7128–7138.
8. M. Uyanik, T. Yasui and K. Ishihara, *Angew. Chem. Int. Ed.*, 2010, **49**, 2175–2177.
9. C. B. Pandey, T. Azaz, R. S. Verma, M. Mishra and J. L. Jat, *J. Org. Chem.*, 2020, **85**, 10175–10181.
10. B. Basdevant and C. Y. Legault, *Org. Lett.*, 2015, **17**, 4918–4921.
11. U. Farid and T. Wirth, *Angew. Chem. Int. Ed.*, 2012, **51**, 3462–3465.
12. J. A. Cabezas and N. Ferllini, *Synthesis*, 2020, **52**, 2387–2394.
13. N. Phadke and M. Findlater, *Molecules*, 2015, **20**, 20195–20205.
14. W. Zhang, S. Kraft and J. S. Moore, *J. Am. Chem. Soc.*, 2004, **126**, 329–335.
15. S. Mehta and R. C. Larock, *J. Org. Chem.*, 2010, **75**, 1652–1658.
16. J. Santandrea, C. Minozzi, C. Cruché and S. K. Collins, *Angew. Chem. Int. Ed.*, 2017, **56**, 12255–12259.
17. Y.-Y. Liu, Y.-Y. Liu, X.-H. Yang, X.-C. Huang, W.-T. Wei, R.-J. Song and J.-H. Li, *J. Org. Chem.*, 2013, **78**, 10421–10426.
18. J. Renner, A. Thakur, P. M. Rutz, J. M. Cowley, J. L. Evangelista, P. Kumar, M. B. Prater, R. M. Stolley and J. Louie, *Org. Lett.*, 2020, **22**, 924–928.
19. S. Liu, J. Sawicki and T. G. Driver, *Org. Lett.*, 2012, **14**, 3744–3747.
20. J. Peng, F. Wu, A. Spannenberg and X. Wu, *Chem. Eur. J.*, 2019, **25**, 8696–8700.
21. H. Chiu and I. A. Tonks, *Angew. Chem. Int. Ed.*, 2018, **57**, 6090–6094.
22. M. M. Cencer, A. J. Greenlee and J. S. Moore, *J. Am. Chem. Soc.*, 2020, **142**, 162–168.
23. F. A. Cruz and V. M. Dong, *J. Am. Chem. Soc.*, 2017, **139**, 1029–1032.
24. R. T. Davison, P. D. Parker, X. Hou, C. P. Chung, S. A. Augustine and V. M. Dong, *Angew. Chem. Int. Ed.*, 2021, **60**, 4599–4603.
25. K. Kobayashi, Y. Yamamoto and N. Miyaura, *Organometallics*, 2011, **30**, 6323–6327.
26. N. A. Strotman, S. Sommer and G. C. Fu, *Angew. Chem. Int. Ed.*, 2007, **46**, 3556–3558.
27. J. I. Lee, *J. Korean Chem. Soc.*, 2017, **61**, 125–128.
28. M. S. Islam, S. Ahmad, M. R. Attu, F. H. Foerstering and M. M. Hossain, *Helv. Chim. Acta*, 2015, **98**, 1273–1286.
29. Q. Zhu and D. G. Nocera, *J. Am. Chem. Soc.*, 2020, **142**, 17913–17918.

30. S. Duez, S. Bernhardt, J. Heppekausen, F. F. Fleming and P. Knochel, *Org. Lett.*, 2011, **13**, 1690–1693.

31. A. H. Mermerian and G. C. Fu, *J. Am. Chem. Soc.*, 2005, **127**, 5604–5607.

32. J. S. Harvey, S. P. Simonovich, C. R. Jamison and D. W. C. MacMillan, *J. Am. Chem. Soc.*, 2011, **133**, 13782–13785.

33. F. Neese, *Wiley Interdiscip. Rev. Comput. Mol. Sci.*, 2022, **12**, e1606.

34. F. Neese, *Wiley Interdiscip. Rev. Comput. Mol. Sci.*, 2018, **8**, e1327.

35. F. Neese, *Wiley Interdiscip. Rev. Comput. Mol. Sci.*, 2012, **2**, 73–78.

36. F. Neese, F. Wennmohs, U. Becker and C. Riplinger, *J. Chem. Phys.*, 2020, **152**, 224108.

37. A. D. Becke, *J. Chem. Phys.*, 1993, **98**, 5648–5652.

38. C. Lee, W. Yang and R. G. Parr, *Phys. Rev. B Condens. Matter*, 1988, **37**, 785–789.

39. S. H. Vosko, L. Wilk and M. Nusair, *Can. J. Phys.*, 1980, **58**, 1200–1211.

40. P. J. Stephens, F. J. Devlin, C. F. Chabalowski and M. J. Frisch, *J. Phys. Chem.*, 1994, **98**, 11623–11627.

41. E. Caldeweyher, C. Bannwarth and S. Grimme, *J. Chem. Phys.*, 2017, **147**, 034112.

42. E. Caldeweyher, S. Ehlert, A. Hansen, H. Neugebauer, S. Spicher, C. Bannwarth and S. Grimme, *J. Chem. Phys.*, 2019, **150**, 154122.

43. E. Caldeweyher, J.-M. Mewes, S. Ehlert and S. Grimme, *Phys. Chem. Chem. Phys.*, 2020, **22**, 8499–8512.

44. F. Weigend and R. Ahlrichs, *Phys. Chem. Chem. Phys.*, 2005, **7**, 3297.

45. B. P. Pritchard, D. Altarawy, B. Didier, T. D. Gibson and T. L. Windus, *J. Chem. Inf. Model.*, 2019, **59**, 4814–4820.

46. D. Feller, *J. Comput. Chem.*, 1996, **17**, 1571–1586.

47. K. L. Schuchardt, B. T. Didier, T. Elsethagen, L. Sun, V. Gurumoorthi, J. Chase, J. Li and T. L. Windus, *J. Chem. Inf. Model.*, 2007, **47**, 1045–1052.

48. K. A. Peterson, D. Figgen, E. Goll, H. Stoll and M. Dolg, *J. Chem. Phys.*, 2003, **119**, 11113–11123.

49. M. Garcia-Ratés and F. Neese, *J. Comput. Chem.*, 2020, **41**, 922–939.

50. V. Barone and M. Cossi, *J. Phys. Chem. A*, 1998, **102**, 1995–2001.

51. M. Cossi, N. Rega, G. Scalmani and V. Barone, *J. Comput. Chem.*, 2003, **24**, 669–681.

52. H. Ryu, J. Park, H. K. Kim, J. Y. Park, S.-T. Kim and M.-H. Baik, *Organometallics*, 2018, **37**, 3228–3239.

53. F. Neese, *Chem. Phys. Lett.*, 2000, **325**, 93–98.

54. F. Neese, *J. Comput. Chem.*, 2023, **44**, 381–396.

55. F. Neese, *J. Comput. Chem.*, 2003, **24**, 1740–1747.

56. B. Helmich-Paris, B. de Souza, F. Neese and R. Izsák, *J. Chem. Phys.*, 2021, **155**, 104109.

57. F. Neese, F. Wennmohs, A. Hansen and U. Becker, *Chem. Phys.*, 2009, **356**, 98–109.

58. R. Izsák and F. Neese, *J. Chem. Phys.*, 2013, **139**, 094111.

59. R. Izsák, A. Hansen and F. Neese, *Mol. Phys.*, 2012, **110**, 2413–2417.

60. D. Bykov, T. Petrenko, R. Izsák, S. Kossmann, U. Becker, E. Valeev and F. Neese, *Mol. Phys.*, 2015, **113**, 1961–1977.

61. F. Weigend, *Phys. Chem. Chem. Phys.*, 2006, **8**, 1057–1065.

62. B. de Souza, *Angew. Chem. Int. Ed.*, 2025, **64**, e202500393.

63. C. Bannwarth, S. Ehlert and S. Grimme, *J. Chem. Theory Comput.*, 2019, **15**, 1652–1671.
